# Pulse Programme Considerations for Quantitative NOE Analysis

**DOI:** 10.1002/mrc.70127

**Published:** 2026-06-22

**Authors:** Lianne H. E. Wieske, Gintarė Lygnugarytė, Máté Erdélyi

**Affiliations:** ^1^ Department of Chemistry for Life Sciences Uppsala University Uppsala Sweden; ^2^ Center of Excellence for the Chemical Mechanisms of Life Uppsala University Uppsala Sweden

**Keywords:** distance determination, NMR, NOESY, ROESY, solvent suppression, structure elucidation

## Abstract

Accurate NOE/ROE‐based distance determination is a crucial tool in stereochemistry and conformational analysis of both protein and small molecule structure elucidation. Despite being widely used in countless fields of chemistry and biology, the factors determining the accuracy of the obtained distances are sometimes neglected. Herein, we present the systematic analysis of the performance of six commonly used NOESY and ROESY pulse programmes for quantitative NOE/ROE analysis, evaluating the quality of the data based on the number of quantifiable correlations and the accuracy of the corresponding NOE‐derived interproton distances. The effect of two solvent suppression schemes on the NOE/ROE quantitativity is assessed. We found that the total number of quantifiable correlations and the errors of the derived distances depend on the pulse programme used, and the error increases drastically when two or more spectra are removed from the build‐up curves for the generation of NOE/ROE build‐up rates, using the initial‐rate approximation. Solvent suppression using excitation sculpting results in the underestimation of the NOE‐derived distances. EASY ROESY run without solvent suppression shows a bias towards overestimated NOE‐derived distances. The obtained data suggest that the determination of quantitative interproton distances necessitates the careful selection of pulse programme.

## Graphical abstract

NOESY and ROESY spectra are frequently used to obtain interatomic distances in solution. Here, the performance of six routinely used pulse programmes is evaluated, with the number of quantified correlations and the accuracy of the NOE‐based distances being used as the main assessment criteria.

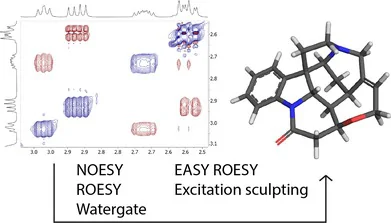

## 1 Introduction

Solution NMR is uniquely capable of capturing the conformational ensembles of dynamic molecules. It is frequently used in the structure elucidation of small organic compounds [1, 2, 3] and of biopolymers, such as proteins [4, 5]. Over the past decade, it has received special interest to assess molecular chameleonicity to explain passive membrane permeability of beyond Rule of Five drugs [6, 7, 8, 9]. Among the available NMR observables, the nuclear Overhauser effect (NOE) [10, 11] is the most ubiquitous in conformational studies as it may provide quantitative internuclear distances. Whereas other approaches also exist, NOEs are commonly quantified using the initial‐rate approximation [5, 12, 13, 14], especially for rapidly tumbling small molecules. Here, the integrals of NOE cross‐peaks in spectra acquired with different mixing times are quantified to determine the build‐up rate of specific NOEs that are subsequently converted into internuclear distances. Some use the simplification of only acquiring one NOESY or ROESY spectrum at a single mixing time. Although a plethora of NOESY and ROESY pulse programmes and strategies are available, the understanding of their limitations for obtaining quantitative internuclear distances remains sparse. Accordingly, pulse programme selection for quantitative studies is often based on rumours or convenience, without a solid understanding of the limitations. In order to bridge this gap, we provide herein a comparative analysis of data obtained on the same sample using a selection of the most commonly used pulse programmes.

**FIGURE 1:**
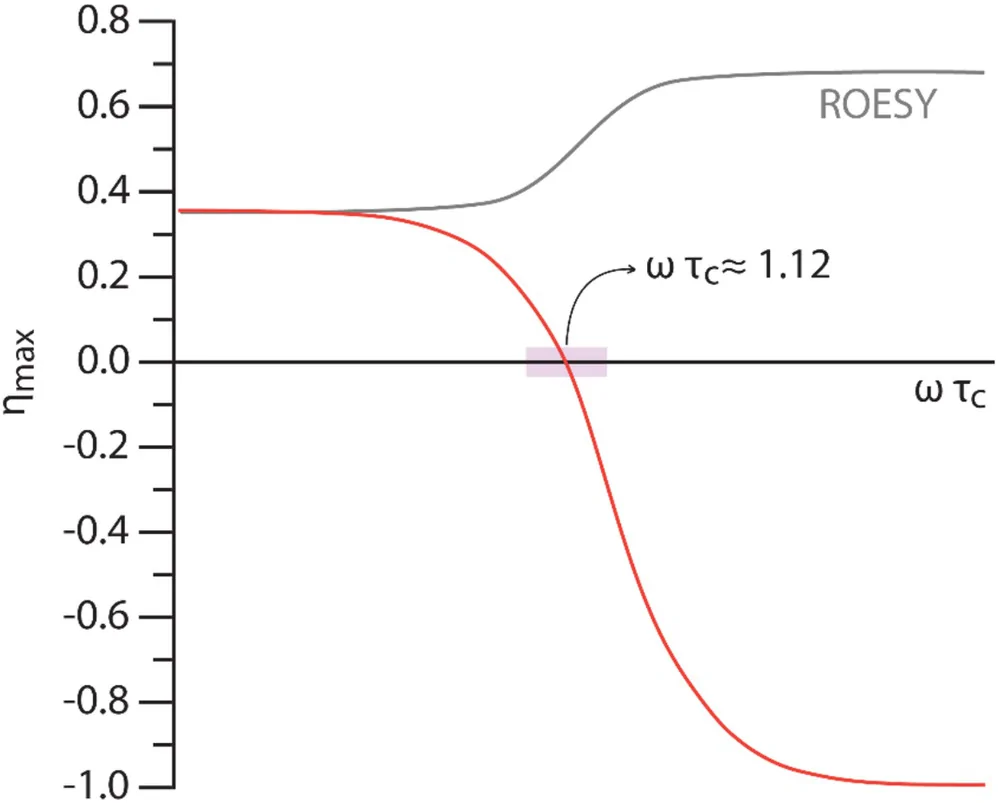
The maximum NOE and ROE enhancement as a function of the correlation time (*τ*_c_) and spectrometer frequency (*ω*).

The size and sign of the NOE cross‐peaks depend on the correlation time (*τ*_c_) of the molecule and on the spectrometer frequency (*ω*) [15]. Large compounds typically tumble slowly in solution and show NOE enhancements close to −100% (Figure 1) [13]. Small compounds, especially in nonviscous solvents, usually tumble rapidly and experience a positive NOE enhancement that may reach up to +38%. Data can be obtained in between these extremes; however, there is a zero‐crossover point where molecules experience a net zero NOE enhancement at a given spectrometer frequency, making the detection and quantification of cross‐peaks difficult. One may move away from the zero‐crossover region by altering the dynamics of the system, either by changing the solvent (viscosity) or the temperature, or by changing spectrometer (frequency). Another option may be the detection of the rotating frame Overhauser effects (ROEs), which do not have a zero‐crossover point as they always are positive, varying from 38% to 68%, dependent of the molecular tumbling rate (Figure 1) [16]. The use of ROESY instead of NOESY, however, may introduce offset dependence and artefacts (TOCSY), for instance, that may have an influence on quantitativity. It should be noted that NOESY spectra are not always free from artefacts either, however, they are of different nature (zero‐quantum peaks, with a DQF‐COSY type pattern) [17]. Such artefacts may jeopardise quantitativity.

Several factors may affect the appearance of the spectrum and its quantitativity. Recording spectra with nonuniform sampling (NUS) schemes might result in improved S/N as compared with uniformly sampled spectra recorded for the same time‐period [18, 19]. However, implementing NUS schemes often results in reduced accuracy of the obtained interproton distances and tends to introduce artefacts [20, 21]. Viscous solvents tend to result in larger errors on the NOE‐derived distances [2]. Another important parameter that affects the appearance and quality of spectra is the relaxation delay (*d1*). Having a d1 of three to five times *T*_1_ allows for near full recovery of the magnetisation (99.33%) and accordingly in the most intense and quantitative NOEs. However, using such ad1 delay would require weeks of spectrometer time for small molecules with *T*_1_ ~ 3–5 s, which is not feasible. Using a relaxation delay of one to two times *T*_1_ instead allows for quantitative NOE analysis by creating a steady state [22]. The quantification process can generally either be achieved through the initial‐rate approximation or full matrix approaches [23]. For compounds with limited variability in internal flexibility, the initial‐rate approximation offers a robust method, with distance errors generally below 10% for 2D spectra [2, 3], and below 20% for 3D experiments [24]. Strongly coupled spins may lead to zero‐quantum artefacts [17]. Pulse programmes developed to circumvent such issues tend to give better result for short than for long‐range interactions [25]. Despite extensive efforts to understand the factors influencing quantitative NOE analysis, and provide improvements, the comparison of the performance of pulse sequences and the influence of solvent suppression on quantitativity is missing.

When studying systems with exchangeable protons (amide, amine, alcohol, thiol) in protic solvents, such as water, the need for solvent suppression arises when one wishes to quantify these exchangeable protons as well. This is to date most commonly achieved by excitation sculpting [26], which makes use of a double pulse‐field‐gradient spin‐echo, or with watergate [27], which uses a selective 180° and two field gradient pulses instead. The influence of solvent suppression on the quantitativity of NOE‐experiments is not only relevant for the exchangeable protons, but might affect nonexchangeable protons as well.

Motivated by the importance of accurate internuclear distances for solution structure determination, we evaluate the applicability of some of the most commonly used NOESY and ROESY pulse‐sequences, available in the Bruker Topspin pulse programme library, for obtaining accurate interproton distances. We recorded NOESY/ROESY build‐up curves, with 7 mixing times each, with the pulse programmes noesyph [12, 28] (phase‐sensitive NOESY), noesyesgpph [26] (NOESY using excitation sculpting (ES)), noesydbphw5 [29, 30] (NOESY W5, using watergate solvent suppression), roesyph [16, 31] (phase‐sensitive ROESY) and roesyadesjsph [17, 26, 32, 33] (EASY ROESY without solvent suppression) and roesyadesjsph (EASY ROESY with solvent suppression using ES). Hence, noesyph is the most basic NOESY sequence, which contains three pulses only and does not incorporate any additional filters. It is short and simple. Among the pulse sequences employing solvent suppression, noesyesgpph is currently the most commonly applied in literature. In short, excitation sculpting [26] utilises a solvent‐selective double spin echo building block with 180° solvent‐selective pulses (ES element). A first frequency‐selective pulse is applied to invert the magnetisation of the targeted molecules while leaving the solvent unaffected. Next, the unwanted transverse magnetisation is dephased by frequency selective pulse‐field gradient, a second selective 180° pulse flips the desired magnetisation back to the z‐axis, and a subsequent second gradient is used to restore the solute signals and simultaneously cancel the uninverted solvent signal. The use of a double pulsed‐field gradient spin‐echo ensures to avoid phase errors. The noesydbphw5 pulse sequence uses a DANTE‐based pulse train (binomial W5 element) that inverts the solute signals but leaves the solvent signal unperturbed. The net effect of the sandwiching identical gradient pair is effectively zero on the inverted signals, and hence these are retained. In contrast, the not inverted solvent signal is dephased by the gradients. The W5 element encompasses a series of varying flip angle hard pulses with a fixed interpulse delay, *τ*. The suppression bandwidth is inversely proportional to this delay. If not optimised, it may cause suppression not only on resonance but also at intervals of 1/*τ* Hz. This may become an issue when a narrow suppression band is required as a long interpulse delay *τ* may lead to additional suppression notches, potentially influencing the intensity of some solute signals. The roesyph sequence is the equivalent of the simplest NOESY sequence, noesyph. In ROESY, the cross‐relaxation occurs under the effect of a spin‐lock that is weaker than the one used in TOCSY experiments. ROESY is surprisingly often used for quantitative purposes by nonexperts, despite its long‐known weaknesses, including the nonuniform enhancement of signals by its spin‐lock, the appearance of in‐phase TOCSY artefacts that undistinguishable from and often overlap with the ROE peaks, and the appearance of mixed TOCSY‐ROE transfers yielding ‘false’ cross‐correlation peaks [34]. The EASY ROESY (roesyadesjsph) pulse programme was developed to compensate for the above weaknesses of ROESY [32]. It uses two off‐resonance spin‐lock pulses, applied to the high‐ and low‐frequency regions of the spectral window, sandwiched in between adiabatic pulses. The use of two spin‐lock pulses, applied outside of the spectral window, is aimed to minimise TOCSY transfers and offset dependence, improving quantitativity. Spectra were acquired on a sample of strychnine, a commonly used model compound with limited and well‐documented flexibility [35, 36], in DMSO‐*d*_6_. Strychnine was used as it is a bioactive natural product commonly applied in the evaluation of the performance of NMR methods [2, 18, 35, 37, 38]. The solution ensemble of strychnine determined by Butts et al. [35] was used as reference for comparison to the obtained interproton distances. Being a quickly tumbling small molecule, the NOEs detected for strychnine in DMSO‐*d*_6_ were positive (when phasing the diagonal negative).

## 2 Experimental

Spectra were recorded on a 500‐MHz Bruker AVANCE NEO spectrometer equipped with a 5‐mm TXO cryogenic probe. A 25.5‐mM strychnine sample was prepared in DMSO‐*d*_6_ (6 mg in 700 μL), and spectra were recorded at 298 K (Figure S1 and Table S1). In total six different data‐sets were recorded using the pulse sequences noesyph [12, 28], noesyesgpph [26], noesydbphw5 [29, 30], roesyph [16, 31], and roesyadesjsph [17, 26, 32, 33] with and without solvent suppression, as available in the Bruker pulse‐programme library. To achieve the roesyadesjsph pulse programme without solvent suppression, the power level of shaped pulse 10 (sp10) was set to zero.

A series of spectra was run with mixing times 100–700 ms with 100‐ms intervals for the NOESY sequences, and 50–350 ms for the spin‐lock pulses with 50‐ms intervals for the ROESY sequences. A 2.5‐s relaxation delay was applied. An integration matrix was generated from manually integrated diagonal peaks observed in the spectrum obtained with the highest mixing time. After manual integration of the diagonal peaks, a matrix was generated to integrate each cross‐peak matching the chemical shift boundaries of the diagonal peaks. In a parallel process, to evaluate the impact of the selection of integration area, the cross‐peak integrals were adjusted to only include parts of the cross‐peaks that contain signal in the spectra corresponding to the lowest mixing time. This was done to minimise the noise included into the cross‐peak integrals. Cross‐peak intensities were normalised to the diagonal according Equation (1), where *I*_*ab*_ is the normalised cross‐peak intensity between *H*_*a*_ and *H*_*b*_, *CP*_*ab*_ and *CP*_*ba*_ are the cross‐peak intensities on each side of the diagonal, and *DP*_*a*_ and *DP*_*b*_ are the diagonal peak intensities.

(1) $$I_{\mathrm{ab}}=\sqrt{\frac{{\mathrm{CP}}_{\mathrm{ab}}\times {\mathrm{CP}}_{\mathrm{ba}}}{{\mathrm{DP}}_{a}\times {\mathrm{DP}}_{b}}}$$

The build‐up rates (*σ*) for each interproton correlation were derived from the linear regression between the normalised intensities (*I*_*ab*_) and the mixing times (*t*_*mix*_), without forcing the fit through the origin (0,0), allowing for an offset (*b*) Equation (2).

(2) $$I_{\mathrm{ab}}=\sigma \times t_{\mathrm{mix}}+b$$

The coefficient of determination (*R* ^2^) [39] was calculated for each linear fit that included at least four data‐points (spectra obtained with different mixing times), and those with an *R* ^2^ of ≥ 0.95 were included in further analysis without adjustments. For the build‐up curves with an *R* ^2^ < 0.95, data‐points from different mixing times were excluded systematically one at the time, and the linear regression and *R* ^2^ was recalculated. Once an *R* ^2^ ≥ 0.95 was reached, no further adjustments to the build‐up curves were made. Data‐points were only excluded if the *R* ^2^ of the build‐up curve was below 0.95 and the outlier data‐point deviated by at least the mean plus 1 standard deviation from the linear fit of the original data‐set (Figure S2). The normalised intensity from the mixing time that deviated most from the linear fit was excluded. This process was repeated until an *R* ^2^ ≥ 0.95 was reached. Build‐up curves with less than four mixing times independent of the *R* ^2^‐value were discarded. Build‐up rates derived from four or more data‐points and an *R* ^2^ ≥ 0.95 were further analysed. The interproton distances of the selected correlations were determined (based on both cross‐peaks and diagonal peaks), using the initial‐rate approximation [1, 14, 40]. The build‐up rate of the proton‐pair at position 15 (*σ*_ref_, Figure S1) was used to calibrate the interproton distances and was set to 1.76 Å (*r*_ref_) [35]. Interproton‐distances (*r*_*ab*_, for protons A and B) were calculated according Equation (3).

(3) $$r_{\mathrm{ab}}=1.76\times \sqrt[{6}]{\frac{\sigma_{\mathrm{ref}}}{\sigma_{\mathrm{ab}}}}$$

Once the distances were quantified, they were sorted based on their theoretically expected distance range from 1.50 to 10.00 Å, using 0.5‐Å intervals. The distances were sorted according to their expected value, [26] so that each distance group contained the same set of correlations for each pulse programme. The expected distances were based on the population‐averaged ensemble of strychnine consisting of two conformations, a major one present for 97.5% and a minor for 2.5% [35]. Distances to overlapping signals (23a/23b and 11b/20b) were treated as overlapping according to Equation (4), rather than averaged signals [41]. The deviation from the expected theoretical distance, by subtracting the theoretical value from the experimental value, as well as the corresponding absolute deviation and absolute error (%) (Tables S17–S22) were determined for each proton pair in each set of spectra.

(4) $$r_{a\left(\mathrm{bc}\right)}={\left({r_{\mathrm{ab}}}^{-6}+{r_{\mathrm{ac}}}^{-6}\right)}^{-\frac{1}{6}}$$

In parallel, the integral values obtained from the integration matrices of the NOESY spectra were reanalysed, where the unadjusted normalised intensities of each spectrum were used to calculate the interproton distance (Equation S1, Supporting Information pp. S48–S55). Once the distances were determined for each mixing time, they were treated as described above. The accuracy of the distances obtained from the normalised intensity (single spectrum) recorded at various mixing times was critically assessed.

Errors on the NOE‐based distances were determined by looking at the absolute difference between theoretical and calculated distance (|Experimental distance − Theoretical distance|). Averaged absolute errors, where the theoretical distances serve as the 100% reference point for the absolute differences, were calculated for grouped correlations (such as distance ranges). Therefore, averaged absolute errors are defined according to Equation (5), where *n* is the number of correlations included in a given grouped set of correlations, *TD* refers to theoretically determined distance, and *ED* corresponds to experimentally determined NOE‐based distance.

(5) $$\text{Average absolute error}=\frac{\sum_{i=1}^{n}\left(\left(100/\mathrm{TD}\right)\times |\mathrm{ED}.-\mathrm{TD}|\right)}{n}$$

## 3 Results

When assessing the performance of pulse programmes, the number of inter‐proton distances following standard protocols (for details, see Section 2) was used as a first indicator, desiring a high number of distances within and a low number of distances beyond the NOE‐reach. Next, the accuracy of the distances was determined. Here, fewer but more accurate distances was seen as favourable over a larger number of less accurate distances. Finally, the correlations were reanalysed by grouping these same correlations based on the number of data‐point (spectra with different mixing‐times) that were included in the linear regression. This was done to assess whether the number of data‐points used to calculate the build‐up rate would have an observable influence on the accuracy of the distances. NOE build‐up rates were converted into interproton distances (Equation 3) and were grouped covering a 0.50‐Å distance ranges. Distances around the NOE‐detection limit (~5.00 Å) might not be readily detectable, and we therefore used an upper limit of 4.49 Å for distances that were expected to be quantified. As those beyond 5.50 Å are most certainly artefacts, they are referred to as ‘artificial distances’ below.

### 3.1 Impact of the Integration Area of the Cross‐Peaks

At assessing the quality of the acquired data, cross‐peaks were integrated in two different ways: (i) In a first approach, the size of the cross‐peak integrals were matched to the size of the diagonal‐peaks. (ii) In a second one, the size of the cross‐peak integrals was adjusted to the peak centre in the spectrum obtained with lowest mixing time to minimise the inclusion of noise.

Adjusting the integration area of the cross‐peaks based on the spectra with the lowest mixing times (ii) resulted in 7.5% fewer interproton distances on average as compared with the analysis without such an adjustment (i). The number of analysed interproton distances were the least affected for the NOESY, NOESY ES, NOESY W5, ROESY and EASY ROESY sequences without solvent suppression and most for the EASY ROESY ES, however, the error of the calculated distances significantly increased upon the adjustment of the integral areas for all data‐sets by 2.5%–14% (5.5% on average). The numbers of interproton distances with and without the above adjustment are given in Tables S17–S22. As the adjustment of the integral areas to the size of the cross‐peaks at the lowest mixing time, to minimise the effect of integrated noise, decreased data quality, only the NOE build‐up curves for which no such adjustment (approach i) has been performed were further analysed.

### 3.2 Impact of Using the Normalised Intensities From One NOESY Spectrum (Single Mixing Time)

Distances derived by integrating the cross‐peaks in a single NOESY spectrum recorded at a given mixing time resulted in distances with large errors and the inclusion of artefacts (Tables S23 and S24), that is, peaks that are not NOE correlations (artificial distances). Distances derived from spectra recorded with 100 or 200 ms mixing times showed 5.5%–44.0% error, independent of the distance range (Tables 1 and S29). For data derived from spectra recorded with 300‐ to 700‐ms mixing time, distances up to 3.00 Å had average absolute errors below 10%. However, the long‐range correlations > 3.00 Å that are the most informative for structure determination processes showed 10%–45% average absolute error. A large number of the observed correlations, 70 out of the 190 integrated cross‐peaks, were well beyond the NOE distance‐limit (Table S23). Their inclusion into a subsequent analysis would obviously distort the determined structure. Even though removing correlations for which the cross‐peaks show the opposite phase (something that would not happen for a real NOE cross‐peak) and excluding distances that are calculated to be beyond 5.50 Å significantly reduced the number of artificial distances, the accuracy of the remaining data is poor (up to 68% absolute error in the determined distances, Supporting Information pages S48–S76, Tables S25–S28, S30 and S31). Distances derived from spectra acquired with a low mixing time, 100 ms, showed larger distance errors (Table 1) than those acquired with 200‐ to 700‐ms mixing times. This is likely the consequence of the low signal per noise of the cross‐peaks when detecting correlations at the very beginning of the build‐up curves. Excessively long mixing times are expected to result in distances with a large error due to other relaxation mechanisms, such as *T*_*2*_. It should be kept in mind that distances determined by integrating a NOESY spectrum with a single mixing time are unsuitable for structure determination, independent of the mixing time.

**TABLE 1:** The total number of distances found when quantifying the correlations from a single NOESY spectrum at a given mixing time (100–700 ms) and their corresponding average absolute error (%) in between brackets separated on distance range.

	Distance range (Å)	Theoretically available	100	200	300	400	500	600	700
	All < 5.50 Å	120	190 (23.5%)	190 (15.2%)	190 (14.3%)	190 (15.0%)	190 (14.3%)	190 (14.5%)	190 (12.2%)
Short reliable	0.00–2.99	34	34 (30.4%)	34 (9.7%)	34 (6.4%)	34 (4.5%)	34 (4.3%)	34 (4.1%)	34 (4.1%)
Long reliable	3.00–4.49	44	44 (28.3%)	44 (14.0%)	44 (14.8%)	44 (19.7%)	44 (17.3%)	44 (15.9%)	44 (12.3%)
At the NOE limit	4.50–5.49	42	42 (21.3%)	42 (13.1%)	42 (13.5%)	42 (16.6%)	42 (16.6%)	42 (15.2%)	42 (12.3%)
Beyond the NOE limit	5.55–9.99		70 (18.5%)	70 (19.9%)	70 (18.2%)	70 (16.3%)	70 (15.8%)	70 (18.1%)	70 (16.0%)

### 3.3 Impact of Excitation Sculpting

Distance quantification using the initial build‐up rate approximation is expected to result in a roughly equal distribution of number of distances that are over‐ and underestimated as compared with the theoretically expected distances (Tables S17–S22). This behaviour was observed for the spectra acquired with the NOESY (53.3% underestimated and 43.3% overestimated, and 3.4% exactly matched the expected interproton distances), NOESY W5 (44.4% underestimated and 54.2% overestimated), and ROESY (55.7% underestimated and 42.6% overestimated) pulse programmes, but not for those obtained with the NOESY ES, EASY ROESY and EASY ROESY ES pulse programmes. Spectra obtained with the EASY ROESY sequence without solvent suppression showed a tendency to overestimate the interproton distances (78.0% overestimated, and 16.7% underestimated). In contrast, the build‐up rates obtained using NOESY ES and EASY ROESY ES, both of which use excitation sculpting for solvent suppression, showed a trend to underestimate interproton distances (69.6% underestimated and 28.6% overestimated for NOESY ES, and 59.4% underestimated and 35.9% overestimated for EASY ROESY ES).

### 3.4 Impact of Pulse Programmes

**FIGURE 2:**
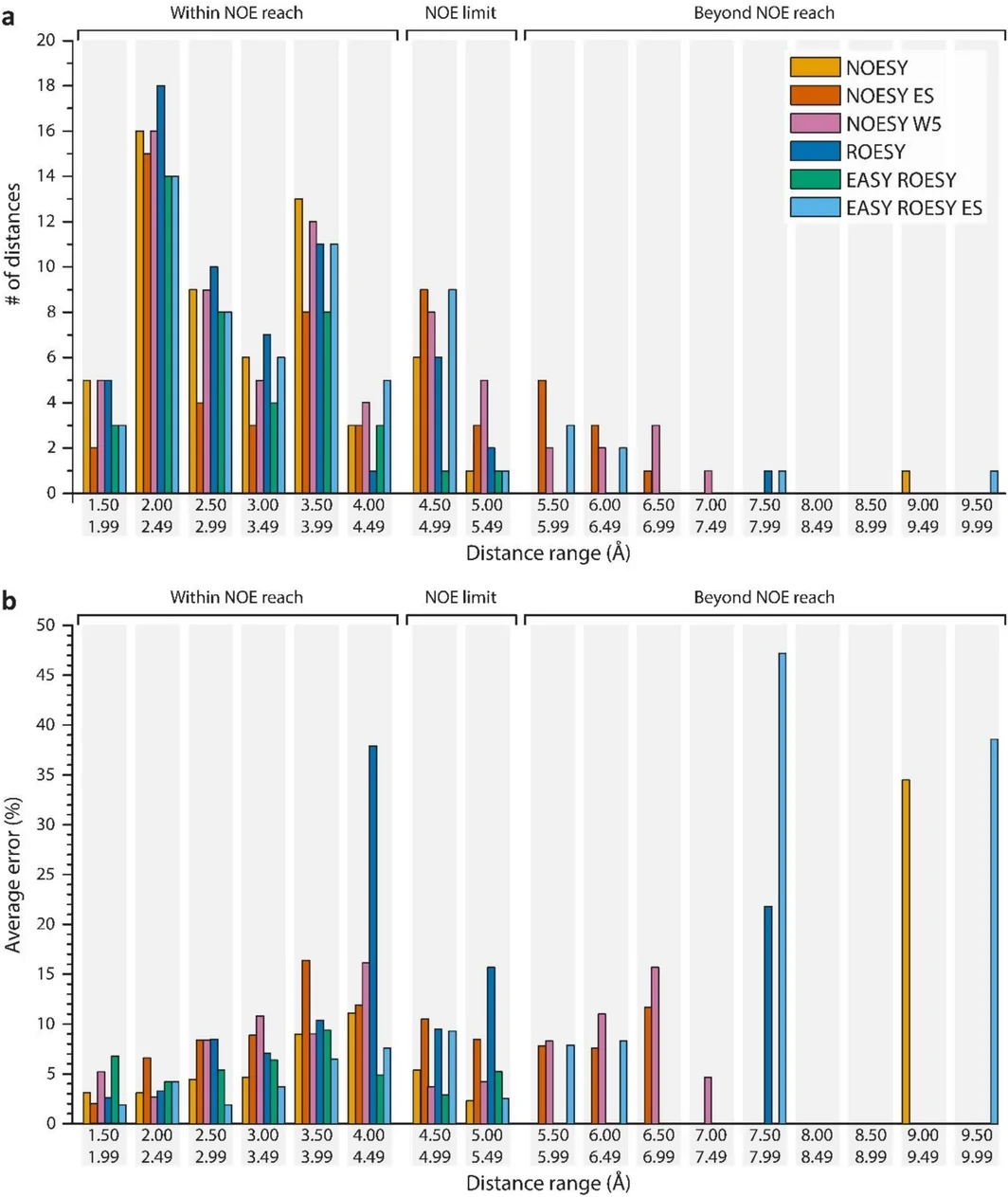
(a) The total number of correlations that could be quantified using the initial‐rate approximation for each pulse programme. It should be emphasised that a larger number of distances does not necessarily mean a better performance of a pulse sequence, as cross‐peaks may appear as the result of artefacts. (b) The average absolute error determined for the NOE‐derived distances. The correlations are clustered into their corresponding distance range as determined based on the time‐averaged solution ensemble of strychnine [26].

The number of correlations that could be quantified and their corresponding average absolute errors for the six data‐sets are shown in Figure 2. These include all obtained correlations, independent of the number of data‐points (spectra with various mixing times) that were included to determine the build‐up rates.

The largest number of quantifiable correlations up to 4.50 Å were found for the NOESY, NOESY W5 and ROESY pulse sequences, with 51 correlations for the NOESY W5 (65.4% of the total expected correlations) and 52 (66.7%) correlations for both NOESY and ROESY. The NOESY ES pulse sequence provided fewest correlations with a total of 35 distances only (44.8%).

All pulse programmes tested performed well for interproton distances up to 3.00 Å (Table 2 and Tables S2–S4), whereas > 10% average absolute errors of the interproton distances were observed for the data acquired with the NOESY ES, NOESY W5 and ROESY sequences at the distances range 3.00–4.50 Å.

**TABLE 2:** The total number of distances found and their corresponding average absolute error (%) in between brackets for each pulse programme separated on distance range. The numbers correspond to all correlations combined (independent of the number of spectra included to generate the build‐up curves).

	Distance range (Å)	Theoretically available	NOESY	NOESY ES	NOESY W5	ROESY	EASY ROESY	EASY ROESY ES
	All < 5.50 Å	120	59 (5.9%)	47 (9.4%)	64 (7.1%)	60 (7.7%)	42 (5.8%)	57 (6.6%)
Short reliable	0.00–2.99	34	30 (3.5%)	21 (6.5%)	30 (4.8%)	33 (4.8%)	25 (4.9%)	25 (3.2%)
Long reliable	3.00–4.49	44	22 (8.1%)	14 (13.8%)	21 (10.8%)	19 (10.7%)	15 (7.7%)	22 (6.0%)
At the NOE limit	4.50–5.49	42	7 (4.9%)	12 (10.0%)	13 (3.9%)	8 (11.1%)	2 (4.0%)	10 (8.6%)
Beyond the NOE limit	5.55–9.99		1 (34.5%)	9 (8.2%)	8 (11.3%)	1 (21.8%)	0	7 (18%)

Distances beyond 5.50 Å, which are most likely artefacts, were observed on more than one occasion for all pulse programmes using solvent suppression, whereas the spectra obtained without solvent suppression did not suffer from this type of artefacts.

Next, we split the obtained correlations into groups based on the number of mixing times that were included for the build‐up rate determinations (Table 3). Each group is analysed on its own, where only the correlations indicated in each row of Table 3 were considered and discussed. The first analysis includes NOE‐build‐up curves based on spectra from seven NOESY or ROESY mixing times with *R* ^2^ ≥ 0.95. Next, the NOE correlations for which one data‐point had to be excluded to yield and *R* ^2^ ≥ 0.95 of the build‐up curve, and hence include spectra from six mixing times are discussed. Subsequently we analysed build‐up curves for which two data‐points (five spectra included in the build‐up curve) were excluded to reach an *R* ^2^ ≥ 0.95, and finally, the correlations for which three data‐points (four spectra included in the build‐up curve) were removed to result in an *R* ^2^ ≥ 0.95 were analysed and discussed.

**TABLE 3:** The number of correlations covering all distance ranges coming from build‐up curves derived from 7, 6, 5 and 4 NOESY/ROESY spectra.

	NOESY	NOESY ES	NOESY W5	ROESY	EASY ROESY	EASY ROESY ES
All *t*_mix_ combined	60	56	72	61	42	64
Based on 7 *t*_mix_	32	21	35	35	23	29
Based on 6 *t*_mix_	7	13	7	8	6	13
Based on 5 *t*_mix_	6	9	13	10	7	7
Based on 4 *t*_mix_	15	13	17	8	6	15

#### 3.4.1 Accuracy of Distances Derived From Build‐Up Curves Based on Seven Mixing Times

**FIGURE 3:**
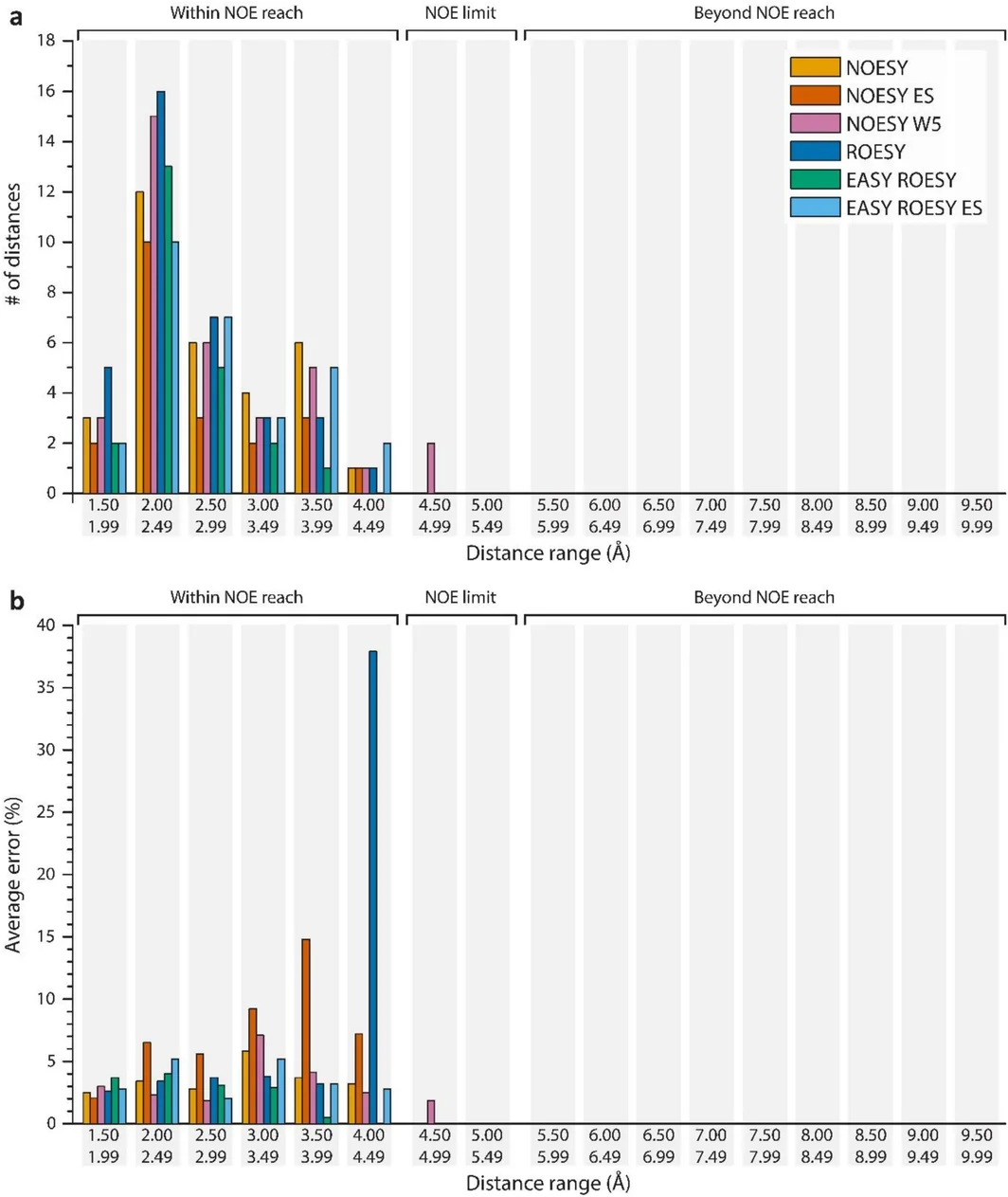
(a) Number of correlations that could be quantified using the initial‐rate approximation based on seven data‐points with an *R* ^2^ of ≥ 0.95 for each pulse programme. It should be emphasised that a larger number of distances does not necessarily mean a better performance of a pulse sequence, as cross‐peaks may appear as the result of an artefact. (b) The average absolute error determined for the NOE‐derived distances of those correlations based on seven data‐points with an *R* ^2^ of ≥ 0.95. The correlations are clustered into their corresponding distance range as determined based on the time‐averaged solution ensemble of strychnine [26].

The interproton distances that were quantified from build‐up rates based on seven data‐points with an *R* ^2^ of ≥ 0.95 and their corresponding errors are plotted in Figure 3.

The largest number of correlations for distances up to 4.50 Å were found by the NOESY, NOESY W5 and ROESY sequences (Table 4, Figure 3, Tables S5–S7). Each of these was able to quantify a total of 32–35 distances, whereas fewest correlations were found using NOESY ES (21) and EASY ROESY (23). Aside from a few outliers within the NOESY ES and ROESY data‐sets, the average absolute errors on the distances were generally below 10% for all pulse programmes. No artificial distances, ≥ 5.50 Å, were derived from the build‐up curves based on all seven spectra.

**TABLE 4:** Number of distances found for correlations based on 7 mixing times and their corresponding average absolute error (%) in between brackets for each pulse programme separated on distance range.

	Distance range (Å)	NOESY	NOESY ES	NOESY W5	ROESY	EASY ROESY	EASY ROESY ES
	All < 5.50 Å	32 (3.5%)	21 (7.4%)	35 (2.9%)	35 (4.4%)	23 (3.6%)	29 (3.8%)
Short reliable	0.00–2.99	21 (3.1%)	15 (5.7%)	24 (2.2%)	28 (3.3%)	20 (3.8%)	19 (3.8%)
Long reliable	3.00–4.49	11 (4.4%)	6 (11.7%)	9 (4.9%)	7 (8.4%)	3 (2.1%)	10 (3.7%)
At the NOE limit	4.50–5.49	0	0	2 (1.8%)	0	0	0
Beyond the NOE limit	5.55–9.99	0	0	0	0	0	0

#### 3.4.2 Accuracy of Distances Derived From Build‐Up Curves Based on Six Mixing Times

**FIGURE 4:**
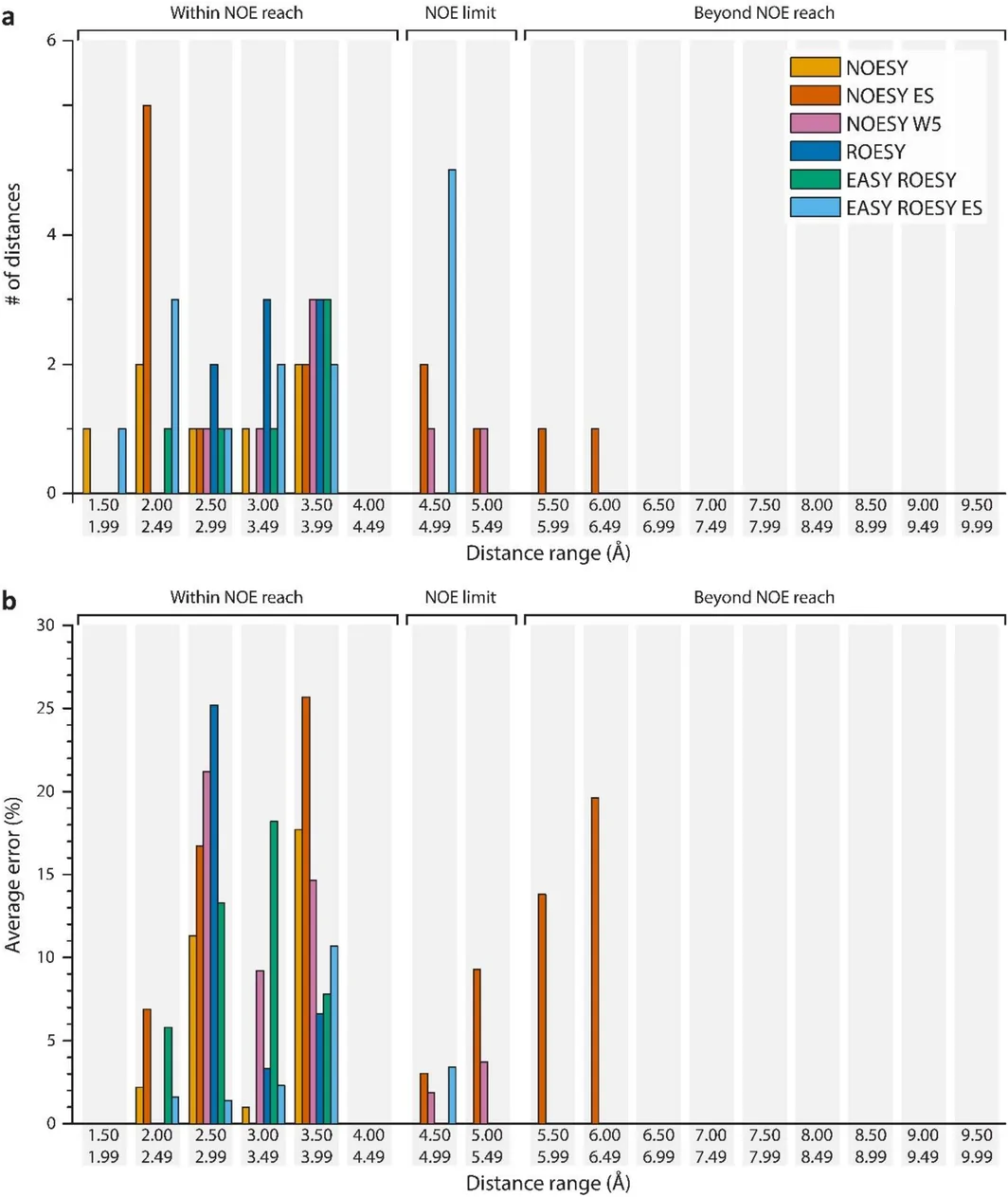
(a) Number of correlations that could be quantified using the initial‐rate approximation based on six data‐points with an *R* ^2^ of ≥ 0.95 for each pulse programme. It should be emphasised that a larger number of distances does not necessarily mean a better performance of a pulse sequence, as cross‐peaks may appear as the result of an artefact. (b) The average absolute error determined for the NOE‐derived distances of those correlations based on six data‐points with an *R* ^2^ of ≥ 0.95. The correlations are clustered into their corresponding distance range as determined based on the time‐averaged solution ensemble of strychnine [26].

In case an initial build‐up rate with an *R* ^2^ ≥ 0.95 could not be determined for a specific correlation using the integrals obtained from spectra with all seven mixing times, the outlier data‐point was excluded, as described above, and the initial build‐up rate was determined based on the data from the remaining six mixing times. A comparable number of correlations, 5–9, corresponding to distances up to 4.50 Å was found using the six pulse programmes looking at the group of correlations based on six data‐points (Figure 4, Table 5 and Tables S8–10). The errors of the calculated distances remained below 10% for most of these correlations up to 3.00 Å for the data‐sets acquired with the NOESY and EASY ROESY ES pulse programmes, but the error increased significantly beyond 3.00 Å. Except for the NOESY data‐set, errors well above 10% were frequently found for the tested pulse programmes. Larger errors on the distances within the NOESY data‐set were occasionally found, but these were outliers rather than recurring trends. In total two artificial distances, ≥ 5.50 Å, were found in the data‐set acquired with NOESY ES, and none for the data obtained with the other pulse programmes.

**TABLE 5:** Number of distances found for correlations based on 6 mixing times and their corresponding average absolute error (%) in between brackets for each pulse programme separated on distance range.

	Distance range (Å)	NOESY	NOESY ES	NOESY W5	ROESY	EASY ROESY	EASY ROESY ES
	All < 5.50 Å	7 (7.5%)	11 (11.6%)	7 (11.3%)	8 (10.0%)	6 (10.1%)	13 (3.5%)
Short reliable	0.00–2.99	4 (4.0%)	6 (8.5%)	1 (21.2%)	2 (25.2%)	2 (9.5%)	5 (1.2%)
Long reliable	3.00–4.49	3 (12.1%)	2 (25.7%)	4 (13.2%)	6 (5.0%)	4 (10.4%)	4 (6.5%)
At the NOE limit	4.50–5.49	0	3 (5.2%)	2 (2.8%)	0	0	4 (3.4%)
Beyond the NOE limit	5.55–9.99	0	2 (16.7%)	0	0	0	0

#### 3.4.3 Accuracy of Distances Derived From Build‐Up Curves Based on Five Mixing Times

**FIGURE 5:**
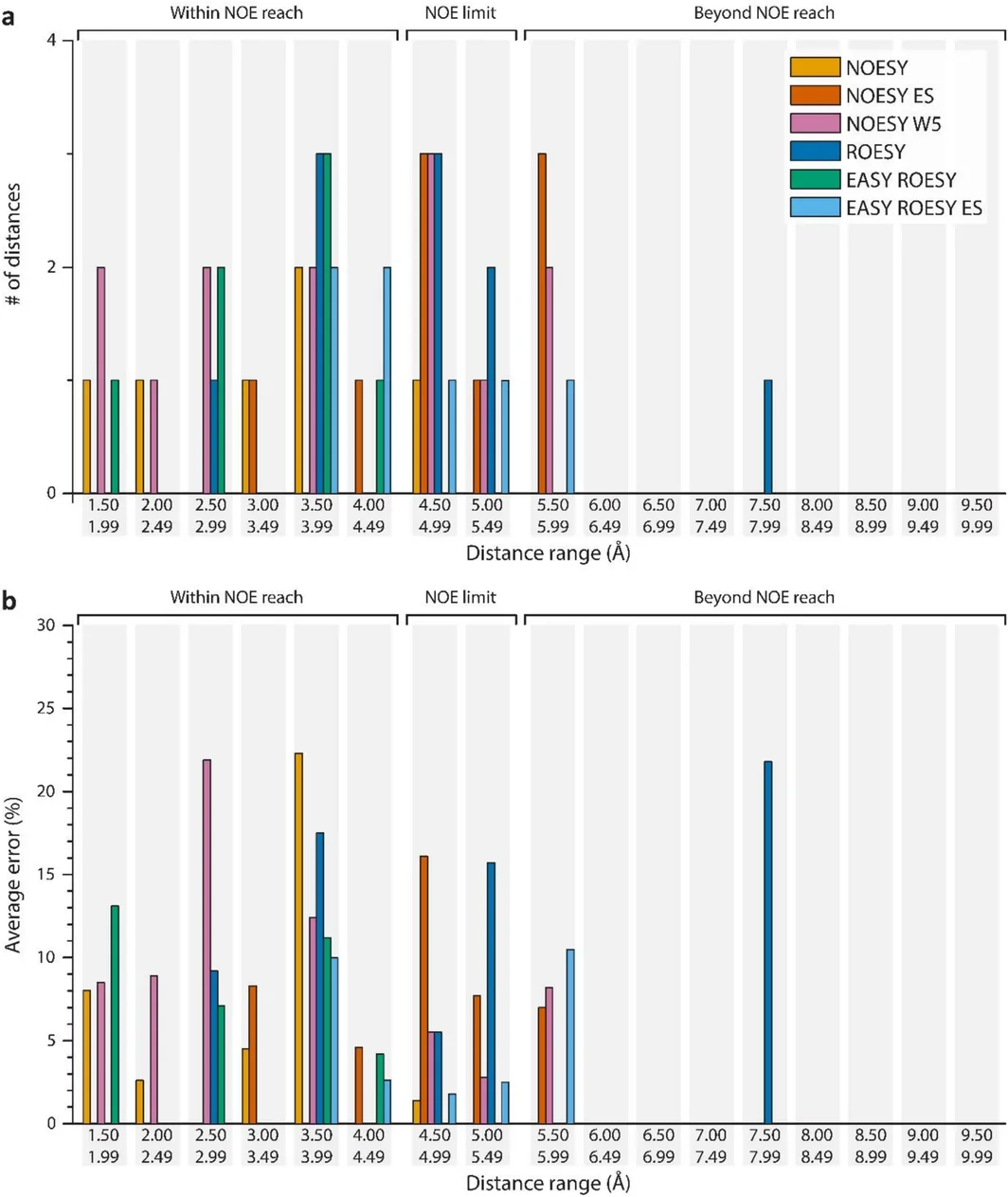
(a) Number of correlations that could be quantified using the initial‐rate approximation based on five data‐points with an *R* ^2^ of ≥ 0.95 for each pulse programme. It should be emphasised that a larger number of distances does not necessarily mean a better performance of a pulse sequence, as cross‐peaks may appear as the result of an artefact. (b) The average absolute error determined for the NOE‐derived distances of those correlations based on five data‐points with an *R* ^2^ of ≥ 0.95. The correlations are clustered into their corresponding distance range as determined based on the time‐averaged solution ensemble of strychnine [26].

In case an initial build‐up rate with an *R* ^2^ ≥ 0.95 could not be determined using the integrals obtained with seven or six mixing times, the outlier data‐points were excluded, as described above, and the initial build‐up rate was determined based on the data from the remaining five mixing times (Figure 5, Tables 6 and S11–S13). Figure 5 shows the number of correlations found and their corresponding error on the NOE‐derived distances, based on five data‐points used for the initial build‐up rate approximation. The number of correlations derived from build‐up curves based on five mixing times for distances up to 4.50 Å were 2 for the NOESY ES data‐set and between 4 and 7 for all the other pulse programmes. A more substantial portion of artificial distances, > 5.50 Å, were found, namely, 3 for the data‐set acquired with NOESY ES, 2 acquired with NOESY W5, and 1 for each of those detected using the EASY ROESY and EASY ROESY ES pulse programmes. The errors on the distances for the correlations within the NOE‐reach were large, frequently above 10%, when compared with the distances derived from build‐up rates using more data‐points (mixing times).

**TABLE 6:** Number of distances found for correlations based on 5 mixing times and their corresponding average absolute error (%) in between brackets for each pulse programme separated on distance range.

	Distance range (Å)	NOESY	NOESY ES	NOESY W5	ROESY	EASY ROESY	EASY ROESY ES
	All < 5.50 Å	6 (10.2%)	6 (10.0%)	11 (10.0%)	9 (13.1%)	7 (9.3%)	6 (5.7%)
Short reliable	0.00–2.99	2 (5.3%)	0	5 (14.0%)	1 (9.2%)	3 (9.1%)	0
Long reliable	3.00–4.49	3 (16.4%)	2 (6.4%)	2 (12.4%)	3 (17.5%)	4 (9.4%)	4 (6.3%)
At the NOE limit	4.50–5.49	1 (1.4%)	4 (14.0%)	4 (4.8%)	5 (9.6%)	0	2 (2.2%)
Beyond the NOE limit	5.55–9.99	0	3 (7.0%)	2 (8.2%)	1 (21.8%)	0	1 (10.5%)

#### 3.4.4 Accuracy of Distances Derived From Build‐Up Curves Based on Four Mixing Times

**FIGURE 6:**
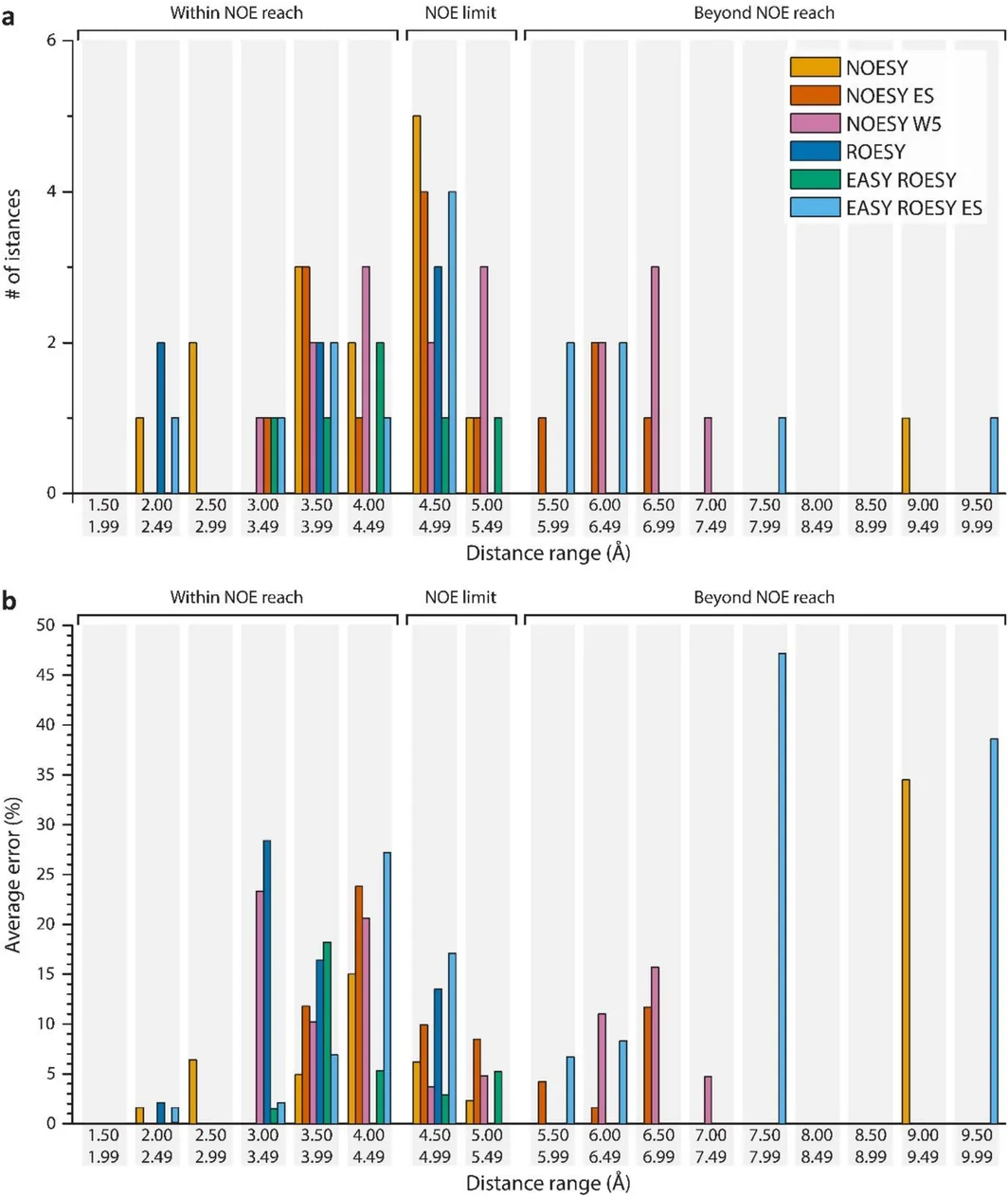
(a) Number of correlations that could be quantified using the initial‐rate approximation based on four data‐points with an *R* ^2^ of ≥ 0.95 for each pulse programme. It should be emphasised that a larger number of distances does not necessarily mean a better performance of a pulse sequence, as cross‐peaks may appear as the result of an artefact. (b) The average absolute error determined for the NOE‐derived distances of those correlations based on four data‐points with an *R* ^2^ of ≥ 0.95. The correlations are clustered into their corresponding distance range as determined based on the time‐averaged solution ensemble of strychnine [26].

When the initial build‐up rate with an *R* ^2^ ≥ 0.95 could not be determined using the integrals obtained with 5–7 of the detected mixing times, the outlier data‐points were excluded, as described above, and the initial build‐up rate was determined based on the data from the remaining 4 mixing times (Figure 6, Tables 7 and S14–S16). Very few additional short‐range correlations (up to 3.00 Å) were found (Figure 6). The six short‐range NOE‐derived distances that could be determined had a small error (2%–9%, Tables S16–S22). Between 2 and 5 correlations ranging from 3.00 to 4.50 Å could be quantified, but with a large average absolute error (between 7.5% and 20.4% average absolute error). A significant number, 4–6, artificial distances > 5.50 Å, were ‘quantified’ for the data obtained with solvent suppression (NOESY ES, NOESY W5 and EASY ROESY ES).

**TABLE 7:** Number of distances found for correlations based on 4 mixing times (and their corresponding average absolute error [%] in between brackets for each pulse programme separated on distance range).

	Distance range (Å)	NOESY	NOESY ES	NOESY W5	ROESY	EASY ROESY	EASY ROESY ES
	All < 5.50 Å	14 (8.5%)	9 (9.7%)	11 (11.8%)	8 (13.3%)	6 (6.4%)	9 (15.3%)
Short reliable	0.00–2.99	3 (4.8%)	0	0	2 (2.1%)	0	1 (1.6%)
Long reliable	3.00–4.49	5 (9.0%)	4 (14.8%)	6 (17.6%)	3 (20.4%)	4 (7.5%)	4 (10.8%)
At the NOE limit	4.50–5.49	6 (5.5%)	5 (9.6%)	5 (4.4%)	3 (13.5%)	2 (4.0%)	4 (17.1%)
Beyond the NOE limit	5.55–9.99	1 (34.5%)	4 (4.8%)	6 (12.3%)	0	0	6 (19.3%)

The reliability of the correlations as well as the accuracy of the corresponding distances decreased significantly whenever one or more data points (spectra) had to be removed from a build‐up with *R* ^2^ ≤ 0.95 to obtain the desired linearity (*R* ^2^ ≥ 0.95). This is indicative of a lower quality correlation. When removing a single spectrum from build‐ups that showed *R* ^2^ ≥ 0.95 with all 7 mixing times included, the error on the corresponding distances did not change significantly (Tables S32–S37). Hence, the key issue is the inclusion of low quality data points into structure determination, not the lower number of points used in the calculation of build‐up rates. The acquisition of build‐ups with a larger number of mixing times is thus helpful in identifying the high quality correlations that can be quantified reliably.

## 4 Discussion and Conclusions

Our data suggests that correlations derived from build‐up rates with 7 mixing times result in distances within the NOE‐reach, and associated average absolute errors mostly within 5% (Table 4 and Tables S5–S7). Excluding the correlations that do not meet the quality criterion of *R* ^2^ ≥ 0.95 provides, however, only 25%–45% of the expected interproton distances up to 4.50 Å (Table S5). The NOESY ES and EASY ROESY provide the fewest correlations covering only about 25%–30% of the expected ones, as compared with the pulse programmes that do not use excitation sculpting (ES) for solvent suppression (40%–45%).

The correlations not meeting the quality criterion were not associated to specific atoms, and no atoms were found problematic to specific experiments (NOESY or ROESY). Atoms with strong or multiple couplings did not show larger errors than those with less or weaker couplings. The data points resulting in lower quality build‐ups were true outliers, and could rarely be ascribed to spin diffusion.

Removing a single data point with highest experimental error from those build‐up curves that do not meet the *R* ^2^ ≥ 0.95 criterion with seven mixing times resulted in an additional 10%–20% of the expected interproton distances (Table 5 and Tables S8–S10). These distances, determined using six mixing times only, are within the expected NOE distance‐range (< 5.00 Å), with rare exceptions only. However, the error for many of these distances is significantly larger, > 10% (Tables S10 and S17–S22), than for the distances obtained using seven data points in the build‐up (Tables S7 and S17–S22).

When calculating interproton distances from build‐up curves with four or five mixing times only, the risk of introducing artificial distances > 5.50 Å becomes significant, especially when using the NOESY W5, NOESY ES or EASY ROESY ES pulse programmes. Thereto, the accuracy of distances derived from five or less data points in a build‐up curve have errors frequently above 10%, and distances with errors of 20% or more are commonly observed. Incorporating these distances into structure elucidation process may therefore be counterproductive and jeopardise the quality of the outcome.

In our hands, solvent suppression using NOESY ES, NOESY W5 and EASY ROESY ES resulted in the appearance of a number of artefacts, for instance 10%–16% of the obtained distances were beyond 5.50 Å (Table S2). Moreover, the use of excitation sculpting led to the underestimation of interproton distances, in general. This effect was quite severe with up to 70% of the distances being underestimated. However, the average absolute error for each specific distance range followed the same trends as seen when acquiring data without excitation sculpting. It should be noted that strychnine has no signals near 4.70 ppm, the frequency of the suppression pulse, and therefore the errors of distances derived from data close to this frequency for other compounds may be different, expectably larger, than obtained for strychnine. Solvent suppression using the W5 watergate pulse provided a larger number of quantifiable correlations and lower distance errors, however, this data‐set also suffered from artefacts in the form of quantifiable build‐up curves for interproton distances > 5.00 Å. Still, its use may be beneficial as compared with solvent suppression with excitation sculpting.

EASY ROESY has originally been developed to compensate for several weaknesses of ROESY for internuclear distance determination and has been in particular recommended for detecting correlations when NOE cross‐peaks are cumbersome to obtain due to the zero‐crossover region of NOESY. In our hands, EASY ROESY provided an unusually large percentage, 78.6%, of overestimated distances. Introduction of excitation sculpting, EASY ROESY ES, provided some compensation and led to a more even underestimated to overestimated distances distribution in the ratio 38:23 (59.4% underestimated and 35.9% overestimated). In our hands, the use of EASY ROESY instead of ROESY did not result in a significantly larger number of quantifiable distances yet a lower error of the interproton distances was found (Tables 1 and 3 and S2–S4). We also note that the use of EASY ROESY or ROESY did neither provide a larger number of quantifiable correlations nor lower error of the determined distances as compared with the data obtained with NOESY (Table 2 and Tables S2–S4). EASY ROESY was acquired with the roesyadesjsph pulse sequence that is 8 ms longer than roesyadjsph, during which time the magnetisation is transverse and may allow the development of coupling artefacts and *T*_*2*_ relaxation to occur that may influence signal intensities. When solvent suppression is not needed, roesyadjsph should be preferably used. As strychnine in DMSO‐*d*_6_ is in the extreme narrowing limit, it provided positive cross‐peaks. Analysis of the data of compounds close to the zero‐crossover region or of those in the slowly tumbling region yielding negative NOEs may provide different conclusions.

### 4.1 Authors' Recommendations

Based on the above discussion, we recommend the use of NOESY (noesyph) over the other investigated pulse programmes whenever possible for any form of quantitative NOE‐based distance determination. (EASY) ROESY reduces the accuracy of the calculated distances and introduces several artificial distances when solvent suppression was used. It is not recommended but may be an alternative when no NOEs are detectable, no alternative spectrometer frequency is available, and the sample conditions cannot be changed (temperature, solvent). Integrating a single NOESY or ROESY spectrum with one mixing time for NOE‐based distance determination should be avoided. This leads to serious contamination of the data by doubling the magnitude of error on the determined distances and introduction of large number of artificial distances. The extent of the introduced error depends on the mixing time. In this work, the error of the determined distances was larger for the spectra recorded with 100–200 ms than with 300–700 ms NOESY mixing times. Finally, when calculating the distances from the build‐up rates, it is important to temper with the build‐up curves as little as possible. The need for excluding data‐points to achieve linearity is often indicative of a lower quality build‐up, and the corresponding obtained distances should therefore be considered with care. Excluding one of the spectra (mixing time) from the build‐up curve results in an average absolute error that is about twice as large as compared with those derived from all the NOESY spectra. Excluding two or more spectra from the build‐up curves led to an even further increase in error for all pulse programmes, and to the introduction of artificial distances when pulse sequences employing solvent suppression were used. We therefore recommend to only use data derived from highly linear (*R* ^2^ ≥ 0.95) build‐up curves using all spectra (mixing times), and avoid quantifying the correlations that show higher errors (*R* ^2^ ≤ 0.95). If data that requires the removal of data points (normalised intensities form certain mixing times of the build‐up curve to reach an *R* ^2^ ≥ 0.95) is to be considered, the corresponding distances should be given a large error in the subsequent structure determination. In our hands, W5 watergate provides a better alternative than excitation sculpting for solvent suppression when quantitative NOEs are to be obtained.

The above conclusions are relevant for any research that implements NMR‐based structure determination using NOE‐derived interatomic distances, independent on the scientific context.

## Author Contributions

**Máté Erdélyi:** conceptualization, investigation, funding acquisition, writing – review and editing, methodology, project administration, supervision, resources, formal analysis. **Lianne H. E. Wieske:** conceptualization, investigation, writing – original draft, methodology, validation, visualization, writing – review and editing, formal analysis, data curation. **Gintarė Lygnugarytė:** formal analysis, investigation.

## Supporting information


**Figure S1:** Molecular structure of strychnine and atom‐numbering used for the NMR assignment.
**Table S1:** mrc_70127‐sup‐0001‐SI_rev_2.docx. ^1^H NMR assignment of strychnine in DMSO‐*d*_*6*_.
**Figure S2:** Data‐selection flow‐chart for the build‐up generation of the interproton distances.
**Table S2:** Number of interproton correlations found for each pulse program sorted by distance range. The distance range each correlation is grouped at is based on the population‐averaged interproton distances determined by Butts *et. al.*.^[3]^ The percentage (%) of correlations found of the total number of theoretically available correlation is given behind the absolute number of correlations found.
**Table S3:** Average absolute error (|Experimental distance—Theoretical distance| in Å) for correlations found for each pulse program sorted by distance range. The distance range each correlation is grouped at is based on the population‐averaged interproton distances determined by Butts *et. al.* ^[3]^
**Table S4:** Average absolute error (%) for correlations found for each pulse program sorted by distance range.
**Table S5:** Number of interproton correlations based on 7 data‐points (mixing times) only, found for each pulse program sorted by distance range. The percentage (%) of correlations found of the total number of theoretically available correlations is given behind the absolute number of correlations found.
**Table S6:** Average absolute error (|Experimental distance—Theoretical distance| in Å) for correlations based on 7 data‐points (mixing times) only found for each pulse program sorted by distance range.
**Table S7:** Average absolute error (%) for correlations based on 7 data‐points (mixing times) only found for each pulse program sorted by distance range.
**Table S8:** Number of interproton correlations based on 6 data‐points (mixing times) only found for each pulse program sorted by distance range.
**Table S9:** Average absolute error (|Experimental distance—Theoretical distance| in Å) for correlations based on 6 data‐points (mixing times) only found for each pulse program sorted by distance range.
**Table S10:** Average absolute error (%) for correlations based on 6 data‐points (mixing times) only found for each pulse program sorted by distance range.
**Table S11:** Number of interproton correlations based on 5 data‐points (mixing times) only found for each pulse program sorted by distance range.
**Table S12:** Average absolute error (|Experimental distance—Theoretical distance| in Å) for correlations based on 5 data‐points (mixing times) only found for each pulse program sorted by distance range.
**Table S13:** Average absolute error (%) for correlations based on 5 data‐points (mixing times) only found for each pulse program sorted by distance range.
**Table S14:** Number of interproton correlations based on 4 data‐points (mixing times) only found for each pulse program sorted by distance range.
**Table S15:** Average absolute error (|Experimental distance—Theoretical distance| in Å) for correlations based on 4 data‐points (mixing times) only found for each pulse program sorted by distance range.
**Table S16:** Average absolute error (%) for correlations based on 4 data‐points (mixing times) only found for each pulse program sorted by distance range.
**Table S17:** Quantified distances obtained using the noesyph pulse program from the matrix generated from the diagonal integrals directly (NOESY), or after which the cross‐peaks were adjusted to prevent the inclusion of noise (NOESY adjusted cross‐peaks). The theoretical population‐averaged interproton distances as determined by Butts *et al.* ^[3]^ are given for each correlation, and used to calculate the error (Exp. – Theor.) as well as the absolute error (|%|). The number of data‐points included (# t_mix_) for the distance determination, as well as the coefficient of determination (R^2^) of the linear regression are given. At the bottom of the table, the total number of quantified correlations as well as the number of over‐ and underestimated correlations are given. The proton‐proton corelation used as a reference for distance calculation is being underscored.
**Table S18:** Quantified distances for the noesyesgpph pulse program from the matrix generated from the diagonal integrals directly (NOESY ES) or after which the cross‐peaks were adjusted to prevent the inclusion of noise (NOESY ES adjusted cross‐peaks). The theoretical population‐averaged interproton distances as determined by Butts *et al.* ^[3]^ are given for each correlation, and used to calculate the error (Exp. – Theor.) as well as the absolute error (|%|). The number of data‐points included (# t_mix_) for the distance determination, as well as the coefficient of determination (R^2^) of the linear regression are given. At the bottom of the table, the total number of quantified correlations as well as the number of over‐ and underestimated correlations are given. The proton‐proton correlation used as a reference for distance calculation is being underscored.
**Table S19:** Quantified distances for the noesydbphw5 pulse program from the matrix generated from the diagonal integrals directly (NOESY W5) or after which the cross‐peaks were adjusted to prevent the inclusion of noise (NOESY W5 adjusted cross‐peaks). The theoretical population‐averaged interproton distances as determined by Butts *et al.* ^[3]^ are given for each correlation, and used to calculate the error (Exp. – Theor.) as well as the absolute error (|%|). The number of data‐points included (# t_mix_) for the distance determination, as well as the coefficient of determination (R^2^) of the linear regression are given. At the bottom of the table, the total number of quantified correlations as well as the number of over‐ and underestimated correlations are given. The proton‐proton correlation used as a reference for distance calculation is being underscored.
**Table S20:** Quantified distances for the roesyph pulse program from the matrix generated from the diagonal integrals directly (ROESY) or after which the cross‐peaks were adjusted to prevent the inclusion of noise (ROESY adjusted cross‐peaks). The theoretical population‐averaged interproton distances as determined by Butts *et al.* ^[3]^ are given for each correlation, and used to calculate the error (Exp. – Theor.) as well as the absolute error (|%|). The number of data‐points included (# t_mix_) for the distance determination, as well as the coefficient of determination (R^2^) of the linear regression are given. At the bottom of the table, the total number of quantified correlations as well as the number of over‐ and underestimated correlations are given. The proton‐proton correlation used as a reference for distance calculation is being underscored.
**Table S21:** Quantified distances for the EASY ROESY without solvent suppression roesyadesjsph pulse program from the matrix generated from the diagonal integrals directly (EASY ROESY) or after which the cross‐peaks were adjusted to prevent the inclusion of noise (EASY ROESY adjusted cross‐peaks). The theoretical population‐averaged interproton distances as determined by Butts *et al.* ^[3]^ are given for each correlation, and used to calculate the error (Exp. – Theor.) as well as the absolute error (|%|). The number of data‐points included (# t_mix_) for the distance determination, as well as the coefficient of determination (R^2^) of the linear regression are given. At the bottom of the table, the total number of quantified correlations as well as the number of over‐ and underestimated correlations are given. The proton‐proton correlation used as a reference for distance calculation is being underscored.
**Table S22:** Quantified distances for the EASY ROESY with solvent suppression roesyadesjsph (*Instead of roesyadesjsph, the use of roesyadjsph is recommended when no solvent suppression is necessary. The roesyadesjsph includes two additional 180° pulses, adding 8 ms when the magnetization is transverse, possibly allowing for T2 relaxation and the development of coupling artefacts.*) pulse program from the matrix generated from the diagonal integrals directly (EASY ROESY ES) or after which the cross‐peaks were adjusted to prevent the inclusion of noise (EASY ROESY ES adjusted cross‐peaks). The theoretical population‐averaged interproton distances as determined by Butts *et al.* ^[3]^ are given for each correlation, and used to calculate the error (Exp. – Theor.) as well as the absolute error (|%|). The number of data‐points included (# t_mix_) for the distance determination, as well as the coefficient of determination (R^2^) of the linear regression are given. At the bottom of the table, the total number of quantified correlations as well as the number of over‐ and underestimated correlations are given. The proton‐proton correlation used as a reference for distance calculation is being underscored.
**Table S23:** Number of correlations that could be quantified with the NOESY pulse program noesyph for each of the 7 mixing times.
**Table S24:** Average absolute error (%) on the calculated distances for which the correlations were quantified based on the normalised intensity (single spectrum) with the NOESY pulse program noesyph for each of the 7 mixing times.
**Table S25:** Number of interproton distances that could be quantified with the NOESY pulse program noesyph from correlations for which the underlaying cross‐peaks have the same phase for each of the 7 mixing times.
**Table S26:** Average absolute error (%) on the calculated distances for which the interproton distances were quantified based on the normalised intensity (single spectrum) for which the underlaying cross‐peaks have the same phase with the NOESY pulse program noesyph for each of the 7 mixing times.
**Table S27:** Number of correlations that could be quantified with the NOESY pulse program noesyph from correlations for which the underlaying cross‐peaks have the same phase for each of the 7 mixing times. Correlations for which distances were calculated to be 5.50 Å or larger were excluded.
**Table S28:** Average absolute error (%) on the calculated distances for which the correlations were quantified based on the normalised intensity (single spectrum) for which the underlaying cross‐peaks have the same phase with the NOESY pulse program noesyph for each of the 7 mixing times. Correlations for which distances were calculated to be 5.50 Å or larger were excluded.
**Table S29:** Quantified distances based on the normalised intensity of each of the spectra recorded at different mixing times (100–700 ms) for the NOESY sequence (noesyph). The theoretical population‐averaged interproton distances as determined by Butts *et al.* ^[3]^ are given for each correlation, and used to calculate the absolute error (|%|). The proton‐proton correlation used as a reference for distance calculation was proton‐pair 15 and is highlighted by being underscored.
**Table S30:** Quantified distances based on the normalised intensity of each of the spectra recorded at different mixing times (100–700 ms) for the NOESY sequence (noesyph). Correlations for which the involved cross‐peaks had opposing signs are excluded (indicated by ‘Phase’ as an entry in the table). The theoretical population‐averaged interproton distances as determined by Butts *et. al.* ^[3]^ are given for each correlation, and used to calculate the absolute error (|%|). The proton‐proton correlation used as a reference for distance calculation was proton‐pair 15 and is highlighted by being underscored.
**Table S31:** Quantified distances based on the normalised intensity of each of the spectra recorded at different mixing times (100–700 ms) for the NOESY sequence (noesyph). Correlations for which the involved cross‐peaks had opposing signs (indicated by ‘Phase’ as an entry in the table), as well as those that were calculated to be 5.50 Å or more are excluded (blank entries). The theoretical population‐averaged interproton distances as determined by Butts *et al.* ^[3]^ are given for each correlation, and used to calculate the absolute error (|%|). The proton‐proton correlation used as a reference for distance calculation was proton‐pair 15 and is highlighted by being underscored.
**Table S32:** Average absolute error of the quantified distances based on all 7 spectra recorded at different mixing times (100–700 ms) for the NOESY sequence (noesyph). Same distances quantified from the same spectra excluding one of the mixing times. The difference between the original average absolute error and average absolute errors obtained when including only 6 different mixing times are given in‐between brackets.
**Table S33:** Average absolute error of the quantified distances based on all 7 spectra recorded at different mixing times (100–700 ms) for the NOESY ES (noesyesgpph). Same distances quantified from the same spectra excluding one of the mixing times. The difference between the original average absolute error and average absolute errors obtained when including only 6 different mixing times are given in‐between brackets.
**Table S34:** Average absolute error of the quantified distances based on all 7 spectra recorded at different mixing times (100–700 ms) for the NOESY W5 (noesydbphw5). Same distances quantified from the same spectra excluding one of the mixing times. The difference between the original average absolute error and average absolute errors obtained when including only 6 different mixing times are given in‐between brackets.
**Table S35:** Average absolute error of the quantified distances based on all 7 spectra recorded at different mixing times (100–700 ms) for the ROESY (roesyph). Same distances quantified from the same spectra excluding one of the mixing times. The difference between the original average absolute error and average absolute errors obtained when including only 6 different mixing times are given in‐between brackets.
**Table S36:** Average absolute error of the quantified distances based on all 7 spectra recorded at different mixing times (100–700 ms) for the EASY ROESY (roesyadesjsph). Same distances quantified from the same spectra excluding one of the mixing times. The difference between the original average absolute error and average absolute errors obtained when including only 6 different mixing times are given in‐between brackets.
**Table S37:** Average absolute error of the quantified distances based on all 7 spectra recorded at different mixing times (100–700 ms) for the EASY ROESY SS (roesyadesjsph). Same distances quantified from the same spectra excluding one of the mixing times. The difference between the original average absolute error and average absolute errors obtained when including only 6 different mixing times are given in‐between brackets.

## Data Availability

Original NMR FIDs are freely available online at Zenodo with doi: 10.5281/zenodo.18116559. The analysed data are available in the Supporting Information.
