# Supplementary material for: Pulse Programme Considerations for Quantitative NOE Analysis
Source: Magn Reson Chem. 2026 Jun 22;64(10):873–89. doi: 10.1002/mrc.70127 (PMC13532781; doi:10.1002/mrc.70127)
Supplement: Supplementary file 1 [file MRC-64-873-s001.docx]

**Supplementary Material**

**Pulse program considerations for quantitative NOE analysis**

Lianne H. E. Wieske, Gintarė Lygnugarytė, and Máté Erdélyi

**Table of contents**

|  | Page |
| --- | --- |
| 1. NMR assignment and data-selection | S2 |
| 2. Correlations grouped by distance range | S3 |
| 3. Ungrouped data | S18 |
| 4. Distances determination from NOEs obtained from a single mixing time | S48 |
| 5. References | S84 |

Original NMR FIDs are freely available online at Zenodo with doi:10.5281/zenodo.18116559

**1. NMR assignment and data-selection**

The ^1^H NMR assignment of strychnine was established in the 1970s^[1]^ in CDCl_3_ and has since been confirmed in other solvents.^[2]^ The ^1^H NMR assignment used in this study is shown in Table S1.

**Figure S1.** Molecular structure of strychnine and atom-numbering used for the NMR assignment.

**Table S1.** ^1^H NMR assignment of strychnine in DMSO-*d_6_*

| **Atom nr.** | **δ ^1^H (ppm)** | **Atom nr.** | **δ ^1^H (ppm)** |
| --- | --- | --- | --- |
| **1** | 7.31 | **15a** | 2.26 |
| **2** | 7.07 | **15b** | 1.29 |
| **3** | 7.21 | **16** | 3.82 |
| **4** | 7.91 | **17a** | 1.82 |
| **8** | 3.72 | **17b** | 1.70 |
| **11a** | 2.92 | **18a** | 3.01 |
| **11b/20b** | 2.59 | **18b** | 2.72 |
| **12** | 4.28 | **20a** | 3.57 |
| **13** | 1.23 | **22** | 5.83 |
| **14** | 3.11 | **23ab** | 4.04 |

*Workflow: Selection of correlations for quantification*

Once the integral values were extracted from the NOESY and ROESY spectra, they were submitted to a series of selection criteria, prior to distance determination. These selection criteria were based on # of spectra that were included for the linear regression and R^2^ values (Figure S2).

**Figure S2.** Data-selection flow-chart for the build-up generation of the interproton distances.

**2. Correlations grouped by distance range**

**Table S2**. Number of interproton correlations found for each pulse program sorted by distance range. The distance range each correlation is grouped at is based on the population-averaged interproton distances determined by Butts *et. al.*.^[3]^ The percentage (%) of correlations found of the total number of theoretically available correlation is given behind the absolute number of correlations found.

|  | **Distance range (Å)** | **Theoretically available** | **NOESY** | **NOESY ES** | **NOESY W5** | **ROESY** | **EASY ROESY** | **EASY ROESY ES** |
| --- | --- | --- | --- | --- | --- | --- | --- | --- |
| **Short reliable** | 0.00-1.99 | 5 | 5  100% | 2  40.0% | 5  100% | 5  100% | 3  60.0% | 3  60.0% |
|  | 2.00-2.49 | 19 | 16  84.2% | 15  78.9% | 16  84.2% | 18  94.7% | 14  73.7% | 14  73.7% |
|  | 2.50-2.99 | 10 | 9  90.0% | 4  40.0% | 9  90.0% | 10  100% | 8  80.0% | 8  80.0% |
| **Long reliable** | 3.00-3.49 | 7 | 6  85.7% | 3  42.9% | 5  71.4% | 7  100% | 4  57.1% | 6  85.7% |
|  | 3.50-3.99 | 22 | 13  59.1% | 8  36.4% | 12  54.5% | 11  50% | 8  36.4% | 11  50% |
|  | 4.00-4.49 | 15 | 3  20.0% | 3  20.0% | 4  26.7% | 1  6.7% | 3  20.0% | 5  33.3% |
| **At the NOE limit** | 4.50-4.99 | 29 | 6  20.7% | 9  31.0% | 8  27.6% | 6  20.7% | 1  3.4% | 9  31.0% |
|  | 5.00-5.49 | 13 | 1  7.7% | 3  23.1% | 5  38.5% | 2  15.4% | 1  7.7% | 1  7.7% |
| **Beyond the NOE limit** | 5.50-5.99 |  | 0 | 5 | 2 | 0 | 0 | 3 |
|  | 6.00-6.49 |  | 0 | 3 | 2 | 0 | 0 | 2 |
|  | 6.50-6.99 |  | 0 | 1 | 3 | 0 | 0 | 0 |
|  | 7.00-7.49 |  | 0 | 0 | 1 | 0 | 0 | 0 |
|  | 7.50-7.99 |  | 0 | 0 | 0 | 1 | 0 | 1 |
|  | 8.00-8.49 |  | 0 | 0 | 0 | 0 | 0 | 0 |
|  | 8.50-8.99 |  | 0 | 0 | 0 | 0 | 0 | 0 |
|  | 9.00-9.49 |  | 1 | 0 | 0 | 0 | 0 | 0 |
|  | 9.50-9.99 |  | 0 | 0 | 0 | 0 | 0 | 1 |
| **Total** |  |  |  |  |  |  |  |  |
| **Short reliable** | 0.00-2.99 | 34 | 30  88.2% | 21  61.8% | 30  88.2% | 33  97.1% | 25  73.5% | 25  73.5% |
| **Long reliable** | 3.00-4.49 | 44 | 22  50.0% | 14  31.8% | 21  47.7% | 19  43.2% | 15  34.1% | 22  50.0% |
| **At the NOE limit** | 4.50-5.49 | 42 | 7  16.7% | 12  28.6% | 13  31.0% | 8  19.0% | 2  4.8% | 10  23.8% |
| **Beyond the NOE limit** | 5.50-9.99 |  | 1 | 9 | 8 | 1 | 0 | 7 |
| **All** | 0.00-9.99 |  | 60 | 56 | 72 | 61 | 42 | 64 |

For the NOES and NOESY W5 pulse-programs, spectra with a mixing time of 100, 200, 300, 400, 500, 600 and 700 ms were used, whereas for NOESY ES mixing times of 100, 200, 300, 400, 500, 700 and 800 ms were recorded. For the ROESY, EASY ROESY and EASY ROESY ES, spin-lock pulses of 50, 100, 150, 200, 250, 300 and 350 ms were used.

**Table S3**. Average absolute error (|Experimental distance – Theoretical distance| in Å) for correlations found for each pulse program sorted by distance range. The distance range each correlation is grouped at is based on the population-averaged interproton distances determined by Butts *et. al.*.^[3]^

|  | **Distance range (Å)** | **NOESY** | **NOESY ES** | **NOESY W5** | **ROESY** | **EASY ROESY** | **EASY ROESY ES** |
| --- | --- | --- | --- | --- | --- | --- | --- |
| **Short reliable** | 0.00-1.99 | 0.05 | 0.04 | 0.09 | 0.05 | 0.12 | 0.03 |
|  | 2.00-2.49 | 0.07 | 0.16 | 0.06 | 0.08 | 0.10 | 0.10 |
|  | 2.50-2.99 | 0.12 | 0.23 | 0.24 | 0.23 | 0.14 | 0.05 |
| **Long reliable** | 3.00-3.49 | 0.16 | 0.28 | 0.34 | 0.24 | 0.21 | 0.12 |
|  | 3.50-3.99 | 0.34 | 0.62 | 0.34 | 0.39 | 0.35 | 0.24 |
|  | 4.00-4.49 | 0.47 | 0.50 | 0.69 | 1.57 | 0.21 | 0.32 |
| **At the NOE limit** | 4.50-4.99 | 0.26 | 0.49 | 0.18 | 0.44 | 0.14 | 0.45 |
|  | 5.00-5.49 | 0.12 | 0.44 | 0.22 | 0.81 | 0.28 | 0.13 |
| **Beyond the NOE limit** | 5.50-5.99 | - | 0.45 | 0.48 | - | - | 0.45 |
|  | 6.00-6.49 | - | 0.47 | 0.68 | - | - | 0.51 |
|  | 6.50-6.99 | - | 0.77 | 1.08 | - | - | - |
|  | 7.00-7.49 | - | - | 0.35 | - | - | - |
|  | 7.50-7.99 | - | - | - | 1.68 | - | 3.62 |
|  | 8.00-8.49 | - | - | - | - | - | - |
|  | 8.50-8.99 | - | - | - | - | - | - |
|  | 9.00-9.49 | 3.17 | - | - | - | - | - |
|  | 9.50-9.99 | - | - | - | - | - | 3.79 |
| **Average** |  |  |  |  |  |  |  |
| **Short reliable** | 0.00-2.99 | 0.09 | 0.16 | 0.12 | 0.12 | 0.12 | 0.08 |
| **Long reliable** | 3.00-4.49 | 0.31 | 0.52 | 0.40 | 0.39 | 0.29 | 0.23 |
| **At the NOE limit** | 4.50-5.49 | 0.24 | 0.48 | 0.20 | 0.53 | 0.21 | 0.42 |
| **Beyond the NOE limit** | 5.50-9.99 | 3.17 | 0.49 | 0.74 | 1.68 | - | 1.40 |
| **All** | 0.00-9.99 | 0.24 | 0.37 | 0.29 | 0.29 | 0.18 | 0.33 |

**Table S4**. Average absolute error (%) for correlations found for each pulse program sorted by distance range.

|  | **Distance range (Å)** | **NOESY** | **NOESY ES** | **NOESY W5** | **ROESY** | **EASY ROESY** | **EASY ROESY ES** |
| --- | --- | --- | --- | --- | --- | --- | --- |
| **Short reliable** | 0.00-1.99 | 3.1 | 2.0 | 5.2 | 2.6 | 6.8 | 1.9 |
|  | 2.00-2.49 | 3.1 | 6.6 | 2.7 | 3.3 | 4.2 | 4.2 |
|  | 2.50-2.99 | 4.5 | 8.4 | 8.4 | 8.5 | 5.4 | 1.9 |
| **Long reliable** | 3.00-3.49 | 4.7 | 8.9 | 10.8 | 7.1 | 6.4 | 3.7 |
|  | 3.50-3.99 | 9.0 | 16.4 | 9.1 | 10.4 | 9.4 | 6.5 |
|  | 4.00-4.49 | 11.1 | 11.9 | 16.1 | 37.9 | 4.9 | 7.6 |
| **At the NOE limit** | 4.50-4.99 | 5.4 | 10.5 | 3.7 | 9.5 | 2.9 | 9.3 |
|  | 5.00-5.49 | 2.3 | 8.5 | 4.2 | 15.7 | 5.2 | 2.5 |
| **Beyond the NOE limit** | 5.50-5.99 | - | 7.8 | 8.2 | - | - | 7.9 |
|  | 6.00-6.49 | - | 7.6 | 11.0 | - | - | 8.3 |
|  | 6.50-6.99 | - | 11.7 | 15.7 | - | - | - |
|  | 7.00-7.49 | - | - | 4.7 | - | - | - |
|  | 7.50-7.99 | - | - | - | 21.8 | - | 47.2 |
|  | 8.00-8.49 | - | - | - | - | - | - |
|  | 8.50-8.99 | - | - | - | - | - | - |
|  | 9.00-9.49 | 34.5 | - | - | - | - | - |
|  | 9.50-9.99 | - | - | - | - | - | 38.6 |
| **Average** |  |  |  |  |  |  |  |
| **Short reliable** | 0.00-2.99 | 3.5 | 6.5 | 4.8 | 4.8 | 4.9 | 3.2 |
| **Long reliable** | 3.00-4.49 | 8.1 | 13.8 | 10.8 | 10.7 | 7.7 | 6.0 |
| **At the NOE limit** | 4.50-5.49 | 4.9 | 10.0 | 3.9 | 11.1 | 4.0 | 8.6 |
| **Beyond the NOE limit** | 5.50-9.99 | 34.5 | 8.2 | 11.3 | 21.8 | - | 18.0 |
| **All** | 0.00-9.99 | 5.9 | 9.4 | 7.1 | 7.7 | 5.8 | 6.6 |

**Table S5**. Number of interproton correlations based on 7 data-points (mixing times) only, found for each pulse program sorted by distance range. The percentage (%) of correlations found of the total number of theoretically available correlations is given behind the absolute number of correlations found.

|  | **Distance range (Å)** | **Theoretically available** | **NOESY** | **NOESY ES** | **NOESY W5** | **ROESY** | **EASY ROESY** | **EASY ROESY ES** |
| --- | --- | --- | --- | --- | --- | --- | --- | --- |
| **Short reliable** | 0.00-1.99 | 5 | 3  60.0% | 2  40.0% | 3  60.0% | 5  100% | 2  40.0 % | 2  40.0% |
|  | 2.00-2.49 | 19 | 12  63.2% | 10  52.6% | 15  78.9% | 16  84.2% | 13  68.4% | 10  52.6% |
|  | 2.50-2.99 | 10 | 6  60.0% | 3  30.0% | 6  60.0% | 7  70.0% | 5  50.0% | 7  70.0% |
| **Long reliable** | 3.00-3.49 | 7 | 4  57.1% | 2  28.6% | 3  42.9% | 3  42.9% | 2  28.6% | 3  42.9% |
|  | 3.50-3.99 | 22 | 6  27.3% | 3  13.6% | 5  22.7% | 3  13.6% | 1  9.1% | 5  22.7% |
|  | 4.00-4.49 | 15 | 1  6.7% | 1  6.7% | 1  6.7% | 1  6.7% | 0 | 2  13.3% |
| **At the NOE limit** | 4.50-4.99 | 29 | 0 | 0 | 2  6.9% | 0 | 0 | 0 |
|  | 5.00-5.49 | 13 | 0 | 0 | 0 | 0 | 0 | 0 |
| **Beyond the NOE limit** | 5.50-5.99 |  | 0 | 0 | 0 | 0 | 0 | 0 |
|  | 6.00-6.49 |  | 0 | 0 | 0 | 0 | 0 | 0 |
|  | 6.50-6.99 |  | 0 | 0 | 0 | 0 | 0 | 0 |
|  | 7.00-7.49 |  | 0 | 0 | 0 | 0 | 0 | 0 |
|  | 7.50-7.99 |  | 0 | 0 | 0 | 0 | 0 | 0 |
|  | 8.00-8.49 |  | 0 | 0 | 0 | 0 | 0 | 0 |
|  | 8.50-8.99 |  | 0 | 0 | 0 | 0 | 0 | 0 |
|  | 9.00-9.49 |  | 0 | 0 | 0 | 0 | 0 | 0 |
|  | 9.50-9.99 |  | 0 | 0 | 0 | 0 | 0 | 0 |
| **Total** |  |  |  |  |  |  |  |  |
| **Short reliable** | 0.00-2.99 | 34 | 21  61.8% | 15  44.1% | 24  70.6% | 28  82.4% | 20  58.8% | 19  55.9% |
| **Long reliable** | 3.00-4.49 | 44 | 11  25.0% | 6  13.6% | 9  20.5% | 7  15.9% | 3  6.8% | 10  22.7% |
| **At the NOE limit** | 4.50-5.49 | 42 | 0 | 0 | 2  4.8% | 0 | 0 | 0 |
| **Beyond the NOE limit** | 5.50-9.99 |  | 0 | 0 | 0 | 0 | 0 | 0 |
| **All** | 0.00-9.99 |  | 32 | 21 | 35 | 35 | 23 | 29 |

**Table S6**. Average absolute error (|Experimental distance – Theoretical distance| in Å) for correlations based on 7 data-points (mixing times) only found for each pulse program sorted by distance range.

|  | **Distance range (Å)** | **NOESY** | **NOESY ES** | **NOESY W5** | **ROESY** | **EASY ROESY** | **EASY ROESY ES** |
| --- | --- | --- | --- | --- | --- | --- | --- |
| **Short reliable** | 0.00-1.99 | 0.04 | 0.04 | 0.05 | 0.05 | 0.06 | 0.05 |
|  | 2.00-2.49 | 0.08 | 0.15 | 0.05 | 0.08 | 0.10 | 0.12 |
|  | 2.50-2.99 | 0.08 | 0.15 | 0.05 | 0.10 | 0.08 | 0.05 |
| **Long reliable** | 3.00-3.49 | 0.19 | 0.30 | 0.23 | 0.12 | 0.10 | 0.17 |
|  | 3.50-3.99 | 0.14 | 0.57 | 0.15 | 0.12 | 0.02 | 0.12 |
|  | 4.00-4.49 | 0.14 | 0.32 | 0.11 | 1.57 | - | 0.12 |
| **At the NOE limit** | 4.50-4.99 | - | - | 0.09 | - | - | - |
|  | 5.00-5.49 | - | - | - | - | - | - |
| **Beyond the NOE limit** | 5.50-5.99 | - | - | - | - | - | - |
|  | 6.00-6.49 | - | - | - | - | - | - |
|  | 6.50-6.99 | - | - | - | - | - | - |
|  | 7.00-7.49 | - | - | - | - | - | - |
|  | 7.50-7.99 | - | - | - | - | - | - |
|  | 8.00-8.49 | - | - | - | - | - | - |
|  | 8.50-8.99 | - | - | - | - | - | - |
|  | 9.00-9.49 | - | - | - | - | - | - |
|  | 9.50-9.99 | - | - | - | - | - | - |
| **Average** |  |  |  |  |  |  |  |
| **Short reliable** | 0.00-2.99 | 0.07 | 0.14 | 0.05 | 0.08 | 0.09 | 0.09 |
| **Long reliable** | 3.00-4.49 | 0.16 | 0.44 | 0.17 | 0.33 | 0.07 | 0.14 |
| **At the NOE limit** | 4.50-5.49 | - | - | 0.09 | - | - | - |
| **Beyond the NOE limit** | 5.50-9.99 | - | - | - | - | - | - |
| **All** | 0.00-9.99 | 0.10 | 0.22 | 0.09 | 0.13 | 0.09 | 0.11 |

**Table S7**. Average absolute error (%) for correlations based on 7 data-points (mixing times) only found for each pulse program sorted by distance range.

|  | **Distance range (Å)** | **NOESY** | **NOESY ES** | **NOESY W5** | **ROESY** | **EASY ROESY** | **EASY ROESY ES** |
| --- | --- | --- | --- | --- | --- | --- | --- |
| **Short reliable** | 0.00-1.99 | 2.5 | 2.0 | 3.0 | 2.6 | 3.7 | 2.8 |
|  | 2.00-2.49 | 3.4 | 6.5 | 2.3 | 3.4 | 4.0 | 5.2 |
|  | 2.50-2.99 | 2.8 | 5.6 | 1.8 | 3.7 | 3.1 | 2.0 |
| **Long reliable** | 3.00-3.49 | 5.8 | 9.2 | 7.1 | 3.8 | 2.9 | 5.2 |
|  | 3.50-3.99 | 3.7 | 14.8 | 4.1 | 3.2 | 0.5 | 3.2 |
|  | 4.00-4.49 | 3.2 | 7.2 | 2.5 | 37.9 | - | 2.8 |
| **At the NOE limit** | 4.50-4.99 | - | - | 1.8 | - | - | - |
|  | 5.00-5.49 | - | - | - | - | - | - |
| **Beyond the NOE limit** | 5.50-5.99 | - | - | - | - | - | - |
|  | 6.00-6.49 | - | - | - | - | - | - |
|  | 6.50-6.99 | - | - | - | - | - | - |
|  | 7.00-7.49 | - | - | - | - | - | - |
|  | 7.50-7.99 | - | - | - | - | - | - |
|  | 8.00-8.49 | - | - | - | - | - | - |
|  | 8.50-8.99 | - | - | - | - | - | - |
|  | 9.00-9.49 | - | - | - | - | - | - |
|  | 9.50-9.99 | - | - | - | - | - | - |
| **Average** |  |  |  |  |  |  |  |
| **Short reliable** | 0.00-2.99 | 3.1 | 5.7 | 2.2 | 3.3 | 3.8 | 3.8 |
| **Long reliable** | 3.00-4.49 | 4.4 | 11.7 | 4.9 | 8.4 | 2.1 | 3.7 |
| **At the NOE limit** | 4.50-5.49 | - | - | 1.8 | - | - | - |
| **Beyond the NOE limit** | 5.50-9.99 | - | - | - | - | - | - |
| **All** | 0.00-9.99 | 3.5 | 7.4 | 2.9 | 4.4 | 3.6 | 3.8 |

**Table S8**. Number of interproton correlations based on 6 data-points (mixing times) only found for each pulse program sorted by distance range.

|  | **Distance range (Å)** | **NOESY** | **NOESY ES** | **NOESY W5** | **ROESY** | **EASY ROESY** | **EASY ROESY ES** |
| --- | --- | --- | --- | --- | --- | --- | --- |
| **Short reliable** | 0.00-1.99 | 1 | 0 | 0 | 0 | 0 | 1 |
|  | 2.00-2.49 | 2 | 5 | 0 | 0 | 1 | 3 |
|  | 2.50-2.99 | 1 | 1 | 1 | 2 | 1 | 1 |
| **Long reliable** | 3.00-3.49 | 1 | 0 | 1 | 3 | 1 | 2 |
|  | 3.50-3.99 | 2 | 2 | 3 | 3 | 3 | 2 |
|  | 4.00-4.49 | 0 | 0 | 0 | 0 | 0 | 0 |
| **At the NOE limit** | 4.50-4.99 | 0 | 2 | 1 | 0 | 0 | 4 |
|  | 5.00-5.49 | 0 | 1 | 1 | 0 | 0 | 0 |
| **Beyond the NOE limit** | 5.50-5.99 | 0 | 1 | 0 | 0 | 0 | 0 |
|  | 6.00-6.49 | 0 | 1 | 0 | 0 | 0 | 0 |
|  | 6.50-6.99 | 0 | 0 | 0 | 0 | 0 | 0 |
|  | 7.00-7.49 | 0 | 0 | 0 | 0 | 0 | 0 |
|  | 7.50-7.99 | 0 | 0 | 0 | 0 | 0 | 0 |
|  | 8.00-8.49 | 0 | 0 | 0 | 0 | 0 | 0 |
|  | 8.50-8.99 | 0 | 0 | 0 | 0 | 0 | 0 |
|  | 9.00-9.49 | 0 | 0 | 0 | 0 | 0 | 0 |
|  | 9.50-9.99 | 0 | 0 | 0 | 0 | 0 | 0 |
| **Total** |  |  |  |  |  |  |  |
| **Short reliable** | 0.00-2.99 | 4 | 6 | 1 | 2 | 2 | 5 |
| **Long reliable** | 3.00-4.49 | 3 | 2 | 4 | 6 | 4 | 4 |
| **At the NOE limit** | 4.50-5.49 | 0 | 3 | 2 | 0 | 0 | 4 |
| **Beyond the NOE limit** | 5.50-9.99 | 0 | 2 | 0 | 0 | 0 | 0 |
| **All** | 0.00-9.99 | 7 | 13 | 7 | 8 | 6 | 13 |

**Table S9**. Average absolute error (|Experimental distance – Theoretical distance| in Å) for correlations based on 6 data-points (mixing times) only found for each pulse program sorted by distance range.

|  | **Distance range (Å)** | **NOESY** | **NOESY ES** | **NOESY W5** | **ROESY** | **EASY ROESY** | **EASY ROESY ES** |
| --- | --- | --- | --- | --- | --- | --- | --- |
| **Short reliable** | 0.00-1.99 | 0.00 | - | - | - | - | 0.00 |
|  | 2.00-2.49 | 0.05 | 0.17 | - | - | 0.14 | 0.04 |
|  | 2.50-2.99 | 0.32 | 0.47 | 0.58 | 0.71 | 0.35 | 0.04 |
| **Long reliable** | 3.00-3.49 | 0.03 | - | 0.31 | 0.11 | 0.59 | 0.07 |
|  | 3.50-3.99 | 0.67 | 0.96 | 0.54 | 0.25 | 0.29 | 0.40 |
|  | 4.00-4.49 | - | - | - | - | - | - |
| **At the NOE limit** | 4.50-4.99 | - | 0.15 | 0.09 | - | - | 0.17 |
|  | 5.00-5.49 | - | 0.47 | 0.19 | - | - | - |
| **Beyond the NOE limit** | 5.50-5.99 | - | 0.81 | - | - | - | - |
|  | 6.00-6.49 | - | 1.21 | - | - | - | - |
|  | 6.50-6.99 | - | - | - | - | - | - |
|  | 7.00-7.49 | - | - | - | - | - | - |
|  | 7.50-7.99 | - | - | - | - | - | - |
|  | 8.00-8.49 | - | - | - | - | - | - |
|  | 8.50-8.99 | - | - | - | - | - | - |
|  | 9.00-9.49 | - | - | - | - | - | - |
|  | 9.50-9.99 | - | - | - | - | - | - |
| **Average** |  |  |  |  |  |  |  |
| **Short reliable** | 0.00-2.99 | 0.11 | 0.22 | 0.58 | 0.71 | 0.25 | 0.03 |
| **Long reliable** | 3.00-4.49 | 0.46 | 0.96 | 0.48 | 0.18 | 0.37 | 0.24 |
| **At the NOE limit** | 4.50-5.49 | - | 0.26 | 0.14 | - | - | 0.17 |
| **Beyond the NOE limit** | 5.50-9.99 | - | 1.01 | - | - | - | - |
| **All** | 0.00-9.99 | 0.26 | 0.46 | 0.40 | 0.31 | 0.33 | 0.13 |

**Table S10**. Average absolute error (%) for correlations based on 6 data-points (mixing times) only found for each pulse program sorted by distance range.

|  | **Distance range (Å)** | **NOESY** | **NOESY ES** | **NOESY W5** | **ROESY** | **EASY ROESY** | **EASY ROESY ES** |
| --- | --- | --- | --- | --- | --- | --- | --- |
| **Short reliable** | 0.00-1.99 | 0.0 | - | - | - | - | 0.0 |
|  | 2.00-2.49 | 2.2 | 6.9 | - | - | 5.8 | 1.6 |
|  | 2.50-2.99 | 11.3 | 16.7 | 21.2 | 25.2 | 13.3 | 1.4 |
| **Long reliable** | 3.00-3.49 | 1.0 | - | 9.2 | 3.3 | 18.2 | 2.3 |
|  | 3.50-3.99 | 17.7 | 25.7 | 14.5 | 6.6 | 7.8 | 10.7 |
|  | 4.00-4.49 | - | - | - | - | - | - |
| **At the NOE limit** | 4.50-4.99 | - | 3.1 | 1.9 | - | - | 3.4 |
|  | 5.00-5.49 | - | 9.3 | 3.7 | - | - | - |
| **Beyond the NOE limit** | 5.50-5.99 | - | 13.8 | - | - | - | - |
|  | 6.00-6.49 | - | 19.6 | - | - | - | - |
|  | 6.50-6.99 | - | - | - | - | - | - |
|  | 7.00-7.49 | - | - | - | - | - | - |
|  | 7.50-7.99 | - | - | - | - | - | - |
|  | 8.00-8.49 | - | - | - | - | - | - |
|  | 8.50-8.99 | - | - | - | - | - | - |
|  | 9.00-9.49 | - | - | - | - | - | - |
|  | 9.50-9.99 | - | - | - | - | - | - |
| **Average** |  |  |  |  |  |  |  |
| **Short reliable** | 0.00-2.99 | 4.0 | 8.5 | 21.2 | 25.2 | 9.5 | 1.2 |
| **Long reliable** | 3.00-4.49 | 12.1 | 25.7 | 13.2 | 5.0 | 10.4 | 6.5 |
| **At the NOE limit** | 4.50-5.49 | - | 5.2 | 2.8 | - | - | 3.4 |
| **Beyond the NOE limit** | 5.50-9.99 | - | 16.7 | - | - | - | - |
| **All** | 0.00-9.99 | 7.5 | 11.6 | 11.3 | 10.0 | 10.1 | 3.5 |

**Table S11**. Number of interproton correlations based on 5 data-points (mixing times) only found for each pulse program sorted by distance range.

|  | **Distance range (Å)** | **NOESY** | **NOESY ES** | **NOESY W5** | **ROESY** | **EASY ROESY** | **EASY ROESY ES** |
| --- | --- | --- | --- | --- | --- | --- | --- |
| **Short reliable** | 0.00-1.99 | 1 | 0 | 2 | 0 | 1 | 0 |
|  | 2.00-2.49 | 1 | 0 | 1 | 0 | 0 | 0 |
|  | 2.50-2.99 | 0 | 0 | 2 | 1 | 2 | 0 |
| **Long reliable** | 3.00-3.49 | 1 | 1 | 0 | 0 | 0 | 0 |
|  | 3.50-3.99 | 2 | 0 | 2 | 3 | 3 | 2 |
|  | 4.00-4.49 | 0 | 1 | 0 | 0 | 1 | 2 |
| **At the NOE limit** | 4.50-4.99 | 1 | 3 | 3 | 3 | 0 | 1 |
|  | 5.00-5.49 | 0 | 1 | 1 | 2 | 0 | 1 |
| **Beyond the NOE limit** | 5.50-5.99 | 0 | 3 | 2 | 0 | 0 | 1 |
|  | 6.00-6.49 | 0 | 0 | 0 | 0 | 0 | 0 |
|  | 6.50-6.99 | 0 | 0 | 0 | 0 | 0 | 0 |
|  | 7.00-7.49 | 0 | 0 | 0 | 0 | 0 | 0 |
|  | 7.50-7.99 | 0 | 0 | 0 | 1 | 0 | 0 |
|  | 8.00-8.49 | 0 | 0 | 0 | 0 | 0 | 0 |
|  | 8.50-8.99 | 0 | 0 | 0 | 0 | 0 | 0 |
|  | 9.00-9.49 | 0 | 0 | 0 | 0 | 0 | 0 |
|  | 9.50-9.99 | 0 | 0 | 0 | 0 | 0 | 0 |
| **Total** |  |  |  |  |  |  |  |
| **Short reliable** | 0.00-2.99 | 2 | 0 | 5 | 1 | 3 | 0 |
| **Long reliable** | 3.00-4.49 | 3 | 2 | 2 | 3 | 4 | 4 |
| **At the NOE limit** | 4.50-5.49 | 1 | 4 | 4 | 5 | 0 | 2 |
| **Beyond the NOE limit** | 5.50-9.99 | 0 | 3 | 2 | 1 | 0 | 1 |
| **All** | 0.00-9.99 | 6 | 9 | 13 | 10 | 7 | 7 |

**Table S12**. Average absolute error (|Experimental distance – Theoretical distance| in Å) for correlations based on 5 data-points (mixing times) only found for each pulse program sorted by distance range.

|  | **Distance range (Å)** | **NOESY** | **NOESY ES** | **NOESY W5** | **ROESY** | **EASY ROESY** | **EASY ROESY ES** |
| --- | --- | --- | --- | --- | --- | --- | --- |
| **Short reliable** | 0.00-1.99 | 0.14 | - | 0.15 | - | 0.23 | - |
|  | 2.00-2.49 | 0.06 | - | 0.22 | - | - | - |
|  | 2.50-2.99 | - | - | 0.64 | 0.23 | 0.20 | - |
| **Long reliable** | 3.00-3.49 | 0.15 | 0.25 | - | - | - | - |
|  | 3.50-3.99 | 0.84 | - | 0.48 | 0.66 | 0.42 | 0.37 |
|  | 4.00-4.49 | - | 0.19 | - | - | 0.17 | 0.11 |
| **At the NOE limit** | 4.50-4.99 | 0.07 | 0.76 | 0.27 | 0.26 | - | 0.09 |
|  | 5.00-5.49 | - | 0.40 | 0.15 | 0.81 | - | 0.13 |
| **Beyond the NOE limit** | 5.50-5.99 | - | 0.40 | 0.48 | - | - | 0.60 |
|  | 6.00-6.49 | - | - | - | - | - | - |
|  | 6.50-6.99 | - | - | - | - | - | - |
|  | 7.00-7.49 | - | - | - | - | - | - |
|  | 7.50-7.99 | - | - | - | 1.68 | - | - |
|  | 8.00-8.49 | - | - | - | - | - | - |
|  | 8.50-8.99 | - | - | - | - | - | - |
|  | 9.00-9.49 | - | - | - | - | - | - |
|  | 9.50-9.99 | - | - | - | - | - | - |
| **Average** |  |  |  |  |  |  |  |
| **Short reliable** | 0.00-2.99 | 0.10 | - | 0.36 | 0.23 | 0.21 | - |
| **Long reliable** | 3.00-4.49 | 0.61 | 0.22 | 0.48 | 0.66 | 0.36 | 0.24 |
| **At the NOE limit** | 4.50-5.49 | 0.07 | 0.67 | 0.24 | 0.48 | - | 0.11 |
| **Beyond the NOE limit** | 5.50-9.99 | - | 0.40 | 0.48 | 1.68 | - | 0.60 |
| **All** | 0.00-9.99 | 0.35 | 0.48 | 0.36 | 0.63 | 0.29 | 0.25 |

**Table S13**. Average absolute error (%) for correlations based on 5 data-points (mixing times) only found for each pulse program sorted by distance range.

|  | **Distance range (Å)** | **NOESY** | **NOESY ES** | **NOESY W5** | **ROESY** | **EASY ROESY** | **EASY ROESY ES** |
| --- | --- | --- | --- | --- | --- | --- | --- |
| **Short reliable** | 0.00-1.99 | 8.0 | - | 8.5 | - | 13.1 | - |
|  | 2.00-2.49 | 2.6 | - | 8.9 | - | - | - |
|  | 2.50-2.99 | - | - | 21.9 | 9.2 | 7.1 | - |
| **Long reliable** | 3.00-3.49 | 4.5 | 8.3 | - | - | - | - |
|  | 3.50-3.99 | 22.3 | - | 12.4 | 17.5 | 11.2 | 10.0 |
|  | 4.00-4.49 | - | 4.6 | - | - | 4.2 | 2.6 |
| **At the NOE limit** | 4.50-4.99 | 1.4 | 16.1 | 5.5 | 5.5 | - | 1.8 |
|  | 5.00-5.49 | - | 7.7 | 2.8 | 15.7 | - | 2.5 |
| **Beyond the NOE limit** | 5.50-5.99 | - | 7.0 | 8.2 | - | - | 10.5 |
|  | 6.00-6.49 | - | - | - | - | - | - |
|  | 6.50-6.99 | - | - | - | - | - | - |
|  | 7.00-7.49 | - | - | - | - | - | - |
|  | 7.50-7.99 | - | - | - | 21.8 | - | - |
|  | 8.00-8.49 | - | - | - | - | - | - |
|  | 8.50-8.99 | - | - | - | - | - | - |
|  | 9.00-9.49 | - | - | - | - | - | - |
|  | 9.50-9.99 | - | - | - | - | - | - |
| **Average** |  |  |  |  |  |  |  |
| **Short reliable** | 0.00-2.99 | 5.3 | - | 14.0 | 9.2 | 9.1 | - |
| **Long reliable** | 3.00-4.49 | 16.4 | 6.4 | 12.4 | 17.5 | 9.4 | 6.3 |
| **At the NOE limit** | 4.50-5.49 | 1.4 | 14.0 | 4.8 | 9.6 | - | 2.2 |
| **Beyond the NOE limit** | 5.50-9.99 | - | 7.0 | 8.2 | 21.8 | - | 10.5 |
| **All** | 0.00-9.99 | 10.2 | 10.0 | 10.0 | 13.1 | 9.3 | 5.7 |

**Table S14**. Number of interproton correlations based on 4 data-points (mixing times) only found for each pulse program sorted by distance range.

|  | **Distance range (Å)** | **NOESY** | **NOESY ES** | **NOESY W5** | **ROESY** | **EASY ROESY** | **EASY ROESY ES** |
| --- | --- | --- | --- | --- | --- | --- | --- |
| **Short reliable** | 0.00-1.99 | 0 | 0 | 0 | 0 | 0 | 0 |
|  | 2.00-2.49 | 1 | 0 | 0 | 2 | 0 | 1 |
|  | 2.50-2.99 | 2 | 0 | 0 | 0 | 0 | 0 |
| **Long reliable** | 3.00-3.49 | 0 | 0 | 1 | 1 | 1 | 1 |
|  | 3.50-3.99 | 3 | 3 | 2 | 2 | 1 | 2 |
|  | 4.00-4.49 | 2 | 1 | 3 | 0 | 2 | 1 |
| **At the NOE limit** | 4.50-4.99 | 5 | 4 | 2 | 3 | 1 | 4 |
|  | 5.00-5.49 | 1 | 1 | 3 | 0 | 1 | 0 |
| **Beyond the NOE limit** | 5.50-5.99 | 0 | 1 | 0 | 0 | 0 | 2 |
|  | 6.00-6.49 | 0 | 2 | 2 | 0 | 0 | 2 |
|  | 6.50-6.99 | 0 | 1 | 3 | 0 | 0 | 0 |
|  | 7.00-7.49 | 0 | 0 | 1 | 0 | 0 | 0 |
|  | 7.50-7.99 | 0 | 0 | 0 | 0 | 0 | 1 |
|  | 8.00-8.49 | 0 | 0 | 0 | 0 | 0 | 0 |
|  | 8.50-8.99 | 0 | 0 | 0 | 0 | 0 | 0 |
|  | 9.00-9.49 | 1 | 0 | 0 | 0 | 0 | 0 |
|  | 9.50-9.99 | 0 | 0 | 0 | 0 | 0 | 1 |
| **Total** |  |  |  |  |  |  |  |
| **Short reliable** | 0.00-2.99 | 3 | 0 | 0 | 2 | 0 | 1 |
| **Long reliable** | 3.00-4.49 | 5 | 4 | 6 | 3 | 4 | 4 |
| **At the NOE limit** | 4.50-5.49 | 6 | 5 | 5 | 3 | 2 | 4 |
| **Beyond the NOE limit** | 5.50-9.99 | 1 | 4 | 6 | 0 | 0 | 6 |
| **All** | 0.00-9.99 | 15 | 13 | 17 | 8 | 6 | 15 |

**Table S15**. Average absolute error (|Experimental distance – Theoretical distance| in Å) for correlations based on 4 data-points (mixing times) only found for each pulse program sorted by distance range.

|  | **Distance range (Å)** | **NOESY** | **NOESY ES** | **NOESY W5** | **ROESY** | **EASY ROESY** | **EASY ROESY ES** |
| --- | --- | --- | --- | --- | --- | --- | --- |
| **Short reliable** | 0.00-1.99 | - | - | - | - | - | - |
|  | 2.00-2.49 | 0.04 | - | - | 0.05 | - | 0.04 |
|  | 2.50-2.99 | 0.17 | - | - | - | - | - |
| **Long reliable** | 3.00-3.49 | - | - | 0.70 | 0.97 | 0.05 | 0.07 |
|  | 3.50-3.99 | 0.18 | 0.45 | 0.37 | 0.60 | 0.67 | 0.26 |
|  | 4.00-4.49 | 0.64 | 0.98 | 0.88 | - | 0.23 | 1.16 |
| **At the NOE limit** | 4.50-4.99 | 0.30 | 0.47 | 0.18 | 0.62 | 0.14 | 0.83 |
|  | 5.00-5.49 | 0.12 | 0.44 | 0.26 | - | 0.28 | - |
| **Beyond the NOE limit** | 5.50-5.99 | - | 0.25 | - | - | - | 0.38 |
|  | 6.00-6.49 | - | 0.10 | 0.68 | - | - | 0.51 |
|  | 6.50-6.99 | - | 0.77 | 1.08 | - | - | - |
|  | 7.00-7.49 | - | - | 0.35 | - | - | - |
|  | 7.50-7.99 | - | - | - | - | - | 3.62 |
|  | 8.00-8.49 | - | - | - | - | - | - |
|  | 8.50-8.99 | - | - | - | - | - | - |
|  | 9.00-9.49 | 3.17 | - | - | - | - | - |
|  | 9.50-9.99 | - | - | - | - | - | 3.79 |
| **Average** |  |  |  |  |  |  |  |
| **Short reliable** | 0.00-2.99 | 0.13 | - | - | 0.05 | - | 0.04 |
| **Long reliable** | 3.00-4.49 | 0.37 | 0.59 | 0.68 | 0.72 | 0.30 | 0.44 |
| **At the NOE limit** | 4.50-5.49 | 0.27 | 0.46 | 0.23 | 0.62 | 0.21 | 0.83 |
| **Beyond the NOE limit** | 5.50-9.99 | 3.17 | 0.31 | 0.82 | - | - | 1.53 |
| **All** | 0.00-9.99 | 0.47 | 0.45 | 0.60 | 0.52 | 0.27 | 0.95 |

**Table S16**. Average absolute error (%) for correlations based on 4 data-points (mixing times) only found for each pulse program sorted by distance range.

|  | **Distance range (Å)** | **NOESY** | **NOESY ES** | **NOESY W5** | **ROESY** | **EASY ROESY** | **EASY ROESY ES** |
| --- | --- | --- | --- | --- | --- | --- | --- |
| **Short reliable** | 0.00-1.99 | - | - | - | - | - | - |
|  | 2.00-2.49 | 1.6 | - | - | 2.1 | - | 1.6 |
|  | 2.50-2.99 | 6.4 | - | - | - | - | - |
| **Long reliable** | 3.00-3.49 | - | - | 23.3 | 28.4 | 1.5 | 2.1 |
|  | 3.50-3.99 | 4.9 | 11.8 | 10.2 | 16.4 | 18.2 | 6.9 |
|  | 4.00-4.49 | 15.0 | 23.8 | 20.6 | - | 5.3 | 27.2 |
| **At the NOE limit** | 4.50-4.99 | 6.2 | 9.9 | 3.7 | 13.5 | 2.9 | 17.1 |
|  | 5.00-5.49 | 2.3 | 8.5 | 4.8 | - | 5.2 | - |
| **Beyond the NOE limit** | 5.50-5.99 | - | 4.2 | - | - | - | 6.7 |
|  | 6.00-6.49 | - | 1.6 | 11.0 | - | - | 8.3 |
|  | 6.50-6.99 | - | 11.7 | 15.7 | - | - | - |
|  | 7.00-7.49 | - | - | 4.7 | - | - | - |
|  | 7.50-7.99 | - | - | - | - | - | 47.2 |
|  | 8.00-8.49 | - | - | - | - | - | - |
|  | 8.50-8.99 | - | - | - | - | - | - |
|  | 9.00-9.49 | 34.5 | - | - | - | - | - |
|  | 9.50-9.99 | - | - | - | - | - | 38.6 |
| **Average** |  |  |  |  |  |  |  |
| **Short reliable** | 0.00-2.99 | 4.8 | - | - | 2.1 | - | 1.6 |
| **Long reliable** | 3.00-4.49 | 9.0 | 14.8 | 17.6 | 20.4 | 7.5 | 10.8 |
| **At the NOE limit** | 4.50-5.49 | 5.5 | 9.6 | 4.4 | 13.5 | 4.0 | 17.1 |
| **Beyond the NOE limit** | 5.50-9.99 | 34.5 | 4.8 | 12.3 | - | - | 19.3 |
| **All** | 0.00-9.99 | 8.5 | 9.7 | 11.8 | 13.3 | 6.4 | 15.3 |

**3. Ungrouped data**

**Table S17**. Quantified distances obtained using the noesyph pulse program from the matrix generated from the diagonal integrals directly (NOESY), or after which the cross-peaks were adjusted to prevent the inclusion of noise (NOESY adjusted cross-peaks). The theoretical population-averaged interproton distances as determined by Butts *et. al.*^[3]^ are given for each correlation, and used to calculate the error (Exp. – Theor.) as well as the absolute error (|%|). The number of data-points included (# t_mix_) for the distance determination, as well as the coefficient of determination (R^2^) of the linear regression are given. At the bottom of the table, the total number of quantified correlations as well as the number of over- and underestimated correlations are given. The proton-proton corelation used as a reference for distance calculation is being underscored.

|  |  |  |  |  | **NOESY** | | | | | **NOESY adjusted cross-peaks** | | | | |
| --- | --- | --- | --- | --- | --- | --- | --- | --- | --- | --- | --- | --- | --- | --- |
|  |  | **H_a_** | **H_b_** | **r_ab_**  **Theor. (Å)** | **# t_mix_** | **R^2^** | **r_ab_ (Å)** | **Exp.**  **-**  **Theor. (Å)** | **\|%\|** | **# t_mix_** | **R^2^** | **r_ab_ (Å)** | **Exp.**  **-**  **Theor. (Å)** | **\|%\|** |
| **Short reliable** | **0.00-1.99** | **20a** | **11b/20b** | 1.76 | 5 | 0.999 | 1.90 | 0.14 | 8.0 |  |  |  |  |  |
|  |  | **15a** | **15b** | 1.76 | 6 | 0.990 | 1.76 | 0.00 | 0.0 |  |  |  |  |  |
|  |  | **11a** | **11b/20b** | 1.76 | 7 | 0.992 | 1.84 | 0.08 | 4.5 | 7 | 0.985 | 1.68 | -0.08 | 4.5 |
|  |  | **17a** | **17b** | 1.77 | 7 | 0.983 | 1.75 | -0.02 | 1.1 | 6 | 0.961 | 1.77 | 0.00 | 0.0 |
|  |  | **18a** | **18b** | 1.77 | 7 | 0.998 | 1.74 | -0.03 | 1.7 | 7 | 0.986 | 1.90 | 0.13 | 7.3 |
|  | **2.00-2.49** | **20a** | **15a** | 2.24 | 7 | 0.998 | 2.12 | -0.12 | 5.4 | 7 | 0.998 | 1.92 | -0.32 | 14.3 |
|  |  | **15b** | **13** | 2.26 |  |  |  |  |  | 5 | 0.962 | 2.29 | 0.03 | 1.3 |
|  |  | **12** | **23ab** | 2.27 | 7 | 0.999 | 2.29 | 0.02 | 0.9 | 7 | 0.999 | 2.09 | -0.18 | 7.9 |
|  |  | **22** | **23ab** | 2.30 | 7 | 0.999 | 2.36 | 0.06 | 2.6 | 7 | 0.999 | 2.14 | -0.16 | 7.0 |
|  |  | **12** | **13** | 2.35 | 7 | 0.999 | 2.30 | -0.05 | 2.1 | 7 | 0.996 | 2.17 | -0.18 | 7.7 |
|  |  | **12** | **11a** | 2.35 | 5 | 0.963 | 2.41 | 0.06 | 2.6 |  |  |  |  |  |
|  |  | **8** | **18b** | 2.37 | 7 | 1.000 | 2.19 | -0.18 | 7.6 | 7 | 1.000 | 2.00 | -0.37 | 15.6 |
|  |  | **1** | **16** | 2.39 | 7 | 1.000 | 2.23 | -0.16 | 6.7 | 7 | 1.000 | 2.03 | -0.36 | 15.1 |
|  |  | **22** | **11b/20b** | 2.39 | 7 | 1.000 | 2.49 | 0.10 | 4.2 | 7 | 1.000 | 2.32 | -0.07 | 2.9 |
|  |  | **14** | **13** | 2.41 | 7 | 0.999 | 2.38 | -0.03 | 1.2 | 7 | 0.996 | 2.26 | -0.15 | 6.2 |
|  |  | **8** | **17a** | 2.42 | 7 | 1.000 | 2.32 | -0.10 | 4.1 | 7 | 1.000 | 2.11 | -0.31 | 12.8 |
|  |  | **18a** | **17b** | 2.43 | 7 | 0.987 | 2.38 | -0.05 | 2.1 | 7 | 0.987 | 2.15 | -0.28 | 11.5 |
|  |  | **18b** | **17a** | 2.43 | 6 | 0.981 | 2.46 | 0.03 | 1.2 |  |  |  |  |  |
|  |  | **18b** | **11b/20b** | 2.45 |  |  |  |  |  | 5 | 0.968 | 2.72 | 0.27 | 11.0 |
|  |  | **16** | **15a** | 2.48 | 7 | 0.980 | 2.44 | -0.04 | 1.6 | 6 | 0.984 | 2.71 | 0.23 | 9.3 |
|  |  | **16** | **15b** | 2.48 | 6 | 0.983 | 2.40 | -0.08 | 3.2 | 6 | 0.983 | 2.17 | -0.31 | 12.5 |
|  |  | **3** | **2** | 2.48 |  |  |  |  |  | 6 | 0.958 | 2.65 | 0.17 | 6.9 |
|  |  | **14** | **15a** | 2.49 | 7 | 0.998 | 2.42 | -0.07 | 2.8 |  |  |  |  |  |
|  |  | **1** | **2** | 2.49 | 4 | 0.964 | 2.53 | 0.04 | 1.6 |  |  |  |  |  |
|  | **2.50-2.99** | **4** | **3** | 2.50 | 4 | 0.988 | 2.60 | 0.10 | 4.0 |  |  |  |  |  |
|  |  | **14** | **15b** | 2.55 | 7 | 0.958 | 2.50 | -0.05 | 2.0 | 7 | 0.958 | 2.26 | -0.29 | 11.4 |
|  |  | **23ab** | **14** | 2.59 | 7 | 1.000 | 2.52 | -0.07 | 2.7 | 7 | 1.000 | 2.35 | -0.24 | 9.3 |
|  |  | **8** | **11b/20b** | 2.63 | 7 | 0.999 | 2.61 | -0.02 | 0.8 | 7 | 0.999 | 2.46 | -0.17 | 6.5 |
|  |  | **16** | **17b** | 2.71 | 7 | 1.000 | 2.58 | -0.13 | 4.8 | 7 | 1.000 | 2.37 | -0.34 | 12.5 |
|  |  | **18a** | **17a** | 2.73 | 4 | 0.950 | 2.97 | 0.24 | 8.8 |  |  |  |  |  |
|  |  | **12** | **14** | 2.74 | 7 | 0.999 | 2.67 | -0.07 | 2.6 | 7 | 0.999 | 2.42 | -0.32 | 11.7 |
|  |  | **1** | **17b** | 2.79 | 7 | 0.999 | 2.68 | -0.11 | 3.9 | 7 | 0.999 | 2.47 | -0.32 | 11.5 |
|  |  | **18a** | **11b/20b** | 2.82 | 6 | 0.970 | 3.14 | 0.32 | 11.3 | 7 | 0.964 | 3.00 | 0.18 | 6.4 |
|  |  | **12** | **11b/20b** | 2.93 |  |  |  |  |  |  |  |  |  |  |
| **Long reliable** | **3.00-3.49** | **8** | **13** | 3.01 | 6 | 0.952 | 2.98 | -0.03 | 1.0 | 6 | 0.952 | 2.70 | -0.31 | 10.3 |
|  |  | **22** | **18b** | 3.16 | 7 | 0.999 | 2.95 | -0.21 | 6.6 | 7 | 0.999 | 2.75 | -0.41 | 13.0 |
|  |  | **23ab** | **11b/20b** | 3.24 | 7 | 0.957 | 3.58 | 0.34 | 10.5 | 7 | 0.975 | 3.37 | 0.13 | 4.0 |
|  |  | **20a** | **14** | 3.36 | 7 | 0.985 | 3.36 | 0.00 | 0.0 | 7 | 0.985 | 3.04 | -0.32 | 9.5 |
|  |  | **16** | **20a** | 3.37 | 5 | 0.951 | 3.52 | 0.15 | 4.5 | 5 | 0.977 | 3.25 | -0.12 | 3.6 |
|  |  | **11a** | **13** | 3.40 | 7 | 0.976 | 3.60 | 0.20 | 5.9 | 7 | 0.972 | 3.41 | 0.01 | 0.3 |
|  |  | **22** | **20a** | 3.42 |  |  |  |  |  |  |  |  |  |  |
|  | **3.50-3.99** | **15a** | **13** | 3.54 |  |  |  |  |  |  |  |  |  |  |
|  |  | **8** | **17b** | 3.61 | 4 | 0.959 | 3.97 | 0.36 | 10.0 |  |  |  |  |  |
|  |  | **11b/20b** | **13** | 3.61 | 7 | 0.961 | 4.19 | 0.58 | 16.1 | 5 | 0.973 | 4.65 | 1.04 | 28.8 |
|  |  | **16** | **18a** | 3.63 | 7 | 0.993 | 3.68 | 0.05 | 1.4 | 7 | 0.978 | 3.49 | -0.14 | 3.9 |
|  |  | **12** | **8** | 3.65 | 7 | 0.969 | 3.67 | 0.02 | 0.5 | 6 | 0.982 | 3.47 | -0.18 | 4.9 |
|  |  | **11b/20b** | **15a** | 3.69 | 5 | 0.980 | 2.90 | -0.79 | 21.4 | 5 | 0.988 | 2.69 | -1.00 | 27.1 |
|  |  | **23ab** | **8** | 3.73 |  |  |  |  |  | 5 | 0.963 | 3.97 | 0.24 | 6.4 |
|  |  | **20a** | **15b** | 3.75 | 6 | 0.986 | 2.81 | -0.94 | 25.1 | 5 | 0.991 | 2.76 | -0.99 | 26.4 |
|  |  | **23ab** | **13** | 3.76 |  |  |  |  |  | 4 | 0.963 | 3.88 | 0.12 | 3.2 |
|  |  | **8** | **18a** | 3.83 | 5 | 0.983 | 2.94 | -0.89 | 23.2 | 6 | 0.952 | 3.01 | -0.82 | 21.4 |
|  |  | **16** | **17a** | 3.84 |  |  |  |  |  |  |  |  |  |  |
|  |  | **1** | **15b** | 3.86 |  |  |  |  |  | 5 | 0.951 | 3.70 | -0.16 | 4.1 |
|  |  | **22** | **14** | 3.86 | 7 | 0.976 | 3.90 | 0.04 | 1.0 | 7 | 0.979 | 3.56 | -0.30 | 7.8 |
|  |  | **8** | **11a** | 3.88 | 4 | 0.953 | 4.03 | 0.15 | 3.9 |  |  |  |  |  |
|  |  | **16** | **18b** | 3.89 | 6 | 0.956 | 4.29 | 0.40 | 10.3 | 6 | 0.993 | 4.01 | 0.12 | 3.1 |
|  |  | **23ab** | **11a** | 3.96 | 4 | 0.960 | 3.92 | -0.04 | 1.0 | 4 | 0.968 | 3.64 | -0.32 | 8.1 |
|  |  | **14** | **11b/20b** | 3.96 |  |  |  |  |  |  |  |  |  |  |
|  |  | **8** | **14** | 3.96 | 7 | 0.968 | 3.93 | -0.03 | 0.8 | 6 | 0.951 | 3.79 | -0.17 | 4.3 |
|  |  | **22** | **8** | 3.96 | 7 | 0.989 | 3.82 | -0.14 | 3.5 | 7 | 0.987 | 3.53 | -0.43 | 10.9 |
|  | **4.00-4.49** | **1** | **17a** | 4.06 |  |  |  |  |  |  |  |  |  |  |
|  |  | **16** | **11b/20b** | 4.07 |  |  |  |  |  |  |  |  |  |  |
|  |  | **8** | **15b** | 4.09 |  |  |  |  |  |  |  |  |  |  |
|  |  | **16** | **8** | 4.14 |  |  |  |  |  | 4 | 0.971 | 3.17 | -0.97 | 23.4 |
|  |  | **11b/20b** | **17a** | 4.17 | 4 | 0.974 | 4.12 | -0.05 | 1.2 | 4 | 0.974 | 3.73 | -0.44 | 10.6 |
|  |  | **23ab** | **18b** | 4.27 | 4 | 0.964 | 5.50 | 1.23 | 28.8 | 4 | 0.989 | 5.55 | 1.28 | 30.0 |
|  |  | **12** | **15b** | 4.27 |  |  |  |  |  |  |  |  |  |  |
|  |  | **4** | **13** | 4.43 | 7 | 0.977 | 4.29 | -0.14 | 3.2 | 6 | 0.964 | 4.70 | 0.27 | 6.1 |
|  |  | **22** | **18a** | 4.47 |  |  |  |  |  |  |  |  |  |  |
| **At the NOE limit** | **4.50-4.99** | **18b** | **15a** | 4.55 |  |  |  |  |  |  |  |  |  |  |
|  |  | **11b/20b** | **15b** | 4.59 |  |  |  |  |  |  |  |  |  |  |
|  |  | **1** | **15a** | 4.62 |  |  |  |  |  |  |  |  |  |  |
|  |  | **8** | **15a** | 4.63 | 4 | 0.970 | 4.83 | 0.20 | 4.3 | 4 | 0.959 | 4.60 | -0.03 | 0.6 |
|  |  | **17b** | **15b** | 4.64 |  |  |  |  |  |  |  |  |  |  |
|  |  | **22** | **12** | 4.65 |  |  |  |  |  |  |  |  |  |  |
|  |  | **14** | **18b** | 4.66 |  |  |  |  |  |  |  |  |  |  |
|  |  | **23ab** | **15a** | 4.68 |  |  |  |  |  |  |  |  |  |  |
|  |  | **8** | **20a** | 4.71 |  |  |  |  |  |  |  |  |  |  |
|  |  | **18b** | **13** | 4.73 |  |  |  |  |  |  |  |  |  |  |
|  |  | **4** | **11a** | 4.77 | 4 | 0.988 | 4.51 | -0.26 | 5.5 | 4 | 0.964 | 5.01 | 0.24 | 5.0 |
|  |  | **23ab** | **15b** | 4.81 |  |  |  |  |  |  |  |  |  |  |
|  |  | **22** | **15a** | 4.81 | 4 | 0.993 | 5.11 | 0.30 | 6.2 |  |  |  |  |  |
|  |  | **2** | **17b** | 4.82 |  |  |  |  |  |  |  |  |  |  |
|  |  | **14** | **11a** | 4.87 |  |  |  |  |  |  |  |  |  |  |
|  |  | **20a** | **13** | 4.89 |  |  |  |  |  |  |  |  |  |  |
|  |  | **18a** | **15a** | 4.89 |  |  |  |  |  |  |  |  |  |  |
|  |  | **4** | **8** | 4.89 | 5 | 0.966 | 4.96 | 0.07 | 1.4 | 6 | 0.956 | 4.47 | -0.42 | 8.6 |
|  |  | **4** | **11b/20b** | 4.90 |  |  |  |  |  |  |  |  |  |  |
|  |  | **1** | **18a** | 4.90 | 4 | 0.997 | 5.57 | 0.67 | 13.7 |  |  |  |  |  |
|  |  | **20a** | **17b** | 4.90 |  |  |  |  |  |  |  |  |  |  |
|  |  | **12** | **15a** | 4.98 | 4 | 0.975 | 4.92 | -0.06 | 1.2 |  |  |  |  |  |
|  |  | **1** | **13** | 4.99 |  |  |  |  |  |  |  |  |  |  |
|  | **5.00-5.49** | **4** | **1** | 5.00 |  |  |  |  |  |  |  |  |  |  |
|  |  | **1** | **8** | 5.06 |  |  |  |  |  |  |  |  |  |  |
|  |  | **17b** | **13** | 5.17 |  |  |  |  |  |  |  |  |  |  |
|  |  | **4** | **17a** | 5.17 | 4 | 0.956 | 5.05 | -0.12 | 2.3 | 4 | 0.961 | 4.71 | -0.46 | 8.9 |
|  |  | **22** | **13** | 5.19 |  |  |  |  |  |  |  |  |  |  |
|  |  | **1** | **18b** | 5.32 |  |  |  |  |  |  |  |  |  |  |
|  |  | **20a** | **17a** | 5.36 |  |  |  |  |  |  |  |  |  |  |
|  |  | **12** | **18b** | 5.38 |  |  |  |  |  |  |  |  |  |  |
|  |  | **2** | **15b** | 5.44 |  |  |  |  |  |  |  |  |  |  |
| **Beyond the NOE limit** | **5.50-5.99** | **22** | **15b** | 5.56 |  |  |  |  |  |  |  |  |  |  |
|  |  | **4** | **17b** | 5.71 |  |  |  |  |  |  |  |  |  |  |
|  |  | **22** | **16** | 5.71 |  |  |  |  |  |  |  |  |  |  |
|  |  | **1** | **20a** | 5.74 |  |  |  |  |  |  |  |  |  |  |
|  |  | **18a** | **13** | 5.89 |  |  |  |  |  |  |  |  |  |  |
|  |  | **3** | **13** | 5.90 |  |  |  |  |  |  |  |  |  |  |
|  |  | **3** | **17b** | 5.95 |  |  |  |  |  |  |  |  |  |  |
|  | **6.00-6.49** | **4** | **16** | 6.07 |  |  |  |  |  |  |  |  |  |  |
|  |  | **3** | **17a** | 6.09 |  |  |  |  |  |  |  |  |  |  |
|  |  | **3** | **15b** | 6.11 |  |  |  |  |  |  |  |  |  |  |
|  |  | **2** | **13** | 6.11 |  |  |  |  |  |  |  |  |  |  |
|  |  | **22** | **11a** | 6.14 |  |  |  |  |  |  |  |  |  |  |
|  |  | **3** | **16** | 6.16 |  |  |  |  |  |  |  |  |  |  |
|  | **6.50-6.99** | **3** | **8** | 6.53 |  |  |  |  |  |  |  |  |  |  |
|  |  | **2** | **8** | 6.59 |  |  |  |  |  |  |  |  |  |  |
|  |  | **11a** | **15a** | 6.74 |  |  |  |  |  |  |  |  |  |  |
|  |  | **4** | **14** | 6.82 |  |  |  |  |  |  |  |  |  |  |
|  |  | **12** | **18a** | 6.94 |  |  |  |  |  |  |  |  |  |  |
|  | **7.00-7.49** | **2** | **11b/20b** | 7.43 |  |  |  |  |  |  |  |  |  |  |
|  | **7.50-7.99** | **2** | **15a** | 7.67 |  |  |  |  |  |  |  |  |  |  |
|  |  | **1** | **22** | 7.71 |  |  |  |  |  |  |  |  |  |  |
|  |  | **2** | **14** | 7.80 |  |  |  |  |  |  |  |  |  |  |
|  | **8.00-8.49** | **3** | **14** | 8.09 |  |  |  |  |  |  |  |  |  |  |
|  | **8.50-8.99** | **4** | **22** | 8.70 |  |  |  |  |  |  |  |  |  |  |
|  | **9.00-9.49** | **3** | **20a** | 9.19 | 4 | 0.964 | 6.02 | -3.17 | 34.5 | 4 | 0.964 | 5.89 | -3.30 | 35.9 |
|  | **9.50-9.99** | **2** | **22** | 9.82 |  |  |  |  |  | 4 | 0.984 | 6.93 | -2.89 | 29.4 |
|  |  |  |  |  | **# Distances** | |  |  |  | **# Distances** | |  |  |  |
| **Total # of distances** | | | | 127 | 60 |  |  |  |  | 55 |  |  |  |  |
| **# Underestimated** | | | |  | 32 |  |  |  |  | 39 |  |  |  |  |
| **# Overestimated** | | | |  | 26 |  |  |  |  | 15 |  |  |  |  |

**Table S18**. Quantified distances for the noesyesgpph pulse program from the matrix generated from the diagonal integrals directly (NOESY ES) or after which the cross-peaks were adjusted to prevent the inclusion of noise (NOESY ES adjusted cross-peaks). The theoretical population-averaged interproton distances as determined by Butts *et. al.*^[3]^ are given for each correlation, and used to calculate the error (Exp. – Theor.) as well as the absolute error (|%|). The number of data-points included (# t_mix_) for the distance determination, as well as the coefficient of determination (R^2^) of the linear regression are given. At the bottom of the table, the total number of quantified correlations as well as the number of over- and underestimated correlations are given. The proton-proton correlation used as a reference for distance calculation is being underscored.

|  |  |  |  |  | **NOESY ES** | | | | | **NOESY ES adjusted cross-peaks** | | | | |
| --- | --- | --- | --- | --- | --- | --- | --- | --- | --- | --- | --- | --- | --- | --- |
|  |  | **H_a_** | **H_b_** | **r_ab_**  **Theor. (Å)** | **# t_mix_** | **R^2^** | **r_ab_ (Å)** | **Exp.**  **-**  **Theor. (Å)** | **\|%\|** | **# t_mix_** | **R^2^** | **r_ab_ (Å)** | **Exp.**  **-**  **Theor. (Å)** | **\|%\|** |
| **Short reliable** | **0.00-1.99** | **20a** | **11b/20b** | 1.76 | 7 | 0.996 | 1.83 | 0.07 | 4.0 |  |  |  |  |  |
|  |  | **15a** | **15b** | 1.76 | 7 | 0.957 | 1.76 | 0.00 | 0.0 | 5 | 0.965 | 1.76 | 0.00 | 0.0 |
|  |  | **11a** | **11b/20b** | 1.76 |  |  |  |  |  |  |  |  |  |  |
|  |  | **17a** | **17b** | 1.77 |  |  |  |  |  | 7 | 0.994 | 1.77 | 0.00 | 0.0 |
|  |  | **18a** | **18b** | 1.77 |  |  |  |  |  |  |  |  |  |  |
|  | **2.00-2.49** | **20a** | **15a** | 2.24 | 7 | 0.999 | 2.10 | -0.14 | 6.3 | 7 | 0.999 | 1.61 | -0.63 | 28.1 |
|  |  | **15b** | **13** | 2.26 |  |  |  |  |  |  |  |  |  |  |
|  |  | **12** | **23ab** | 2.27 | 7 | 1.000 | 2.22 | -0.05 | 2.2 | 7 | 1.000 | 1.72 | -0.55 | 24.2 |
|  |  | **22** | **23ab** | 2.30 | 6 | 0.997 | 2.29 | -0.01 | 0.4 | 6 | 0.994 | 1.78 | -0.52 | 22.6 |
|  |  | **12** | **13** | 2.35 | 7 | 0.999 | 2.24 | -0.11 | 4.7 | 7 | 0.999 | 1.77 | -0.58 | 24.7 |
|  |  | **12** | **11a** | 2.35 | 7 | 0.984 | 1.98 | -0.37 | 15.7 | 6 | 0.958 | 1.56 | -0.79 | 33.6 |
|  |  | **8** | **18b** | 2.37 | 7 | 1.000 | 2.13 | -0.24 | 10.1 | 7 | 1.000 | 1.66 | -0.71 | 30.0 |
|  |  | **1** | **16** | 2.39 | 7 | 1.000 | 2.17 | -0.22 | 9.2 | 7 | 1.000 | 1.67 | -0.72 | 30.1 |
|  |  | **22** | **11b/20b** | 2.39 | 7 | 1.000 | 2.41 | 0.02 | 0.8 | 7 | 1.000 | 1.88 | -0.51 | 21.3 |
|  |  | **14** | **13** | 2.41 |  |  |  |  |  |  |  |  |  |  |
|  |  | **8** | **17a** | 2.42 | 7 | 1.000 | 2.24 | -0.18 | 7.4 | 7 | 1.000 | 1.73 | -0.69 | 28.5 |
|  |  | **18a** | **17b** | 2.43 | 6 | 0.990 | 1.99 | -0.44 | 18.1 | 6 | 0.990 | 1.53 | -0.90 | 37.0 |
|  |  | **18b** | **17a** | 2.43 | 6 | 0.974 | 2.44 | 0.01 | 0.4 |  |  |  |  |  |
|  |  | **18b** | **11b/20b** | 2.45 | 6 | 0.973 | 2.66 | 0.21 | 8.6 | 7 | 0.993 | 2.34 | -0.11 | 4.5 |
|  |  | **16** | **15a** | 2.48 | 7 | 0.988 | 2.40 | -0.08 | 3.2 | 6 | 0.970 | 2.05 | -0.43 | 17.3 |
|  |  | **16** | **15b** | 2.48 | 7 | 0.967 | 2.34 | -0.14 | 5.6 | 7 | 0.967 | 1.79 | -0.69 | 27.8 |
|  |  | **3** | **2** | 2.48 |  |  |  |  |  | 4 | 0.969 | 2.71 | 0.23 | 9.3 |
|  |  | **14** | **15a** | 2.49 | 6 | 0.958 | 2.30 | -0.19 | 7.6 |  |  |  |  |  |
|  |  | **1** | **2** | 2.49 |  |  |  |  |  | 4 | 0.970 | 2.46 | -0.03 | 1.2 |
|  | **2.50-2.99** | **4** | **3** | 2.50 |  |  |  |  |  |  |  |  |  |  |
|  |  | **14** | **15b** | 2.55 |  |  |  |  |  |  |  |  |  |  |
|  |  | **23ab** | **14** | 2.59 |  |  |  |  |  | 4 | 0.981 | 2.15 | -0.44 | 17.0 |
|  |  | **8** | **11b/20b** | 2.63 | 7 | 0.999 | 2.57 | -0.06 | 2.3 | 7 | 0.999 | 2.02 | -0.61 | 23.2 |
|  |  | **16** | **17b** | 2.71 | 7 | 1.000 | 2.50 | -0.21 | 7.7 | 7 | 1.000 | 1.93 | -0.78 | 28.8 |
|  |  | **18a** | **17a** | 2.73 |  |  |  |  |  |  |  |  |  |  |
|  |  | **12** | **14** | 2.74 |  |  |  |  |  |  |  |  |  |  |
|  |  | **1** | **17b** | 2.79 | 7 | 1.000 | 2.60 | -0.19 | 6.8 | 7 | 1.000 | 2.06 | -0.73 | 26.2 |
|  |  | **18a** | **11b/20b** | 2.82 | 6 | 0.982 | 2.35 | -0.47 | 16.7 | 6 | 0.978 | 1.97 | -0.85 | 30.1 |
|  |  | **12** | **11b/20b** | 2.93 |  |  |  |  |  |  |  |  |  |  |
| **Long reliable** | **3.00-3.49** | **8** | **13** | 3.01 | 5 | 0.970 | 2.76 | -0.25 | 8.3 |  |  |  |  |  |
|  |  | **22** | **18b** | 3.16 | 7 | 0.997 | 2.88 | -0.28 | 8.9 | 7 | 0.997 | 2.28 | -0.88 | 27.8 |
|  |  | **23ab** | **11b/20b** | 3.24 | 7 | 0.977 | 3.55 | 0.31 | 9.6 | 7 | 0.979 | 2.75 | -0.49 | 15.1 |
|  |  | **20a** | **14** | 3.36 |  |  |  |  |  | 7 | 0.965 | 2.05 | -1.31 | 39.0 |
|  |  | **16** | **20a** | 3.37 |  |  |  |  |  |  |  |  |  |  |
|  |  | **11a** | **13** | 3.40 |  |  |  |  |  |  |  |  |  |  |
|  |  | **22** | **20a** | 3.42 |  |  |  |  |  |  |  |  |  |  |
|  | **3.50-3.99** | **15a** | **13** | 3.54 |  |  |  |  |  |  |  |  |  |  |
|  |  | **8** | **17b** | 3.61 |  |  |  |  |  | 4 | 0.975 | 2.74 | -0.87 | 24.1 |
|  |  | **11b/20b** | **13** | 3.61 | 7 | 0.955 | 4.08 | 0.47 | 13.0 | 6 | 0.978 | 3.20 | -0.41 | 11.4 |
|  |  | **16** | **18a** | 3.63 |  |  |  |  |  | 4 | 0.964 | 2.83 | -0.80 | 22.0 |
|  |  | **12** | **8** | 3.65 | 4 | 0.986 | 3.81 | 0.16 | 4.4 |  |  |  |  |  |
|  |  | **11b/20b** | **15a** | 3.69 | 6 | 0.967 | 2.82 | -0.87 | 23.6 | 5 | 0.985 | 2.75 | -0.94 | 25.5 |
|  |  | **23ab** | **8** | 3.73 |  |  |  |  |  |  |  |  |  |  |
|  |  | **20a** | **15b** | 3.75 | 6 | 0.978 | 2.71 | -1.04 | 27.7 | 5 | 0.985 | 2.26 | -1.49 | 39.7 |
|  |  | **23ab** | **13** | 3.76 |  |  |  |  |  |  |  |  |  |  |
|  |  | **8** | **18a** | 3.83 |  |  |  |  |  |  |  |  |  |  |
|  |  | **16** | **17a** | 3.84 |  |  |  |  |  |  |  |  |  |  |
|  |  | **1** | **15b** | 3.86 | 4 | 0.956 | 4.38 | 0.52 | 13.5 | 4 | 0.958 | 3.42 | -0.44 | 11.4 |
|  |  | **22** | **14** | 3.86 | 7 | 0.959 | 2.81 | -1.05 | 27.2 | 6 | 0.962 | 2.21 | -1.65 | 42.7 |
|  |  | **8** | **11a** | 3.88 |  |  |  |  |  |  |  |  |  |  |
|  |  | **16** | **18b** | 3.89 | 4 | 0.973 | 4.57 | 0.68 | 17.5 |  |  |  |  |  |
|  |  | **23ab** | **11a** | 3.96 |  |  |  |  |  |  |  |  |  |  |
|  |  | **14** | **11b/20b** | 3.96 |  |  |  |  |  |  |  |  |  |  |
|  |  | **8** | **14** | 3.96 |  |  |  |  |  |  |  |  |  |  |
|  |  | **22** | **8** | 3.96 | 7 | 0.995 | 3.73 | -0.23 | 5.8 | 7 | 0.997 | 3.01 | -0.95 | 24.0 |
|  | **4.00-4.49** | **1** | **17a** | 4.06 |  |  |  |  |  |  |  |  |  |  |
|  |  | **16** | **11b/20b** | 4.07 | 4 | 0.996 | 5.09 | 1.02 | 25.1 |  |  |  |  |  |
|  |  | **8** | **15b** | 4.09 |  |  |  |  |  |  |  |  |  |  |
|  |  | **16** | **8** | 4.14 |  |  |  |  |  |  |  |  |  |  |
|  |  | **11b/20b** | **17a** | 4.17 | 5 | 0.970 | 4.36 | 0.19 | 4.6 | 5 | 0.970 | 3.35 | -0.82 | 19.7 |
|  |  | **23ab** | **18b** | 4.27 |  |  |  |  |  |  |  |  |  |  |
|  |  | **12** | **15b** | 4.27 |  |  |  |  |  |  |  |  |  |  |
|  |  | **4** | **13** | 4.43 | 7 | 0.993 | 4.11 | -0.32 | 7.2 | 7 | 0.996 | 3.31 | -1.12 | 25.3 |
|  |  | **22** | **18a** | 4.47 |  |  |  |  |  |  |  |  |  |  |
| **At the NOE limit** | **4.50-4.99** | **18b** | **15a** | 4.55 |  |  |  |  |  |  |  |  |  |  |
|  |  | **11b/20b** | **15b** | 4.59 | 5 | 0.965 | 3.90 | -0.69 | 15.0 |  |  |  |  |  |
|  |  | **1** | **15a** | 4.62 | 5 | 0.954 | 3.84 | -0.78 | 16.9 |  |  |  |  |  |
|  |  | **8** | **15a** | 4.63 | 5 | 0.989 | 4.07 | -0.56 | 12.1 | 4 | 0.961 | 4.35 | -0.28 | 6.0 |
|  |  | **17b** | **15b** | 4.64 | 6 | 0.978 | 4.58 | -0.06 | 1.3 |  |  |  |  |  |
|  |  | **22** | **12** | 4.65 |  |  |  |  |  | 4 | 0.976 | 4.92 | 0.27 | 5.8 |
|  |  | **14** | **18b** | 4.66 | 4 | 0.981 | 3.35 | -1.31 | 28.1 | 5 | 0.977 | 2.47 | -2.19 | 47.0 |
|  |  | **23ab** | **15a** | 4.68 |  |  |  |  |  |  |  |  |  |  |
|  |  | **8** | **20a** | 4.71 |  |  |  |  |  |  |  |  |  |  |
|  |  | **18b** | **13** | 4.73 | 4 | 0.969 | 4.64 | -0.09 | 1.9 |  |  |  |  |  |
|  |  | **4** | **11a** | 4.77 |  |  |  |  |  |  |  |  |  |  |
|  |  | **23ab** | **15b** | 4.81 |  |  |  |  |  | 4 | 0.985 | 4.19 | -0.62 | 12.9 |
|  |  | **22** | **15a** | 4.81 | 4 | 0.971 | 4.92 | 0.11 | 2.3 |  |  |  |  |  |
|  |  | **2** | **17b** | 4.82 | 4 | 0.976 | 5.17 | 0.35 | 7.3 | 4 | 0.960 | 4.14 | -0.68 | 14.1 |
|  |  | **14** | **11a** | 4.87 |  |  |  |  |  |  |  |  |  |  |
|  |  | **20a** | **13** | 4.89 |  |  |  |  |  |  |  |  |  |  |
|  |  | **18a** | **15a** | 4.89 |  |  |  |  |  |  |  |  |  |  |
|  |  | **4** | **8** | 4.89 | 6 | 0.958 | 4.65 | -0.24 | 4.9 | 7 | 0.979 | 4.32 | -0.57 | 11.7 |
|  |  | **4** | **11b/20b** | 4.90 |  |  |  |  |  | 5 | 0.956 | 4.49 | -0.41 | 8.4 |
|  |  | **1** | **18a** | 4.90 |  |  |  |  |  |  |  |  |  |  |
|  |  | **20a** | **17b** | 4.90 |  |  |  |  |  |  |  |  |  |  |
|  |  | **12** | **15a** | 4.98 |  |  |  |  |  |  |  |  |  |  |
|  |  | **1** | **13** | 4.99 |  |  |  |  |  | 5 | 0.964 | 4.53 | -0.46 | 9.2 |
|  | **5.00-5.49** | **4** | **1** | 5.00 |  |  |  |  |  |  |  |  |  |  |
|  |  | **1** | **8** | 5.06 | 6 | 0.980 | 4.59 | -0.47 | 9.3 | 7 | 0.954 | 3.94 | -1.12 | 22.1 |
|  |  | **17b** | **13** | 5.17 |  |  |  |  |  |  |  |  |  |  |
|  |  | **4** | **17a** | 5.17 | 5 | 0.979 | 4.77 | -0.40 | 7.7 | 5 | 0.979 | 3.67 | -1.50 | 29.0 |
|  |  | **22** | **13** | 5.19 | 4 | 0.964 | 5.63 | 0.44 | 8.5 | 4 | 0.999 | 4.67 | -0.52 | 10.0 |
|  |  | **1** | **18b** | 5.32 |  |  |  |  |  |  |  |  |  |  |
|  |  | **20a** | **17a** | 5.36 |  |  |  |  |  |  |  |  |  |  |
|  |  | **12** | **18b** | 5.38 |  |  |  |  |  |  |  |  |  |  |
|  |  | **2** | **15b** | 5.44 |  |  |  |  |  |  |  |  |  |  |
| **Beyond the NOE limit** | **5.50-5.99** | **22** | **15b** | 5.56 |  |  |  |  |  |  |  |  |  |  |
|  |  | **4** | **17b** | 5.71 | 5 | 0.977 | 5.67 | -0.04 | 0.7 |  |  |  |  |  |
|  |  | **22** | **16** | 5.71 | 5 | 0.972 | 5.66 | -0.05 | 0.9 | 7 | 0.973 | 4.32 | -1.39 | 24.3 |
|  |  | **1** | **20a** | 5.74 | 5 | 0.960 | 4.61 | -1.13 | 19.7 |  |  |  |  |  |
|  |  | **18a** | **13** | 5.89 | 6 | 0.973 | 5.08 | -0.81 | 13.8 | 6 | 0.973 | 3.90 | -1.99 | 33.8 |
|  |  | **3** | **13** | 5.90 | 4 | 0.982 | 5.65 | -0.25 | 4.2 |  |  |  |  |  |
|  |  | **3** | **17b** | 5.95 |  |  |  |  |  |  |  |  |  |  |
|  | **6.00-6.49** | **4** | **16** | 6.07 | 4 | 0.997 | 6.19 | 0.12 | 2.0 | 5 | 0.952 | 4.82 | -1.25 | 20.6 |
|  |  | **3** | **17a** | 6.09 |  |  |  |  |  |  |  |  |  |  |
|  |  | **3** | **15b** | 6.11 |  |  |  |  |  |  |  |  |  |  |
|  |  | **2** | **13** | 6.11 | 4 | 0.976 | 6.19 | 0.08 | 1.3 |  |  |  |  |  |
|  |  | **22** | **11a** | 6.14 |  |  |  |  |  |  |  |  |  |  |
|  |  | **3** | **16** | 6.16 | 6 | 0.962 | 4.95 | -1.21 | 19.6 | 4 | 0.982 | 4.28 | -1.88 | 30.5 |
|  | **6.50-6.99** | **3** | **8** | 6.53 |  |  |  |  |  |  |  |  |  |  |
|  |  | **2** | **8** | 6.59 | 4 | 0.970 | 5.82 | -0.77 | 11.7 | 4 | 0.959 | 4.69 | -1.90 | 28.8 |
|  |  | **11a** | **15a** | 6.74 |  |  |  |  |  |  |  |  |  |  |
|  |  | **4** | **14** | 6.82 |  |  |  |  |  |  |  |  |  |  |
|  |  | **12** | **18a** | 6.94 |  |  |  |  |  |  |  |  |  |  |
|  | **7.00-7.49** | **2** | **11b/20b** | 7.43 |  |  |  |  |  |  |  |  |  |  |
|  | **7.50-7.99** | **2** | **15a** | 7.67 |  |  |  |  |  |  |  |  |  |  |
|  |  | **1** | **22** | 7.71 |  |  |  |  |  |  |  |  |  |  |
|  |  | **2** | **14** | 7.80 |  |  |  |  |  | 4 | 0.965 | 3.33 | -4.47 | 57.3 |
|  | **8.00-8.49** | **3** | **14** | 8.09 |  |  |  |  |  | 4 | 0.963 | 3.35 | -4.74 | 58.6 |
|  | **8.50-8.99** | **4** | **22** | 8.70 |  |  |  |  |  |  |  |  |  |  |
|  | **9.00-9.49** | **3** | **20a** | 9.19 |  |  |  |  |  |  |  |  |  |  |
|  | **9.50-9.99** | **2** | **22** | 9.82 |  |  |  |  |  |  |  |  |  |  |
|  |  |  |  |  | **# Distances** | |  |  |  | **# Distances** | |  |  |  |
| **Total # of distances** | | | | 127 | 56 |  |  |  |  | 53 |  |  |  |  |
| **# Underestimated** | | | |  | 39 |  |  |  |  | 49 |  |  |  |  |
| **# Overestimated** | | | |  | 16 |  |  |  |  | 2 |  |  |  |  |

**Table S19**. Quantified distances for the noesydbphw5 pulse program from the matrix generated from the diagonal integrals directly (NOESY W5) or after which the cross-peaks were adjusted to prevent the inclusion of noise (NOESY W5 adjusted cross-peaks). The theoretical population-averaged interproton distances as determined by Butts *et. al.*^[3]^ are given for each correlation, and used to calculate the error (Exp. – Theor.) as well as the absolute error (|%|). The number of data-points included (# t_mix_) for the distance determination, as well as the coefficient of determination (R^2^) of the linear regression are given. At the bottom of the table, the total number of quantified correlations as well as the number of over- and underestimated correlations are given. The proton-proton correlation used as a reference for distance calculation is being underscored.

|  |  |  |  |  | **NOESY W5** | | | | | **NOESY W5 adjusted cross-peaks** | | | | |
| --- | --- | --- | --- | --- | --- | --- | --- | --- | --- | --- | --- | --- | --- | --- |
|  |  | **H_a_** | **H_b_** | **r_ab_**  **Theor. (Å)** | **# t_mix_** | **R^2^** | **r_ab_ (Å)** | **Exp.**  **-**  **Theor. (Å)** | **\|%\|** | **# t_mix_** | **R^2^** | **r_ab_ (Å)** | **Exp.**  **-**  **Theor. (Å)** | **\|%\|** |
| **Short reliable** | **0.00-1.99** | **20a** | **11b/20b** | 1.76 | 5 | 0.969 | 1.97 | 0.21 | 11.9 |  |  |  |  |  |
|  |  | **15a** | **15b** | 1.76 | 7 | 0.998 | 1.76 | 0.00 | 0.0 | 7 | 0.998 | 1.76 | 0.00 | 0.0 |
|  |  | **11a** | **11b/20b** | 1.76 | 7 | 0.959 | 1.91 | 0.15 | 8.5 | 6 | 0.989 | 2.44 | 0.68 | 38.6 |
|  |  | **17a** | **17b** | 1.77 | 5 | 0.981 | 1.86 | 0.09 | 5.1 | 5 | 0.981 | 1.86 | 0.09 | 5.1 |
|  |  | **18a** | **18b** | 1.77 | 7 | 0.999 | 1.78 | 0.01 | 0.6 | 7 | 0.992 | 1.98 | 0.21 | 11.9 |
|  | **2.00-2.49** | **20a** | **15a** | 2.24 | 7 | 1.000 | 2.18 | -0.06 | 2.7 | 7 | 1.000 | 2.19 | -0.05 | 2.2 |
|  |  | **15b** | **13** | 2.26 |  |  |  |  |  | 7 | 0.976 | 2.80 | 0.54 | 23.9 |
|  |  | **12** | **23ab** | 2.27 | 7 | 1.000 | 2.30 | 0.03 | 1.3 | 7 | 1.000 | 2.34 | 0.07 | 3.1 |
|  |  | **22** | **23ab** | 2.30 | 7 | 0.999 | 2.40 | 0.10 | 4.3 | 7 | 0.999 | 2.40 | 0.10 | 4.3 |
|  |  | **12** | **13** | 2.35 | 7 | 1.000 | 2.32 | -0.03 | 1.3 | 7 | 1.000 | 2.44 | 0.09 | 3.8 |
|  |  | **12** | **11a** | 2.35 | 7 | 0.997 | 2.36 | 0.01 | 0.4 | 7 | 0.996 | 2.50 | 0.15 | 6.4 |
|  |  | **8** | **18b** | 2.37 | 7 | 1.000 | 2.24 | -0.13 | 5.5 | 7 | 1.000 | 2.25 | -0.12 | 5.1 |
|  |  | **1** | **16** | 2.39 | 7 | 1.000 | 2.28 | -0.11 | 4.6 | 7 | 1.000 | 2.30 | -0.09 | 3.8 |
|  |  | **22** | **11b/20b** | 2.39 | 7 | 0.985 | 2.58 | 0.19 | 7.9 | 7 | 0.999 | 2.62 | 0.23 | 9.6 |
|  |  | **14** | **13** | 2.41 | 7 | 1.000 | 2.39 | -0.02 | 0.8 | 7 | 0.999 | 2.47 | 0.06 | 2.5 |
|  |  | **8** | **17a** | 2.42 | 7 | 1.000 | 2.36 | -0.06 | 2.5 | 7 | 1.000 | 2.36 | -0.06 | 2.5 |
|  |  | **18a** | **17b** | 2.43 | 7 | 0.989 | 2.45 | 0.02 | 0.8 | 7 | 0.989 | 2.45 | 0.02 | 0.8 |
|  |  | **18b** | **17a** | 2.43 | 7 | 0.999 | 2.44 | 0.01 | 0.4 | 5 | 0.974 | 2.76 | 0.33 | 13.6 |
|  |  | **18b** | **11b/20b** | 2.45 | 5 | 0.962 | 2.68 | 0.23 | 9.4 | 6 | 0.988 | 3.14 | 0.69 | 28.2 |
|  |  | **16** | **15a** | 2.48 | 7 | 0.984 | 2.47 | -0.01 | 0.4 | 6 | 0.957 | 2.67 | 0.19 | 7.7 |
|  |  | **16** | **15b** | 2.48 | 7 | 0.986 | 2.51 | 0.03 | 1.2 | 5 | 0.967 | 2.80 | 0.32 | 12.9 |
|  |  | **3** | **2** | 2.48 |  |  |  |  |  |  |  |  |  |  |
|  |  | **14** | **15a** | 2.49 | 7 | 0.997 | 2.48 | -0.01 | 0.4 | 7 | 0.997 | 2.48 | -0.01 | 0.4 |
|  |  | **1** | **2** | 2.49 |  |  |  |  |  |  |  |  |  |  |
|  | **2.50-2.99** | **4** | **3** | 2.50 |  |  |  |  |  |  |  |  |  |  |
|  |  | **14** | **15b** | 2.55 | 7 | 0.996 | 2.62 | 0.07 | 2.7 | 6 | 0.957 | 3.03 | 0.48 | 18.8 |
|  |  | **23ab** | **14** | 2.59 | 7 | 1.000 | 2.56 | -0.03 | 1.2 | 7 | 1.000 | 2.65 | 0.06 | 2.3 |
|  |  | **8** | **11b/20b** | 2.63 | 7 | 0.998 | 2.66 | 0.03 | 1.1 | 7 | 0.999 | 2.71 | 0.08 | 3.0 |
|  |  | **16** | **17b** | 2.71 | 7 | 1.000 | 2.64 | -0.07 | 2.6 | 7 | 1.000 | 2.68 | -0.03 | 1.1 |
|  |  | **18a** | **17a** | 2.73 | 6 | 0.957 | 3.31 | 0.58 | 21.2 | 6 | 0.957 | 3.31 | 0.58 | 21.2 |
|  |  | **12** | **14** | 2.74 | 7 | 0.999 | 2.72 | -0.02 | 0.7 | 7 | 0.999 | 2.72 | -0.02 | 0.7 |
|  |  | **1** | **17b** | 2.79 | 7 | 0.999 | 2.73 | -0.06 | 2.2 | 7 | 0.999 | 2.79 | 0.00 | 0.0 |
|  |  | **18a** | **11b/20b** | 2.82 | 5 | 0.973 | 3.29 | 0.47 | 16.7 | 4 | 0.957 | 3.37 | 0.55 | 19.5 |
|  |  | **12** | **11b/20b** | 2.93 | 5 | 0.983 | 3.74 | 0.81 | 27.6 | 5 | 0.983 | 3.74 | 0.81 | 27.6 |
| **Long reliable** | **3.00-3.49** | **8** | **13** | 3.01 | 4 | 0.978 | 3.71 | 0.70 | 23.3 |  |  |  |  |  |
|  |  | **22** | **18b** | 3.16 | 7 | 1.000 | 2.99 | -0.17 | 5.4 | 7 | 0.999 | 3.02 | -0.14 | 4.4 |
|  |  | **23ab** | **11b/20b** | 3.24 | 7 | 0.950 | 3.65 | 0.41 | 12.7 | 7 | 0.957 | 3.73 | 0.49 | 15.1 |
|  |  | **20a** | **14** | 3.36 | 7 | 0.987 | 3.47 | 0.11 | 3.3 | 7 | 0.970 | 3.71 | 0.35 | 10.4 |
|  |  | **16** | **20a** | 3.37 | 6 | 0.959 | 3.68 | 0.31 | 9.2 | 7 | 0.973 | 4.13 | 0.76 | 22.6 |
|  |  | **11a** | **13** | 3.40 |  |  |  |  |  |  |  |  |  |  |
|  |  | **22** | **20a** | 3.42 |  |  |  |  |  |  |  |  |  |  |
|  | **3.50-3.99** | **15a** | **13** | 3.54 | 4 | 0.976 | 2.95 | -0.59 | 16.7 |  |  |  |  |  |
|  |  | **8** | **17b** | 3.61 |  |  |  |  |  |  |  |  |  |  |
|  |  | **11b/20b** | **13** | 3.61 | 7 | 0.970 | 4.06 | 0.45 | 12.5 | 7 | 0.966 | 4.20 | 0.59 | 16.3 |
|  |  | **16** | **18a** | 3.63 | 7 | 0.990 | 3.80 | 0.17 | 4.7 | 7 | 0.981 | 3.96 | 0.33 | 9.1 |
|  |  | **12** | **8** | 3.65 | 7 | 0.996 | 3.70 | 0.05 | 1.4 | 7 | 0.997 | 3.97 | 0.32 | 8.8 |
|  |  | **11b/20b** | **15a** | 3.69 | 6 | 0.976 | 3.05 | -0.64 | 17.3 | 6 | 0.979 | 3.16 | -0.53 | 14.4 |
|  |  | **23ab** | **8** | 3.73 | 4 | 0.953 | 3.87 | 0.14 | 3.8 | 4 | 0.953 | 3.87 | 0.14 | 3.8 |
|  |  | **20a** | **15b** | 3.75 | 6 | 0.972 | 2.87 | -0.88 | 23.5 | 4 | 0.964 | 3.56 | -0.19 | 5.1 |
|  |  | **23ab** | **13** | 3.76 |  |  |  |  |  |  |  |  |  |  |
|  |  | **8** | **18a** | 3.83 | 5 | 0.993 | 3.03 | -0.80 | 20.9 |  |  |  |  |  |
|  |  | **16** | **17a** | 3.84 |  |  |  |  |  | 4 | 0.963 | 4.99 | 1.15 | 29.9 |
|  |  | **1** | **15b** | 3.86 |  |  |  |  |  | 4 | 0.976 | 4.46 | 0.60 | 15.5 |
|  |  | **22** | **14** | 3.86 | 6 | 0.995 | 3.96 | 0.10 | 2.6 | 6 | 0.997 | 4.27 | 0.41 | 10.6 |
|  |  | **8** | **11a** | 3.88 |  |  |  |  |  |  |  |  |  |  |
|  |  | **16** | **18b** | 3.89 | 5 | 0.982 | 4.04 | 0.15 | 3.9 | 7 | 0.966 | 4.16 | 0.27 | 6.9 |
|  |  | **23ab** | **11a** | 3.96 |  |  |  |  |  |  |  |  |  |  |
|  |  | **14** | **11b/20b** | 3.96 |  |  |  |  |  |  |  |  |  |  |
|  |  | **8** | **14** | 3.96 | 7 | 0.987 | 3.92 | -0.04 | 1.0 | 5 | 0.960 | 4.47 | 0.51 | 12.9 |
|  |  | **22** | **8** | 3.96 | 7 | 0.997 | 3.87 | -0.09 | 2.3 | 7 | 0.993 | 4.09 | 0.13 | 3.3 |
|  | **4.00-4.49** | **1** | **17a** | 4.06 |  |  |  |  |  |  |  |  |  |  |
|  |  | **16** | **11b/20b** | 4.07 | 4 | 0.988 | 5.27 | 1.20 | 29.5 | 5 | 0.966 | 5.49 | 1.42 | 34.9 |
|  |  | **8** | **15b** | 4.09 |  |  |  |  |  |  |  |  |  |  |
|  |  | **16** | **8** | 4.14 |  |  |  |  |  |  |  |  |  |  |
|  |  | **11b/20b** | **17a** | 4.17 |  |  |  |  |  |  |  |  |  |  |
|  |  | **23ab** | **18b** | 4.27 | 4 | 0.954 | 4.88 | 0.61 | 14.3 |  |  |  |  |  |
|  |  | **12** | **15b** | 4.27 |  |  |  |  |  | 4 | 0.990 | 4.52 | 0.25 | 5.9 |
|  |  | **4** | **13** | 4.43 | 7 | 0.999 | 4.32 | -0.11 | 2.5 | 7 | 0.996 | 4.52 | 0.09 | 2.0 |
|  |  | **22** | **18a** | 4.47 | 4 | 0.988 | 5.33 | 0.86 | 19.2 |  |  |  |  |  |
| **At the NOE limit** | **4.50-4.99** | **18b** | **15a** | 4.55 |  |  |  |  |  |  |  |  |  |  |
|  |  | **11b/20b** | **15b** | 4.59 | 4 | 0.972 | 5.16 | 0.57 | 12.4 |  |  |  |  |  |
|  |  | **1** | **15a** | 4.62 | 4 | 0.991 | 4.58 | -0.04 | 0.9 |  |  |  |  |  |
|  |  | **8** | **15a** | 4.63 |  |  |  |  |  |  |  |  |  |  |
|  |  | **17b** | **15b** | 4.64 |  |  |  |  |  |  |  |  |  |  |
|  |  | **22** | **12** | 4.65 |  |  |  |  |  |  |  |  |  |  |
|  |  | **14** | **18b** | 4.66 |  |  |  |  |  |  |  |  |  |  |
|  |  | **23ab** | **15a** | 4.68 |  |  |  |  |  |  |  |  |  |  |
|  |  | **8** | **20a** | 4.71 |  |  |  |  |  |  |  |  |  |  |
|  |  | **18b** | **13** | 4.73 |  |  |  |  |  |  |  |  |  |  |
|  |  | **4** | **11a** | 4.77 | 6 | 0.992 | 4.68 | -0.09 | 1.9 | 7 | 0.959 | 5.05 | 0.28 | 5.9 |
|  |  | **23ab** | **15b** | 4.81 |  |  |  |  |  |  |  |  |  |  |
|  |  | **22** | **15a** | 4.81 | 5 | 0.975 | 5.00 | 0.19 | 4.0 | 4 | 0.988 | 5.39 | 0.58 | 12.1 |
|  |  | **2** | **17b** | 4.82 |  |  |  |  |  | 5 | 0.968 | 5.85 | 1.03 | 21.4 |
|  |  | **14** | **11a** | 4.87 |  |  |  |  |  |  |  |  |  |  |
|  |  | **20a** | **13** | 4.89 | 5 | 0.964 | 5.15 | 0.26 | 5.3 | 4 | 0.976 | 6.34 | 1.45 | 29.7 |
|  |  | **18a** | **15a** | 4.89 |  |  |  |  |  |  |  |  |  |  |
|  |  | **4** | **8** | 4.89 | 7 | 0.957 | 4.78 | -0.11 | 2.2 | 7 | 0.985 | 5.11 | 0.22 | 4.5 |
|  |  | **4** | **11b/20b** | 4.90 | 5 | 0.986 | 5.26 | 0.36 | 7.3 | 6 | 0.962 | 6.44 | 1.54 | 31.4 |
|  |  | **1** | **18a** | 4.90 |  |  |  |  |  |  |  |  |  |  |
|  |  | **20a** | **17b** | 4.90 |  |  |  |  |  |  |  |  |  |  |
|  |  | **12** | **15a** | 4.98 |  |  |  |  |  |  |  |  |  |  |
|  |  | **1** | **13** | 4.99 | 7 | 0.968 | 5.05 | 0.06 | 1.2 | 6 | 0.983 | 5.44 | 0.45 | 9.0 |
|  | **5.00-5.49** | **4** | **1** | 5.00 |  |  |  |  |  |  |  |  |  |  |
|  |  | **1** | **8** | 5.06 | 4 | 0.988 | 5.14 | 0.08 | 1.6 | 6 | 0.973 | 5.16 | 0.10 | 2.0 |
|  |  | **17b** | **13** | 5.17 |  |  |  |  |  | 5 | 0.954 | 4.45 | -0.72 | 13.9 |
|  |  | **4** | **17a** | 5.17 | 6 | 0.961 | 4.98 | -0.19 | 3.7 |  |  |  |  |  |
|  |  | **22** | **13** | 5.19 | 4 | 1.000 | 5.14 | -0.05 | 1.0 | 5 | 0.977 | 5.29 | 0.10 | 1.9 |
|  |  | **1** | **18b** | 5.32 |  |  |  |  |  | 4 | 0.969 | 6.81 | 1.49 | 28.0 |
|  |  | **20a** | **17a** | 5.36 |  |  |  |  |  |  |  |  |  |  |
|  |  | **12** | **18b** | 5.38 | 4 | 0.982 | 4.74 | -0.64 | 11.9 | 4 | 0.982 | 4.74 | -0.64 | 11.9 |
|  |  | **2** | **15b** | 5.44 | 5 | 0.963 | 5.59 | 0.15 | 2.8 |  |  |  |  |  |
| **Beyond the NOE limit** | **5.50-5.99** | **22** | **15b** | 5.56 |  |  |  |  |  |  |  |  |  |  |
|  |  | **4** | **17b** | 5.71 |  |  |  |  |  |  |  |  |  |  |
|  |  | **22** | **16** | 5.71 |  |  |  |  |  |  |  |  |  |  |
|  |  | **1** | **20a** | 5.74 | 5 | 0.968 | 5.41 | -0.33 | 5.7 |  |  |  |  |  |
|  |  | **18a** | **13** | 5.89 |  |  |  |  |  |  |  |  |  |  |
|  |  | **3** | **13** | 5.90 | 5 | 0.997 | 5.27 | -0.63 | 10.7 | 5 | 0.997 | 5.27 | -0.63 | 10.7 |
|  |  | **3** | **17b** | 5.95 |  |  |  |  |  | 4 | 0.977 | 5.82 | -0.13 | 2.2 |
|  | **6.00-6.49** | **4** | **16** | 6.07 |  |  |  |  |  |  |  |  |  |  |
|  |  | **3** | **17a** | 6.09 |  |  |  |  |  |  |  |  |  |  |
|  |  | **3** | **15b** | 6.11 | 4 | 0.951 | 5.83 | -0.28 | 4.6 |  |  |  |  |  |
|  |  | **2** | **13** | 6.11 |  |  |  |  |  |  |  |  |  |  |
|  |  | **22** | **11a** | 6.14 | 4 | 0.978 | 7.21 | 1.07 | 17.4 | 4 | 0.985 | 5.94 | -0.20 | 3.3 |
|  |  | **3** | **16** | 6.16 |  |  |  |  |  |  |  |  |  |  |
|  | **6.50-6.99** | **3** | **8** | 6.53 | 4 | 0.988 | 6.10 | -0.43 | 6.6 | 4 | 0.965 | 6.24 | -0.29 | 4.4 |
|  |  | **2** | **8** | 6.59 |  |  |  |  |  |  |  |  |  |  |
|  |  | **11a** | **15a** | 6.74 |  |  |  |  |  |  |  |  |  |  |
|  |  | **4** | **14** | 6.82 | 4 | 0.960 | 6.79 | -0.03 | 0.4 | 4 | 0.981 | 6.88 | 0.06 | 0.9 |
|  |  | **12** | **18a** | 6.94 | 4 | 0.996 | 4.16 | -2.78 | 40.1 | 4 | 0.996 | 4.20 | -2.74 | 39.5 |
|  | **7.00-7.49** | **2** | **11b/20b** | 7.43 | 4 | 0.985 | 7.78 | 0.35 | 4.7 |  |  |  |  |  |
|  | **7.50-7.99** | **2** | **15a** | 7.67 |  |  |  |  |  |  |  |  |  |  |
|  |  | **1** | **22** | 7.71 |  |  |  |  |  |  |  |  |  |  |
|  |  | **2** | **14** | 7.80 |  |  |  |  |  |  |  |  |  |  |
|  | **8.00-8.49** | **3** | **14** | 8.09 |  |  |  |  |  |  |  |  |  |  |
|  | **8.50-8.99** | **4** | **22** | 8.70 |  |  |  |  |  |  |  |  |  |  |
|  | **9.00-9.49** | **3** | **20a** | 9.19 |  |  |  |  |  |  |  |  |  |  |
|  | **9.50-9.99** | **2** | **22** | 9.82 |  |  |  |  |  |  |  |  |  |  |
|  |  |  |  |  | **# Distances** | |  |  |  | **# Distances** | |  |  |  |
| **Total # of distances** | | | | 127 | 72 |  |  |  |  | 67 |  |  |  |  |
| **# Underestimated** | | | |  | 32 |  |  |  |  | 17 |  |  |  |  |
| **# Overestimated** | | | |  | 39 |  |  |  |  | 48 |  |  |  |  |

**Table S20**. Quantified distances for the roesyph pulse program from the matrix generated from the diagonal integrals directly (ROESY) or after which the cross-peaks were adjusted to prevent the inclusion of noise (ROESY adjusted cross-peaks). The theoretical population-averaged interproton distances as determined by Butts *et. al.*^[3]^ are given for each correlation, and used to calculate the error (Exp. – Theor.) as well as the absolute error (|%|). The number of data-points included (# t_mix_) for the distance determination, as well as the coefficient of determination (R^2^) of the linear regression are given. At the bottom of the table, the total number of quantified correlations as well as the number of over- and underestimated correlations are given. The proton-proton correlation used as a reference for distance calculation is being underscored.

|  |  |  |  |  | **ROESY** | | | | | **ROESY adjusted cross-peaks** | | | | |
| --- | --- | --- | --- | --- | --- | --- | --- | --- | --- | --- | --- | --- | --- | --- |
|  |  | **H_a_** | **H_b_** | **r_ab_**  **Theor. (Å)** | **# t_mix_** | **R^2^** | **r_ab_ (Å)** | **Exp.**  **-**  **Theor. (Å)** | **\|%\|** | **# t_mix_** | **R^2^** | **r_ab_ (Å)** | **Exp.**  **-**  **Theor. (Å)** | **\|%\|** |
| **Short reliable** | **0.00-1.99** | **20a** | **11b/20b** | 1.76 | 7 | 0.970 | 1.78 | 0.02 | 1.1 | 5 | 1.000 | 2.25 | 0.49 | 27.8 |
|  |  | **15a** | **15b** | 1.76 | 7 | 0.951 | 1.76 | 0.00 | 0.0 | 7 | 0.951 | 1.76 | 0.00 | 0.0 |
|  |  | **11a** | **11b/20b** | 1.76 | 7 | 0.992 | 1.90 | 0.14 | 8.0 | 7 | 0.992 | 1.90 | 0.14 | 8.0 |
|  |  | **17a** | **17b** | 1.77 | 7 | 0.977 | 1.80 | 0.03 | 1.7 | 4 | 0.984 | 2.65 | 0.88 | 49.7 |
|  |  | **18a** | **18b** | 1.77 | 7 | 0.997 | 1.73 | -0.04 | 2.3 | 7 | 0.997 | 1.73 | -0.04 | 2.3 |
|  | **2.00-2.49** | **20a** | **15a** | 2.24 | 7 | 1.000 | 2.09 | -0.15 | 6.7 | 7 | 1.000 | 2.14 | -0.10 | 4.5 |
|  |  | **15b** | **13** | 2.26 | 4 | 0.960 | 2.22 | -0.04 | 1.8 | 6 | 0.991 | 2.20 | -0.06 | 2.7 |
|  |  | **12** | **23ab** | 2.27 | 7 | 1.000 | 2.18 | -0.09 | 4.0 | 7 | 1.000 | 2.22 | -0.05 | 2.2 |
|  |  | **22** | **23ab** | 2.30 | 7 | 0.999 | 2.41 | 0.11 | 4.8 | 7 | 0.999 | 2.41 | 0.11 | 4.8 |
|  |  | **12** | **13** | 2.35 | 7 | 1.000 | 2.37 | 0.02 | 0.9 | 7 | 1.000 | 2.52 | 0.17 | 7.2 |
|  |  | **12** | **11a** | 2.35 | 7 | 0.991 | 2.32 | -0.03 | 1.3 | 7 | 0.950 | 2.44 | 0.09 | 3.8 |
|  |  | **8** | **18b** | 2.37 | 7 | 1.000 | 2.18 | -0.19 | 8.0 | 7 | 1.000 | 2.20 | -0.17 | 7.2 |
|  |  | **1** | **16** | 2.39 | 7 | 1.000 | 2.36 | -0.03 | 1.3 | 7 | 1.000 | 2.41 | 0.02 | 0.8 |
|  |  | **22** | **11b/20b** | 2.39 | 7 | 0.999 | 2.66 | 0.27 | 11.3 | 7 | 0.999 | 2.71 | 0.32 | 13.4 |
|  |  | **14** | **13** | 2.41 | 7 | 1.000 | 2.35 | -0.06 | 2.5 | 7 | 1.000 | 2.35 | -0.06 | 2.5 |
|  |  | **8** | **17a** | 2.42 | 7 | 1.000 | 2.34 | -0.08 | 3.3 | 7 | 1.000 | 2.39 | -0.03 | 1.2 |
|  |  | **18a** | **17b** | 2.43 | 7 | 0.997 | 2.31 | -0.12 | 4.9 | 7 | 0.952 | 2.69 | 0.26 | 10.7 |
|  |  | **18b** | **17a** | 2.43 | 7 | 0.997 | 2.37 | -0.06 | 2.5 | 7 | 0.997 | 2.37 | -0.06 | 2.5 |
|  |  | **18b** | **11b/20b** | 2.45 | 7 | 0.951 | 2.48 | 0.03 | 1.2 | 6 | 0.975 | 2.82 | 0.37 | 15.1 |
|  |  | **16** | **15a** | 2.48 | 7 | 1.000 | 2.42 | -0.06 | 2.4 | 7 | 1.000 | 2.42 | -0.06 | 2.4 |
|  |  | **16** | **15b** | 2.48 | 7 | 0.994 | 2.47 | -0.01 | 0.4 | 7 | 0.996 | 2.65 | 0.17 | 6.9 |
|  |  | **3** | **2** | 2.48 | 4 | 0.986 | 2.54 | 0.06 | 2.4 | 4 | 0.986 | 2.54 | 0.06 | 2.4 |
|  |  | **14** | **15a** | 2.49 | 7 | 0.996 | 2.47 | -0.02 | 0.8 | 7 | 0.996 | 2.47 | -0.02 | 0.8 |
|  |  | **1** | **2** | 2.49 |  |  |  |  |  |  |  |  |  |  |
|  | **2.50-2.99** | **4** | **3** | 2.50 | 5 | 0.953 | 2.73 | 0.23 | 9.2 | 5 | 0.953 | 2.73 | 0.23 | 9.2 |
|  |  | **14** | **15b** | 2.55 | 7 | 0.989 | 2.50 | -0.05 | 2.0 | 7 | 0.985 | 2.60 | 0.05 | 2.0 |
|  |  | **23ab** | **14** | 2.59 | 7 | 1.000 | 2.46 | -0.13 | 5.0 | 7 | 0.993 | 2.85 | 0.26 | 10.0 |
|  |  | **8** | **11b/20b** | 2.63 | 7 | 0.995 | 2.67 | 0.04 | 1.5 | 7 | 0.992 | 2.73 | 0.10 | 3.8 |
|  |  | **16** | **17b** | 2.71 | 7 | 1.000 | 2.60 | -0.11 | 4.1 | 7 | 1.000 | 2.63 | -0.08 | 3.0 |
|  |  | **18a** | **17a** | 2.73 | 7 | 0.975 | 2.92 | 0.19 | 7.0 | 7 | 0.975 | 3.04 | 0.31 | 11.4 |
|  |  | **12** | **14** | 2.74 | 7 | 1.000 | 2.65 | -0.09 | 3.3 | 7 | 1.000 | 2.74 | 0.00 | 0.0 |
|  |  | **1** | **17b** | 2.79 | 6 | 0.980 | 3.94 | 1.15 | 41.2 | 6 | 0.980 | 3.94 | 1.15 | 41.2 |
|  |  | **18a** | **11b/20b** | 2.82 | 7 | 0.959 | 2.90 | 0.08 | 2.8 | 5 | 0.961 | 3.29 | 0.47 | 16.7 |
|  |  | **12** | **11b/20b** | 2.93 | 6 | 0.955 | 3.21 | 0.28 | 9.6 |  |  |  |  |  |
| **Long reliable** | **3.00-3.49** | **8** | **13** | 3.01 | 6 | 0.992 | 2.94 | -0.07 | 2.3 | 6 | 0.992 | 2.94 | -0.07 | 2.3 |
|  |  | **22** | **18b** | 3.16 | 7 | 0.997 | 3.11 | -0.05 | 1.6 | 7 | 0.997 | 3.23 | 0.07 | 2.2 |
|  |  | **23ab** | **11b/20b** | 3.24 | 7 | 0.969 | 3.48 | 0.24 | 7.4 | 7 | 0.966 | 3.88 | 0.64 | 19.8 |
|  |  | **20a** | **14** | 3.36 | 6 | 0.993 | 3.38 | 0.02 | 0.6 | 6 | 0.993 | 3.38 | 0.02 | 0.6 |
|  |  | **16** | **20a** | 3.37 | 6 | 0.967 | 3.61 | 0.24 | 7.1 | 6 | 0.967 | 3.61 | 0.24 | 7.1 |
|  |  | **11a** | **13** | 3.40 | 7 | 0.999 | 3.48 | 0.08 | 2.4 | 7 | 0.999 | 3.62 | 0.22 | 6.5 |
|  |  | **22** | **20a** | 3.42 | 5 | 0.959 | 4.38 | 0.96 | 28.1 | 5 | 0.958 | 4.28 | 0.86 | 25.1 |
|  | **3.50-3.99** | **15a** | **13** | 3.54 |  |  |  |  |  |  |  |  |  |  |
|  |  | **8** | **17b** | 3.61 | 4 | 0.985 | 4.71 | 1.10 | 30.5 |  |  |  |  |  |
|  |  | **11b/20b** | **13** | 3.61 | 5 | 0.970 | 4.10 | 0.49 | 13.6 | 6 | 0.967 | 5.06 | 1.45 | 40.2 |
|  |  | **16** | **18a** | 3.63 | 7 | 0.957 | 3.61 | -0.02 | 0.6 | 7 | 0.955 | 3.74 | 0.11 | 3.0 |
|  |  | **12** | **8** | 3.65 | 7 | 0.978 | 3.46 | -0.19 | 5.2 | 7 | 0.978 | 3.46 | -0.19 | 5.2 |
|  |  | **11b/20b** | **15a** | 3.69 | 6 | 0.954 | 3.08 | -0.61 | 16.5 | 6 | 0.954 | 3.08 | -0.61 | 16.5 |
|  |  | **23ab** | **8** | 3.73 | 4 | 0.999 | 3.64 | -0.09 | 2.4 | 4 | 0.999 | 3.64 | -0.09 | 2.4 |
|  |  | **20a** | **15b** | 3.75 | 5 | 0.984 | 2.89 | -0.86 | 22.9 | 5 | 0.978 | 3.20 | -0.55 | 14.7 |
|  |  | **23ab** | **13** | 3.76 |  |  |  |  |  |  |  |  |  |  |
|  |  | **8** | **18a** | 3.83 | 5 | 0.981 | 3.16 | -0.67 | 17.5 | 5 | 0.975 | 3.17 | -0.66 | 17.2 |
|  |  | **16** | **17a** | 3.84 |  |  |  |  |  |  |  |  |  |  |
|  |  | **1** | **15b** | 3.86 |  |  |  |  |  |  |  |  |  |  |
|  |  | **22** | **14** | 3.86 |  |  |  |  |  |  |  |  |  |  |
|  |  | **8** | **11a** | 3.88 |  |  |  |  |  |  |  |  |  |  |
|  |  | **16** | **18b** | 3.89 | 6 | 0.953 | 3.91 | 0.02 | 0.5 | 5 | 0.955 | 4.65 | 0.76 | 19.5 |
|  |  | **23ab** | **11a** | 3.96 |  |  |  |  |  |  |  |  |  |  |
|  |  | **14** | **11b/20b** | 3.96 |  |  |  |  |  |  |  |  |  |  |
|  |  | **8** | **14** | 3.96 | 6 | 0.952 | 3.85 | -0.11 | 2.8 | 6 | 0.952 | 3.85 | -0.11 | 2.8 |
|  |  | **22** | **8** | 3.96 | 7 | 0.980 | 3.80 | -0.16 | 4.0 | 7 | 0.973 | 3.93 | -0.03 | 0.8 |
|  | **4.00-4.49** | **1** | **17a** | 4.06 |  |  |  |  |  |  |  |  |  |  |
|  |  | **16** | **11b/20b** | 4.07 |  |  |  |  |  |  |  |  |  |  |
|  |  | **8** | **15b** | 4.09 |  |  |  |  |  |  |  |  |  |  |
|  |  | **16** | **8** | 4.14 | 7 | 0.959 | 2.57 | -1.57 | 37.9 | 7 | 0.959 | 2.57 | -1.57 | 37.9 |
|  |  | **11b/20b** | **17a** | 4.17 |  |  |  |  |  |  |  |  |  |  |
|  |  | **23ab** | **18b** | 4.27 |  |  |  |  |  |  |  |  |  |  |
|  |  | **12** | **15b** | 4.27 |  |  |  |  |  |  |  |  |  |  |
|  |  | **4** | **13** | 4.43 |  |  |  |  |  |  |  |  |  |  |
|  |  | **22** | **18a** | 4.47 |  |  |  |  |  |  |  |  |  |  |
| **At the NOE limit** | **4.50-4.99** | **18b** | **15a** | 4.55 | 4 | 0.956 | 5.32 | 0.77 | 16.9 | 4 | 0.988 | 5.23 | 0.68 | 14.9 |
|  |  | **11b/20b** | **15b** | 4.59 |  |  |  |  |  |  |  |  |  |  |
|  |  | **1** | **15a** | 4.62 |  |  |  |  |  |  |  |  |  |  |
|  |  | **8** | **15a** | 4.63 | 4 | 0.997 | 3.98 | -0.65 | 14.0 | 4 | 0.997 | 3.98 | -0.65 | 14.0 |
|  |  | **17b** | **15b** | 4.64 |  |  |  |  |  |  |  |  |  |  |
|  |  | **22** | **12** | 4.65 | 5 | 0.954 | 5.01 | 0.36 | 7.7 | 4 | 0.994 | 5.43 | 0.78 | 16.8 |
|  |  | **14** | **18b** | 4.66 |  |  |  |  |  |  |  |  |  |  |
|  |  | **23ab** | **15a** | 4.68 | 4 | 0.965 | 5.13 | 0.45 | 9.6 | 4 | 0.965 | 5.13 | 0.45 | 9.6 |
|  |  | **8** | **20a** | 4.71 |  |  |  |  |  |  |  |  |  |  |
|  |  | **18b** | **13** | 4.73 |  |  |  |  |  |  |  |  |  |  |
|  |  | **4** | **11a** | 4.77 |  |  |  |  |  |  |  |  |  |  |
|  |  | **23ab** | **15b** | 4.81 |  |  |  |  |  |  |  |  |  |  |
|  |  | **22** | **15a** | 4.81 | 5 | 0.985 | 4.43 | -0.38 | 7.9 |  |  |  |  |  |
|  |  | **2** | **17b** | 4.82 |  |  |  |  |  |  |  |  |  |  |
|  |  | **14** | **11a** | 4.87 |  |  |  |  |  |  |  |  |  |  |
|  |  | **20a** | **13** | 4.89 |  |  |  |  |  |  |  |  |  |  |
|  |  | **18a** | **15a** | 4.89 |  |  |  |  |  |  |  |  |  |  |
|  |  | **4** | **8** | 4.89 |  |  |  |  |  |  |  |  |  |  |
|  |  | **4** | **11b/20b** | 4.90 |  |  |  |  |  |  |  |  |  |  |
|  |  | **1** | **18a** | 4.90 |  |  |  |  |  |  |  |  |  |  |
|  |  | **20a** | **17b** | 4.90 | 5 | 0.967 | 4.94 | 0.04 | 0.8 | 5 | 0.967 | 4.94 | 0.04 | 0.8 |
|  |  | **12** | **15a** | 4.98 |  |  |  |  |  |  |  |  |  |  |
|  |  | **1** | **13** | 4.99 |  |  |  |  |  |  |  |  |  |  |
|  | **5.00-5.49** | **4** | **1** | 5.00 |  |  |  |  |  | 4 | 0.961 | 5.14 | 0.14 | 2.8 |
|  |  | **1** | **8** | 5.06 | 5 | 0.952 | 4.35 | -0.71 | 14.0 | 5 | 0.967 | 5.08 | 0.02 | 0.4 |
|  |  | **17b** | **13** | 5.17 |  |  |  |  |  |  |  |  |  |  |
|  |  | **4** | **17a** | 5.17 |  |  |  |  |  |  |  |  |  |  |
|  |  | **22** | **13** | 5.19 | 5 | 0.951 | 4.29 | -0.90 | 17.3 | 4 | 0.962 | 4.79 | -0.40 | 7.7 |
|  |  | **1** | **18b** | 5.32 |  |  |  |  |  |  |  |  |  |  |
|  |  | **20a** | **17a** | 5.36 |  |  |  |  |  |  |  |  |  |  |
|  |  | **12** | **18b** | 5.38 |  |  |  |  |  |  |  |  |  |  |
|  |  | **2** | **15b** | 5.44 |  |  |  |  |  |  |  |  |  |  |
| **Beyond the NOE limit** | **5.50-5.99** | **22** | **15b** | 5.56 |  |  |  |  |  | 5 | 0.953 | 6.15 | 0.59 | 10.6 |
|  |  | **4** | **17b** | 5.71 |  |  |  |  |  |  |  |  |  |  |
|  |  | **22** | **16** | 5.71 |  |  |  |  |  |  |  |  |  |  |
|  |  | **1** | **20a** | 5.74 |  |  |  |  |  |  |  |  |  |  |
|  |  | **18a** | **13** | 5.89 |  |  |  |  |  |  |  |  |  |  |
|  |  | **3** | **13** | 5.90 |  |  |  |  |  |  |  |  |  |  |
|  |  | **3** | **17b** | 5.95 |  |  |  |  |  |  |  |  |  |  |
|  | **6.00-6.49** | **4** | **16** | 6.07 |  |  |  |  |  |  |  |  |  |  |
|  |  | **3** | **17a** | 6.09 |  |  |  |  |  |  |  |  |  |  |
|  |  | **3** | **15b** | 6.11 |  |  |  |  |  |  |  |  |  |  |
|  |  | **2** | **13** | 6.11 |  |  |  |  |  |  |  |  |  |  |
|  |  | **22** | **11a** | 6.14 |  |  |  |  |  |  |  |  |  |  |
|  |  | **3** | **16** | 6.16 |  |  |  |  |  |  |  |  |  |  |
|  | **6.50-6.99** | **3** | **8** | 6.53 |  |  |  |  |  |  |  |  |  |  |
|  |  | **2** | **8** | 6.59 |  |  |  |  |  |  |  |  |  |  |
|  |  | **11a** | **15a** | 6.74 |  |  |  |  |  |  |  |  |  |  |
|  |  | **4** | **14** | 6.82 |  |  |  |  |  |  |  |  |  |  |
|  |  | **12** | **18a** | 6.94 |  |  |  |  |  |  |  |  |  |  |
|  | **7.00-7.49** | **2** | **11b/20b** | 7.43 |  |  |  |  |  |  |  |  |  |  |
|  | **7.50-7.99** | **2** | **15a** | 7.67 |  |  |  |  |  |  |  |  |  |  |
|  |  | **1** | **22** | 7.71 | 5 | 0.961 | 6.03 | -1.68 | 21.8 |  |  |  |  |  |
|  |  | **2** | **14** | 7.80 |  |  |  |  |  |  |  |  |  |  |
|  | **8.00-8.49** | **3** | **14** | 8.09 |  |  |  |  |  |  |  |  |  |  |
|  | **8.50-8.99** | **4** | **22** | 8.70 |  |  |  |  |  |  |  |  |  |  |
|  | **9.00-9.49** | **3** | **20a** | 9.19 |  |  |  |  |  |  |  |  |  |  |
|  | **9.50-9.99** | **2** | **22** | 9.82 |  |  |  |  |  |  |  |  |  |  |
|  |  |  |  |  | **# Distances** | |  |  |  | **# Distances** | |  |  |  |
| **Total # of distances** | | | | 127 | 61 |  |  |  |  | 59 |  |  |  |  |
| **# Underestimated** | | | |  | 34 |  |  |  |  | 22 |  |  |  |  |
| **# Overestimated** | | | |  | 26 |  |  |  |  | 35 |  |  |  |  |

**Table S21**. Quantified distances for the EASY ROESY without solvent suppression roesyadesjsph pulse program from the matrix generated from the diagonal integrals directly (EASY ROESY) or after which the cross-peaks were adjusted to prevent the inclusion of noise (EASY ROESY adjusted cross-peaks). The theoretical population-averaged interproton distances as determined by Butts *et. al.*^[3]^ are given for each correlation, and used to calculate the error (Exp. – Theor.) as well as the absolute error (|%|). The number of data-points included (# t_mix_) for the distance determination, as well as the coefficient of determination (R^2^) of the linear regression are given. At the bottom of the table, the total number of quantified correlations as well as the number of over- and underestimated correlations are given. The proton-proton correlation used as a reference for distance calculation is being underscored.

|  |  |  |  |  | **EASY ROESY** | | | | | **EASY ROESY adjusted cross-peaks** | | | | |
| --- | --- | --- | --- | --- | --- | --- | --- | --- | --- | --- | --- | --- | --- | --- |
|  |  | **H_a_** | **H_b_** | **r_ab_**  **Theor. (Å)** | **# t_mix_** | **R^2^** | **r_ab_ (Å)** | **Exp.**  **-**  **Theor. (Å)** | **\|%\|** | **# t_mix_** | **R^2^** | **r_ab_ (Å)** | **Exp.**  **-**  **Theor. (Å)** | **\|%\|** |
| **Short reliable** | **0.00-1.99** | **20a** | **11b/20b** | 1.76 | 5 | 0.957 | 1.99 | 0.23 | 13.1 |  |  |  |  |  |
|  |  | **15a** | **15b** | 1.76 | 7 | 0.957 | 1.76 | 0.00 | 0.0 | 7 | 0.957 | 1.76 | 0.00 | 0.0 |
|  |  | **11a** | **11b/20b** | 1.76 |  |  |  |  |  |  |  |  |  |  |
|  |  | **17a** | **17b** | 1.77 |  |  |  |  |  | 6 | 0.967 | 2.39 | 0.62 | 35.0 |
|  |  | **18a** | **18b** | 1.77 | 7 | 0.973 | 1.90 | 0.13 | 7.3 | 7 | 0.973 | 1.90 | 0.13 | 7.3 |
|  | **2.00-2.49** | **20a** | **15a** | 2.24 | 7 | 0.998 | 2.22 | -0.02 | 0.9 | 7 | 0.998 | 2.22 | -0.02 | 0.9 |
|  |  | **15b** | **13** | 2.26 |  |  |  |  |  |  |  |  |  |  |
|  |  | **12** | **23ab** | 2.27 | 7 | 0.999 | 2.38 | 0.11 | 4.8 | 7 | 1.000 | 2.43 | 0.16 | 7.0 |
|  |  | **22** | **23ab** | 2.30 | 7 | 0.997 | 2.57 | 0.27 | 11.7 | 7 | 0.999 | 2.62 | 0.32 | 13.9 |
|  |  | **12** | **13** | 2.35 | 7 | 1.000 | 2.43 | 0.08 | 3.4 | 7 | 1.000 | 2.45 | 0.10 | 4.3 |
|  |  | **12** | **11a** | 2.35 | 7 | 0.996 | 2.40 | 0.05 | 2.1 | 5 | 0.968 | 2.62 | 0.27 | 11.5 |
|  |  | **8** | **18b** | 2.37 | 7 | 1.000 | 2.34 | -0.03 | 1.3 | 7 | 1.000 | 2.34 | -0.03 | 1.3 |
|  |  | **1** | **16** | 2.39 | 7 | 1.000 | 2.36 | -0.03 | 1.3 | 7 | 1.000 | 2.37 | -0.02 | 0.8 |
|  |  | **22** | **11b/20b** | 2.39 | 7 | 1.000 | 2.56 | 0.17 | 7.1 | 7 | 1.000 | 2.60 | 0.21 | 8.8 |
|  |  | **14** | **13** | 2.41 | 7 | 1.000 | 2.46 | 0.05 | 2.1 | 7 | 1.000 | 2.63 | 0.22 | 9.1 |
|  |  | **8** | **17a** | 2.42 | 7 | 0.999 | 2.48 | 0.06 | 2.5 | 7 | 0.999 | 2.48 | 0.06 | 2.5 |
|  |  | **18a** | **17b** | 2.43 | 6 | 0.975 | 2.57 | 0.14 | 5.8 |  |  |  |  |  |
|  |  | **18b** | **17a** | 2.43 |  |  |  |  |  |  |  |  |  |  |
|  |  | **18b** | **11b/20b** | 2.45 |  |  |  |  |  |  |  |  |  |  |
|  |  | **16** | **15a** | 2.48 | 7 | 0.985 | 2.53 | 0.05 | 2.0 | 7 | 0.988 | 2.90 | 0.42 | 16.9 |
|  |  | **16** | **15b** | 2.48 | 7 | 0.990 | 2.64 | 0.16 | 6.5 | 5 | 0.959 | 3.66 | 1.18 | 47.6 |
|  |  | **3** | **2** | 2.48 |  |  |  |  |  |  |  |  |  |  |
|  |  | **14** | **15a** | 2.49 | 7 | 0.956 | 2.66 | 0.17 | 6.8 | 6 | 0.993 | 2.64 | 0.15 | 6.0 |
|  |  | **1** | **2** | 2.49 |  |  |  |  |  |  |  |  |  |  |
|  | **2.50-2.99** | **4** | **3** | 2.50 |  |  |  |  |  |  |  |  |  |  |
|  |  | **14** | **15b** | 2.55 | 7 | 0.998 | 2.68 | 0.13 | 5.1 | 6 | 0.959 | 3.05 | 0.50 | 19.6 |
|  |  | **23ab** | **14** | 2.59 | 7 | 1.000 | 2.67 | 0.08 | 3.1 | 7 | 1.000 | 2.69 | 0.10 | 3.9 |
|  |  | **8** | **11b/20b** | 2.63 | 6 | 0.998 | 2.98 | 0.35 | 13.3 | 6 | 0.998 | 2.98 | 0.35 | 13.3 |
|  |  | **16** | **17b** | 2.71 | 7 | 1.000 | 2.72 | 0.01 | 0.4 | 7 | 1.000 | 2.73 | 0.02 | 0.7 |
|  |  | **18a** | **17a** | 2.73 | 5 | 0.961 | 3.03 | 0.30 | 11.0 | 5 | 0.969 | 3.28 | 0.55 | 20.1 |
|  |  | **12** | **14** | 2.74 | 7 | 0.999 | 2.83 | 0.09 | 3.3 | 7 | 0.999 | 2.89 | 0.15 | 5.5 |
|  |  | **1** | **17b** | 2.79 | 7 | 0.999 | 2.89 | 0.10 | 3.6 | 7 | 0.998 | 2.99 | 0.20 | 7.2 |
|  |  | **18a** | **11b/20b** | 2.82 | 5 | 0.984 | 2.91 | 0.09 | 3.2 | 5 | 0.973 | 3.03 | 0.21 | 7.4 |
|  |  | **12** | **11b/20b** | 2.93 |  |  |  |  |  |  |  |  |  |  |
| **Long reliable** | **3.00-3.49** | **8** | **13** | 3.01 |  |  |  |  |  |  |  |  |  |  |
|  |  | **22** | **18b** | 3.16 | 7 | 0.999 | 3.16 | 0.00 | 0.0 | 7 | 0.999 | 3.22 | 0.06 | 1.9 |
|  |  | **23ab** | **11b/20b** | 3.24 | 6 | 0.986 | 3.83 | 0.59 | 18.2 | 6 | 0.975 | 4.07 | 0.83 | 25.6 |
|  |  | **20a** | **14** | 3.36 | 4 | 0.959 | 3.31 | -0.05 | 1.5 | 5 | 0.972 | 3.92 | 0.56 | 16.7 |
|  |  | **16** | **20a** | 3.37 |  |  |  |  |  |  |  |  |  |  |
|  |  | **11a** | **13** | 3.40 | 7 | 0.979 | 3.60 | 0.20 | 5.9 | 7 | 0.968 | 4.13 | 0.73 | 21.5 |
|  |  | **22** | **20a** | 3.42 |  |  |  |  |  |  |  |  |  |  |
|  | **3.50-3.99** | **15a** | **13** | 3.54 |  |  |  |  |  |  |  |  |  |  |
|  |  | **8** | **17b** | 3.61 | 5 | 0.965 | 3.78 | 0.17 | 4.7 | 5 | 0.994 | 4.12 | 0.51 | 14.1 |
|  |  | **11b/20b** | **13** | 3.61 | 6 | 0.965 | 4.15 | 0.54 | 15.0 | 6 | 0.979 | 4.51 | 0.90 | 24.9 |
|  |  | **16** | **18a** | 3.63 |  |  |  |  |  |  |  |  |  |  |
|  |  | **12** | **8** | 3.65 |  |  |  |  |  |  |  |  |  |  |
|  |  | **11b/20b** | **15a** | 3.69 | 4 | 1.000 | 3.02 | -0.67 | 18.2 | 6 | 0.977 | 3.08 | -0.61 | 16.5 |
|  |  | **23ab** | **8** | 3.73 |  |  |  |  |  |  |  |  |  |  |
|  |  | **20a** | **15b** | 3.75 | 5 | 0.997 | 3.01 | -0.74 | 19.7 | 4 | 0.998 | 3.23 | -0.52 | 13.9 |
|  |  | **23ab** | **13** | 3.76 |  |  |  |  |  |  |  |  |  |  |
|  |  | **8** | **18a** | 3.83 |  |  |  |  |  |  |  |  |  |  |
|  |  | **16** | **17a** | 3.84 | 6 | 0.959 | 4.02 | 0.18 | 4.7 | 6 | 0.973 | 3.89 | 0.05 | 1.3 |
|  |  | **1** | **15b** | 3.86 | 5 | 0.980 | 4.21 | 0.35 | 9.1 | 4 | 0.992 | 4.66 | 0.80 | 20.7 |
|  |  | **22** | **14** | 3.86 | 6 | 0.994 | 4.06 | 0.20 | 5.2 | 6 | 0.987 | 4.47 | 0.61 | 15.8 |
|  |  | **8** | **11a** | 3.88 |  |  |  |  |  |  |  |  |  |  |
|  |  | **16** | **18b** | 3.89 |  |  |  |  |  |  |  |  |  |  |
|  |  | **23ab** | **11a** | 3.96 |  |  |  |  |  |  |  |  |  |  |
|  |  | **14** | **11b/20b** | 3.96 |  |  |  |  |  |  |  |  |  |  |
|  |  | **8** | **14** | 3.96 |  |  |  |  |  | 4 | 0.994 | 3.98 | 0.02 | 0.5 |
|  |  | **22** | **8** | 3.96 | 7 | 0.981 | 3.98 | 0.02 | 0.5 | 6 | 0.973 | 4.27 | 0.31 | 7.8 |
|  | **4.00-4.49** | **1** | **17a** | 4.06 | 4 | 0.999 | 4.13 | 0.07 | 1.7 | 7 | 0.976 | 4.11 | 0.05 | 1.2 |
|  |  | **16** | **11b/20b** | 4.07 |  |  |  |  |  |  |  |  |  |  |
|  |  | **8** | **15b** | 4.09 | 5 | 0.973 | 4.26 | 0.17 | 4.2 | 5 | 0.995 | 4.51 | 0.42 | 10.3 |
|  |  | **16** | **8** | 4.14 |  |  |  |  |  |  |  |  |  |  |
|  |  | **11b/20b** | **17a** | 4.17 |  |  |  |  |  |  |  |  |  |  |
|  |  | **23ab** | **18b** | 4.27 |  |  |  |  |  |  |  |  |  |  |
|  |  | **12** | **15b** | 4.27 |  |  |  |  |  |  |  |  |  |  |
|  |  | **4** | **13** | 4.43 | 4 | 0.965 | 4.82 | 0.39 | 8.8 |  |  |  |  |  |
|  |  | **22** | **18a** | 4.47 |  |  |  |  |  |  |  |  |  |  |
| **At the NOE limit** | **4.50-4.99** | **18b** | **15a** | 4.55 |  |  |  |  |  |  |  |  |  |  |
|  |  | **11b/20b** | **15b** | 4.59 |  |  |  |  |  |  |  |  |  |  |
|  |  | **1** | **15a** | 4.62 |  |  |  |  |  |  |  |  |  |  |
|  |  | **8** | **15a** | 4.63 |  |  |  |  |  | 4 | 0.963 | 5.64 | 1.01 | 21.8 |
|  |  | **17b** | **15b** | 4.64 |  |  |  |  |  |  |  |  |  |  |
|  |  | **22** | **12** | 4.65 |  |  |  |  |  |  |  |  |  |  |
|  |  | **14** | **18b** | 4.66 |  |  |  |  |  |  |  |  |  |  |
|  |  | **23ab** | **15a** | 4.68 |  |  |  |  |  |  |  |  |  |  |
|  |  | **8** | **20a** | 4.71 |  |  |  |  |  |  |  |  |  |  |
|  |  | **18b** | **13** | 4.73 |  |  |  |  |  |  |  |  |  |  |
|  |  | **4** | **11a** | 4.77 |  |  |  |  |  |  |  |  |  |  |
|  |  | **23ab** | **15b** | 4.81 |  |  |  |  |  |  |  |  |  |  |
|  |  | **22** | **15a** | 4.81 |  |  |  |  |  |  |  |  |  |  |
|  |  | **2** | **17b** | 4.82 |  |  |  |  |  |  |  |  |  |  |
|  |  | **14** | **11a** | 4.87 |  |  |  |  |  |  |  |  |  |  |
|  |  | **20a** | **13** | 4.89 |  |  |  |  |  |  |  |  |  |  |
|  |  | **18a** | **15a** | 4.89 |  |  |  |  |  |  |  |  |  |  |
|  |  | **4** | **8** | 4.89 |  |  |  |  |  |  |  |  |  |  |
|  |  | **4** | **11b/20b** | 4.90 |  |  |  |  |  |  |  |  |  |  |
|  |  | **1** | **18a** | 4.90 |  |  |  |  |  |  |  |  |  |  |
|  |  | **20a** | **17b** | 4.90 | 4 | 0.999 | 4.76 | -0.14 | 2.9 |  |  |  |  |  |
|  |  | **12** | **15a** | 4.98 |  |  |  |  |  |  |  |  |  |  |
|  |  | **1** | **13** | 4.99 |  |  |  |  |  |  |  |  |  |  |
|  | **5.00-5.49** | **4** | **1** | 5.00 |  |  |  |  |  |  |  |  |  |  |
|  |  | **1** | **8** | 5.06 |  |  |  |  |  |  |  |  |  |  |
|  |  | **17b** | **13** | 5.17 |  |  |  |  |  |  |  |  |  |  |
|  |  | **4** | **17a** | 5.17 |  |  |  |  |  |  |  |  |  |  |
|  |  | **22** | **13** | 5.19 |  |  |  |  |  |  |  |  |  |  |
|  |  | **1** | **18b** | 5.32 |  |  |  |  |  |  |  |  |  |  |
|  |  | **20a** | **17a** | 5.36 | 4 | 0.999 | 5.63 | 0.27 | 5.0 |  |  |  |  |  |
|  |  | **12** | **18b** | 5.38 |  |  |  |  |  |  |  |  |  |  |
|  |  | **2** | **15b** | 5.44 |  |  |  |  |  |  |  |  |  |  |
| **Beyond the NOE limit** | **5.50-5.99** | **22** | **15b** | 5.56 |  |  |  |  |  |  |  |  |  |  |
|  |  | **4** | **17b** | 5.71 |  |  |  |  |  |  |  |  |  |  |
|  |  | **22** | **16** | 5.71 |  |  |  |  |  |  |  |  |  |  |
|  |  | **1** | **20a** | 5.74 |  |  |  |  |  |  |  |  |  |  |
|  |  | **18a** | **13** | 5.89 |  |  |  |  |  |  |  |  |  |  |
|  |  | **3** | **13** | 5.90 |  |  |  |  |  |  |  |  |  |  |
|  |  | **3** | **17b** | 5.95 |  |  |  |  |  |  |  |  |  |  |
|  | **6.00-6.49** | **4** | **16** | 6.07 |  |  |  |  |  |  |  |  |  |  |
|  |  | **3** | **17a** | 6.09 |  |  |  |  |  |  |  |  |  |  |
|  |  | **3** | **15b** | 6.11 |  |  |  |  |  |  |  |  |  |  |
|  |  | **2** | **13** | 6.11 |  |  |  |  |  |  |  |  |  |  |
|  |  | **22** | **11a** | 6.14 |  |  |  |  |  |  |  |  |  |  |
|  |  | **3** | **16** | 6.16 |  |  |  |  |  |  |  |  |  |  |
|  | **6.50-6.99** | **3** | **8** | 6.53 |  |  |  |  |  |  |  |  |  |  |
|  |  | **2** | **8** | 6.59 |  |  |  |  |  |  |  |  |  |  |
|  |  | **11a** | **15a** | 6.74 |  |  |  |  |  | 4 | 0.979 | 4.10 | -2.64 | 39.2 |
|  |  | **4** | **14** | 6.82 |  |  |  |  |  |  |  |  |  |  |
|  |  | **12** | **18a** | 6.94 |  |  |  |  |  |  |  |  |  |  |
|  | **7.00-7.49** | **2** | **11b/20b** | 7.43 |  |  |  |  |  |  |  |  |  |  |
|  | **7.50-7.99** | **2** | **15a** | 7.67 |  |  |  |  |  |  |  |  |  |  |
|  |  | **1** | **22** | 7.71 |  |  |  |  |  |  |  |  |  |  |
|  |  | **2** | **14** | 7.80 |  |  |  |  |  |  |  |  |  |  |
|  | **8.00-8.49** | **3** | **14** | 8.09 |  |  |  |  |  |  |  |  |  |  |
|  | **8.50-8.99** | **4** | **22** | 8.70 |  |  |  |  |  |  |  |  |  |  |
|  | **9.00-9.49** | **3** | **20a** | 9.19 |  |  |  |  |  |  |  |  |  |  |
|  | **9.50-9.99** | **2** | **22** | 9.82 |  |  |  |  |  |  |  |  |  |  |
|  |  |  |  |  | **# Distances** | |  |  |  | **# Distances** | |  |  |  |
| **Total # of distances** | | | | 127 | 42 |  |  |  |  | 41 |  |  |  |  |
| **# Underestimated** | | | |  | 7 |  |  |  |  | 6 |  |  |  |  |
| **# Overestimated** | | | |  | 33 |  |  |  |  | 34 |  |  |  |  |

**Table S22**. Quantified distances for the EASY ROESY with solvent suppression roesyadesjsph^[1]^ pulse program from the matrix generated from the diagonal integrals directly (EASY ROESY ES) or after which the cross-peaks were adjusted to prevent the inclusion of noise (EASY ROESY ES adjusted cross-peaks). The theoretical population-averaged interproton distances as determined by Butts *et. al.*^[3]^ are given for each correlation, and used to calculate the error (Exp. – Theor.) as well as the absolute error (|%|). The number of data-points included (# t_mix_) for the distance determination, as well as the coefficient of determination (R^2^) of the linear regression are given. At the bottom of the table, the total number of quantified correlations as well as the number of over- and underestimated correlations are given. The proton-proton correlation used as a reference for distance calculation is being underscored.

|  |  |  |  |  | **EASY ROESY ES** | | | | | **EASY ROESY ES adjusted cross-peaks** | | | | |
| --- | --- | --- | --- | --- | --- | --- | --- | --- | --- | --- | --- | --- | --- | --- |
|  |  | **H_a_** | **H_b_** | **r_ab_**  **Theor. (Å)** | **# t_mix_** | **R^2^** | **r_ab_ (Å)** | **Exp.**  **-**  **Theor. (Å)** | **\|%\|** | **# t_mix_** | **R^2^** | **r_ab_ (Å)** | **Exp.**  **-**  **Theor. (Å)** | **\|%\|** |
| **Short reliable** | **0.00-1.99** | **20a** | **11b/20b** | 1.76 | 7 | 0.998 | 1.84 | 0.08 | 4.5 |  |  |  |  |  |
|  |  | **15a** | **15b** | 1.76 | 6 | 1.000 | 1.76 | 0.00 | 0.0 | 6 | 1.000 | 1.76 | 0.00 | 0.0 |
|  |  | **11a** | **11b/20b** | 1.76 |  |  |  |  |  |  |  |  |  |  |
|  |  | **17a** | **17b** | 1.77 |  |  |  |  |  |  |  |  |  |  |
|  |  | **18a** | **18b** | 1.77 | 7 | 0.970 | 1.79 | 0.02 | 1.1 | 6 | 0.991 | 1.78 | 0.01 | 0.6 |
|  | **2.00-2.49** | **20a** | **15a** | 2.24 | 7 | 1.000 | 2.11 | -0.13 | 5.8 | 7 | 1.000 | 2.13 | -0.11 | 4.9 |
|  |  | **15b** | **13** | 2.26 |  |  |  |  |  |  |  |  |  |  |
|  |  | **12** | **23ab** | 2.27 | 6 | 0.987 | 2.22 | -0.05 | 2.2 | 7 | 0.978 | 2.53 | 0.26 | 11.5 |
|  |  | **22** | **23ab** | 2.30 |  |  |  |  |  |  |  |  |  |  |
|  |  | **12** | **13** | 2.35 | 7 | 0.998 | 2.27 | -0.08 | 3.4 | 7 | 0.993 | 2.36 | 0.01 | 0.4 |
|  |  | **12** | **11a** | 2.35 | 7 | 0.959 | 2.00 | -0.35 | 14.9 | 4 | 0.982 | 2.20 | -0.15 | 6.4 |
|  |  | **8** | **18b** | 2.37 | 7 | 1.000 | 2.23 | -0.14 | 5.9 | 7 | 1.000 | 2.24 | -0.13 | 5.5 |
|  |  | **1** | **16** | 2.39 | 7 | 1.000 | 2.24 | -0.15 | 6.3 | 7 | 1.000 | 2.24 | -0.15 | 6.3 |
|  |  | **22** | **11b/20b** | 2.39 | 7 | 0.999 | 2.49 | 0.10 | 4.2 | 7 | 1.000 | 2.53 | 0.14 | 5.9 |
|  |  | **14** | **13** | 2.41 | 7 | 0.999 | 2.33 | -0.08 | 3.3 | 7 | 0.998 | 2.39 | -0.02 | 0.8 |
|  |  | **8** | **17a** | 2.42 | 7 | 0.999 | 2.33 | -0.09 | 3.7 | 7 | 0.999 | 2.33 | -0.09 | 3.7 |
|  |  | **18a** | **17b** | 2.43 | 6 | 0.993 | 2.39 | -0.04 | 1.6 |  |  |  |  |  |
|  |  | **18b** | **17a** | 2.43 | 4 | 0.985 | 2.47 | 0.04 | 1.6 |  |  |  |  |  |
|  |  | **18b** | **11b/20b** | 2.45 |  |  |  |  |  |  |  |  |  |  |
|  |  | **16** | **15a** | 2.48 | 7 | 0.988 | 2.42 | -0.06 | 2.4 | 7 | 0.969 | 2.50 | 0.02 | 0.8 |
|  |  | **16** | **15b** | 2.48 | 7 | 0.983 | 2.55 | 0.07 | 2.8 | 5 | 0.958 | 3.16 | 0.68 | 27.4 |
|  |  | **3** | **2** | 2.48 |  |  |  |  |  |  |  |  |  |  |
|  |  | **14** | **15a** | 2.49 | 6 | 1.000 | 2.50 | 0.01 | 0.4 | 6 | 1.000 | 2.50 | 0.01 | 0.4 |
|  |  | **1** | **2** | 2.49 |  |  |  |  |  |  |  |  |  |  |
|  | **2.50-2.99** | **4** | **3** | 2.50 |  |  |  |  |  |  |  |  |  |  |
|  |  | **14** | **15b** | 2.55 | 7 | 0.997 | 2.55 | 0.00 | 0.0 | 5 | 0.959 | 2.97 | 0.42 | 16.5 |
|  |  | **23ab** | **14** | 2.59 | 7 | 0.999 | 2.54 | -0.05 | 1.9 | 7 | 0.999 | 2.58 | -0.01 | 0.4 |
|  |  | **8** | **11b/20b** | 2.63 | 7 | 1.000 | 2.67 | 0.04 | 1.5 | 7 | 1.000 | 2.70 | 0.07 | 2.7 |
|  |  | **16** | **17b** | 2.71 | 7 | 0.999 | 2.59 | -0.12 | 4.4 | 7 | 0.996 | 2.61 | -0.10 | 3.7 |
|  |  | **18a** | **17a** | 2.73 | 7 | 0.981 | 2.76 | 0.03 | 1.1 | 6 | 0.985 | 2.88 | 0.15 | 5.5 |
|  |  | **12** | **14** | 2.74 | 7 | 0.997 | 2.65 | -0.09 | 3.3 | 7 | 0.997 | 2.68 | -0.06 | 2.2 |
|  |  | **1** | **17b** | 2.79 | 7 | 0.999 | 2.75 | -0.04 | 1.4 | 7 | 0.999 | 2.75 | -0.04 | 1.4 |
|  |  | **18a** | **11b/20b** | 2.82 | 6 | 0.973 | 2.78 | -0.04 | 1.4 | 7 | 0.952 | 3.20 | 0.38 | 13.5 |
|  |  | **12** | **11b/20b** | 2.93 |  |  |  |  |  |  |  |  |  |  |
| **Long reliable** | **3.00-3.49** | **8** | **13** | 3.01 | 6 | 1.000 | 2.91 | -0.10 | 3.3 | 6 | 0.999 | 2.91 | -0.10 | 3.3 |
|  |  | **22** | **18b** | 3.16 | 7 | 0.999 | 2.82 | -0.34 | 10.8 | 7 | 1.000 | 2.87 | -0.29 | 9.2 |
|  |  | **23ab** | **11b/20b** | 3.24 | 7 | 0.980 | 3.36 | 0.12 | 3.7 | 7 | 0.977 | 3.51 | 0.27 | 8.3 |
|  |  | **20a** | **14** | 3.36 | 6 | 0.985 | 3.32 | -0.04 | 1.2 | 6 | 0.963 | 3.40 | 0.04 | 1.2 |
|  |  | **16** | **20a** | 3.37 | 4 | 0.951 | 3.44 | 0.07 | 2.1 | 4 | 0.951 | 3.47 | 0.10 | 3.0 |
|  |  | **11a** | **13** | 3.40 | 7 | 0.977 | 3.44 | 0.04 | 1.2 | 7 | 0.975 | 3.78 | 0.38 | 11.2 |
|  |  | **22** | **20a** | 3.42 |  |  |  |  |  |  |  |  |  |  |
|  | **3.50-3.99** | **15a** | **13** | 3.54 |  |  |  |  |  |  |  |  |  |  |
|  |  | **8** | **17b** | 3.61 | 6 | 0.960 | 3.56 | -0.05 | 1.4 | 7 | 0.965 | 3.53 | -0.08 | 2.2 |
|  |  | **11b/20b** | **13** | 3.61 | 4 | 0.980 | 4.00 | 0.39 | 10.8 | 4 | 0.964 | 4.87 | 1.26 | 34.9 |
|  |  | **16** | **18a** | 3.63 | 5 | 0.959 | 3.68 | 0.05 | 1.4 | 5 | 0.957 | 3.69 | 0.06 | 1.7 |
|  |  | **12** | **8** | 3.65 |  |  |  |  |  |  |  |  |  |  |
|  |  | **11b/20b** | **15a** | 3.69 | 5 | 0.976 | 3.01 | -0.68 | 18.4 | 5 | 0.990 | 3.07 | -0.62 | 16.8 |
|  |  | **23ab** | **8** | 3.73 |  |  |  |  |  |  |  |  |  |  |
|  |  | **20a** | **15b** | 3.75 | 6 | 0.980 | 3.00 | -0.75 | 20.0 | 6 | 0.968 | 3.10 | -0.65 | 17.3 |
|  |  | **23ab** | **13** | 3.76 |  |  |  |  |  |  |  |  |  |  |
|  |  | **8** | **18a** | 3.83 |  |  |  |  |  |  |  |  |  |  |
|  |  | **16** | **17a** | 3.84 | 7 | 0.952 | 3.71 | -0.13 | 3.4 | 7 | 0.958 | 3.69 | -0.15 | 3.9 |
|  |  | **1** | **15b** | 3.86 | 7 | 0.958 | 4.11 | 0.25 | 6.5 | 7 | 0.985 | 4.40 | 0.54 | 14.0 |
|  |  | **22** | **14** | 3.86 | 7 | 0.967 | 3.86 | 0.00 | 0.0 | 7 | 0.980 | 4.11 | 0.25 | 6.5 |
|  |  | **8** | **11a** | 3.88 |  |  |  |  |  |  |  |  |  |  |
|  |  | **16** | **18b** | 3.89 | 7 | 0.984 | 3.97 | 0.08 | 2.1 | 7 | 0.980 | 4.13 | 0.24 | 6.2 |
|  |  | **23ab** | **11a** | 3.96 |  |  |  |  |  |  |  |  |  |  |
|  |  | **14** | **11b/20b** | 3.96 |  |  |  |  |  | 4 | 0.956 | 4.51 | 0.55 | 13.9 |
|  |  | **8** | **14** | 3.96 | 4 | 0.995 | 3.78 | -0.18 | 4.5 | 4 | 0.982 | 4.13 | 0.17 | 4.3 |
|  |  | **22** | **8** | 3.96 | 7 | 0.997 | 3.80 | -0.16 | 4.0 | 7 | 0.997 | 3.97 | 0.01 | 0.3 |
|  | **4.00-4.49** | **1** | **17a** | 4.06 | 5 | 0.972 | 4.22 | 0.16 | 3.9 |  |  |  |  |  |
|  |  | **16** | **11b/20b** | 4.07 | 7 | 0.982 | 4.25 | 0.18 | 4.4 |  |  |  |  |  |
|  |  | **8** | **15b** | 4.09 |  |  |  |  |  |  |  |  |  |  |
|  |  | **16** | **8** | 4.14 |  |  |  |  |  |  |  |  |  |  |
|  |  | **11b/20b** | **17a** | 4.17 |  |  |  |  |  |  |  |  |  |  |
|  |  | **23ab** | **18b** | 4.27 | 5 | 0.999 | 4.22 | -0.05 | 1.2 | 5 | 0.969 | 4.33 | 0.06 | 1.4 |
|  |  | **12** | **15b** | 4.27 | 4 | 0.999 | 3.11 | -1.16 | 27.2 |  |  |  |  |  |
|  |  | **4** | **13** | 4.43 | 7 | 0.967 | 4.33 | -0.10 | 2.3 | 7 | 0.959 | 4.73 | 0.30 | 6.8 |
|  |  | **22** | **18a** | 4.47 |  |  |  |  |  |  |  |  |  |  |
| **At the NOE limit** | **4.50-4.99** | **18b** | **15a** | 4.55 |  |  |  |  |  |  |  |  |  |  |
|  |  | **11b/20b** | **15b** | 4.59 |  |  |  |  |  |  |  |  |  |  |
|  |  | **1** | **15a** | 4.62 |  |  |  |  |  | 4 | 0.965 | 7.43 | 2.81 | 60.8 |
|  |  | **8** | **15a** | 4.63 |  |  |  |  |  |  |  |  |  |  |
|  |  | **17b** | **15b** | 4.64 |  |  |  |  |  |  |  |  |  |  |
|  |  | **22** | **12** | 4.65 |  |  |  |  |  |  |  |  |  |  |
|  |  | **14** | **18b** | 4.66 |  |  |  |  |  |  |  |  |  |  |
|  |  | **23ab** | **15a** | 4.68 |  |  |  |  |  |  |  |  |  |  |
|  |  | **8** | **20a** | 4.71 | 4 | 0.963 | 3.78 | -0.93 | 19.7 |  |  |  |  |  |
|  |  | **18b** | **13** | 4.73 |  |  |  |  |  |  |  |  |  |  |
|  |  | **4** | **11a** | 4.77 | 6 | 0.969 | 4.83 | 0.06 | 1.3 |  |  |  |  |  |
|  |  | **23ab** | **15b** | 4.81 |  |  |  |  |  |  |  |  |  |  |
|  |  | **22** | **15a** | 4.81 |  |  |  |  |  |  |  |  |  |  |
|  |  | **2** | **17b** | 4.82 | 6 | 0.992 | 4.67 | -0.15 | 3.1 | 5 | 0.984 | 4.95 | 0.13 | 2.7 |
|  |  | **14** | **11a** | 4.87 | 4 | 0.988 | 3.87 | -1.00 | 20.5 |  |  |  |  |  |
|  |  | **20a** | **13** | 4.89 |  |  |  |  |  |  |  |  |  |  |
|  |  | **18a** | **15a** | 4.89 | 4 | 0.983 | 3.52 | -1.37 | 28.0 | 4 | 0.967 | 3.66 | -1.23 | 25.2 |
|  |  | **4** | **8** | 4.89 | 6 | 0.981 | 5.13 | 0.24 | 4.9 |  |  |  |  |  |
|  |  | **4** | **11b/20b** | 4.90 | 6 | 0.952 | 5.13 | 0.23 | 4.7 | 6 | 0.964 | 5.60 | 0.70 | 14.3 |
|  |  | **1** | **18a** | 4.90 |  |  |  |  |  |  |  |  |  |  |
|  |  | **20a** | **17b** | 4.90 |  |  |  |  |  |  |  |  |  |  |
|  |  | **12** | **15a** | 4.98 | 4 | 0.965 | 4.97 | -0.01 | 0.2 |  |  |  |  |  |
|  |  | **1** | **13** | 4.99 | 5 | 0.975 | 5.07 | 0.08 | 1.6 | 7 | 0.981 | 5.52 | 0.53 | 10.6 |
|  | **5.00-5.49** | **4** | **1** | 5.00 |  |  |  |  |  |  |  |  |  |  |
|  |  | **1** | **8** | 5.06 |  |  |  |  |  | 4 | 0.985 | 5.25 | 0.19 | 3.8 |
|  |  | **17b** | **13** | 5.17 |  |  |  |  |  |  |  |  |  |  |
|  |  | **4** | **17a** | 5.17 | 5 | 0.966 | 5.04 | -0.13 | 2.5 | 5 | 0.967 | 5.27 | 0.10 | 1.9 |
|  |  | **22** | **13** | 5.19 |  |  |  |  |  |  |  |  |  |  |
|  |  | **1** | **18b** | 5.32 |  |  |  |  |  |  |  |  |  |  |
|  |  | **20a** | **17a** | 5.36 |  |  |  |  |  |  |  |  |  |  |
|  |  | **12** | **18b** | 5.38 |  |  |  |  |  |  |  |  |  |  |
|  |  | **2** | **15b** | 5.44 |  |  |  |  |  |  |  |  |  |  |
| **Beyond the NOE limit** | **5.50-5.99** | **22** | **15b** | 5.56 |  |  |  |  |  |  |  |  |  |  |
|  |  | **4** | **17b** | 5.71 | 4 | 0.988 | 4.95 | -0.76 | 13.3 |  |  |  |  |  |
|  |  | **22** | **16** | 5.71 | 5 | 0.977 | 5.11 | -0.60 | 10.5 | 4 | 0.989 | 5.11 | -0.60 | 10.5 |
|  |  | **1** | **20a** | 5.74 |  |  |  |  |  |  |  |  |  |  |
|  |  | **18a** | **13** | 5.89 |  |  |  |  |  |  |  |  |  |  |
|  |  | **3** | **13** | 5.90 | 4 | 0.978 | 5.91 | 0.01 | 0.2 |  |  |  |  |  |
|  |  | **3** | **17b** | 5.95 |  |  |  |  |  |  |  |  |  |  |
|  | **6.00-6.49** | **4** | **16** | 6.07 |  |  |  |  |  |  |  |  |  |  |
|  |  | **3** | **17a** | 6.09 | 4 | 0.953 | 6.37 | 0.28 | 4.6 |  |  |  |  |  |
|  |  | **3** | **15b** | 6.11 |  |  |  |  |  |  |  |  |  |  |
|  |  | **2** | **13** | 6.11 |  |  |  |  |  |  |  |  |  |  |
|  |  | **22** | **11a** | 6.14 |  |  |  |  |  | 4 | 0.955 | 5.77 | -0.37 | 6.0 |
|  |  | **3** | **16** | 6.16 | 4 | 0.988 | 5.42 | -0.74 | 12.0 |  |  |  |  |  |
|  | **6.50-6.99** | **3** | **8** | 6.53 |  |  |  |  |  |  |  |  |  |  |
|  |  | **2** | **8** | 6.59 |  |  |  |  |  |  |  |  |  |  |
|  |  | **11a** | **15a** | 6.74 |  |  |  |  |  |  |  |  |  |  |
|  |  | **4** | **14** | 6.82 |  |  |  |  |  |  |  |  |  |  |
|  |  | **12** | **18a** | 6.94 |  |  |  |  |  |  |  |  |  |  |
|  | **7.00-7.49** | **2** | **11b/20b** | 7.43 |  |  |  |  |  |  |  |  |  |  |
|  | **7.50-7.99** | **2** | **15a** | 7.67 | 4 | 0.988 | 4.05 | -3.62 | 47.2 | 4 | 0.988 | 4.05 | -3.62 | 47.2 |
|  |  | **1** | **22** | 7.71 |  |  |  |  |  |  |  |  |  |  |
|  |  | **2** | **14** | 7.80 |  |  |  |  |  |  |  |  |  |  |
|  | **8.00-8.49** | **3** | **14** | 8.09 |  |  |  |  |  |  |  |  |  |  |
|  | **8.50-8.99** | **4** | **22** | 8.70 |  |  |  |  |  |  |  |  |  |  |
|  | **9.00-9.49** | **3** | **20a** | 9.19 |  |  |  |  |  |  |  |  |  |  |
|  | **9.50-9.99** | **2** | **22** | 9.82 | 4 | 0.994 | 6.03 | -3.79 | 38.6 |  |  |  |  |  |
|  |  |  |  |  | **# Distances** | |  |  |  | **# Distances** | |  |  |  |
| **Total # of distances** | | | | 127 | 64 |  |  |  |  | 52 |  |  |  |  |
| **# Underestimated** | | | |  | 38 |  |  |  |  | 20 |  |  |  |  |
| **# Overestimated** | | | |  | 23 |  |  |  |  | 31 |  |  |  |  |

**4. Distance determination from NOEs obtained from a single mixing time**

Instead of calculating the interproton distances based on the build-up rates, one may wish to record a single NOESY or ROESY spectrum and determine the interproton distances based on the normalised intensity obtained from that single spectrum (Eq. [1] and [2]) using Eq. [S1] instead of Eq. [3].

$r_{ab}=1.76\times\sqrt[6]{\frac{I_{ref}}{I_{ab}}}$	[S1]

The integration matrices that were used for the generation of the build-up curves of the seven NOESY spectra (noesyph, with 100-700 ms mixing times) were used to calculate the intensity based interproton distances, based on a single spectrum recorded at various mixing times. Here, the absolute integral values were used for distance determination, ignoring the sign of the cross-peaks, resulting in a maximum number of quantified correlations. There is a total of 190 potential correlations for strychnine, 70 of which are separated by 5.50 Å or more. These 70 correlations will not give rise to an NOE, as the NOE has a reach of only about 5.00 Å. Correlations that correspond to protons separated by 5.50 Å or more but could be quantified are the result of an artefact rather than an NOE.

*All cross-peaks*

In total all 190 correlations could be quantified when the absolute cross-peak intensities were used (Table S23 and S29), with an average absolute error on the determined distances between 3.5 and 44.0% for the different distance ranges (Table S24). Distances derived from the spectra recorded with 100 and 200 ms mixing times showed the largest average absolute errors and were generally found between 15 and 45% for all distance ranges. The error on the distances derived from spectra with higher mixing times (300-700 ms) was found not to be much lower. Even though a lower average absolute error for distances up to 3.00 Å was found (generally around 5%), the average absolute errors still varied between 7.2 and 43.3% for distances beyond 3.00 Å. Because of the excessively large error on the distances, especially on those of 3.00 Å and beyond, distances derived by this method are not suitable for structure determination.

**Table S23**. Number of correlations that could be quantified with the NOESY pulse program noesyph for each of the 7 mixing times.

|  | **Distance range (Å)** | **100** | **200** | **300** | **400** | **500** | **600** | **700** |
| --- | --- | --- | --- | --- | --- | --- | --- | --- |
| **Short reliable** | 0.00-1.99 | 5 | 5 | 5 | 5 | 5 | 5 | 5 |
|  | 2.00-2.49 | 19 | 19 | 19 | 19 | 19 | 19 | 19 |
|  | 2.50-2.99 | 10 | 10 | 10 | 10 | 10 | 10 | 10 |
| **Long reliable** | 3.00-3.49 | 7 | 7 | 7 | 7 | 7 | 7 | 7 |
|  | 3.50-3.99 | 22 | 22 | 22 | 22 | 22 | 22 | 22 |
|  | 4.00-4.49 | 15 | 15 | 15 | 15 | 15 | 15 | 15 |
| **At the NOE limit** | 4.50-4.99 | 29 | 29 | 29 | 29 | 29 | 29 | 29 |
|  | 5.00-5.49 | 13 | 13 | 13 | 13 | 13 | 13 | 13 |
| **Beyond the NOE limit** | 5.50-5.99 | 19 | 19 | 19 | 19 | 19 | 19 | 19 |
|  | 6.00-6.49 | 11 | 11 | 11 | 11 | 11 | 11 | 11 |
|  | 6.50-6.99 | 9 | 9 | 9 | 9 | 9 | 9 | 9 |
|  | 7.00-7.49 | 10 | 10 | 10 | 10 | 10 | 10 | 10 |
|  | 7.50-7.99 | 8 | 8 | 8 | 8 | 8 | 8 | 8 |
|  | 8.00-8.49 | 7 | 7 | 7 | 7 | 7 | 7 | 7 |
|  | 8.50-8.99 | 3 | 3 | 3 | 3 | 3 | 3 | 3 |
|  | 9.00-9.49 | 1 | 1 | 1 | 1 | 1 | 1 | 1 |
|  | 9.50-9.99 | 1 | 1 | 1 | 1 | 1 | 1 | 1 |
|  | 10.00-10.49 | 1 | 1 | 1 | 1 | 1 | 1 | 1 |
| **Total** |  |  |  |  |  |  |  |  |
| **Short reliable** | 0.00-2.99 | 34 | 34 | 34 | 34 | 34 | 34 | 34 |
| **Long reliable** | 3.00-4.49 | 44 | 44 | 44 | 44 | 44 | 44 | 44 |
| **At the NOE limit** | 4.50-5.49 | 42 | 42 | 42 | 42 | 42 | 42 | 42 |
| **Beyond the NOE limit** | 5.50-9.99 | 70 | 70 | 70 | 70 | 70 | 70 | 70 |
| **All** | 0.00-10.49 | 190 | 190 | 190 | 190 | 190 | 190 | 190 |

**Table S24**. Average absolute error (%) on the calculated distances for which the correlations were quantified based on the normalised intensity (single spectrum) with the NOESY pulse program noesyph for each of the 7 mixing times.

|  | **Distance range (Å)** | **100** | **200** | **300** | **400** | **500** | **600** | **700** |
| --- | --- | --- | --- | --- | --- | --- | --- | --- |
| **Short reliable** | 0.00-1.99 | 22.4 | 15.3 | 4.0 | 3.8 | 4.2 | 3.8 | 3.5 |
|  | 2.00-2.49 | 29.5 | 10.1 | 5.4 | 5.0 | 4.4 | 3.9 | 3.5 |
|  | 2.50-2.99 | 36.1 | 5.9 | 9.5 | 3.7 | 4.0 | 4.8 | 5.6 |
| **Long reliable** | 3.00-3.49 | 25.1 | 12.2 | 8.8 | 26.2 | 16.1 | 9.5 | 7.2 |
|  | 3.50-3.99 | 32.9 | 14.3 | 14.9 | 16.5 | 17.7 | 12.2 | 11.4 |
|  | 4.00-4.49 | 23.2 | 14.4 | 17.6 | 21.3 | 17.4 | 24.4 | 15.8 |
| **At the NOE limit** | 4.50-4.99 | 23.8 | 14.1 | 14.7 | 15.7 | 17.3 | 14.3 | 11.2 |
|  | 5.00-5.49 | 15.8 | 10.9 | 11.0 | 18.6 | 15.2 | 17.3 | 14.6 |
| **Beyond the NOE limit** | 5.50-5.99 | 12.5 | 14.4 | 13.3 | 10.4 | 15.2 | 16.8 | 15.3 |
|  | 6.00-6.49 | 5.5 | 12.2 | 10.5 | 14.6 | 9.9 | 12.2 | 7.9 |
|  | 6.50-6.99 | 18.7 | 20.7 | 19.3 | 13.6 | 14.1 | 17.3 | 14.3 |
|  | 7.00-7.49 | 20.1 | 20.1 | 17.7 | 15.0 | 16.1 | 13.8 | 13.2 |
|  | 7.50-7.99 | 19.6 | 23.2 | 22.9 | 20.0 | 20.2 | 19.9 | 22.6 |
|  | 8.00-8.49 | 23.9 | 27.8 | 27.2 | 24.6 | 16.4 | 24.1 | 18.7 |
|  | 8.50-8.99 | 44.0 | 36.7 | 32.0 | 32.5 | 19.8 | 28.4 | 30.9 |
|  | 9.00-9.49 | 16.0 | 26.8 | 33.6 | 17.5 | 29.5 | 31.9 | 33.5 |
|  | 9.50-9.99 | 37.2 | 37.3 | 11.5 | 43.3 | 30.6 | 35.5 | 32.6 |
|  | 10.00-10.49 | 40.6 | 42.8 | 41.8 | 17.6 | 28.7 | 39.1 | 12.4 |
| **Total** |  |  |  |  |  |  |  |  |
| **Short reliable** | 0.00-2.99 | 30.4 | 9.7 | 6.4 | 4.5 | 4.3 | 4.1 | 4.1 |
| **Long reliable** | 3.00-4.49 | 28.3 | 14.0 | 14.8 | 19.7 | 17.3 | 15.9 | 12.3 |
| **At the NOE limit** | 4.50-5.49 | 21.3 | 13.1 | 13.5 | 16.6 | 16.6 | 15.2 | 12.3 |
| **Beyond the NOE limit** | 5.50-9.99 | 18.5 | 19.9 | 18.2 | 16.3 | 15.8 | 18.1 | 16.0 |
| **All** | 0.00-10.49 | 23.5 | 15.2 | 14.3 | 15.0 | 14.3 | 14.5 | 12.2 |

*Data quality checks*

To ensure that only the reliable NOE correlations are used for structure elucidation, the following quality checks are recommended, independently of whether a full build-up analysis is performed or the intensities from a single NOESY spectrum are assessed.

*Excluding correlations with opposite phases*

The first quality check concerns inspection of the phase of the cross-peaks. Independent of whether the studied object tumbles fast (positive cross-peaks, opposite phase of the diagonal peaks) or slow (negative cross-peaks, the same phase of the diagonal peaks), the cross-peaks belonging to a specific correlation should have the same phase on either side of the diagonal. Correlations with cross-peaks of opposite phases on each side of the diagonal are artefacts rather than genuine correlations. By excluding correlations that have cross-peaks with opposite signs, 107 to 144 quantified correlations in the single NOESY spectrum could be obtained for each of the mixing times (Table S25 and S30). Furthermore, the overall average absolute error was reduced by about 2% for most mixing times, but the effect of this quality check is most noticeable at the reduction in number of correlations that were theoretically beyond 4.0 Å (Table S26). The NOE has a reach of only about 5.00 Å, and the longer the interproton spacing the weaker the NOE cross-peak. About half of the correlations yielding distances beyond 4.50 Å were excluded by this first quality check.

**Table S25**. Number of interproton distances that could be quantified with the NOESY pulse program noesyph from correlations for which the underlaying cross-peaks have the same phase for each of the 7 mixing times.

|  | **Distance range (Å)** | **100** | **200** | **300** | **400** | **500** | **600** | **700** |
| --- | --- | --- | --- | --- | --- | --- | --- | --- |
| **Short reliable** | 0.00-1.99 | 5 | 5 | 5 | 5 | 5 | 5 | 5 |
|  | 2.00-2.49 | 15 | 17 | 19 | 19 | 19 | 19 | 19 |
|  | 2.50-2.99 | 9 | 10 | 9 | 10 | 10 | 10 | 10 |
| **Long reliable** | 3.00-3.49 | 7 | 7 | 7 | 7 | 7 | 7 | 7 |
|  | 3.50-3.99 | 14 | 19 | 21 | 16 | 18 | 19 | 22 |
|  | 4.00-4.49 | 10 | 11 | 11 | 9 | 12 | 11 | 13 |
| **At the NOE limit** | 4.50-4.99 | 7 | 12 | 13 | 15 | 17 | 17 | 24 |
|  | 5.00-5.49 | 6 | 4 | 4 | 7 | 6 | 7 | 7 |
| **Beyond the NOE limit** | 5.50-5.99 | 8 | 7 | 12 | 11 | 9 | 9 | 9 |
|  | 6.00-6.49 | 4 | 5 | 6 | 4 | 7 | 6 | 7 |
|  | 6.50-6.99 | 5 | 6 | 4 | 4 | 4 | 6 | 6 |
|  | 7.00-7.49 | 5 | 6 | 5 | 4 | 5 | 4 | 3 |
|  | 7.50-7.99 | 4 | 5 | 4 | 3 | 3 | 3 | 6 |
|  | 8.00-8.49 | 3 | 4 | 5 | 4 | 4 | 3 | 2 |
|  | 8.50-8.99 | 3 | 2 | 3 | 3 | 0 | 2 | 2 |
|  | 9.00-9.49 | 0 | 1 | 1 | 1 | 1 | 1 | 1 |
|  | 9.50-9.99 | 1 | 0 | 0 | 0 | 1 | 1 | 1 |
|  | 10.00-10.49 | 1 | 0 | 1 | 1 | 1 | 0 | 0 |
| **Total** |  |  |  |  |  |  |  |  |
| **Short reliable** | 0.00-2.99 | 29 | 32 | 33 | 34 | 34 | 34 | 34 |
| **Long reliable** | 3.00-4.49 | 31 | 37 | 39 | 32 | 37 | 37 | 42 |
| **At the NOE limit** | 4.50-5.49 | 13 | 16 | 17 | 22 | 23 | 24 | 31 |
| **Beyond the NOE limit** | 5.50-9.99 | 34 | 36 | 41 | 35 | 35 | 35 | 37 |
| **All** | 0.00-10.49 | 107 | 121 | 130 | 123 | 129 | 130 | 144 |

**Table S26**. Average absolute error (%) on the calculated distances for which the interproton distances were quantified based on the normalised intensity (single spectrum) for which the underlaying cross-peaks have the same phase with the NOESY pulse program noesyph for each of the 7 mixing times.

|  | **Distance range (Å)** | **100** | **200** | **300** | **400** | **500** | **600** | **700** |
| --- | --- | --- | --- | --- | --- | --- | --- | --- |
| **Short reliable** | 0.00-1.99 | 22.4 | 15.3 | 4.0 | 3.8 | 4.2 | 3.8 | 3.5 |
|  | 2.00-2.49 | 20.0 | 7.2 | 5.4 | 5.0 | 4.4 | 3.9 | 3.5 |
|  | 2.50-2.99 | 29.2 | 5.9 | 4.7 | 3.7 | 4.0 | 4.8 | 5.6 |
| **Long reliable** | 3.00-3.49 | 25.1 | 12.2 | 8.8 | 26.2 | 16.1 | 9.5 | 7.2 |
|  | 3.50-3.99 | 33.7 | 10.3 | 14.0 | 10.8 | 14.4 | 9.6 | 11.4 |
|  | 4.00-4.49 | 23.6 | 15.1 | 15.7 | 16.0 | 13.6 | 18.6 | 14.3 |
| **At the NOE limit** | 4.50-4.99 | 26.3 | 8.8 | 14.7 | 14.5 | 17.6 | 14.0 | 11.8 |
|  | 5.00-5.49 | 13.0 | 7.1 | 4.5 | 12.5 | 11.5 | 10.4 | 10.2 |
| **Beyond the NOE limit** | 5.50-5.99 | 13.0 | 10.2 | 14.6 | 10.0 | 12.6 | 13.4 | 20.5 |
|  | 6.00-6.49 | 10.3 | 7.4 | 8.9 | 17.9 | 7.2 | 14.3 | 7.3 |
|  | 6.50-6.99 | 20.2 | 21.1 | 23.6 | 10.4 | 10.5 | 11.7 | 18.3 |
|  | 7.00-7.49 | 21.2 | 19.3 | 20.3 | 16.5 | 13.3 | 9.4 | 13.5 |
|  | 7.50-7.99 | 18.7 | 26.4 | 31.4 | 16.2 | 20.4 | 28.0 | 24.0 |
|  | 8.00-8.49 | 26.0 | 24.7 | 28.4 | 22.4 | 20.6 | 24.7 | 26.2 |
|  | 8.50-8.99 | 44.0 | 39.8 | 32.0 | 30.1 | - | 32.0 | 26.6 |
|  | 9.00-9.49 | - | 26.8 | 33.6 | 17.5 | 29.5 | 31.9 | 33.5 |
|  | 9.50-9.99 | 37.2 | - | - | - | 30.6 | 35.5 | 32.6 |
|  | 10.00-10.49 | 40.6 | - | 41.8 | 17.6 | 28.7 | - | - |
| **Total** |  |  |  |  |  |  |  |  |
| **Short reliable** | 0.00-2.99 | 23.3 | 8.1 | 5.0 | 4.5 | 4.3 | 4.1 | 4.1 |
| **Long reliable** | 3.00-4.49 | 28.5 | 12.1 | 13.6 | 15.6 | 14.5 | 12.2 | 11.6 |
| **At the NOE limit** | 4.50-5.49 | 20.1 | 8.3 | 12.3 | 13.9 | 16.0 | 12.9 | 11.4 |
| **Beyond the NOE limit** | 5.50-9.99 | 21.0 | 19.1 | 21.1 | 14.9 | 14.4 | 17.3 | 19.0 |
| **All** | 0.00-10.49 | 23.7 | 12.6 | 13.6 | 12.0 | 12.1 | 11.6 | 11.7 |

*Excluding distances beyond the NOE-regime*

NOEs are detectable up to about 5.00 Å interproton distance. Thus, it is safe to assume that NOE-derived distances well beyond this limit are a result of an artefact rather than from genuine NOEs. Therefore, in addition to removing correlations with cross-peaks with opposite phases, distances that are calculated to be beyond 5.50 Å should, as a rule, be removed from any NOE-based analyses, and distances beyond 4.50 Å should be carefully considered and be given a lower weight (larger expected error) than shorter distances.

Tables S27, S28 and S31 show the number of distances that could be quantified when including these two quality checks and their corresponding average absolute errors (%). The total number of correlations were found to be between 72 and 101, depending on the mixing time, with the above two checks in place. The number of distances found beyond 4.50 Å was drastically reduced, with about 20% of the originally quantified correlations left. As a result, the remaining correlations that were found around the NOE-limit (between 4.50 and 5.49 Å) are most likely the result of genuine NOEs. Removing the artefacts reduced the average absolute error by 10-15% around the NOE-limit. Following the exclusion of obvious artefacts using the above two basic checks, there were still 2-14 quantified correlations that led to artificial distances, with at least 5 correlations for all spectra except for those obtained with 500 ms mixing time. These peaks would ultimately skew any structure elucidation process by including false restraints.

The implementation of these two quality checks vastly reduces the number of artificial distances being included. However, the accuracy of the distances derived for the remaining correlations did not improve significantly. The average absolute errors of distances obtained from spectra recorded with 100 or 200 ms mixing time were well above 5%, and even 20% in most instances. The improvement of the accuracy of the distances obtained from 300-700 ms mixing time spectra was minimal. Distances beyond 3.00 Å still had average absolute errors 10-20%.

Overall, performing the two basic quality checks of (i) removing correlations from cross-peaks with different phases, and (ii) removing correlations yielding distances >5.50 Å removes the outliers suffering from the most extreme individual errors (up to 130%, Table S29). Still, the individual error of the remaining correlations, after the above exclusion of obvious artefacts, may reach as high as 67% (Table S31). However, this does not improve the accuracy of the determined distances from the remaining cross-peaks. The error of the distances determined from a single NOESY spectrum is beyond 10% in most instances, which is too large for any meaningful structure determination.

**Table S27**. Number of correlations that could be quantified with the NOESY pulse program noesyph from correlations for which the underlaying cross-peaks have the same phase for each of the 7 mixing times. Correlations for which distances were calculated to be 5.50 Å or larger were excluded.

|  | **Distance range (Å)** | **100** | **200** | **300** | **400** | **500** | **600** | **700** |
| --- | --- | --- | --- | --- | --- | --- | --- | --- |
| **Short reliable** | 0.00-1.99 | 5 | 5 | 5 | 5 | 5 | 5 | 5 |
|  | 2.00-2.49 | 16 | 17 | 19 | 19 | 19 | 19 | 19 |
|  | 2.50-2.99 | 9 | 10 | 10 | 10 | 10 | 10 | 10 |
| **Long reliable** | 3.00-3.49 | 7 | 7 | 7 | 7 | 7 | 7 | 7 |
|  | 3.50-3.99 | 12 | 19 | 21 | 16 | 16 | 19 | 22 |
|  | 4.00-4.49 | 8 | 10 | 10 | 8 | 11 | 10 | 12 |
| **At the NOE limit** | 4.50-4.99 | 4 | 9 | 7 | 9 | 10 | 11 | 16 |
|  | 5.00-5.49 | 2 | 3 | 2 | 4 | 3 | 2 | 4 |
| **Beyond the NOE limit** | 5.50-5.99 | 2 | 4 | 4 | 6 | 1 | 3 | 2 |
|  | 6.00-6.49 | 1 | 1 | 1 | 2 | 0 | 0 | 0 |
|  | 6.50-6.99 | 2 | 4 | 2 | 1 | 0 | 1 | 1 |
|  | 7.00-7.49 | 2 | 2 | 2 | 1 | 1 | 0 | 0 |
|  | 7.50-7.99 | 0 | 1 | 2 | 0 | 0 | 1 | 2 |
|  | 8.00-8.49 | 1 | 0 | 1 | 1 | 0 | 0 | 0 |
|  | 8.50-8.99 | 1 | 1 | 2 | 0 | 0 | 0 | 0 |
|  | 9.00-9.49 | 0 | 0 | 0 | 0 | 0 | 0 | 0 |
|  | 9.50-9.99 | 0 | 0 | 0 | 0 | 0 | 0 | 0 |
|  | 10.00-10.49 | 0 | 0 | 0 | 0 | 0 | 0 | 0 |
| **Total** |  |  |  |  |  |  |  |  |
| **Short reliable** | 0.00-2.99 | 30 | 32 | 34 | 34 | 34 | 34 | 34 |
| **Long reliable** | 3.00-4.49 | 27 | 36 | 38 | 31 | 34 | 36 | 41 |
| **At the NOE limit** | 4.50-5.49 | 6 | 12 | 9 | 13 | 13 | 13 | 20 |
| **Beyond the NOE limit** | 5.50-9.99 | 9 | 13 | 14 | 11 | 2 | 5 | 5 |
| **All** | 0.00-10.49 | 72 | 93 | 95 | 89 | 83 | 87 | 100 |

**Table S28**. Average absolute error (%) on the calculated distances for which the correlations were quantified based on the normalised intensity (single spectrum) for which the underlaying cross-peaks have the same phase with the NOESY pulse program noesyph for each of the 7 mixing times. Correlations for which distances were calculated to be 5.50 Å or larger were excluded.

|  | **Distance range (Å)** | **100** | **200** | **300** | **400** | **500** | **600** | **700** |
| --- | --- | --- | --- | --- | --- | --- | --- | --- |
| **Short reliable** | 0.00-1.99 | 22.4 | 15.3 | 4.0 | 3.8 | 4.2 | 3.8 | 3.5 |
|  | 2.00-2.49 | 20.0 | 7.2 | 5.4 | 5.0 | 4.4 | 3.9 | 3.5 |
|  | 2.50-2.99 | 29.2 | 5.9 | 4.7 | 3.7 | 4.0 | 4.8 | 5.6 |
| **Long reliable** | 3.00-3.49 | 25.1 | 12.2 | 8.8 | 9.4 | 16.1 | 9.5 | 7.2 |
|  | 3.50-3.99 | 25.8 | 10.3 | 14.0 | 8.8 | 8.4 | 9.6 | 11.4 |
|  | 4.00-4.49 | 13.4 | 14.2 | 11.7 | 13.3 | 12.0 | 13.6 | 12.4 |
| **At the NOE limit** | 4.50-4.99 | 5.1 | 3.5 | 4.5 | 8.4 | 5.5 | 5.3 | 5.9 |
|  | 5.00-5.49 | 9.6 | 6.5 | 3.0 | 7.3 | 4.7 | 5.3 | 3.5 |
| **Beyond the NOE limit** | 5.50-5.99 | 6.8 | 10.1 | 17.9 | 12.5 | 3.8 | 17.2 | 25.1 |
|  | 6.00-6.49 | 13.9 | 11.4 | 13.1 | 14.5 | - | - | - |
|  | 6.50-6.99 | 34.0 | 26.3 | 29.4 | 17.3 | - | 15.4 | 23.1 |
|  | 7.00-7.49 | 30.2 | 29.8 | 26.4 | 26.4 | 26.6 | - | - |
|  | 7.50-7.99 | - | 57.0 | 44.3 | - | - | 53.1 | 43.7 |
|  | 8.00-8.49 | 37.6 | - | 36.1 | 31.8 | - | - | - |
|  | 8.50-8.99 | 40.2 | 41.4 | 41.3 | - | - | - | - |
|  | 9.00-9.49 | - | - | - | - | - | - | - |
|  | 9.50-9.99 | - | - | - | - | - | - | - |
|  | 10.00-10.49 | - | - | - | - | - | - | - |
| **Total** |  |  |  |  |  |  |  |  |
| **Short reliable** | 0.00-2.99 | 23.3 | 8.1 | 5.0 | 4.5 | 4.3 | 4.1 | 4.1 |
| **Long reliable** | 3.00-4.49 | 22.3 | 11.7 | 12.4 | 10.2 | 11.2 | 10.7 | 11.0 |
| **At the NOE limit** | 4.50-5.49 | 6.6 | 4.2 | 4.2 | 8.1 | 5.3 | 5.3 | 5.4 |
| **Beyond the NOE limit** | 5.50-9.99 | 26.0 | 24.2 | 28.8 | 16.3 | 15.2 | 24.0 | 32.2 |
| **All** | 0.00-10.49 | 21.8 | 11.3 | 11.5 | 8.4 | 7.5 | 8.1 | 8.6 |

*Key findings*

Distances derived from a single NOESY spectrum recorded at a given mixing times led to distances with larger errors than acceptable for structure determination. Distances derived from spectra recorded with 100 or 200 ms mixing times resulted in average absolute errors between 10 and 45% independent of the different distance ranges. For data derived from spectra recorded with 300-700 ms mixing time, distances up to 3.00 Å had average absolute errors below 10%, whereas distance beyond 3.00 Å, which are most informative for structural analysis, showed errors ≥10%.

Following an (semi) automated protocol, without any quality checks results in place, a large number of correlations (roughly 40% of the total number of correlations) well beyond the NOE-limit, resulting in the inclusion of artificial distance constraints. Performing a structural analysis with data of which about half is artefacts is ultimately condemned to fail.

The recommended quality checks of removing correlations for which the cross-peaks show the opposite phase and excluding distances that are calculated to be beyond 5.50 Å significantly reduced the number of false correlations that are quantified, however, the accuracy of the distances determined from the NOE cross-peaks remains unsuitably low for structure determination.

**Table S29**. Quantified distances based on the normalised intensity of each of the spectra recorded at different mixing times (100-700 ms) for the NOESY sequence (noesyph). The theoretical population-averaged interproton distances as determined by Butts *et. al.*^[3]^ are given for each correlation, and used to calculate the absolute error (|%|). The proton-proton correlation used as a reference for distance calculation was proton-pair 15 and is highlighted by being underscored

|  |  |  |  | **100 ms** | | **200 ms** | | **300 ms** | | **400 ms** | | **500 ms** | | **600 ms** | | **700 ms** | |
| --- | --- | --- | --- | --- | --- | --- | --- | --- | --- | --- | --- | --- | --- | --- | --- | --- | --- |
|  | **H_a_** | **H_b_** | **r_ab_**  **Theor. (Å)** | **r_ab_ (Å)** | **Error (%)** | **r_ab_ (Å)** | **Error (%)** | **r_ab_ (Å)** | **Error (%)** | **r_ab_ (Å)** | **Error (%)** | **r_ab_ (Å)** | **Error (%)** | **r_ab_ (Å)** | **Error (%)** | **r_ab_ (Å)** | **Error (%)** |
| **0.00-1.99** | **20a** | **11b/20b** | 1.75 | 1.84 | 5.4 | 2.35 | 34.1 | 1.86 | 6.3 | 1.86 | 6.2 | 1.89 | 8.1 | 1.89 | 7.9 | 1.89 | 7.9 |
|  | **11a** | **11b/20b** | 1.76 | 2.46 | 40.0 | 2.16 | 22.6 | 1.95 | 11.0 | 1.91 | 8.5 | 1.91 | 8.8 | 1.90 | 8.0 | 1.89 | 7.6 |
|  | **15a** | **15b** | 1.76 | 1.76 | 0.0 | 1.76 | 0.0 | 1.76 | 0.0 | 1.76 | 0.0 | 1.76 | 0.0 | 1.76 | 0.0 | 1.76 | 0.0 |
|  | **17a** | **17b** | 1.77 | 2.24 | 26.4 | 1.84 | 4.1 | 1.79 | 0.9 | 1.75 | 1.1 | 1.79 | 1.0 | 1.78 | 0.5 | 1.77 | 0.2 |
|  | **18a** | **18b** | 1.77 | 2.48 | 40.0 | 2.05 | 15.9 | 1.80 | 1.7 | 1.83 | 3.2 | 1.82 | 3.0 | 1.82 | 2.7 | 1.80 | 1.9 |
| **2.00-2.49** | **20a** | **15a** | 2.24 | 2.77 | 23.8 | 2.21 | 1.2 | 2.18 | 2.6 | 2.14 | 4.3 | 2.17 | 3.1 | 2.16 | 3.4 | 2.16 | 3.8 |
|  | **15b** | **13** | 2.26 | 3.01 | 33.4 | 3.12 | 38.1 | 2.89 | 27.8 | 2.90 | 28.4 | 2.75 | 21.6 | 2.49 | 10.3 | 2.53 | 11.9 |
|  | **12** | **23ab** | 2.27 | 2.88 | 27.0 | 2.40 | 5.6 | 2.35 | 3.4 | 2.31 | 1.6 | 2.33 | 2.8 | 2.33 | 2.9 | 2.32 | 2.0 |
|  | **22** | **23ab** | 2.30 | 2.96 | 28.9 | 2.50 | 8.9 | 2.43 | 5.7 | 2.40 | 4.2 | 2.43 | 5.5 | 2.41 | 4.6 | 2.39 | 4.0 |
|  | **12** | **11a** | 2.35 | 5.29 | 125.0 | 2.22 | 5.4 | 2.24 | 4.6 | 2.31 | 1.8 | 2.36 | 0.3 | 2.33 | 0.9 | 2.32 | 1.5 |
|  | **12** | **13** | 2.35 | 2.86 | 21.5 | 2.45 | 4.2 | 2.36 | 0.3 | 2.33 | 0.9 | 2.36 | 0.3 | 2.34 | 0.2 | 2.33 | 0.7 |
|  | **8** | **18b** | 2.37 | 2.77 | 17.1 | 2.32 | 2.2 | 2.26 | 4.7 | 2.22 | 6.2 | 2.25 | 5.0 | 2.24 | 5.6 | 2.23 | 6.0 |
|  | **1** | **16** | 2.38 | 2.83 | 18.8 | 2.36 | 1.0 | 2.29 | 3.7 | 2.25 | 5.3 | 2.28 | 4.1 | 2.27 | 4.6 | 2.26 | 4.9 |
|  | **22** | **11b/20b** | 2.39 | 3.12 | 30.7 | 2.60 | 8.9 | 2.54 | 6.2 | 2.51 | 4.9 | 2.54 | 6.4 | 2.53 | 5.8 | 2.52 | 5.5 |
|  | **14** | **13** | 2.41 | 2.93 | 21.5 | 2.52 | 4.6 | 2.44 | 1.4 | 2.40 | 0.4 | 2.43 | 0.9 | 2.42 | 0.3 | 2.41 | 0.1 |
|  | **8** | **17a** | 2.42 | 2.93 | 21.0 | 2.44 | 0.9 | 2.39 | 1.2 | 2.34 | 3.3 | 2.38 | 1.8 | 2.36 | 2.5 | 2.35 | 2.8 |
|  | **18a** | **17b** | 2.43 | 2.83 | 16.3 | 2.47 | 1.7 | 2.49 | 2.5 | 2.38 | 1.9 | 2.41 | 0.8 | 2.41 | 1.0 | 2.40 | 1.2 |
|  | **18b** | **17a** | 2.43 | 2.81 | 15.5 | 3.19 | 31.2 | 2.39 | 1.7 | 2.46 | 1.3 | 2.46 | 1.1 | 2.45 | 0.7 | 2.44 | 0.6 |
|  | **18b** | **11b/20b** | 2.45 | 3.24 | 32.4 | 3.24 | 32.3 | 2.82 | 15.0 | 2.69 | 10.0 | 2.72 | 10.9 | 2.78 | 13.5 | 2.80 | 14.2 |
|  | **3** | **2** | 2.48 | 2.90 | 16.8 | 3.11 | 25.3 | 2.33 | 6.0 | 2.75 | 11.0 | 2.58 | 4.0 | 2.75 | 11.0 | 2.44 | 1.7 |
|  | **16** | **15a** | 2.48 | 2.86 | 15.3 | 2.60 | 4.8 | 2.40 | 3.3 | 2.42 | 2.4 | 2.46 | 0.9 | 2.45 | 1.0 | 2.46 | 1.0 |
|  | **16** | **15b** | 2.48 | 4.19 | 68.8 | 2.58 | 4.1 | 2.43 | 2.1 | 2.39 | 3.7 | 2.44 | 1.6 | 2.44 | 1.8 | 2.44 | 1.6 |
|  | **14** | **15a** | 2.48 | 2.96 | 19.2 | 2.55 | 2.9 | 2.48 | 0.1 | 2.42 | 2.2 | 2.46 | 0.6 | 2.45 | 1.1 | 2.45 | 1.2 |
|  | **1** | **2** | 2.49 | 2.66 | 6.8 | 2.27 | 8.9 | 2.73 | 9.8 | 2.45 | 1.6 | 2.80 | 12.6 | 2.43 | 2.5 | 2.44 | 1.9 |
| **2.50-2.99** | **4** | **3** | 2.50 | 2.60 | 3.8 | 2.20 | 12.1 | 3.81 | 52.5 | 2.50 | 0.2 | 2.49 | 0.4 | 2.47 | 1.0 | 2.48 | 0.9 |
|  | **14** | **15b** | 2.55 | 3.58 | 40.5 | 2.51 | 1.7 | 2.49 | 2.2 | 2.47 | 3.2 | 2.53 | 0.8 | 2.53 | 0.7 | 2.54 | 0.4 |
|  | **23ab** | **14** | 2.59 | 3.18 | 22.7 | 2.67 | 3.0 | 2.59 | 0.2 | 2.55 | 1.5 | 2.59 | 0.1 | 2.56 | 1.0 | 2.56 | 1.3 |
|  | **8** | **11b/20b** | 2.63 | 3.43 | 30.3 | 2.76 | 4.8 | 2.70 | 2.6 | 2.65 | 0.7 | 2.69 | 2.1 | 2.66 | 1.2 | 2.66 | 1.0 |
|  | **16** | **17b** | 2.71 | 3.24 | 19.4 | 2.70 | 0.2 | 2.64 | 2.6 | 2.60 | 4.0 | 2.63 | 2.8 | 2.62 | 3.3 | 2.61 | 3.6 |
|  | **18a** | **17a** | 2.73 | 4.58 | 67.7 | 3.37 | 23.4 | 2.63 | 3.8 | 2.88 | 5.4 | 2.89 | 5.9 | 3.00 | 10.1 | 3.12 | 14.4 |
|  | **12** | **14** | 2.74 | 3.26 | 19.0 | 2.79 | 1.8 | 2.71 | 1.0 | 2.67 | 2.5 | 2.71 | 0.9 | 2.70 | 1.4 | 2.70 | 1.5 |
|  | **1** | **17b** | 2.79 | 3.39 | 21.4 | 2.82 | 1.0 | 2.75 | 1.3 | 2.70 | 3.3 | 2.75 | 1.6 | 2.72 | 2.3 | 2.72 | 2.3 |
|  | **18a** | **11b/20b** | 2.82 | 3.88 | 37.5 | 2.94 | 4.2 | 3.07 | 8.8 | 3.06 | 8.5 | 3.18 | 12.7 | 3.16 | 12.2 | 3.16 | 11.9 |
|  | **12** | **11b/20b** | 2.93 | 5.82 | 98.5 | 3.14 | 7.3 | 3.52 | 20.3 | 3.17 | 8.0 | 3.31 | 13.0 | 3.37 | 15.1 | 3.47 | 18.4 |
| **3.00-3.49** | **8** | **13** | 3.01 | 3.81 | 26.6 | 2.80 | 6.9 | 2.89 | 4.0 | 3.00 | 0.5 | 3.05 | 1.5 | 3.00 | 0.2 | 3.00 | 0.3 |
|  | **18b** | **17b** | 3.06 | 3.03 | 0.9 | 4.71 | 53.8 | 3.58 | 17.0 | 4.30 | 40.5 | 4.66 | 52.3 | 3.87 | 26.4 | 3.72 | 21.6 |
|  | **22** | **18b** | 3.16 | 3.73 | 18.0 | 3.10 | 1.9 | 3.00 | 5.2 | 2.97 | 5.9 | 3.02 | 4.4 | 3.00 | 4.9 | 2.99 | 5.4 |
|  | **23ab** | **11b/20b** | 3.24 | 4.66 | 43.8 | 3.61 | 11.3 | 3.53 | 9.1 | 3.50 | 8.1 | 3.66 | 13.1 | 3.57 | 10.2 | 3.63 | 12.1 |
|  | **20a** | **14** | 3.36 | 4.33 | 28.8 | 3.42 | 1.7 | 3.40 | 1.1 | 3.33 | 0.9 | 3.38 | 0.7 | 3.41 | 1.5 | 3.40 | 1.2 |
|  | **16** | **20a** | 3.37 | 3.74 | 11.1 | 3.45 | 2.3 | 3.59 | 6.5 | 3.40 | 0.8 | 3.39 | 0.5 | 3.49 | 3.6 | 3.42 | 1.4 |
|  | **22** | **20a** | 3.41 | 5.00 | 46.6 | 3.65 | 7.1 | 4.05 | 18.7 | 7.72 | 126.5 | 4.79 | 40.5 | 4.08 | 19.6 | 3.71 | 8.7 |
| **3.50-3.99** | **15a** | **13** | 3.54 | 2.61 | 26.3 | 3.61 | 2.1 | 5.41 | 52.9 | 4.09 | 15.4 | 3.43 | 3.2 | 3.25 | 8.3 | 3.16 | 10.6 |
|  | **20a** | **18b** | 3.60 | 3.02 | 16.1 | 3.04 | 15.6 | 4.77 | 32.6 | 4.10 | 14.0 | 4.13 | 14.6 | 4.05 | 12.6 | 3.73 | 3.6 |
|  | **8** | **17b** | 3.61 | 4.50 | 24.6 | 4.01 | 11.0 | 4.16 | 15.1 | 5.08 | 40.6 | 5.73 | 58.6 | 4.26 | 17.9 | 3.96 | 9.8 |
|  | **11b/20b** | **13** | 3.61 | 6.94 | 92.4 | 4.60 | 27.4 | 4.32 | 19.6 | 4.20 | 16.4 | 4.29 | 18.9 | 4.29 | 18.9 | 4.35 | 20.6 |
|  | **16** | **18a** | 3.62 | 4.43 | 22.3 | 3.74 | 3.2 | 3.70 | 2.3 | 3.65 | 0.7 | 3.71 | 2.4 | 3.68 | 1.7 | 3.72 | 2.8 |
|  | **12** | **8** | 3.65 | 5.20 | 42.4 | 3.73 | 2.3 | 3.74 | 2.4 | 3.68 | 0.8 | 3.72 | 2.0 | 3.79 | 3.8 | 3.72 | 1.9 |
|  | **11a** | **13** | 3.66 | 4.75 | 29.7 | 3.76 | 2.7 | 3.62 | 1.1 | 3.62 | 1.0 | 3.62 | 1.1 | 3.64 | 0.6 | 3.68 | 0.6 |
|  | **11b/20b** | **15a** | 3.69 | 4.03 | 9.3 | 4.43 | 20.0 | 3.67 | 0.5 | 3.46 | 6.2 | 3.38 | 8.3 | 3.20 | 13.3 | 3.14 | 14.9 |
|  | **23ab** | **8** | 3.73 | 4.24 | 13.8 | 4.12 | 10.4 | 5.27 | 41.3 | 4.67 | 25.1 | 3.90 | 4.5 | 4.11 | 10.1 | 3.88 | 4.1 |
|  | **20a** | **15b** | 3.75 | 6.40 | 70.8 | 4.46 | 19.0 | 3.62 | 3.4 | 3.26 | 13.0 | 3.20 | 14.6 | 3.09 | 17.7 | 3.00 | 20.0 |
|  | **23ab** | **13** | 3.76 | 5.43 | 44.3 | 6.78 | 80.4 | 5.23 | 39.0 | 6.23 | 65.6 | 6.25 | 66.1 | 4.62 | 22.8 | 4.44 | 18.0 |
|  | **20a** | **18a** | 3.81 | 4.53 | 18.8 | 4.27 | 12.0 | 4.43 | 16.2 | 4.59 | 20.5 | 4.52 | 18.7 | 4.67 | 22.5 | 5.37 | 41.1 |
|  | **8** | **18a** | 3.83 | 5.38 | 40.5 | 4.52 | 18.1 | 4.01 | 4.6 | 3.63 | 5.2 | 3.49 | 8.8 | 3.32 | 13.3 | 3.21 | 16.2 |
|  | **16** | **17a** | 3.84 | 4.76 | 23.9 | 4.13 | 7.6 | 4.74 | 23.6 | 4.58 | 19.2 | 5.83 | 51.7 | 5.06 | 31.7 | 4.41 | 15.0 |
|  | **1** | **15b** | 3.86 | 4.97 | 28.6 | 3.81 | 1.3 | 3.95 | 2.5 | 3.84 | 0.5 | 4.10 | 6.2 | 3.97 | 3.0 | 4.26 | 10.3 |
|  | **22** | **14** | 3.86 | 4.73 | 22.5 | 4.08 | 5.8 | 3.86 | 0.1 | 3.86 | 0.0 | 3.91 | 1.2 | 3.91 | 1.3 | 3.97 | 2.7 |
|  | **8** | **11a** | 3.88 | 5.37 | 38.3 | 4.42 | 13.9 | 4.66 | 20.1 | 5.31 | 36.9 | 5.58 | 43.8 | 4.54 | 17.0 | 4.13 | 6.4 |
|  | **16** | **18b** | 3.89 | 4.51 | 15.9 | 3.91 | 0.6 | 4.05 | 4.0 | 3.94 | 1.3 | 3.96 | 1.7 | 4.11 | 5.7 | 4.17 | 7.3 |
|  | **22** | **8** | 3.96 | 4.72 | 19.2 | 4.01 | 1.4 | 3.95 | 0.2 | 3.90 | 1.4 | 3.86 | 2.5 | 3.87 | 2.2 | 3.89 | 1.8 |
|  | **23ab** | **11a** | 3.96 | 5.40 | 36.3 | 4.97 | 25.5 | 4.69 | 18.4 | 5.60 | 41.3 | 4.83 | 21.9 | 4.31 | 8.8 | 4.09 | 3.4 |
|  | **8** | **14** | 3.96 | 6.72 | 69.8 | 4.44 | 12.2 | 4.22 | 6.6 | 3.95 | 0.2 | 4.06 | 2.6 | 4.07 | 2.9 | 4.09 | 3.3 |
|  | **14** | **11b/20b** | 3.96 | 4.64 | 17.1 | 4.81 | 21.4 | 4.78 | 20.7 | 5.41 | 36.6 | 5.34 | 34.8 | 5.25 | 32.5 | 5.44 | 37.3 |
| **4.00-4.49** | **1** | **17a** | 4.06 | 5.02 | 23.7 | 4.39 | 8.1 | 5.09 | 25.5 | 4.85 | 19.4 | 6.32 | 55.7 | 5.35 | 31.8 | 4.60 | 13.4 |
|  | **16** | **13** | 4.06 | 3.71 | 8.6 | 3.84 | 5.4 | 4.13 | 1.6 | 4.46 | 9.9 | 4.37 | 7.7 | 6.82 | 68.0 | 5.16 | 27.1 |
|  | **16** | **11b/20b** | 4.07 | 5.53 | 35.9 | 4.71 | 15.8 | 4.59 | 12.9 | 5.04 | 23.8 | 4.79 | 17.7 | 4.88 | 19.9 | 5.60 | 37.6 |
|  | **8** | **15b** | 4.09 | 4.76 | 16.4 | 3.31 | 19.0 | 5.01 | 22.6 | 4.29 | 5.0 | 3.95 | 3.3 | 5.03 | 23.1 | 4.27 | 4.5 |
|  | **16** | **8** | 4.14 | 3.51 | 15.2 | 2.97 | 28.3 | 4.98 | 20.4 | 3.19 | 23.0 | 3.27 | 21.1 | 3.61 | 12.8 | 3.47 | 16.2 |
|  | **11b/20b** | **17a** | 4.17 | 5.21 | 25.0 | 4.65 | 11.5 | 6.50 | 55.8 | 6.63 | 58.9 | 5.09 | 22.0 | 4.71 | 13.0 | 4.55 | 9.0 |
|  | **12** | **15b** | 4.27 | 6.93 | 62.2 | 4.11 | 3.6 | 4.51 | 5.6 | 5.07 | 18.7 | 5.58 | 30.7 | 5.13 | 20.2 | 4.28 | 0.3 |
|  | **23ab** | **18b** | 4.27 | 6.16 | 44.1 | 5.05 | 18.3 | 4.51 | 5.6 | 5.06 | 18.6 | 5.17 | 21.1 | 4.88 | 14.3 | 5.30 | 24.1 |
|  | **1** | **3** | 4.30 | 3.77 | 12.3 | 2.96 | 31.1 | 4.26 | 1.0 | 4.00 | 6.9 | 3.34 | 22.2 | 4.10 | 4.7 | 3.60 | 16.3 |
|  | **16** | **14** | 4.30 | 5.72 | 33.1 | 4.57 | 6.3 | 4.58 | 6.5 | 5.21 | 21.1 | 5.18 | 20.6 | 8.12 | 88.9 | 5.80 | 34.9 |
|  | **4** | **2** | 4.32 | 3.92 | 9.2 | 3.26 | 24.5 | 3.70 | 14.5 | 5.12 | 18.4 | 4.23 | 2.0 | 4.80 | 11.1 | 4.79 | 10.9 |
|  | **4** | **13** | 4.43 | 6.01 | 35.8 | 4.60 | 3.9 | 4.71 | 6.4 | 4.34 | 2.0 | 4.47 | 0.9 | 4.40 | 0.8 | 4.42 | 0.2 |
|  | **23ab** | **20a** | 4.44 | 4.67 | 5.1 | 4.31 | 2.9 | 5.67 | 27.8 | 4.96 | 11.6 | 4.70 | 5.9 | 5.69 | 28.1 | 5.40 | 21.5 |
|  | **22** | **18a** | 4.47 | 5.05 | 13.0 | 5.56 | 24.3 | 6.03 | 34.8 | 6.48 | 44.9 | 5.22 | 16.8 | 4.86 | 8.8 | 4.70 | 5.2 |
|  | **11b/20b** | **17b** | 4.49 | 4.86 | 8.1 | 5.05 | 12.5 | 5.50 | 22.4 | 6.19 | 38.0 | 5.06 | 12.8 | 5.41 | 20.5 | 5.22 | 16.2 |
| **4.50-4.99** | **18b** | **15a** | 4.55 | 4.21 | 7.4 | 3.89 | 14.5 | 4.43 | 2.6 | 4.37 | 4.0 | 4.79 | 5.4 | 4.71 | 3.4 | 6.14 | 34.9 |
|  | **11b/20b** | **15b** | 4.59 | 4.48 | 2.5 | 4.72 | 2.8 | 5.84 | 27.3 | 6.49 | 41.4 | 6.02 | 31.2 | 6.15 | 34.0 | 5.10 | 11.1 |
|  | **1** | **15a** | 4.62 | 7.11 | 53.9 | 5.26 | 13.9 | 6.11 | 32.3 | 5.38 | 16.3 | 4.92 | 6.4 | 4.61 | 0.2 | 4.22 | 8.6 |
|  | **8** | **15a** | 4.63 | 4.76 | 2.8 | 4.73 | 2.1 | 6.46 | 39.6 | 4.88 | 5.5 | 4.82 | 4.2 | 4.85 | 4.8 | 4.75 | 2.6 |
|  | **17b** | **15b** | 4.64 | 3.97 | 14.5 | 3.73 | 19.6 | 4.07 | 12.2 | 4.00 | 13.8 | 4.11 | 11.5 | 5.10 | 9.9 | 5.64 | 21.5 |
|  | **22** | **12** | 4.65 | 6.37 | 36.9 | 6.60 | 41.9 | 6.06 | 30.2 | 5.70 | 22.5 | 9.96 | 114.1 | 6.71 | 44.3 | 5.59 | 20.2 |
|  | **14** | **18b** | 4.66 | 4.73 | 1.4 | 4.80 | 3.0 | 4.54 | 2.6 | 6.82 | 46.4 | 4.40 | 5.5 | 4.63 | 0.6 | 4.11 | 11.8 |
|  | **23ab** | **15a** | 4.68 | 5.67 | 21.1 | 5.32 | 13.7 | 5.17 | 10.6 | 4.77 | 2.0 | 5.86 | 25.2 | 4.67 | 0.2 | 4.91 | 4.8 |
|  | **11a** | **17a** | 4.68 | 4.58 | 2.2 | 4.58 | 2.1 | 4.91 | 5.0 | 5.30 | 13.3 | 6.12 | 30.7 | 6.01 | 28.5 | 5.50 | 17.5 |
|  | **8** | **20a** | 4.71 | 4.24 | 10.0 | 3.60 | 23.5 | 4.14 | 12.2 | 4.13 | 12.4 | 4.86 | 3.3 | 4.06 | 13.7 | 4.79 | 1.6 |
|  | **18b** | **13** | 4.73 | 7.26 | 53.6 | 5.12 | 8.2 | 4.54 | 4.0 | 6.46 | 36.5 | 4.92 | 4.0 | 5.27 | 11.5 | 5.68 | 20.0 |
|  | **2** | **16** | 4.75 | 5.67 | 19.4 | 6.32 | 33.1 | 5.61 | 18.2 | 5.50 | 15.8 | 6.93 | 45.9 | 5.38 | 13.2 | 4.87 | 2.4 |
|  | **4** | **11a** | 4.77 | 6.16 | 29.1 | 4.81 | 0.8 | 4.79 | 0.3 | 4.95 | 3.8 | 4.72 | 1.0 | 4.94 | 3.5 | 4.60 | 3.6 |
|  | **11a** | **18b** | 4.77 | 3.40 | 28.7 | 3.34 | 29.9 | 3.86 | 19.1 | 3.68 | 22.8 | 3.77 | 21.1 | 4.04 | 15.3 | 5.44 | 14.1 |
|  | **17a** | **13** | 4.79 | 4.36 | 9.0 | 4.32 | 9.7 | 4.28 | 10.6 | 4.48 | 6.4 | 4.60 | 3.9 | 4.71 | 1.7 | 4.82 | 0.7 |
|  | **22** | **15a** | 4.81 | 6.39 | 32.8 | 5.47 | 13.7 | 5.57 | 15.7 | 5.43 | 12.8 | 5.46 | 13.5 | 5.34 | 11.1 | 4.94 | 2.8 |
|  | **23ab** | **15b** | 4.81 | 5.43 | 13.0 | 4.62 | 4.0 | 6.27 | 30.3 | 5.13 | 6.6 | 4.94 | 2.7 | 5.01 | 4.1 | 4.91 | 2.1 |
|  | **2** | **17b** | 4.82 | 6.25 | 29.7 | 5.25 | 8.9 | 5.30 | 9.9 | 5.81 | 20.6 | 5.41 | 12.2 | 7.45 | 54.5 | 5.87 | 21.7 |
|  | **18b** | **15b** | 4.83 | 4.70 | 2.6 | 5.07 | 5.0 | 5.24 | 8.5 | 5.25 | 8.7 | 6.48 | 34.2 | 5.39 | 11.6 | 6.69 | 38.6 |
|  | **14** | **11a** | 4.87 | 3.74 | 23.3 | 3.60 | 26.2 | 4.06 | 16.6 | 3.94 | 19.0 | 4.05 | 16.8 | 4.41 | 9.4 | 4.83 | 0.9 |
|  | **15a** | **17b** | 4.87 | 4.20 | 13.8 | 4.17 | 14.3 | 4.20 | 13.7 | 4.15 | 14.7 | 4.50 | 7.6 | 4.39 | 10.0 | 4.99 | 2.4 |
|  | **20a** | **13** | 4.88 | 8.53 | 74.8 | 4.88 | 0.0 | 5.45 | 11.6 | 5.64 | 15.5 | 6.18 | 26.7 | 6.12 | 25.3 | 6.19 | 26.9 |
|  | **4** | **8** | 4.89 | 5.35 | 9.4 | 4.86 | 0.7 | 4.77 | 2.5 | 4.71 | 3.6 | 4.95 | 1.2 | 4.78 | 2.3 | 4.88 | 0.3 |
|  | **4** | **11b/20b** | 4.89 | 4.51 | 7.8 | 5.92 | 21.1 | 5.39 | 10.3 | 5.33 | 9.0 | 5.53 | 13.1 | 6.08 | 24.4 | 5.63 | 15.2 |
|  | **18a** | **15a** | 4.89 | 5.62 | 15.0 | 4.57 | 6.5 | 5.00 | 2.2 | 5.75 | 17.7 | 5.26 | 7.5 | 6.26 | 27.9 | 4.65 | 5.0 |
|  | **1** | **18a** | 4.90 | 8.82 | 80.1 | 4.94 | 0.8 | 6.56 | 34.0 | 5.93 | 21.0 | 5.90 | 20.3 | 5.90 | 20.5 | 5.76 | 17.5 |
|  | **20a** | **17b** | 4.90 | 6.61 | 35.0 | 6.69 | 36.5 | 6.32 | 28.9 | 4.98 | 1.6 | 5.52 | 12.6 | 5.78 | 17.9 | 5.27 | 7.5 |
|  | **1** | **13** | 4.98 | 5.80 | 16.4 | 5.71 | 14.7 | 4.86 | 2.5 | 6.35 | 27.6 | 4.98 | 0.1 | 5.28 | 6.1 | 5.17 | 3.8 |
|  | **12** | **15a** | 4.98 | 7.14 | 43.5 | 6.86 | 37.7 | 5.61 | 12.7 | 5.65 | 13.4 | 5.96 | 19.6 | 5.23 | 5.0 | 5.27 | 5.8 |
| **5.00-5.49** | **4** | **1** | 5.00 | 4.30 | 14.1 | 4.29 | 14.2 | 4.27 | 14.7 | 5.15 | 3.1 | 4.90 | 2.0 | 9.67 | 93.3 | 5.14 | 2.8 |
|  | **17a** | **13** | 5.05 | 4.36 | 13.6 | 4.22 | 16.4 | 4.41 | 12.6 | 4.14 | 18.1 | 4.54 | 10.1 | 5.32 | 5.3 | 5.79 | 14.6 |
|  | **1** | **8** | 5.06 | 6.31 | 24.8 | 4.80 | 5.0 | 5.33 | 5.4 | 5.32 | 5.2 | 4.73 | 6.5 | 5.41 | 7.0 | 4.72 | 6.8 |
|  | **4** | **17a** | 5.17 | 5.55 | 7.4 | 5.44 | 5.3 | 5.20 | 0.6 | 4.75 | 8.2 | 5.03 | 2.8 | 5.35 | 3.6 | 4.96 | 4.0 |
|  | **17b** | **13** | 5.17 | 4.02 | 22.3 | 3.84 | 25.8 | 4.23 | 18.2 | 4.17 | 19.3 | 4.29 | 17.1 | 4.82 | 6.8 | 5.44 | 5.3 |
|  | **22** | **13** | 5.19 | 6.54 | 26.0 | 5.17 | 0.5 | 5.55 | 7.0 | 6.82 | 31.5 | 7.51 | 44.8 | 6.29 | 21.2 | 7.14 | 37.5 |
|  | **1** | **18b** | 5.32 | 5.97 | 12.3 | 5.79 | 8.9 | 5.94 | 11.6 | 6.43 | 20.9 | 8.01 | 50.6 | 6.74 | 26.7 | 7.21 | 35.5 |
|  | **20a** | **17a** | 5.35 | 6.05 | 13.0 | 5.99 | 12.0 | 6.77 | 26.6 | 6.54 | 22.3 | 5.55 | 3.7 | 5.60 | 4.7 | 5.32 | 0.5 |
|  | **12** | **18b** | 5.38 | 5.11 | 5.1 | 4.63 | 14.0 | 5.66 | 5.3 | 5.29 | 1.7 | 5.13 | 4.7 | 6.02 | 12.0 | 7.55 | 40.2 |
|  | **18a** | **15b** | 5.39 | 6.28 | 16.5 | 5.12 | 5.0 | 5.84 | 8.4 | 9.32 | 73.0 | 5.75 | 6.6 | 5.60 | 4.0 | 6.85 | 27.1 |
|  | **22** | **17a** | 5.41 | 6.57 | 21.4 | 5.33 | 1.5 | 5.68 | 4.9 | 5.67 | 4.7 | 6.27 | 16.0 | 5.66 | 4.7 | 5.57 | 2.9 |
|  | **2** | **15b** | 5.44 | 5.80 | 6.6 | 5.79 | 6.5 | 5.80 | 6.6 | 4.74 | 12.9 | 6.18 | 13.6 | 6.51 | 19.6 | 5.46 | 0.3 |
|  | **15a** | **17a** | 5.46 | 4.24 | 22.3 | 3.98 | 27.1 | 4.34 | 20.6 | 4.30 | 21.2 | 4.44 | 18.7 | 4.56 | 16.4 | 4.80 | 12.1 |
| **5.50-5.99** | **4** | **15b** | 5.53 | 5.35 | 3.3 | 5.08 | 8.2 | 5.28 | 4.6 | 5.43 | 1.8 | 6.28 | 13.6 | 6.83 | 23.5 | 7.63 | 38.0 |
|  | **22** | **15b** | 5.56 | 5.63 | 1.2 | 6.59 | 18.6 | 6.32 | 13.7 | 5.70 | 2.5 | 7.30 | 31.2 | 7.20 | 29.5 | 5.42 | 2.5 |
|  | **2** | **17a** | 5.67 | 6.93 | 22.2 | 5.61 | 1.0 | 5.74 | 1.2 | 5.26 | 7.3 | 7.16 | 26.2 | 6.30 | 11.1 | 6.31 | 11.4 |
|  | **11a** | **15b** | 5.69 | 5.60 | 1.6 | 4.83 | 15.2 | 4.95 | 13.0 | 4.73 | 16.9 | 5.95 | 4.5 | 6.53 | 14.7 | 6.31 | 11.0 |
|  | **4** | **17b** | 5.70 | 7.12 | 24.9 | 6.30 | 10.5 | 8.38 | 47.0 | 6.39 | 12.0 | 6.85 | 20.3 | 5.69 | 0.3 | 7.45 | 30.8 |
|  | **12** | **20a** | 5.70 | 4.93 | 13.5 | 5.11 | 10.3 | 4.81 | 15.6 | 4.89 | 14.2 | 5.69 | 0.1 | 6.23 | 9.3 | 6.15 | 7.9 |
|  | **23ab** | **17a** | 5.70 | 5.68 | 0.4 | 4.71 | 17.3 | 4.92 | 13.7 | 4.70 | 17.6 | 5.19 | 9.0 | 5.64 | 1.1 | 5.40 | 5.2 |
|  | **22** | **16** | 5.71 | 7.07 | 23.8 | 6.72 | 17.7 | 5.73 | 0.3 | 5.92 | 3.7 | 7.80 | 36.7 | 6.62 | 15.9 | 7.73 | 35.3 |
|  | **23ab** | **18a** | 5.72 | 5.30 | 7.3 | 5.22 | 8.7 | 6.13 | 7.1 | 5.32 | 7.0 | 5.50 | 3.8 | 5.03 | 12.1 | 5.32 | 7.0 |
|  | **1** | **20a** | 5.74 | 5.64 | 1.7 | 6.20 | 8.0 | 6.07 | 5.8 | 5.44 | 5.3 | 6.59 | 14.9 | 5.94 | 3.5 | 6.55 | 14.2 |
|  | **14** | **18a** | 5.79 | 3.38 | 41.6 | 3.24 | 44.0 | 4.08 | 29.5 | 4.10 | 29.2 | 3.63 | 37.3 | 4.02 | 30.6 | 3.18 | 45.0 |
|  | **14** | **17a** | 5.79 | 6.16 | 6.4 | 4.98 | 14.0 | 6.04 | 4.3 | 6.23 | 7.5 | 6.62 | 14.3 | 5.31 | 8.4 | 7.17 | 23.9 |
|  | **4** | **12** | 5.87 | 5.26 | 10.4 | 6.57 | 11.9 | 5.47 | 6.8 | 5.53 | 5.8 | 5.87 | 0.0 | 7.36 | 25.3 | 6.25 | 6.5 |
|  | **1** | **11b/20b** | 5.89 | 7.71 | 30.9 | 6.42 | 8.9 | 7.03 | 19.4 | 6.14 | 4.2 | 6.53 | 10.9 | 9.15 | 55.4 | 6.18 | 5.0 |
|  | **18a** | **13** | 5.89 | 5.64 | 4.2 | 5.68 | 3.6 | 5.01 | 14.9 | 5.15 | 12.6 | 5.65 | 4.0 | 4.96 | 15.9 | 4.95 | 16.0 |
|  | **3** | **13** | 5.90 | 6.40 | 8.5 | 5.08 | 13.9 | 5.99 | 1.5 | 6.49 | 10.1 | 6.78 | 15.0 | 5.54 | 6.1 | 5.92 | 0.3 |
|  | **23ab** | **16** | 5.93 | 4.01 | 32.4 | 3.80 | 35.9 | 4.10 | 30.8 | 4.32 | 27.1 | 4.28 | 27.9 | 4.09 | 31.1 | 4.71 | 20.6 |
|  | **3** | **17b** | 5.95 | 5.82 | 2.2 | 4.95 | 16.8 | 6.33 | 6.4 | 6.06 | 1.9 | 6.13 | 3.0 | 6.64 | 11.6 | 5.85 | 1.6 |
|  | **14** | **17b** | 5.95 | 6.07 | 2.0 | 5.45 | 8.4 | 4.92 | 17.3 | 6.62 | 11.3 | 6.89 | 15.8 | 6.76 | 13.7 | 6.42 | 7.9 |
| **6.00-6.49** | **12** | **17a** | 6.01 | 5.87 | 2.4 | 5.60 | 6.9 | 5.81 | 3.4 | 6.15 | 2.4 | 7.22 | 20.1 | 5.44 | 9.4 | 6.05 | 0.7 |
|  | **22** | **17b** | 6.03 | 7.02 | 16.5 | 5.92 | 1.8 | 5.68 | 5.9 | 5.64 | 6.5 | 7.30 | 21.1 | 6.80 | 12.8 | 6.22 | 3.2 |
|  | **12** | **16** | 6.03 | 4.47 | 25.8 | 4.26 | 29.3 | 4.87 | 19.2 | 4.70 | 22.1 | 4.90 | 18.7 | 5.48 | 9.1 | 6.02 | 0.1 |
|  | **4** | **16** | 6.07 | 5.23 | 13.9 | 5.38 | 11.4 | 6.50 | 7.0 | 5.44 | 10.4 | 5.77 | 5.0 | 7.95 | 31.1 | 6.71 | 10.5 |
|  | **3** | **17a** | 6.09 | 6.16 | 1.2 | 6.37 | 4.6 | 5.95 | 2.2 | 6.96 | 14.2 | 7.09 | 16.4 | 8.04 | 32.0 | 5.48 | 9.9 |
|  | **3** | **15b** | 6.11 | 8.70 | 42.4 | 6.84 | 12.0 | 5.61 | 8.2 | 6.07 | 0.7 | 5.91 | 3.2 | 5.49 | 10.1 | 5.80 | 5.0 |
|  | **2** | **13** | 6.11 | 6.29 | 2.9 | 5.51 | 9.9 | 6.94 | 13.6 | 8.29 | 35.7 | 5.96 | 2.4 | 6.37 | 4.2 | 7.25 | 18.7 |
|  | **11a** | **17b** | 6.11 | 4.94 | 19.2 | 4.42 | 27.7 | 4.78 | 21.8 | 4.97 | 18.7 | 5.98 | 2.1 | 5.83 | 4.6 | 5.51 | 9.8 |
|  | **1** | **14** | 6.12 | 6.04 | 1.3 | 5.25 | 14.2 | 7.22 | 18.0 | 5.84 | 4.6 | 6.31 | 3.1 | 7.00 | 14.4 | 6.33 | 3.4 |
|  | **22** | **11a** | 6.14 | 5.54 | 9.8 | 5.56 | 9.4 | 6.33 | 3.1 | 5.72 | 6.9 | 7.01 | 14.1 | 5.90 | 3.9 | 7.12 | 16.0 |
|  | **3** | **16** | 6.16 | 5.80 | 5.8 | 5.72 | 7.1 | 5.36 | 13.1 | 8.51 | 38.1 | 6.03 | 2.0 | 6.29 | 2.1 | 5.56 | 9.7 |
| **6.50-6.99** | **23ab** | **17b** | 6.50 | 5.51 | 15.3 | 5.21 | 19.8 | 5.35 | 17.7 | 5.37 | 17.3 | 4.96 | 23.6 | 5.50 | 15.4 | 5.71 | 12.1 |
|  | **3** | **8** | 6.53 | 6.30 | 3.6 | 5.69 | 12.8 | 5.84 | 10.6 | 6.24 | 4.4 | 7.62 | 16.7 | 10.07 | 54.2 | 5.70 | 12.7 |
|  | **11a** | **15a** | 6.53 | 4.46 | 31.7 | 4.14 | 36.7 | 4.61 | 29.5 | 5.27 | 19.2 | 5.60 | 14.3 | 7.07 | 8.2 | 8.46 | 29.6 |
|  | **2** | **8** | 6.59 | 7.88 | 19.5 | 7.16 | 8.7 | 6.86 | 4.1 | 5.95 | 9.7 | 6.90 | 4.6 | 8.61 | 30.6 | 6.83 | 3.7 |
|  | **2** | **15a** | 6.64 | 6.66 | 0.3 | 5.92 | 10.8 | 5.51 | 17.1 | 6.06 | 8.8 | 7.27 | 9.5 | 6.41 | 3.4 | 6.81 | 2.6 |
|  | **4** | **14** | 6.82 | 6.15 | 9.8 | 6.03 | 11.6 | 5.51 | 19.2 | 5.83 | 14.6 | 6.04 | 11.4 | 6.60 | 3.2 | 8.86 | 30.0 |
|  | **12** | **17b** | 6.83 | 5.43 | 20.5 | 5.36 | 21.5 | 5.45 | 20.2 | 6.14 | 10.2 | 7.05 | 3.2 | 6.08 | 11.0 | 6.53 | 4.3 |
|  | **4** | **18b** | 6.88 | 4.97 | 27.8 | 5.49 | 20.2 | 5.76 | 16.3 | 6.49 | 5.7 | 6.11 | 11.2 | 6.99 | 1.6 | 6.17 | 10.4 |
|  | **12** | **18a** | 6.94 | 4.15 | 40.2 | 3.89 | 43.9 | 4.26 | 38.7 | 4.70 | 32.3 | 4.68 | 32.6 | 5.00 | 28.0 | 5.33 | 23.1 |
| **7.00-7.49** | **3** | **11b/20b** | 7.02 | 6.48 | 7.7 | 5.28 | 24.8 | 5.73 | 18.4 | 5.16 | 26.4 | 7.31 | 4.2 | 6.94 | 1.2 | 5.76 | 18.0 |
|  | **16** | **11a** | 7.08 | 5.11 | 27.9 | 5.65 | 20.2 | 5.30 | 25.1 | 6.44 | 9.0 | 5.80 | 18.1 | 5.34 | 24.6 | 6.56 | 7.3 |
|  | **4** | **15a** | 7.13 | 5.67 | 20.4 | 6.77 | 5.0 | 5.76 | 19.3 | 7.28 | 2.0 | 5.98 | 16.1 | 6.67 | 6.4 | 6.90 | 3.2 |
|  | **3** | **11a** | 7.13 | 5.49 | 23.1 | 5.91 | 17.1 | 5.16 | 27.6 | 7.96 | 11.6 | 7.03 | 1.4 | 8.26 | 15.9 | 6.31 | 11.6 |
|  | **2** | **18a** | 7.16 | 5.39 | 24.8 | 6.16 | 13.9 | 6.61 | 7.7 | 6.40 | 10.6 | 6.80 | 5.1 | 5.86 | 18.1 | 6.21 | 13.3 |
|  | **4** | **23ab** | 7.21 | 5.43 | 24.7 | 6.18 | 14.2 | 5.78 | 19.8 | 6.36 | 11.8 | 5.84 | 19.0 | 5.98 | 17.0 | 5.73 | 20.6 |
|  | **1** | **12** | 7.22 | 6.65 | 8.0 | 6.47 | 10.4 | 6.99 | 3.2 | 6.13 | 15.0 | 5.89 | 18.4 | 7.40 | 2.5 | 6.96 | 3.7 |
|  | **2** | **18b** | 7.42 | 6.15 | 17.2 | 5.67 | 23.6 | 5.55 | 25.2 | 5.51 | 25.7 | 6.38 | 14.0 | 6.56 | 11.7 | 7.11 | 4.2 |
|  | **2** | **11b/20b** | 7.43 | 6.60 | 11.2 | 4.84 | 34.8 | 7.83 | 5.3 | 7.50 | 0.9 | 5.45 | 26.6 | 7.65 | 3.0 | 5.72 | 23.0 |
|  | **20a** | **11a** | 7.48 | 4.81 | 35.7 | 4.70 | 37.2 | 5.60 | 25.2 | 4.74 | 36.7 | 4.62 | 38.2 | 4.70 | 37.1 | 5.44 | 27.3 |
| **7.50-7.99** | **1** | **23ab** | 7.50 | 7.70 | 2.7 | 6.10 | 18.7 | 7.40 | 1.3 | 6.47 | 13.7 | 5.71 | 23.8 | 6.72 | 10.5 | 5.45 | 27.4 |
|  | **4** | **18a** | 7.60 | 6.53 | 14.0 | 5.57 | 26.7 | 7.38 | 2.9 | 6.77 | 10.9 | 6.26 | 17.7 | 6.63 | 12.8 | 6.03 | 20.6 |
|  | **1** | **11a** | 7.64 | 6.11 | 20.1 | 6.02 | 21.1 | 5.64 | 26.2 | 8.22 | 7.6 | 5.60 | 26.7 | 6.02 | 21.2 | 6.12 | 19.9 |
|  | **3** | **15a** | 7.67 | 6.60 | 13.9 | 7.07 | 7.8 | 5.88 | 23.3 | 5.77 | 24.8 | 6.30 | 17.9 | 6.10 | 20.5 | 6.45 | 15.9 |
|  | **18a** | **11a** | 7.69 | 3.81 | 50.4 | 3.31 | 57.0 | 3.36 | 56.3 | 3.45 | 55.2 | 3.57 | 53.6 | 3.60 | 53.1 | 3.07 | 60.1 |
|  | **1** | **22** | 7.71 | 6.58 | 14.7 | 6.73 | 12.7 | 5.21 | 32.4 | 6.13 | 20.5 | 7.32 | 5.1 | 6.13 | 20.5 | 7.48 | 3.0 |
|  | **2** | **14** | 7.80 | 6.29 | 19.4 | 6.06 | 22.4 | 5.67 | 27.3 | 6.79 | 13.0 | 8.64 | 10.8 | 6.93 | 11.1 | 6.71 | 13.9 |
|  | **3** | **12** | 7.83 | 6.16 | 21.4 | 6.33 | 19.2 | 6.76 | 13.7 | 6.74 | 14.0 | 7.37 | 5.9 | 7.07 | 9.7 | 6.27 | 20.0 |
| **8.00-8.49** | **3** | **18b** | 8.03 | 6.09 | 24.1 | 6.09 | 24.2 | 5.68 | 29.2 | 5.48 | 31.8 | 6.46 | 19.6 | 6.70 | 16.5 | 5.98 | 25.6 |
|  | **3** | **14** | 8.08 | 9.23 | 14.2 | 6.36 | 21.3 | 5.71 | 29.4 | 6.17 | 23.6 | 7.18 | 11.2 | 5.77 | 28.6 | 8.27 | 2.3 |
|  | **2** | **20a** | 8.09 | 6.12 | 24.4 | 5.62 | 30.5 | 5.93 | 26.7 | 6.22 | 23.1 | 6.85 | 15.4 | 6.48 | 20.0 | 5.76 | 28.8 |
|  | **3** | **18a** | 8.25 | 6.13 | 25.6 | 5.03 | 39.0 | 6.56 | 20.5 | 5.58 | 32.3 | 5.69 | 31.0 | 5.79 | 29.8 | 6.99 | 15.2 |
|  | **2** | **11a** | 8.28 | 5.17 | 37.6 | 5.73 | 30.9 | 7.18 | 13.3 | 6.71 | 18.9 | 8.58 | 3.6 | 6.26 | 24.4 | 6.32 | 23.6 |
|  | **2** | **12** | 8.37 | 6.26 | 25.2 | 5.37 | 35.9 | 5.35 | 36.1 | 8.54 | 2.1 | 9.11 | 8.8 | 6.40 | 23.5 | 6.83 | 18.4 |
|  | **4** | **20a** | 8.46 | 7.09 | 16.2 | 7.35 | 13.1 | 5.47 | 35.4 | 5.03 | 40.6 | 6.35 | 24.9 | 6.26 | 26.0 | 7.01 | 17.2 |
| **8.50-8.99** | **4** | **22** | 8.70 | 13.66 | 57.1 | 6.04 | 30.6 | 5.09 | 41.5 | 6.08 | 30.1 | 8.04 | 7.6 | 6.84 | 21.3 | 11.20 | 28.8 |
|  | **3** | **23ab** | 8.88 | 5.31 | 40.2 | 5.20 | 41.4 | 7.69 | 13.4 | 5.92 | 33.3 | 6.59 | 25.8 | 6.26 | 29.5 | 5.39 | 39.3 |
|  | **2** | **23ab** | 8.99 | 5.88 | 34.6 | 5.55 | 38.3 | 5.28 | 41.2 | 5.93 | 34.1 | 6.65 | 26.1 | 5.89 | 34.5 | 6.79 | 24.5 |
| **9.00-9.49** | **3** | **20a** | 9.19 | 7.72 | 16.0 | 6.73 | 26.8 | 6.10 | 33.6 | 7.58 | 17.5 | 6.48 | 29.5 | 6.26 | 31.9 | 6.11 | 33.5 |
| **9.50-9.99** | **2** | **22** | 9.82 | 6.17 | 37.2 | 6.16 | 37.3 | 8.69 | 11.5 | 5.57 | 43.3 | 6.81 | 30.6 | 6.34 | 35.5 | 6.62 | 32.6 |
| **10.00+** | **3** | **22** | 10.22 | 6.07 | 40.6 | 5.84 | 42.8 | 5.95 | 41.8 | 8.42 | 17.6 | 7.29 | 28.7 | 6.22 | 39.1 | 8.95 | 12.4 |

**Table S30**. Quantified distances based on the normalised intensity of each of the spectra recorded at different mixing times (100-700 ms) for the NOESY sequence (noesyph). Correlations for which the involved cross-peaks had opposing signs are excluded (indicated by “Phase” as an entry in the table). The theoretical population-averaged interproton distances as determined by Butts *et. al.*^[3]^ are given for each correlation, and used to calculate the absolute error (|%|). The proton-proton correlation used as a reference for distance calculation was proton-pair 15 and is highlighted by being underscored

|  |  |  |  | **100 ms** | | **200 ms** | | **300 ms** | | **400 ms** | | **500 ms** | | **600 ms** | | **700 ms** | |
| --- | --- | --- | --- | --- | --- | --- | --- | --- | --- | --- | --- | --- | --- | --- | --- | --- | --- |
|  | **H_a_** | **H_b_** | **r_ab_**  **Theor. (Å)** | **r_ab_ (Å)** | **Error (%)** | **r_ab_ (Å)** | **Error (%)** | **r_ab_ (Å)** | **Error (%)** | **r_ab_ (Å)** | **Error (%)** | **r_ab_ (Å)** | **Error (%)** | **r_ab_ (Å)** | **Error (%)** | **r_ab_ (Å)** | **Error (%)** |
| **0.00-1.99** | **20a** | **11b/20b** | 1.75 | 1.84 | 5.4 | 2.35 | 34.1 | 1.86 | 6.3 | 1.86 | 6.2 | 1.89 | 8.1 | 1.89 | 7.9 | 1.89 | 7.9 |
|  | **11a** | **11b/20b** | 1.76 | 2.46 | 40.0 | 2.16 | 22.6 | 1.95 | 11.0 | 1.91 | 8.5 | 1.91 | 8.8 | 1.90 | 8.0 | 1.89 | 7.6 |
|  | **15a** | **15b** | 1.76 | 1.76 | 0.0 | 1.76 | 0.0 | 1.76 | 0.0 | 1.76 | 0.0 | 1.76 | 0.0 | 1.76 | 0.0 | 1.76 | 0.0 |
|  | **17a** | **17b** | 1.77 | 2.24 | 26.4 | 1.84 | 4.1 | 1.79 | 0.9 | 1.75 | 1.1 | 1.79 | 1.0 | 1.78 | 0.5 | 1.77 | 0.2 |
|  | **18a** | **18b** | 1.77 | 2.48 | 40.0 | 2.05 | 15.9 | 1.80 | 1.7 | 1.83 | 3.2 | 1.82 | 3.0 | 1.82 | 2.7 | 1.80 | 1.9 |
| **2.00-2.49** | **20a** | **15a** | 2.24 | 2.77 | 23.8 | 2.21 | 1.2 | 2.18 | 2.6 | 2.14 | 4.3 | 2.17 | 3.1 | 2.16 | 3.4 | 2.16 | 3.8 |
|  | **15b** | **13** | 2.26 | Phase |  | Phase |  | 2.89 | 27.8 | 2.90 | 28.4 | 2.75 | 21.6 | 2.49 | 10.3 | 2.53 | 11.9 |
|  | **12** | **23ab** | 2.27 | 2.88 | 27.0 | 2.40 | 5.6 | 2.35 | 3.4 | 2.31 | 1.6 | 2.33 | 2.8 | 2.33 | 2.9 | 2.32 | 2.0 |
|  | **22** | **23ab** | 2.30 | 2.96 | 28.9 | 2.50 | 8.9 | 2.43 | 5.7 | 2.40 | 4.2 | 2.43 | 5.5 | 2.41 | 4.6 | 2.39 | 4.0 |
|  | **12** | **11a** | 2.35 | Phase |  | 2.22 | 5.4 | 2.24 | 4.6 | 2.31 | 1.8 | 2.36 | 0.3 | 2.33 | 0.9 | 2.32 | 1.5 |
|  | **12** | **13** | 2.35 | 2.86 | 21.5 | 2.45 | 4.2 | 2.36 | 0.3 | 2.33 | 0.9 | 2.36 | 0.3 | 2.34 | 0.2 | 2.33 | 0.7 |
|  | **8** | **18b** | 2.37 | 2.77 | 17.1 | 2.32 | 2.2 | 2.26 | 4.7 | 2.22 | 6.2 | 2.25 | 5.0 | 2.24 | 5.6 | 2.23 | 6.0 |
|  | **1** | **16** | 2.38 | 2.83 | 18.8 | 2.36 | 1.0 | 2.29 | 3.7 | 2.25 | 5.3 | 2.28 | 4.1 | 2.27 | 4.6 | 2.26 | 4.9 |
|  | **22** | **11b/20b** | 2.39 | 3.12 | 30.7 | 2.60 | 8.9 | 2.54 | 6.2 | 2.51 | 4.9 | 2.54 | 6.4 | 2.53 | 5.8 | 2.52 | 5.5 |
|  | **14** | **13** | 2.41 | 2.93 | 21.5 | 2.52 | 4.6 | 2.44 | 1.4 | 2.40 | 0.4 | 2.43 | 0.9 | 2.42 | 0.3 | 2.41 | 0.1 |
|  | **8** | **17a** | 2.42 | 2.93 | 21.0 | 2.44 | 0.9 | 2.39 | 1.2 | 2.34 | 3.3 | 2.38 | 1.8 | 2.36 | 2.5 | 2.35 | 2.8 |
|  | **18a** | **17b** | 2.43 | 2.83 | 16.3 | 2.47 | 1.7 | 2.49 | 2.5 | 2.38 | 1.9 | 2.41 | 0.8 | 2.41 | 1.0 | 2.40 | 1.2 |
|  | **18b** | **17a** | 2.43 | 2.81 | 15.5 | 3.19 | 31.2 | 2.39 | 1.7 | 2.46 | 1.3 | 2.46 | 1.1 | 2.45 | 0.7 | 2.44 | 0.6 |
|  | **18b** | **11b/20b** | 2.45 | Phase |  | Phase |  | 2.82 | 15.0 | 2.69 | 10.0 | 2.72 | 10.9 | 2.78 | 13.5 | 2.80 | 14.2 |
|  | **3** | **2** | 2.48 | 2.90 | 16.8 | 3.11 | 25.3 | 2.33 | 6.0 | 2.75 | 11.0 | 2.58 | 4.0 | 2.75 | 11.0 | 2.44 | 1.7 |
|  | **16** | **15a** | 2.48 | 2.86 | 15.3 | 2.60 | 4.8 | 2.40 | 3.3 | 2.42 | 2.4 | 2.46 | 0.9 | 2.45 | 1.0 | 2.46 | 1.0 |
|  | **16** | **15b** | 2.48 | Phase |  | 2.58 | 4.1 | 2.43 | 2.1 | 2.39 | 3.7 | 2.44 | 1.6 | 2.44 | 1.8 | 2.44 | 1.6 |
|  | **14** | **15a** | 2.48 | 2.96 | 19.2 | 2.55 | 2.9 | 2.48 | 0.1 | 2.42 | 2.2 | 2.46 | 0.6 | 2.45 | 1.1 | 2.45 | 1.2 |
|  | **1** | **2** | 2.49 | 2.66 | 6.8 | 2.27 | 8.9 | 2.73 | 9.8 | 2.45 | 1.6 | 2.80 | 12.6 | 2.43 | 2.5 | 2.44 | 1.9 |
| **2.50-2.99** | **4** | **3** | 2.50 | 2.60 | 3.8 | 2.20 | 12.1 | Phase |  | 2.50 | 0.2 | 2.49 | 0.4 | 2.47 | 1.0 | 2.48 | 0.9 |
|  | **14** | **15b** | 2.55 | 3.58 | 40.5 | 2.51 | 1.7 | 2.49 | 2.2 | 2.47 | 3.2 | 2.53 | 0.8 | 2.53 | 0.7 | 2.54 | 0.4 |
|  | **23ab** | **14** | 2.59 | 3.18 | 22.7 | 2.67 | 3.0 | 2.59 | 0.2 | 2.55 | 1.5 | 2.59 | 0.1 | 2.56 | 1.0 | 2.56 | 1.3 |
|  | **8** | **11b/20b** | 2.63 | 3.43 | 30.3 | 2.76 | 4.8 | 2.70 | 2.6 | 2.65 | 0.7 | 2.69 | 2.1 | 2.66 | 1.2 | 2.66 | 1.0 |
|  | **16** | **17b** | 2.71 | 3.24 | 19.4 | 2.70 | 0.2 | 2.64 | 2.6 | 2.60 | 4.0 | 2.63 | 2.8 | 2.62 | 3.3 | 2.61 | 3.6 |
|  | **18a** | **17a** | 2.73 | 4.58 | 67.7 | 3.37 | 23.4 | 2.63 | 3.8 | 2.88 | 5.4 | 2.89 | 5.9 | 3.00 | 10.1 | 3.12 | 14.4 |
|  | **12** | **14** | 2.74 | 3.26 | 19.0 | 2.79 | 1.8 | 2.71 | 1.0 | 2.67 | 2.5 | 2.71 | 0.9 | 2.70 | 1.4 | 2.70 | 1.5 |
|  | **1** | **17b** | 2.79 | 3.39 | 21.4 | 2.82 | 1.0 | 2.75 | 1.3 | 2.70 | 3.3 | 2.75 | 1.6 | 2.72 | 2.3 | 2.72 | 2.3 |
|  | **18a** | **11b/20b** | 2.82 | 3.88 | 37.5 | 2.94 | 4.2 | 3.07 | 8.8 | 3.06 | 8.5 | 3.18 | 12.7 | 3.16 | 12.2 | 3.16 | 11.9 |
|  | **12** | **11b/20b** | 2.93 | Phase |  | 3.14 | 7.3 | 3.52 | 20.3 | 3.17 | 8.0 | 3.31 | 13.0 | 3.37 | 15.1 | 3.47 | 18.4 |
| **3.00-3.49** | **8** | **13** | 3.01 | 3.81 | 26.6 | 2.80 | 6.9 | 2.89 | 4.0 | 3.00 | 0.5 | 3.05 | 1.5 | 3.00 | 0.2 | 3.00 | 0.3 |
|  | **18b** | **17b** | 3.06 | 3.03 | 0.9 | 4.71 | 53.8 | 3.58 | 17.0 | 4.30 | 40.5 | 4.66 | 52.3 | 3.87 | 26.4 | 3.72 | 21.6 |
|  | **22** | **18b** | 3.16 | 3.73 | 18.0 | 3.10 | 1.9 | 3.00 | 5.2 | 2.97 | 5.9 | 3.02 | 4.4 | 3.00 | 4.9 | 2.99 | 5.4 |
|  | **23ab** | **11b/20b** | 3.24 | 4.66 | 43.8 | 3.61 | 11.3 | 3.53 | 9.1 | 3.50 | 8.1 | 3.66 | 13.1 | 3.57 | 10.2 | 3.63 | 12.1 |
|  | **20a** | **14** | 3.36 | 4.33 | 28.8 | 3.42 | 1.7 | 3.40 | 1.1 | 3.33 | 0.9 | 3.38 | 0.7 | 3.41 | 1.5 | 3.40 | 1.2 |
|  | **16** | **20a** | 3.37 | 3.74 | 11.1 | 3.45 | 2.3 | 3.59 | 6.5 | 3.40 | 0.8 | 3.39 | 0.5 | 3.49 | 3.6 | 3.42 | 1.4 |
|  | **22** | **20a** | 3.41 | 5.00 | 46.6 | 3.65 | 7.1 | 4.05 | 18.7 | 7.72 | 126.5 | 4.79 | 40.5 | 4.08 | 19.6 | 3.71 | 8.7 |
| **3.50-3.99** | **15a** | **13** | 3.54 | 2.61 | 26.3 | 3.61 | 2.1 | 5.41 | 52.9 | Phase |  | 3.43 | 3.2 | 3.25 | 8.3 | 3.16 | 10.6 |
|  | **20a** | **18b** | 3.60 | 3.02 | 16.1 | 3.04 | 15.6 | Phase |  | Phase |  | Phase |  | 4.05 | 12.6 | 3.73 | 3.6 |
|  | **8** | **17b** | 3.61 | 4.50 | 24.6 | 4.01 | 11.0 | 4.16 | 15.1 | 5.08 | 40.6 | 5.73 | 58.6 | 4.26 | 17.9 | 3.96 | 9.8 |
|  | **11b/20b** | **13** | 3.61 | 6.94 | 92.4 | 4.60 | 27.4 | 4.32 | 19.6 | 4.20 | 16.4 | 4.29 | 18.9 | 4.29 | 18.9 | 4.35 | 20.6 |
|  | **16** | **18a** | 3.62 | 4.43 | 22.3 | 3.74 | 3.2 | 3.70 | 2.3 | 3.65 | 0.7 | 3.71 | 2.4 | 3.68 | 1.7 | 3.72 | 2.8 |
|  | **12** | **8** | 3.65 | 5.20 | 42.4 | 3.73 | 2.3 | 3.74 | 2.4 | 3.68 | 0.8 | 3.72 | 2.0 | 3.79 | 3.8 | 3.72 | 1.9 |
|  | **11a** | **13** | 3.66 | 4.75 | 29.7 | 3.76 | 2.7 | 3.62 | 1.1 | 3.62 | 1.0 | 3.62 | 1.1 | 3.64 | 0.6 | 3.68 | 0.6 |
|  | **11b/20b** | **15a** | 3.69 | Phase |  | Phase |  | 3.67 | 0.5 | 3.46 | 6.2 | 3.38 | 8.3 | 3.20 | 13.3 | 3.14 | 14.9 |
|  | **23ab** | **8** | 3.73 | Phase |  | 4.12 | 10.4 | 5.27 | 41.3 | 4.67 | 25.1 | 3.90 | 4.5 | 4.11 | 10.1 | 3.88 | 4.1 |
|  | **20a** | **15b** | 3.75 | Phase |  | 4.46 | 19.0 | 3.62 | 3.4 | 3.26 | 13.0 | 3.20 | 14.6 | 3.09 | 17.7 | 3.00 | 20.0 |
|  | **23ab** | **13** | 3.76 | Phase |  | Phase |  | 5.23 | 39.0 | Phase |  | 6.25 | 66.1 | 4.62 | 22.8 | 4.44 | 18.0 |
|  | **20a** | **18a** | 3.81 | Phase |  | 4.27 | 12.0 | 4.43 | 16.2 | Phase |  | Phase |  | Phase |  | 5.37 | 41.1 |
|  | **8** | **18a** | 3.83 | Phase |  | Phase |  | 4.01 | 4.6 | 3.63 | 5.2 | 3.49 | 8.8 | 3.32 | 13.3 | 3.21 | 16.2 |
|  | **16** | **17a** | 3.84 | 4.76 | 23.9 | 4.13 | 7.6 | 4.74 | 23.6 | 4.58 | 19.2 | Phase |  | Phase |  | 4.41 | 15.0 |
|  | **1** | **15b** | 3.86 | 4.97 | 28.6 | 3.81 | 1.3 | 3.95 | 2.5 | 3.84 | 0.5 | 4.10 | 6.2 | 3.97 | 3.0 | 4.26 | 10.3 |
|  | **22** | **14** | 3.86 | 4.73 | 22.5 | 4.08 | 5.8 | 3.86 | 0.1 | 3.86 | 0.0 | 3.91 | 1.2 | 3.91 | 1.3 | 3.97 | 2.7 |
|  | **8** | **11a** | 3.88 | 5.37 | 38.3 | 4.42 | 13.9 | 4.66 | 20.1 | Phase |  | Phase |  | 4.54 | 17.0 | 4.13 | 6.4 |
|  | **16** | **18b** | 3.89 | 4.51 | 15.9 | 3.91 | 0.6 | 4.05 | 4.0 | 3.94 | 1.3 | 3.96 | 1.7 | 4.11 | 5.7 | 4.17 | 7.3 |
|  | **22** | **8** | 3.96 | 4.72 | 19.2 | 4.01 | 1.4 | 3.95 | 0.2 | 3.90 | 1.4 | 3.86 | 2.5 | 3.87 | 2.2 | 3.89 | 1.8 |
|  | **23ab** | **11a** | 3.96 | Phase |  | 4.97 | 25.5 | 4.69 | 18.4 | 5.60 | 41.3 | 4.83 | 21.9 | 4.31 | 8.8 | 4.09 | 3.4 |
|  | **8** | **14** | 3.96 | 6.72 | 69.8 | 4.44 | 12.2 | 4.22 | 6.6 | 3.95 | 0.2 | 4.06 | 2.6 | 4.07 | 2.9 | 4.09 | 3.3 |
|  | **14** | **11b/20b** | 3.96 | Phase |  | 4.81 | 21.4 | 4.78 | 20.7 | Phase |  | 5.34 | 34.8 | Phase |  | 5.44 | 37.3 |
| **4.00-4.49** | **1** | **17a** | 4.06 | 5.02 | 23.7 | 4.39 | 8.1 | 5.09 | 25.5 | 4.85 | 19.4 | Phase |  | Phase |  | 4.60 | 13.4 |
|  | **16** | **13** | 4.06 | 3.71 | 8.6 | 3.84 | 5.4 | 4.13 | 1.6 | 4.46 | 9.9 | 4.37 | 7.7 | 6.82 | 68.0 | 5.16 | 27.1 |
|  | **16** | **11b/20b** | 4.07 | Phase |  | 4.71 | 15.8 | 4.59 | 12.9 | 5.04 | 23.8 | 4.79 | 17.7 | 4.88 | 19.9 | 5.60 | 37.6 |
|  | **8** | **15b** | 4.09 | 4.76 | 16.4 | 3.31 | 19.0 | Phase |  | 4.29 | 5.0 | 3.95 | 3.3 | 5.03 | 23.1 | 4.27 | 4.5 |
|  | **16** | **8** | 4.14 | 3.51 | 15.2 | Phase |  | 4.98 | 20.4 | Phase |  | Phase |  | Phase |  | Phase |  |
|  | **11b/20b** | **17a** | 4.17 | Phase |  | Phase |  | 6.50 | 55.8 | Phase |  | 5.09 | 22.0 | 4.71 | 13.0 | 4.55 | 9.0 |
|  | **12** | **15b** | 4.27 | 6.93 | 62.2 | 4.11 | 3.6 | Phase |  | Phase |  | 5.58 | 30.7 | 5.13 | 20.2 | 4.28 | 0.3 |
|  | **23ab** | **18b** | 4.27 | 6.16 | 44.1 | 5.05 | 18.3 | 4.51 | 5.6 | 5.06 | 18.6 | 5.17 | 21.1 | 4.88 | 14.3 | 5.30 | 24.1 |
|  | **1** | **3** | 4.30 | 3.77 | 12.3 | 2.96 | 31.1 | 4.26 | 1.0 | 4.00 | 6.9 | 3.34 | 22.2 | 4.10 | 4.7 | 3.60 | 16.3 |
|  | **16** | **14** | 4.30 | Phase |  | Phase |  | 4.58 | 6.5 | 5.21 | 21.1 | Phase |  | Phase |  | Phase |  |
|  | **4** | **2** | 4.32 | 3.92 | 9.2 | 3.26 | 24.5 | 3.70 | 14.5 | Phase |  | 4.23 | 2.0 | 4.80 | 11.1 | 4.79 | 10.9 |
|  | **4** | **13** | 4.43 | 6.01 | 35.8 | 4.60 | 3.9 | 4.71 | 6.4 | 4.34 | 2.0 | 4.47 | 0.9 | 4.40 | 0.8 | 4.42 | 0.2 |
|  | **23ab** | **20a** | 4.44 | Phase |  | Phase |  | Phase |  | Phase |  | 4.70 | 5.9 | Phase |  | 5.40 | 21.5 |
|  | **22** | **18a** | 4.47 | Phase |  | 5.56 | 24.3 | Phase |  | Phase |  | 5.22 | 16.8 | 4.86 | 8.8 | 4.70 | 5.2 |
|  | **11b/20b** | **17b** | 4.49 | 4.86 | 8.1 | 5.05 | 12.5 | 5.50 | 22.4 | 6.19 | 38.0 | 5.06 | 12.8 | 5.41 | 20.5 | 5.22 | 16.2 |
| **4.50-4.99** | **18b** | **15a** | 4.55 | Phase |  | Phase |  | Phase |  | Phase |  | Phase |  | Phase |  | 6.14 | 34.9 |
|  | **11b/20b** | **15b** | 4.59 | 4.48 | 2.5 | 4.72 | 2.8 | 5.84 | 27.3 | 6.49 | 41.4 | Phase |  | 6.15 | 34.0 | Phase |  |
|  | **1** | **15a** | 4.62 | 7.11 | 53.9 | 5.26 | 13.9 | Phase |  | 5.38 | 16.3 | 4.92 | 6.4 | 4.61 | 0.2 | 4.22 | 8.6 |
|  | **8** | **15a** | 4.63 | Phase |  | 4.73 | 2.1 | 6.46 | 39.6 | 4.88 | 5.5 | 4.82 | 4.2 | 4.85 | 4.8 | 4.75 | 2.6 |
|  | **17b** | **15b** | 4.64 | Phase |  | Phase |  | Phase |  | Phase |  | Phase |  | Phase |  | 5.64 | 21.5 |
|  | **22** | **12** | 4.65 | Phase |  | Phase |  | Phase |  | Phase |  | 9.96 | 114.1 | Phase |  | 5.59 | 20.2 |
|  | **14** | **18b** | 4.66 | Phase |  | Phase |  | 4.54 | 2.6 | Phase |  | 4.40 | 5.5 | 4.63 | 0.6 | 4.11 | 11.8 |
|  | **23ab** | **15a** | 4.68 | Phase |  | Phase |  | Phase |  | 4.77 | 2.0 | Phase |  | 4.67 | 0.2 | 4.91 | 4.8 |
|  | **11a** | **17a** | 4.68 | 4.58 | 2.2 | 4.58 | 2.1 | Phase |  | Phase |  | 6.12 | 30.7 | Phase |  | 5.50 | 17.5 |
|  | **8** | **20a** | 4.71 | Phase |  | Phase |  | Phase |  | Phase |  | 4.86 | 3.3 | Phase |  | Phase |  |
|  | **18b** | **13** | 4.73 | Phase |  | 5.12 | 8.2 | Phase |  | Phase |  | Phase |  | Phase |  | 5.68 | 20.0 |
|  | **2** | **16** | 4.75 | Phase |  | Phase |  | 5.61 | 18.2 | 5.50 | 15.8 | Phase |  | 5.38 | 13.2 | 4.87 | 2.4 |
|  | **4** | **11a** | 4.77 | Phase |  | 4.81 | 0.8 | 4.79 | 0.3 | 4.95 | 3.8 | 4.72 | 1.0 | 4.94 | 3.5 | 4.60 | 3.6 |
|  | **11a** | **18b** | 4.77 | Phase |  | Phase |  | Phase |  | Phase |  | Phase |  | Phase |  | 5.44 | 14.1 |
|  | **17a** | **13** | 4.79 | Phase |  | Phase |  | Phase |  | Phase |  | Phase |  | Phase |  | Phase |  |
|  | **22** | **15a** | 4.81 | Phase |  | Phase |  | 5.57 | 15.7 | 5.43 | 12.8 | 5.46 | 13.5 | 5.34 | 11.1 | 4.94 | 2.8 |
|  | **23ab** | **15b** | 4.81 | 5.43 | 13.0 | Phase |  | 6.27 | 30.3 | 5.13 | 6.6 | Phase |  | Phase |  | 4.91 | 2.1 |
|  | **2** | **17b** | 4.82 | 6.25 | 29.7 | Phase |  | 5.30 | 9.9 | 5.81 | 20.6 | 5.41 | 12.2 | 7.45 | 54.5 | 5.87 | 21.7 |
|  | **18b** | **15b** | 4.83 | 4.70 | 2.6 | Phase |  | Phase |  | Phase |  | 6.48 | 34.2 | 5.39 | 11.6 | 6.69 | 38.6 |
|  | **14** | **11a** | 4.87 | Phase |  | Phase |  | Phase |  | Phase |  | Phase |  | Phase |  | 4.83 | 0.9 |
|  | **15a** | **17b** | 4.87 | Phase |  | Phase |  | Phase |  | Phase |  | Phase |  | Phase |  | Phase |  |
|  | **20a** | **13** | 4.88 | Phase |  | 4.88 | 0.0 | 5.45 | 11.6 | Phase |  | Phase |  | Phase |  | Phase |  |
|  | **4** | **8** | 4.89 | Phase |  | 4.86 | 0.7 | 4.77 | 2.5 | 4.71 | 3.6 | 4.95 | 1.2 | 4.78 | 2.3 | 4.88 | 0.3 |
|  | **4** | **11b/20b** | 4.89 | Phase |  | 5.92 | 21.1 | Phase |  | 5.33 | 9.0 | 5.53 | 13.1 | 6.08 | 24.4 | 5.63 | 15.2 |
|  | **18a** | **15a** | 4.89 | Phase |  | Phase |  | 5.00 | 2.2 | 5.75 | 17.7 | 5.26 | 7.5 | 6.26 | 27.9 | 4.65 | 5.0 |
|  | **1** | **18a** | 4.90 | 8.82 | 80.1 | 4.94 | 0.8 | Phase |  | 5.93 | 21.0 | 5.90 | 20.3 | 5.90 | 20.5 | 5.76 | 17.5 |
|  | **20a** | **17b** | 4.90 | Phase |  | Phase |  | 6.32 | 28.9 | Phase |  | 5.52 | 12.6 | 5.78 | 17.9 | 5.27 | 7.5 |
|  | **1** | **13** | 4.98 | Phase |  | 5.71 | 14.7 | 4.86 | 2.5 | 6.35 | 27.6 | 4.98 | 0.1 | 5.28 | 6.1 | 5.17 | 3.8 |
|  | **12** | **15a** | 4.98 | Phase |  | 6.86 | 37.7 | Phase |  | 5.65 | 13.4 | 5.96 | 19.6 | 5.23 | 5.0 | 5.27 | 5.8 |
| **5.00-5.49** | **4** | **1** | 5.00 | 4.30 | 14.1 | Phase |  | Phase |  | 5.15 | 3.1 | Phase |  | Phase |  | 5.14 | 2.8 |
|  | **17a** | **13** | 5.05 | Phase |  | Phase |  | Phase |  | Phase |  | Phase |  | Phase |  | 5.79 | 14.6 |
|  | **1** | **8** | 5.06 | Phase |  | 4.80 | 5.0 | 5.33 | 5.4 | 5.32 | 5.2 | 4.73 | 6.5 | 5.41 | 7.0 | 4.72 | 6.8 |
|  | **4** | **17a** | 5.17 | 5.55 | 7.4 | Phase |  | 5.20 | 0.6 | 4.75 | 8.2 | 5.03 | 2.8 | 5.35 | 3.6 | 4.96 | 4.0 |
|  | **17b** | **13** | 5.17 | Phase |  | Phase |  | Phase |  | Phase |  | Phase |  | Phase |  | Phase |  |
|  | **22** | **13** | 5.19 | 6.54 | 26.0 | 5.17 | 0.5 | Phase |  | 6.82 | 31.5 | 7.51 | 44.8 | 6.29 | 21.2 | Phase |  |
|  | **1** | **18b** | 5.32 | 5.97 | 12.3 | 5.79 | 8.9 | Phase |  | Phase |  | Phase |  | Phase |  | Phase |  |
|  | **20a** | **17a** | 5.35 | 6.05 | 13.0 | Phase |  | Phase |  | 6.54 | 22.3 | 5.55 | 3.7 | 5.60 | 4.7 | Phase |  |
|  | **12** | **18b** | 5.38 | 5.11 | 5.1 | 4.63 | 14.0 | 5.66 | 5.3 | Phase |  | 5.13 | 4.7 | 6.02 | 12.0 | 7.55 | 40.2 |
|  | **18a** | **15b** | 5.39 | Phase |  | Phase |  | Phase |  | Phase |  | 5.75 | 6.6 | Phase |  | Phase |  |
|  | **22** | **17a** | 5.41 | Phase |  | Phase |  | Phase |  | 5.67 | 4.7 | Phase |  | 5.66 | 4.7 | 5.57 | 2.9 |
|  | **2** | **15b** | 5.44 | Phase |  | Phase |  | 5.80 | 6.6 | 4.74 | 12.9 | Phase |  | 6.51 | 19.6 | 5.46 | 0.3 |
|  | **15a** | **17a** | 5.46 | Phase |  | Phase |  | Phase |  | Phase |  | Phase |  | Phase |  | Phase |  |
| **5.50-5.99** | **4** | **15b** | 5.53 | 5.35 | 3.3 | 5.08 | 8.2 | 5.28 | 4.6 | 5.43 | 1.8 | 6.28 | 13.6 | 6.83 | 23.5 | 7.63 | 38.0 |
|  | **22** | **15b** | 5.56 | Phase |  | Phase |  | 6.32 | 13.7 | Phase |  | 7.30 | 31.2 | Phase |  | Phase |  |
|  | **2** | **17a** | 5.67 | 6.93 | 22.2 | Phase |  | Phase |  | 5.26 | 7.3 | Phase |  | 6.30 | 11.1 | 6.31 | 11.4 |
|  | **11a** | **15b** | 5.69 | 5.60 | 1.6 | 4.83 | 15.2 | Phase |  | 4.73 | 16.9 | 5.95 | 4.5 | 6.53 | 14.7 | 6.31 | 11.0 |
|  | **4** | **17b** | 5.70 | 7.12 | 24.9 | 6.30 | 10.5 | 8.38 | 47.0 | Phase |  | 6.85 | 20.3 | 5.69 | 0.3 | Phase |  |
|  | **12** | **20a** | 5.70 | Phase |  | Phase |  | Phase |  | Phase |  | Phase |  | Phase |  | Phase |  |
|  | **23ab** | **17a** | 5.70 | Phase |  | Phase |  | Phase |  | Phase |  | Phase |  | Phase |  | 5.40 | 5.2 |
|  | **22** | **16** | 5.71 | Phase |  | Phase |  | 5.73 | 0.3 | 5.92 | 3.7 | Phase |  | Phase |  | 7.73 | 35.3 |
|  | **23ab** | **18a** | 5.72 | Phase |  | 5.22 | 8.7 | 6.13 | 7.1 | 5.32 | 7.0 | 5.50 | 3.8 | 5.03 | 12.1 | Phase |  |
|  | **1** | **20a** | 5.74 | Phase |  | Phase |  | 6.07 | 5.8 | Phase |  | Phase |  | Phase |  | 6.55 | 14.2 |
|  | **14** | **18a** | 5.79 | Phase |  | Phase |  | 4.08 | 29.5 | 4.10 | 29.2 | Phase |  | Phase |  | 3.18 | 45.0 |
|  | **14** | **17a** | 5.79 | Phase |  | Phase |  | 6.04 | 4.3 | Phase |  | 6.62 | 14.3 | 5.31 | 8.4 | 7.17 | 23.9 |
|  | **4** | **12** | 5.87 | 5.26 | 10.4 | 6.57 | 11.9 | 5.47 | 6.8 | 5.53 | 5.8 | 5.87 | 0.0 | Phase |  | Phase |  |
|  | **1** | **11b/20b** | 5.89 | 7.71 | 30.9 | 6.42 | 8.9 | 7.03 | 19.4 | 6.14 | 4.2 | 6.53 | 10.9 | Phase |  | Phase |  |
|  | **18a** | **13** | 5.89 | Phase |  | Phase |  | Phase |  | 5.15 | 12.6 | Phase |  | Phase |  | Phase |  |
|  | **3** | **13** | 5.90 | 6.40 | 8.5 | Phase |  | Phase |  | 6.49 | 10.1 | 6.78 | 15.0 | 5.54 | 6.1 | 5.92 | 0.3 |
|  | **23ab** | **16** | 5.93 | Phase |  | Phase |  | 4.10 | 30.8 | Phase |  | Phase |  | 4.09 | 31.1 | Phase |  |
|  | **3** | **17b** | 5.95 | Phase |  | Phase |  | 6.33 | 6.4 | Phase |  | Phase |  | Phase |  | Phase |  |
|  | **14** | **17b** | 5.95 | 6.07 | 2.0 | 5.45 | 8.4 | Phase |  | 6.62 | 11.3 | Phase |  | 6.76 | 13.7 | Phase |  |
| **6.00-6.49** | **12** | **17a** | 6.01 | Phase |  | 5.60 | 6.9 | 5.81 | 3.4 | Phase |  | 7.22 | 20.1 | Phase |  | 6.05 | 0.7 |
|  | **22** | **17b** | 6.03 | 7.02 | 16.5 | 5.92 | 1.8 | Phase |  | Phase |  | Phase |  | 6.80 | 12.8 | 6.22 | 3.2 |
|  | **12** | **16** | 6.03 | Phase |  | Phase |  | Phase |  | Phase |  | Phase |  | Phase |  | Phase |  |
|  | **4** | **16** | 6.07 | 5.23 | 13.9 | 5.38 | 11.4 | Phase |  | 5.44 | 10.4 | 5.77 | 5.0 | 7.95 | 31.1 | 6.71 | 10.5 |
|  | **3** | **17a** | 6.09 | Phase |  | Phase |  | 5.95 | 2.2 | Phase |  | Phase |  | 8.04 | 32.0 | Phase |  |
|  | **3** | **15b** | 6.11 | Phase |  | Phase |  | Phase |  | Phase |  | 5.91 | 3.2 | Phase |  | 5.80 | 5.0 |
|  | **2** | **13** | 6.11 | Phase |  | 5.51 | 9.9 | 6.94 | 13.6 | 8.29 | 35.7 | 5.96 | 2.4 | 6.37 | 4.2 | 7.25 | 18.7 |
|  | **11a** | **17b** | 6.11 | Phase |  | Phase |  | Phase |  | 4.97 | 18.7 | 5.98 | 2.1 | Phase |  | Phase |  |
|  | **1** | **14** | 6.12 | 6.04 | 1.3 | Phase |  | 7.22 | 18.0 | Phase |  | 6.31 | 3.1 | Phase |  | 6.33 | 3.4 |
|  | **22** | **11a** | 6.14 | 5.54 | 9.8 | Phase |  | 6.33 | 3.1 | 5.72 | 6.9 | 7.01 | 14.1 | 5.90 | 3.9 | Phase |  |
|  | **3** | **16** | 6.16 | Phase |  | 5.72 | 7.1 | 5.36 | 13.1 | Phase |  | Phase |  | 6.29 | 2.1 | 5.56 | 9.7 |
| **6.50-6.99** | **23ab** | **17b** | 6.50 | Phase |  | 5.21 | 19.8 | Phase |  | 5.37 | 17.3 | Phase |  | 5.50 | 15.4 | Phase |  |
|  | **3** | **8** | 6.53 | 6.30 | 3.6 | 5.69 | 12.8 | Phase |  | 6.24 | 4.4 | 7.62 | 16.7 | Phase |  | 5.70 | 12.7 |
|  | **11a** | **15a** | 6.53 | Phase |  | Phase |  | Phase |  | Phase |  | Phase |  | 7.07 | 8.2 | 8.46 | 29.6 |
|  | **2** | **8** | 6.59 | 7.88 | 19.5 | 7.16 | 8.7 | Phase |  | 5.95 | 9.7 | 6.90 | 4.6 | 8.61 | 30.6 | Phase |  |
|  | **2** | **15a** | 6.64 | Phase |  | Phase |  | Phase |  | Phase |  | 7.27 | 9.5 | Phase |  | Phase |  |
|  | **4** | **14** | 6.82 | 6.15 | 9.8 | Phase |  | 5.51 | 19.2 | Phase |  | Phase |  | 6.60 | 3.2 | 8.86 | 30.0 |
|  | **12** | **17b** | 6.83 | Phase |  | 5.36 | 21.5 | 5.45 | 20.2 | 6.14 | 10.2 | Phase |  | 6.08 | 11.0 | 6.53 | 4.3 |
|  | **4** | **18b** | 6.88 | 4.97 | 27.8 | 5.49 | 20.2 | 5.76 | 16.3 | Phase |  | 6.11 | 11.2 | 6.99 | 1.6 | 6.17 | 10.4 |
|  | **12** | **18a** | 6.94 | 4.15 | 40.2 | 3.89 | 43.9 | 4.26 | 38.7 | Phase |  | Phase |  | Phase |  | 5.33 | 23.1 |
| **7.00-7.49** | **3** | **11b/20b** | 7.02 | Phase |  | 5.28 | 24.8 | 5.73 | 18.4 | 5.16 | 26.4 | 7.31 | 4.2 | 6.94 | 1.2 | Phase |  |
|  | **16** | **11a** | 7.08 | Phase |  | Phase |  | 5.30 | 25.1 | Phase |  | Phase |  | Phase |  | Phase |  |
|  | **4** | **15a** | 7.13 | 5.67 | 20.4 | 6.77 | 5.0 | Phase |  | 7.28 | 2.0 | 5.98 | 16.1 | Phase |  | Phase |  |
|  | **3** | **11a** | 7.13 | Phase |  | 5.91 | 17.1 | 5.16 | 27.6 | Phase |  | 7.03 | 1.4 | 8.26 | 15.9 | Phase |  |
|  | **2** | **18a** | 7.16 | 5.39 | 24.8 | Phase |  | Phase |  | Phase |  | Phase |  | 5.86 | 18.1 | 6.21 | 13.3 |
|  | **4** | **23ab** | 7.21 | Phase |  | Phase |  | Phase |  | 6.36 | 11.8 | Phase |  | Phase |  | Phase |  |
|  | **1** | **12** | 7.22 | 6.65 | 8.0 | 6.47 | 10.4 | Phase |  | Phase |  | 5.89 | 18.4 | 7.40 | 2.5 | Phase |  |
|  | **2** | **18b** | 7.42 | 6.15 | 17.2 | 5.67 | 23.6 | 5.55 | 25.2 | 5.51 | 25.7 | Phase |  | Phase |  | 7.11 | 4.2 |
|  | **2** | **11b/20b** | 7.43 | Phase |  | 4.84 | 34.8 | 7.83 | 5.3 | Phase |  | 5.45 | 26.6 | Phase |  | 5.72 | 23.0 |
|  | **20a** | **11a** | 7.48 | 4.81 | 35.7 | Phase |  | Phase |  | Phase |  | Phase |  | Phase |  | Phase |  |
| **7.50-7.99** | **1** | **23ab** | 7.50 | Phase |  | 6.10 | 18.7 | Phase |  | Phase |  | 5.71 | 23.8 | Phase |  | 5.45 | 27.4 |
|  | **4** | **18a** | 7.60 | Phase |  | Phase |  | Phase |  | 6.77 | 10.9 | Phase |  | Phase |  | Phase |  |
|  | **1** | **11a** | 7.64 | 6.11 | 20.1 | 6.02 | 21.1 | Phase |  | Phase |  | 5.60 | 26.7 | 6.02 | 21.2 | 6.12 | 19.9 |
|  | **3** | **15a** | 7.67 | 6.60 | 13.9 | Phase |  | 5.88 | 23.3 | 5.77 | 24.8 | Phase |  | Phase |  | Phase |  |
|  | **18a** | **11a** | 7.69 | Phase |  | 3.31 | 57.0 | 3.36 | 56.3 | Phase |  | Phase |  | 3.60 | 53.1 | 3.07 | 60.1 |
|  | **1** | **22** | 7.71 | Phase |  | 6.73 | 12.7 | 5.21 | 32.4 | Phase |  | Phase |  | Phase |  | 7.48 | 3.0 |
|  | **2** | **14** | 7.80 | 6.29 | 19.4 | 6.06 | 22.4 | Phase |  | 6.79 | 13.0 | 8.64 | 10.8 | Phase |  | 6.71 | 13.9 |
|  | **3** | **12** | 7.83 | 6.16 | 21.4 | Phase |  | 6.76 | 13.7 | Phase |  | Phase |  | 7.07 | 9.7 | 6.27 | 20.0 |
| **8.00-8.49** | **3** | **18b** | 8.03 | Phase |  | 6.09 | 24.2 | 5.68 | 29.2 | 5.48 | 31.8 | Phase |  | Phase |  | Phase |  |
|  | **3** | **14** | 8.08 | Phase |  | Phase |  | 5.71 | 29.4 | 6.17 | 23.6 | 7.18 | 11.2 | Phase |  | Phase |  |
|  | **2** | **20a** | 8.09 | 6.12 | 24.4 | 5.62 | 30.5 | 5.93 | 26.7 | Phase |  | 6.85 | 15.4 | 6.48 | 20.0 | 5.76 | 28.8 |
|  | **3** | **18a** | 8.25 | Phase |  | Phase |  | 6.56 | 20.5 | 5.58 | 32.3 | 5.69 | 31.0 | 5.79 | 29.8 | Phase |  |
|  | **2** | **11a** | 8.28 | 5.17 | 37.6 | 5.73 | 30.9 | Phase |  | Phase |  | Phase |  | 6.26 | 24.4 | 6.32 | 23.6 |
|  | **2** | **12** | 8.37 | Phase |  | Phase |  | 5.35 | 36.1 | 8.54 | 2.1 | Phase |  | Phase |  | Phase |  |
|  | **4** | **20a** | 8.46 | 7.09 | 16.2 | 7.35 | 13.1 | Phase |  | Phase |  | 6.35 | 24.9 | Phase |  | Phase |  |
| **8.50-8.99** | **4** | **22** | 8.70 | 13.66 | 57.1 | Phase |  | 5.09 | 41.5 | 6.08 | 30.1 | Phase |  | Phase |  | 11.20 | 28.8 |
|  | **3** | **23ab** | 8.88 | 5.31 | 40.2 | 5.20 | 41.4 | 7.69 | 13.4 | Phase |  | Phase |  | 6.26 | 29.5 | Phase |  |
|  | **2** | **23ab** | 8.99 | 5.88 | 34.6 | 5.55 | 38.3 | 5.28 | 41.2 | Phase |  | Phase |  | 5.89 | 34.5 | 6.79 | 24.5 |
| **9.00-9.49** | **3** | **20a** | 9.19 | Phase |  | 6.73 | 26.8 | 6.10 | 33.6 | 7.58 | 17.5 | 6.48 | 29.5 | 6.26 | 31.9 | 6.11 | 33.5 |
| **9.50-9.99** | **2** | **22** | 9.82 | 6.17 | 37.2 | Phase |  | Phase |  | Phase |  | 6.81 | 30.6 | 6.34 | 35.5 | 6.62 | 32.6 |
| **10.00+** | **3** | **22** | 10.22 | 6.07 | 40.6 | Phase |  | 5.95 | 41.8 | 8.42 | 17.6 | 7.29 | 28.7 | Phase |  | Phase |  |

**Table S31**. Quantified distances based on the normalised intensity of each of the spectra recorded at different mixing times (100-700 ms) for the NOESY sequence (noesyph). Correlations for which the involved cross-peaks had opposing signs (indicated by “Phase” as an entry in the table), as well as those that were calculated to be 5.50 Å or more are excluded (blank entries). The theoretical population-averaged interproton distances as determined by Butts *et. al.*^[3]^ are given for each correlation, and used to calculate the absolute error (|%|). The proton-proton correlation used as a reference for distance calculation was proton-pair 15 and is highlighted by being underscored

|  |  |  |  | **100 ms** | | **200 ms** | | **300 ms** | | **400 ms** | | **500 ms** | | **600 ms** | | **700 ms** | |
| --- | --- | --- | --- | --- | --- | --- | --- | --- | --- | --- | --- | --- | --- | --- | --- | --- | --- |
|  | **H_a_** | **H_b_** | **r_ab_**  **Theor. (Å)** | **r_ab_ (Å)** | **Error (%)** | **r_ab_ (Å)** | **Error (%)** | **r_ab_ (Å)** | **Error (%)** | **r_ab_ (Å)** | **Error (%)** | **r_ab_ (Å)** | **Error (%)** | **r_ab_ (Å)** | **Error (%)** | **r_ab_ (Å)** | **Error (%)** |
| **0.00-1.99** | **20a** | **11b/20b** | 1.75 | 1.84 | 5.4 | 2.35 | 34.1 | 1.86 | 6.3 | 1.86 | 6.2 | 1.89 | 8.1 | 1.89 | 7.9 | 1.89 | 7.9 |
|  | **11a** | **11b/20b** | 1.76 | 2.46 | 40.0 | 2.16 | 22.6 | 1.95 | 11.0 | 1.91 | 8.5 | 1.91 | 8.8 | 1.90 | 8.0 | 1.89 | 7.6 |
|  | **15a** | **15b** | 1.76 | 1.76 | 0.0 | 1.76 | 0.0 | 1.76 | 0.0 | 1.76 | 0.0 | 1.76 | 0.0 | 1.76 | 0.0 | 1.76 | 0.0 |
|  | **17a** | **17b** | 1.77 | 2.24 | 26.4 | 1.84 | 4.1 | 1.79 | 0.9 | 1.75 | 1.1 | 1.79 | 1.0 | 1.78 | 0.5 | 1.77 | 0.2 |
|  | **18a** | **18b** | 1.77 | 2.48 | 40.0 | 2.05 | 15.9 | 1.80 | 1.7 | 1.83 | 3.2 | 1.82 | 3.0 | 1.82 | 2.7 | 1.80 | 1.9 |
| **2.00-2.49** | **20a** | **15a** | 2.24 | 2.77 | 23.8 | 2.21 | 1.2 | 2.18 | 2.6 | 2.14 | 4.3 | 2.17 | 3.1 | 2.16 | 3.4 | 2.16 | 3.8 |
|  | **15b** | **13** | 2.26 | Phase |  | Phase |  | 2.89 | 27.8 | 2.90 | 28.4 | 2.75 | 21.6 | 2.49 | 10.3 | 2.53 | 11.9 |
|  | **12** | **23ab** | 2.27 | 2.88 | 27.0 | 2.40 | 5.6 | 2.35 | 3.4 | 2.31 | 1.6 | 2.33 | 2.8 | 2.33 | 2.9 | 2.32 | 2.0 |
|  | **22** | **23ab** | 2.30 | 2.96 | 28.9 | 2.50 | 8.9 | 2.43 | 5.7 | 2.40 | 4.2 | 2.43 | 5.5 | 2.41 | 4.6 | 2.39 | 4.0 |
|  | **12** | **11a** | 2.35 | Phase |  | 2.22 | 5.4 | 2.24 | 4.6 | 2.31 | 1.8 | 2.36 | 0.3 | 2.33 | 0.9 | 2.32 | 1.5 |
|  | **12** | **13** | 2.35 | 2.86 | 21.5 | 2.45 | 4.2 | 2.36 | 0.3 | 2.33 | 0.9 | 2.36 | 0.3 | 2.34 | 0.2 | 2.33 | 0.7 |
|  | **8** | **18b** | 2.37 | 2.77 | 17.1 | 2.32 | 2.2 | 2.26 | 4.7 | 2.22 | 6.2 | 2.25 | 5.0 | 2.24 | 5.6 | 2.23 | 6.0 |
|  | **1** | **16** | 2.38 | 2.83 | 18.8 | 2.36 | 1.0 | 2.29 | 3.7 | 2.25 | 5.3 | 2.28 | 4.1 | 2.27 | 4.6 | 2.26 | 4.9 |
|  | **22** | **11b/20b** | 2.39 | 3.12 | 30.7 | 2.60 | 8.9 | 2.54 | 6.2 | 2.51 | 4.9 | 2.54 | 6.4 | 2.53 | 5.8 | 2.52 | 5.5 |
|  | **14** | **13** | 2.41 | 2.93 | 21.5 | 2.52 | 4.6 | 2.44 | 1.4 | 2.40 | 0.4 | 2.43 | 0.9 | 2.42 | 0.3 | 2.41 | 0.1 |
|  | **8** | **17a** | 2.42 | 2.93 | 21.0 | 2.44 | 0.9 | 2.39 | 1.2 | 2.34 | 3.3 | 2.38 | 1.8 | 2.36 | 2.5 | 2.35 | 2.8 |
|  | **18a** | **17b** | 2.43 | 2.83 | 16.3 | 2.47 | 1.7 | 2.49 | 2.5 | 2.38 | 1.9 | 2.41 | 0.8 | 2.41 | 1.0 | 2.40 | 1.2 |
|  | **18b** | **17a** | 2.43 | 2.81 | 15.5 | 3.19 | 31.2 | 2.39 | 1.7 | 2.46 | 1.3 | 2.46 | 1.1 | 2.45 | 0.7 | 2.44 | 0.6 |
|  | **18b** | **11b/20b** | 2.45 | Phase |  | Phase |  | 2.82 | 15.0 | 2.69 | 10.0 | 2.72 | 10.9 | 2.78 | 13.5 | 2.80 | 14.2 |
|  | **3** | **2** | 2.48 | 2.90 | 16.8 | 3.11 | 25.3 | 2.33 | 6.0 | 2.75 | 11.0 | 2.58 | 4.0 | 2.75 | 11.0 | 2.44 | 1.7 |
|  | **16** | **15a** | 2.48 | 2.86 | 15.3 | 2.60 | 4.8 | 2.40 | 3.3 | 2.42 | 2.4 | 2.46 | 0.9 | 2.45 | 1.0 | 2.46 | 1.0 |
|  | **16** | **15b** | 2.48 | Phase |  | 2.58 | 4.1 | 2.43 | 2.1 | 2.39 | 3.7 | 2.44 | 1.6 | 2.44 | 1.8 | 2.44 | 1.6 |
|  | **14** | **15a** | 2.48 | 2.96 | 19.2 | 2.55 | 2.9 | 2.48 | 0.1 | 2.42 | 2.2 | 2.46 | 0.6 | 2.45 | 1.1 | 2.45 | 1.2 |
|  | **1** | **2** | 2.49 | 2.66 | 6.8 | 2.27 | 8.9 | 2.73 | 9.8 | 2.45 | 1.6 | 2.80 | 12.6 | 2.43 | 2.5 | 2.44 | 1.9 |
| **2.50-2.99** | **4** | **3** | 2.50 | 2.60 | 3.8 | 2.20 | 12.1 | Phase |  | 2.50 | 0.2 | 2.49 | 0.4 | 2.47 | 1.0 | 2.48 | 0.9 |
|  | **14** | **15b** | 2.55 | 3.58 | 40.5 | 2.51 | 1.7 | 2.49 | 2.2 | 2.47 | 3.2 | 2.53 | 0.8 | 2.53 | 0.7 | 2.54 | 0.4 |
|  | **23ab** | **14** | 2.59 | 3.18 | 22.7 | 2.67 | 3.0 | 2.59 | 0.2 | 2.55 | 1.5 | 2.59 | 0.1 | 2.56 | 1.0 | 2.56 | 1.3 |
|  | **8** | **11b/20b** | 2.63 | 3.43 | 30.3 | 2.76 | 4.8 | 2.70 | 2.6 | 2.65 | 0.7 | 2.69 | 2.1 | 2.66 | 1.2 | 2.66 | 1.0 |
|  | **16** | **17b** | 2.71 | 3.24 | 19.4 | 2.70 | 0.2 | 2.64 | 2.6 | 2.60 | 4.0 | 2.63 | 2.8 | 2.62 | 3.3 | 2.61 | 3.6 |
|  | **18a** | **17a** | 2.73 | 4.58 | 67.7 | 3.37 | 23.4 | 2.63 | 3.8 | 2.88 | 5.4 | 2.89 | 5.9 | 3.00 | 10.1 | 3.12 | 14.4 |
|  | **12** | **14** | 2.74 | 3.26 | 19.0 | 2.79 | 1.8 | 2.71 | 1.0 | 2.67 | 2.5 | 2.71 | 0.9 | 2.70 | 1.4 | 2.70 | 1.5 |
|  | **1** | **17b** | 2.79 | 3.39 | 21.4 | 2.82 | 1.0 | 2.75 | 1.3 | 2.70 | 3.3 | 2.75 | 1.6 | 2.72 | 2.3 | 2.72 | 2.3 |
|  | **18a** | **11b/20b** | 2.82 | 3.88 | 37.5 | 2.94 | 4.2 | 3.07 | 8.8 | 3.06 | 8.5 | 3.18 | 12.7 | 3.16 | 12.2 | 3.16 | 11.9 |
|  | **12** | **11b/20b** | 2.93 | Phase |  | 3.14 | 7.3 | 3.52 | 20.3 | 3.17 | 8.0 | 3.31 | 13.0 | 3.37 | 15.1 | 3.47 | 18.4 |
| **3.00-3.49** | **8** | **13** | 3.01 | 3.81 | 26.6 | 2.80 | 6.9 | 2.89 | 4.0 | 3.00 | 0.5 | 3.05 | 1.5 | 3.00 | 0.2 | 3.00 | 0.3 |
|  | **18b** | **17b** | 3.06 | 3.03 | 0.9 | 4.71 | 53.8 | 3.58 | 17.0 | 4.30 | 40.5 | 4.66 | 52.3 | 3.87 | 26.4 | 3.72 | 21.6 |
|  | **22** | **18b** | 3.16 | 3.73 | 18.0 | 3.10 | 1.9 | 3.00 | 5.2 | 2.97 | 5.9 | 3.02 | 4.4 | 3.00 | 4.9 | 2.99 | 5.4 |
|  | **23ab** | **11b/20b** | 3.24 | 4.66 | 43.8 | 3.61 | 11.3 | 3.53 | 9.1 | 3.50 | 8.1 | 3.66 | 13.1 | 3.57 | 10.2 | 3.63 | 12.1 |
|  | **20a** | **14** | 3.36 | 4.33 | 28.8 | 3.42 | 1.7 | 3.40 | 1.1 | 3.33 | 0.9 | 3.38 | 0.7 | 3.41 | 1.5 | 3.40 | 1.2 |
|  | **16** | **20a** | 3.37 | 3.74 | 11.1 | 3.45 | 2.3 | 3.59 | 6.5 | 3.40 | 0.8 | 3.39 | 0.5 | 3.49 | 3.6 | 3.42 | 1.4 |
|  | **22** | **20a** | 3.41 | 5.00 | 46.6 | 3.65 | 7.1 | 4.05 | 18.7 |  |  | 4.79 | 40.5 | 4.08 | 19.6 | 3.71 | 8.7 |
| **3.50-3.99** | **15a** | **13** | 3.54 | 2.61 | 26.3 | 3.61 | 2.1 | 5.41 | 52.9 | Phase |  | 3.43 | 3.2 | 3.25 | 8.3 | 3.16 | 10.6 |
|  | **20a** | **18b** | 3.60 | 3.02 | 16.1 | 3.04 | 15.6 | Phase |  | Phase |  | Phase |  | 4.05 | 12.6 | 3.73 | 3.6 |
|  | **8** | **17b** | 3.61 | 4.50 | 24.6 | 4.01 | 11.0 | 4.16 | 15.1 | 5.08 | 40.6 |  |  | 4.26 | 17.9 | 3.96 | 9.8 |
|  | **11b/20b** | **13** | 3.61 |  |  | 4.60 | 27.4 | 4.32 | 19.6 | 4.20 | 16.4 | 4.29 | 18.9 | 4.29 | 18.9 | 4.35 | 20.6 |
|  | **16** | **18a** | 3.62 | 4.43 | 22.3 | 3.74 | 3.2 | 3.70 | 2.3 | 3.65 | 0.7 | 3.71 | 2.4 | 3.68 | 1.7 | 3.72 | 2.8 |
|  | **12** | **8** | 3.65 | 5.20 | 42.4 | 3.73 | 2.3 | 3.74 | 2.4 | 3.68 | 0.8 | 3.72 | 2.0 | 3.79 | 3.8 | 3.72 | 1.9 |
|  | **11a** | **13** | 3.66 | 4.75 | 29.7 | 3.76 | 2.7 | 3.62 | 1.1 | 3.62 | 1.0 | 3.62 | 1.1 | 3.64 | 0.6 | 3.68 | 0.6 |
|  | **11b/20b** | **15a** | 3.69 | Phase |  | Phase |  | 3.67 | 0.5 | 3.46 | 6.2 | 3.38 | 8.3 | 3.20 | 13.3 | 3.14 | 14.9 |
|  | **23ab** | **8** | 3.73 | Phase |  | 4.12 | 10.4 | 5.27 | 41.3 | 4.67 | 25.1 | 3.90 | 4.5 | 4.11 | 10.1 | 3.88 | 4.1 |
|  | **20a** | **15b** | 3.75 | Phase |  | 4.46 | 19.0 | 3.62 | 3.4 | 3.26 | 13.0 | 3.20 | 14.6 | 3.09 | 17.7 | 3.00 | 20.0 |
|  | **23ab** | **13** | 3.76 | Phase |  | Phase |  | 5.23 | 39.0 | Phase |  |  |  | 4.62 | 22.8 | 4.44 | 18.0 |
|  | **20a** | **18a** | 3.81 | Phase |  | 4.27 | 12.0 | 4.43 | 16.2 | Phase |  | Phase |  | Phase |  | 5.37 | 41.1 |
|  | **8** | **18a** | 3.83 | Phase |  | Phase |  | 4.01 | 4.6 | 3.63 | 5.2 | 3.49 | 8.8 | 3.32 | 13.3 | 3.21 | 16.2 |
|  | **16** | **17a** | 3.84 | 4.76 | 23.9 | 4.13 | 7.6 | 4.74 | 23.6 | 4.58 | 19.2 | Phase |  | Phase |  | 4.41 | 15.0 |
|  | **1** | **15b** | 3.86 | 4.97 | 28.6 | 3.81 | 1.3 | 3.95 | 2.5 | 3.84 | 0.5 | 4.10 | 6.2 | 3.97 | 3.0 | 4.26 | 10.3 |
|  | **22** | **14** | 3.86 | 4.73 | 22.5 | 4.08 | 5.8 | 3.86 | 0.1 | 3.86 | 0.0 | 3.91 | 1.2 | 3.91 | 1.3 | 3.97 | 2.7 |
|  | **8** | **11a** | 3.88 | 5.37 | 38.3 | 4.42 | 13.9 | 4.66 | 20.1 | Phase |  | Phase |  | 4.54 | 17.0 | 4.13 | 6.4 |
|  | **16** | **18b** | 3.89 | 4.51 | 15.9 | 3.91 | 0.6 | 4.05 | 4.0 | 3.94 | 1.3 | 3.96 | 1.7 | 4.11 | 5.7 | 4.17 | 7.3 |
|  | **22** | **8** | 3.96 | 4.72 | 19.2 | 4.01 | 1.4 | 3.95 | 0.2 | 3.90 | 1.4 | 3.86 | 2.5 | 3.87 | 2.2 | 3.89 | 1.8 |
|  | **23ab** | **11a** | 3.96 | Phase |  | 4.97 | 25.5 | 4.69 | 18.4 |  |  | 4.83 | 21.9 | 4.31 | 8.8 | 4.09 | 3.4 |
|  | **8** | **14** | 3.96 |  |  | 4.44 | 12.2 | 4.22 | 6.6 | 3.95 | 0.2 | 4.06 | 2.6 | 4.07 | 2.9 | 4.09 | 3.3 |
|  | **14** | **11b/20b** | 3.96 | Phase |  | 4.81 | 21.4 | 4.78 | 20.7 | Phase |  | 5.34 | 34.8 | Phase |  | 5.44 | 37.3 |
| **4.00-4.49** | **1** | **17a** | 4.06 | 5.02 | 23.7 | 4.39 | 8.1 | 5.09 | 25.5 | 4.85 | 19.4 | Phase |  | Phase |  | 4.60 | 13.4 |
|  | **16** | **13** | 4.06 | 3.71 | 8.6 | 3.84 | 5.4 | 4.13 | 1.6 | 4.46 | 9.9 | 4.37 | 7.7 |  |  | 5.16 | 27.1 |
|  | **16** | **11b/20b** | 4.07 | Phase |  | 4.71 | 15.8 | 4.59 | 12.9 | 5.04 | 23.8 | 4.79 | 17.7 | 4.88 | 19.9 |  |  |
|  | **8** | **15b** | 4.09 | 4.76 | 16.4 | 3.31 | 19.0 | Phase |  | 4.29 | 5.0 | 3.95 | 3.3 | 5.03 | 23.1 | 4.27 | 4.5 |
|  | **16** | **8** | 4.14 | 3.51 | 15.2 | Phase |  | 4.98 | 20.4 | Phase |  | Phase |  | Phase |  | Phase |  |
|  | **11b/20b** | **17a** | 4.17 | Phase |  | Phase |  |  |  | Phase |  | 5.09 | 22.0 | 4.71 | 13.0 | 4.55 | 9.0 |
|  | **12** | **15b** | 4.27 |  |  | 4.11 | 3.6 | Phase |  | Phase |  |  |  | 5.13 | 20.2 | 4.28 | 0.3 |
|  | **23ab** | **18b** | 4.27 |  |  | 5.05 | 18.3 | 4.51 | 5.6 | 5.06 | 18.6 | 5.17 | 21.1 | 4.88 | 14.3 | 5.30 | 24.1 |
|  | **1** | **3** | 4.30 | 3.77 | 12.3 | 2.96 | 31.1 | 4.26 | 1.0 | 4.00 | 6.9 | 3.34 | 22.2 | 4.10 | 4.7 | 3.60 | 16.3 |
|  | **16** | **14** | 4.30 | Phase |  | Phase |  | 4.58 | 6.5 | 5.21 | 21.1 | Phase |  | Phase |  | Phase |  |
|  | **4** | **2** | 4.32 | 3.92 | 9.2 | 3.26 | 24.5 | 3.70 | 14.5 | Phase |  | 4.23 | 2.0 | 4.80 | 11.1 | 4.79 | 10.9 |
|  | **4** | **13** | 4.43 |  |  | 4.60 | 3.9 | 4.71 | 6.4 | 4.34 | 2.0 | 4.47 | 0.9 | 4.40 | 0.8 | 4.42 | 0.2 |
|  | **23ab** | **20a** | 4.44 | Phase |  | Phase |  | Phase |  | Phase |  | 4.70 | 5.9 | Phase |  | 5.40 | 21.5 |
|  | **22** | **18a** | 4.47 | Phase |  |  |  | Phase |  | Phase |  | 5.22 | 16.8 | 4.86 | 8.8 | 4.70 | 5.2 |
|  | **11b/20b** | **17b** | 4.49 | 4.86 | 8.1 | 5.05 | 12.5 | 5.50 | 22.4 |  |  | 5.06 | 12.8 | 5.41 | 20.5 | 5.22 | 16.2 |
| **4.50-4.99** | **18b** | **15a** | 4.55 | Phase |  | Phase |  | Phase |  | Phase |  | Phase |  | Phase |  |  |  |
|  | **11b/20b** | **15b** | 4.59 | 4.48 | 2.5 | 4.72 | 2.8 |  |  |  |  | Phase |  |  |  | Phase |  |
|  | **1** | **15a** | 4.62 |  |  | 5.26 | 13.9 | Phase |  | 5.38 | 16.3 | 4.92 | 6.4 | 4.61 | 0.2 | 4.22 | 8.6 |
|  | **8** | **15a** | 4.63 | Phase |  | 4.73 | 2.1 |  |  | 4.88 | 5.5 | 4.82 | 4.2 | 4.85 | 4.8 | 4.75 | 2.6 |
|  | **17b** | **15b** | 4.64 | Phase |  | Phase |  | Phase |  | Phase |  | Phase |  | Phase |  |  |  |
|  | **22** | **12** | 4.65 | Phase |  | Phase |  | Phase |  | Phase |  |  |  | Phase |  |  |  |
|  | **14** | **18b** | 4.66 | Phase |  | Phase |  | 4.54 | 2.6 | Phase |  | 4.40 | 5.5 | 4.63 | 0.6 | 4.11 | 11.8 |
|  | **23ab** | **15a** | 4.68 | Phase |  | Phase |  | Phase |  | 4.77 | 2.0 | Phase |  | 4.67 | 0.2 | 4.91 | 4.8 |
|  | **11a** | **17a** | 4.68 | 4.58 | 2.2 | 4.58 | 2.1 | Phase |  | Phase |  |  |  | Phase |  | 5.50 | 17.5 |
|  | **8** | **20a** | 4.71 | Phase |  | Phase |  | Phase |  | Phase |  | 4.86 | 3.3 | Phase |  | Phase |  |
|  | **18b** | **13** | 4.73 | Phase |  | 5.12 | 8.2 | Phase |  | Phase |  | Phase |  | Phase |  |  |  |
|  | **2** | **16** | 4.75 | Phase |  | Phase |  |  |  | 5.50 | 15.8 | Phase |  | 5.38 | 13.2 | 4.87 | 2.4 |
|  | **4** | **11a** | 4.77 | Phase |  | 4.81 | 0.8 | 4.79 | 0.3 | 4.95 | 3.8 | 4.72 | 1.0 | 4.94 | 3.5 | 4.60 | 3.6 |
|  | **11a** | **18b** | 4.77 | Phase |  | Phase |  | Phase |  | Phase |  | Phase |  | Phase |  | 5.44 | 14.1 |
|  | **17a** | **13** | 4.79 | Phase |  | Phase |  | Phase |  | Phase |  | Phase |  | Phase |  | Phase |  |
|  | **22** | **15a** | 4.81 | Phase |  | Phase |  |  |  | 5.43 | 12.8 | 5.46 | 13.5 | 5.34 | 11.1 | 4.94 | 2.8 |
|  | **23ab** | **15b** | 4.81 | 5.43 | 13.0 | Phase |  |  |  | 5.13 | 6.6 | Phase |  | Phase |  | 4.91 | 2.1 |
|  | **2** | **17b** | 4.82 |  |  | Phase |  | 5.30 | 9.9 |  |  | 5.41 | 12.2 |  |  |  |  |
|  | **18b** | **15b** | 4.83 | 4.70 | 2.6 | Phase |  | Phase |  | Phase |  |  |  | 5.39 | 11.6 |  |  |
|  | **14** | **11a** | 4.87 | Phase |  | Phase |  | Phase |  | Phase |  | Phase |  | Phase |  | 4.83 | 0.9 |
|  | **15a** | **17b** | 4.87 | Phase |  | Phase |  | Phase |  | Phase |  | Phase |  | Phase |  | Phase |  |
|  | **20a** | **13** | 4.88 | Phase |  | 4.88 | 0.0 | 5.45 | 11.6 | Phase |  | Phase |  | Phase |  | Phase |  |
|  | **4** | **8** | 4.89 | Phase |  | 4.86 | 0.7 | 4.77 | 2.5 | 4.71 | 3.6 | 4.95 | 1.2 | 4.78 | 2.3 | 4.88 | 0.3 |
|  | **4** | **11b/20b** | 4.89 | Phase |  |  |  | Phase |  | 5.33 | 9.0 |  |  |  |  |  |  |
|  | **18a** | **15a** | 4.89 | Phase |  | Phase |  | 5.00 | 2.2 |  |  | 5.26 | 7.5 |  |  | 4.65 | 5.0 |
|  | **1** | **18a** | 4.90 |  |  | 4.94 | 0.8 | Phase |  |  |  |  |  |  |  |  |  |
|  | **20a** | **17b** | 4.90 | Phase |  | Phase |  |  |  | Phase |  |  |  |  |  | 5.27 | 7.5 |
|  | **1** | **13** | 4.98 | Phase |  |  |  | 4.86 | 2.5 |  |  | 4.98 | 0.1 | 5.28 | 6.1 | 5.17 | 3.8 |
|  | **12** | **15a** | 4.98 | Phase |  |  |  | Phase |  |  |  |  |  | 5.23 | 5.0 | 5.27 | 5.8 |
| **5.00-5.49** | **4** | **1** | 5.00 | 4.30 | 14.1 | Phase |  | Phase |  | 5.15 | 3.1 | Phase |  | Phase |  | 5.14 | 2.8 |
|  | **17a** | **13** | 5.05 | Phase |  | Phase |  | Phase |  | Phase |  | Phase |  | Phase |  |  |  |
|  | **1** | **8** | 5.06 | Phase |  | 4.80 | 5.0 | 5.33 | 5.4 | 5.32 | 5.2 | 4.73 | 6.5 | 5.41 | 7.0 | 4.72 | 6.8 |
|  | **4** | **17a** | 5.17 |  |  | Phase |  | 5.20 | 0.6 | 4.75 | 8.2 | 5.03 | 2.8 | 5.35 | 3.6 | 4.96 | 4.0 |
|  | **17b** | **13** | 5.17 | Phase |  | Phase |  | Phase |  | Phase |  | Phase |  | Phase |  | Phase |  |
|  | **22** | **13** | 5.19 |  |  | 5.17 | 0.5 | Phase |  |  |  |  |  |  |  | Phase |  |
|  | **1** | **18b** | 5.32 |  |  |  |  | Phase |  | Phase |  | Phase |  | Phase |  | Phase |  |
|  | **20a** | **17a** | 5.35 |  |  | Phase |  | Phase |  |  |  |  |  |  |  | Phase |  |
|  | **12** | **18b** | 5.38 | 5.11 | 5.1 | 4.63 | 14.0 |  |  | Phase |  | 5.13 | 4.7 |  |  |  |  |
|  | **18a** | **15b** | 5.39 | Phase |  | Phase |  | Phase |  | Phase |  |  |  | Phase |  | Phase |  |
|  | **22** | **17a** | 5.41 | Phase |  | Phase |  | Phase |  |  |  | Phase |  |  |  |  |  |
|  | **2** | **15b** | 5.44 | Phase |  | Phase |  |  |  | 4.74 | 12.9 | Phase |  |  |  | 5.46 | 0.3 |
|  | **15a** | **17a** | 5.46 | Phase |  | Phase |  | Phase |  | Phase |  | Phase |  | Phase |  | Phase |  |
| **5.50-5.99** | **4** | **15b** | 5.53 | 5.35 | 3.3 | 5.08 | 8.2 | 5.28 | 4.6 | 5.43 | 1.8 |  |  |  |  |  |  |
|  | **22** | **15b** | 5.56 | Phase |  | Phase |  |  |  | Phase |  |  |  | Phase |  | Phase |  |
|  | **2** | **17a** | 5.67 |  |  | Phase |  | Phase |  | 5.26 | 7.3 | Phase |  |  |  |  |  |
|  | **11a** | **15b** | 5.69 |  |  | 4.83 | 15.2 | Phase |  | 4.73 | 16.9 |  |  |  |  |  |  |
|  | **4** | **17b** | 5.70 |  |  |  |  |  |  | Phase |  |  |  |  |  | Phase |  |
|  | **12** | **20a** | 5.70 | Phase |  | Phase |  | Phase |  | Phase |  | Phase |  | Phase |  | Phase |  |
|  | **23ab** | **17a** | 5.70 | Phase |  | Phase |  | Phase |  | Phase |  | Phase |  | Phase |  | 5.40 | 5.2 |
|  | **22** | **16** | 5.71 | Phase |  | Phase |  |  |  |  |  | Phase |  | Phase |  |  |  |
|  | **23ab** | **18a** | 5.72 | Phase |  | 5.22 | 8.7 |  |  | 5.32 | 7.0 | 5.50 | 3.8 | 5.03 | 12.1 | Phase |  |
|  | **1** | **20a** | 5.74 | Phase |  | Phase |  |  |  | Phase |  | Phase |  | Phase |  |  |  |
|  | **14** | **18a** | 5.79 | Phase |  | Phase |  | 4.08 | 29.5 | 4.10 | 29.2 | Phase |  | Phase |  | 3.18 | 45.0 |
|  | **14** | **17a** | 5.79 | Phase |  | Phase |  |  |  | Phase |  |  |  | 5.31 | 8.4 |  |  |
|  | **4** | **12** | 5.87 | 5.26 | 10.4 |  |  | 5.47 | 6.8 |  |  |  |  | Phase |  | Phase |  |
|  | **1** | **11b/20b** | 5.89 |  |  |  |  |  |  |  |  |  |  | Phase |  | Phase |  |
|  | **18a** | **13** | 5.89 | Phase |  | Phase |  | Phase |  | 5.15 | 12.6 | Phase |  | Phase |  | Phase |  |
|  | **3** | **13** | 5.90 |  |  | Phase |  | Phase |  |  |  |  |  |  |  |  |  |
|  | **23ab** | **16** | 5.93 | Phase |  | Phase |  | 4.10 | 30.8 | Phase |  | Phase |  | 4.09 | 31.1 | Phase |  |
|  | **3** | **17b** | 5.95 | Phase |  | Phase |  |  |  | Phase |  | Phase |  | Phase |  | Phase |  |
|  | **14** | **17b** | 5.95 |  |  | 5.45 | 8.4 | Phase |  |  |  | Phase |  |  |  | Phase |  |
| **6.00-6.49** | **12** | **17a** | 6.01 | Phase |  |  |  |  |  | Phase |  |  |  | Phase |  |  |  |
|  | **22** | **17b** | 6.03 |  |  |  |  | Phase |  | Phase |  | Phase |  |  |  |  |  |
|  | **12** | **16** | 6.03 | Phase |  | Phase |  | Phase |  | Phase |  | Phase |  | Phase |  | Phase |  |
|  | **4** | **16** | 6.07 | 5.23 | 13.9 | 5.38 | 11.4 | Phase |  | 5.44 | 10.4 |  |  |  |  |  |  |
|  | **3** | **17a** | 6.09 | Phase |  | Phase |  |  |  | Phase |  | Phase |  |  |  | Phase |  |
|  | **3** | **15b** | 6.11 | Phase |  | Phase |  | Phase |  | Phase |  |  |  | Phase |  |  |  |
|  | **2** | **13** | 6.11 | Phase |  |  |  |  |  |  |  |  |  |  |  |  |  |
|  | **11a** | **17b** | 6.11 | Phase |  | Phase |  | Phase |  | 4.97 | 18.7 |  |  | Phase |  | Phase |  |
|  | **1** | **14** | 6.12 |  |  | Phase |  |  |  | Phase |  |  |  | Phase |  |  |  |
|  | **22** | **11a** | 6.14 |  |  | Phase |  |  |  |  |  |  |  |  |  | Phase |  |
|  | **3** | **16** | 6.16 | Phase |  |  |  | 5.36 | 13.1 | Phase |  | Phase |  |  |  |  |  |
| **6.50-6.99** | **23ab** | **17b** | 6.50 | Phase |  | 5.21 | 19.8 | Phase |  | 5.37 | 17.3 | Phase |  | 5.50 | 15.4 | Phase |  |
|  | **3** | **8** | 6.53 |  |  |  |  | Phase |  |  |  |  |  | Phase |  |  |  |
|  | **11a** | **15a** | 6.53 | Phase |  | Phase |  | Phase |  | Phase |  | Phase |  |  |  |  |  |
|  | **2** | **8** | 6.59 |  |  |  |  | Phase |  |  |  |  |  |  |  | Phase |  |
|  | **2** | **15a** | 6.64 | Phase |  | Phase |  | Phase |  | Phase |  |  |  | Phase |  | Phase |  |
|  | **4** | **14** | 6.82 |  |  | Phase |  |  |  | Phase |  | Phase |  |  |  |  |  |
|  | **12** | **17b** | 6.83 | Phase |  | 5.36 | 21.5 | 5.45 | 20.2 |  |  | Phase |  |  |  |  |  |
|  | **4** | **18b** | 6.88 | 4.97 | 27.8 | 5.49 | 20.2 |  |  | Phase |  |  |  |  |  |  |  |
|  | **12** | **18a** | 6.94 | 4.15 | 40.2 | 3.89 | 43.9 | 4.26 | 38.7 | Phase |  | Phase |  | Phase |  | 5.33 | 23.1 |
| **7.00-7.49** | **3** | **11b/20b** | 7.02 | Phase |  | 5.28 | 24.8 |  |  | 5.16 | 26.4 |  |  |  |  | Phase |  |
|  | **16** | **11a** | 7.08 | Phase |  | Phase |  | 5.30 | 25.1 | Phase |  | Phase |  | Phase |  | Phase |  |
|  | **4** | **15a** | 7.13 |  |  |  |  | Phase |  |  |  |  |  | Phase |  | Phase |  |
|  | **3** | **11a** | 7.13 | Phase |  |  |  | 5.16 | 27.6 | Phase |  |  |  |  |  | Phase |  |
|  | **2** | **18a** | 7.16 | 5.39 | 24.8 | Phase |  | Phase |  | Phase |  | Phase |  |  |  |  |  |
|  | **4** | **23ab** | 7.21 | Phase |  | Phase |  | Phase |  |  |  | Phase |  | Phase |  | Phase |  |
|  | **1** | **12** | 7.22 |  |  |  |  | Phase |  | Phase |  |  |  |  |  | Phase |  |
|  | **2** | **18b** | 7.42 |  |  |  |  |  |  |  |  | Phase |  | Phase |  |  |  |
|  | **2** | **11b/20b** | 7.43 | Phase |  | 4.84 | 34.8 |  |  | Phase |  | 5.45 | 26.6 | Phase |  |  |  |
|  | **20a** | **11a** | 7.48 | 4.81 | 35.7 | Phase |  | Phase |  | Phase |  | Phase |  | Phase |  | Phase |  |
| **7.50-7.99** | **1** | **23ab** | 7.50 | Phase |  |  |  | Phase |  | Phase |  |  |  | Phase |  | 5.45 | 27.4 |
|  | **4** | **18a** | 7.60 | Phase |  | Phase |  | Phase |  |  |  | Phase |  | Phase |  | Phase |  |
|  | **1** | **11a** | 7.64 |  |  |  |  | Phase |  | Phase |  |  |  |  |  |  |  |
|  | **3** | **15a** | 7.67 |  |  | Phase |  |  |  |  |  | Phase |  | Phase |  | Phase |  |
|  | **18a** | **11a** | 7.69 | Phase |  | 3.31 | 57.0 | 3.36 | 56.3 | Phase |  | Phase |  | 3.60 | 53.1 | 3.07 | 60.1 |
|  | **1** | **22** | 7.71 | Phase |  |  |  | 5.21 | 32.4 | Phase |  | Phase |  | Phase |  |  |  |
|  | **2** | **14** | 7.80 |  |  |  |  | Phase |  |  |  |  |  | Phase |  |  |  |
|  | **3** | **12** | 7.83 |  |  | Phase |  |  |  | Phase |  | Phase |  |  |  |  |  |
| **8.00-8.49** | **3** | **18b** | 8.03 | Phase |  |  |  |  |  | 5.48 | 31.8 | Phase |  | Phase |  | Phase |  |
|  | **3** | **14** | 8.08 | Phase |  | Phase |  |  |  |  |  |  |  | Phase |  | Phase |  |
|  | **2** | **20a** | 8.09 |  |  |  |  |  |  | Phase |  |  |  |  |  |  |  |
|  | **3** | **18a** | 8.25 | Phase |  | Phase |  |  |  |  |  |  |  |  |  | Phase |  |
|  | **2** | **11a** | 8.28 | 5.17 | 37.6 |  |  | Phase |  | Phase |  | Phase |  |  |  |  |  |
|  | **2** | **12** | 8.37 | Phase |  | Phase |  | 5.35 | 36.1 |  |  | Phase |  | Phase |  | Phase |  |
|  | **4** | **20a** | 8.46 |  |  |  |  | Phase |  | Phase |  |  |  | Phase |  | Phase |  |
| **8.50-8.99** | **4** | **22** | 8.70 |  |  | Phase |  | 5.09 | 41.5 |  |  | Phase |  | Phase |  |  |  |
|  | **3** | **23ab** | 8.88 | 5.31 | 40.2 | 5.20 | 41.4 |  |  | Phase |  | Phase |  |  |  | Phase |  |
|  | **2** | **23ab** | 8.99 |  |  |  |  | 5.28 | 41.2 | Phase |  | Phase |  |  |  |  |  |
| **9.00-9.49** | **3** | **20a** | 9.19 | Phase |  |  |  |  |  |  |  |  |  |  |  |  |  |
| **9.50-9.99** | **2** | **22** | 9.82 |  |  | Phase |  | Phase |  | Phase |  |  |  |  |  |  |  |
| **10.00+** | **3** | **22** | 10.22 |  |  | Phase |  |  |  |  |  |  |  | Phase |  | Phase |  |

**Table S32**. Average absolute error of the quantified distances based on all 7 spectra recorded at different mixing times (100-700 ms) for the NOESY sequence (noesyph). Same distances quantified from the same spectra excluding one of the mixing times. The difference between the original average absolute error and average absolute errors obtained when including only 6 different mixing times are given in-between brackets.

|  | **Distance range (Å)** |  | **Error** | **Removed mixing time** | | | | | | |
| --- | --- | --- | --- | --- | --- | --- | --- | --- | --- | --- |
|  |  |  | **All** | **100** | **200** | **300** | **400** | **500** | **600** | **700** |
| **Short reliable** | **0.00-1.99** | **Min** | 1.1 | 1.1  (0.0) | 0.6  (-0.5) | 1.1  (0.0) | 1.1  (0.0) | 1.1  (0.0) | 1.1  (0.0) | 1.1  (0.0) |
|  |  | **Average** | 2.5 | 1.9  (-0.6) | 2.3  (-0.2) | 2.5  (0.0) | 2.5  (0.0) | 2.5  (0.0) | 2.5  (0.0) | 2.3  (-0.2) |
|  |  | **Max** | 4.5 | 3.4  (-1.1) | 5.1  (0.6) | 4.5  (0.0) | 4.5  (0.0) | 4.5  (0.0) | 4.5  (0.0) | 4.0  (-0.5) |
|  | **2.00-2.49** | **Min** | 0.9 | 0.9  (0.0) | 0.4  (-0.5) | 0.9  (0.0) | 0.9  (0.0) | 0.9  (0.0) | 0.4  (-0.5) | 0.9  (0.0) |
|  |  | **Average** | 3.4 | 3.5  (0.0) | 3.3  (-0.1) | 3.4  (0.0) | 3.4  (0.0) | 3.4  (0.0) | 3.4  (0.0) | 3.4  (0.0) |
|  |  | **Max** | 7.6 | 7.6  (0.0) | 7.6  (0.0) | 7.6  (0.0) | 7.6  (0.0) | 7.6  (0.0) | 7.6  (0.0) | 7.2  (-0.4) |
|  | **2.50-2.99** | **Min** | 0.8 | 0.4  (-0.4) | 1.1  (0.3) | 0.8  (0.0) | 0.8  (0.0) | 0.8  (0.0) | 0.8  (0.0) | 1.1  (0.3) |
|  |  | **Average** | 2.8 | 2.5  (-0.3) | 3.0  (0.2) | 2.9  (0.1) | 2.8  (0.0) | 2.8  (0.0) | 2.9  (0.1) | 3.2  (0.4) |
|  |  | **Max** | 4.8 | 4.8  (0.0) | 4.8  (0.0) | 4.8  (0.0) | 4.8  (0.0) | 4.8  (0.0) | 4.8  (0.0) | 5.2  (0.4) |
| **Long reliable** | **3.00-3.49** | **Min** | 0.0 | 1.2  (1.2) | 0.6  (0.6) | 0.0  (0.0) | 0.0  (0.0) | 0.0  (0.0) | 0.3  (0.3) | 0.9  (0.9) |
|  |  | **Average** | 5.8 | 6.9  (1.1) | 5.8  (0.0) | 5.6  (-0.2) | 5.8  (0.0) | 5.8  (0.0) | 6.0  (0.2 | 5.2  (-0.6) |
|  |  | **Max** | 10.5 | 13.0  (2.5) | 10.2  (-0.3) | 10.2  (-0.3) | 10.5  (0.0) | 10.2  (-0.3) | 11.1  (0.6) | 9.0  (-1.5) |
|  | **3.50-3.99** | **Min** | 0.5 | 0.0  (0.5) | 0.0  (0.5) | 0.5  (0.0) | 0.5  (0.0) | 0.5  (0.0) | 0.3  (-0.2) | 0.5  (0.0) |
|  |  | **Average** | 3.9 | 4.7  (0.8) | 3.9  (0.0) | 3.8  (-0.1) | 3.9  (0.0) | 4.0  (0.1) | 4.0  (0.1) | 3.6  (-0.3) |
|  |  | **Max** | 16.1 | 18  (1.9) | 16.1  (0.0) | 15.8  (-0.3) | 16.1  (0.0) | 16.3  (0.2) | 16.1  (0.0) | 13.6  (-2.5) |
|  | **4.00-4.49** | **Min** | 3.2 | 2.9  (-0.3) | 3.6  (0.4) | 2.7  (-0.5) | 3.2  (0.0) | 3.2  (0.0) | 2.9  (-0.3) | 3.8  (0.6) |
|  |  | **Average** | 3.2 | 2.9  (-0.3) | 3.6  (0.4) | 2.7  (-0.5) | 3.2  (0.0) | 3.2  (0.0) | 2.9  (-0.3) | 3.8  (0.6) |
|  |  | **Max** | 3.2 | 2.9  (-0.3) | 3.6  (0.4) | 2.7  (-0.5) | 3.2  (0.0) | 3.2  (0.0) | 2.9  (-0.3) | 3.8  (0.6) |
| **At the NOE limit** | **4.50-4.99** | **Min** | n.a. | n.a. | n.a. | n.a. | n.a. | n.a. | n.a. | n.a. |
|  |  | **Average** | n.a. | n.a. | n.a. | n.a. | n.a. | n.a. | n.a. | n.a. |
|  |  | **Max** | n.a. | n.a. | n.a. | n.a. | n.a. | n.a. | n.a. | n.a. |
|  | **0.00-4.99** | **Min** | 0.0 | 0.0  (0.0) | 0.0  (0.0) | 0.0  (0.0) | 0.0  (0.0) | 0.0  (0.0) | 0.3  (0.3) | 0.5  (0.5) |
|  |  | **Average** | 3.6 | 3.8  (0.2) | 3.6  (0.0) | 3.6  (0.0) | 3.6  (0.0) | 3.6  (0.0) | 3.6 (0.0) | 3.5  (-0.1) |
|  |  | **Max** | 16.1 | 18  (1.9) | 16.1  (0.0) | 15.8  (-0.3) | 16.1  (0.0) | 16.3  (0.2) | 16.1 (0.0) | 13.6  (-2.5) |

**Table S33**. Average absolute error of the quantified distances based on all 7 spectra recorded at different mixing times (100-700 ms) for the NOESY ES (noesyesgpph). Same distances quantified from the same spectra excluding one of the mixing times. The difference between the original average absolute error and average absolute errors obtained when including only 6 different mixing times are given in-between brackets.

|  | **Distance range (Å)** |  | **Error** | **Removed mixing time** | | | | | | |
| --- | --- | --- | --- | --- | --- | --- | --- | --- | --- | --- |
|  |  |  | **All** | **100** | **200** | **300** | **400** | **500** | **700** | **800** |
| **Short reliable** | **0.00-1.99** | **Min** | 0.0 | 2.3  (2.3) | 0.6  (0.6) | 0.0  (0.0) | 0.0  (0.0) | 0.0  (0.0) | 0.0  (0.0) | 0.6  (0.6) |
|  |  | **Average** | 2.0 | 3.1  (1.1) | 2.3  (0.3) | 1.7  (-0.3) | 1.7  (-0.3) | 2.0  (0.0) | 1.7  (-0.3) | 1.7  (-0.3) |
|  |  | **Max** | 4.0 | 4.0  (0.0) | 4.0  (0.0) | 3.4  (-0.6) | 3.4  (-0.6) | 4.0  (0.0) | 3.4  (-0.6) | 2.8  (-1.2) |
|  | **2.00-2.49** | **Min** | 0.8 | 0.8  (0.0) | 0.8  (0.0) | 0.8  (0.0) | 0.8  (0.0) | 0.8  (0.0) | 0.8  (0.0) | 0.4  (-0.4) |
|  |  | **Average** | 6.5 | 6.4  (-0.1) | 6.5  (0.0) | 6.5  (0.0) | 6.6  (0.1) | 6.5  (0.0) | 6.5  (0.0) | 6.7  (0.2) |
|  |  | **Max** | 15.7 | 14.5  (-1.2) | 16.2  (0.5) | 15.7  (0.0) | 15.7  (0.0) | 15.7  (0.0) | 15.7  (0.0) | 15.3  (-0.4) |
|  | **2.50-2.99** | **Min** | 2.3 | 2.7  (0.4) | 2.3  (0.0) | 2.3  (0.0) | 2.3  (0.0) | 2.3  (0.0) | 2.3  (0.0) | 2.3  (0.0) |
|  |  | **Average** | 5.6 | 5.7  (0.1) | 5.6  (0.0) | 5.6  (0.0) | 5.6  (0.0) | 5.6  (0.0) | 5.6  (0.0) | 5.6  (0.0) |
|  |  | **Max** | 7.7 | 7.7  (0.0) | 7.7  (0.0) | 7.7  (0.0) | 7.7  (0.0) | 7.7  (0.0) | 7.7  (0.0) | 7.7  (0.0) |
| **Long reliable** | **3.00-3.49** | **Min** | 8.9 | 8.5  (0.4) | 8.9  (0.0) | 8.9  (0.0) | 8.9  (0.0) | 8.9  (0.0) | 8.6  (-0.3) | 9.0  (0.1) |
|  |  | **Average** | 9.2 | 9.7  (0.5) | 9.1  (-0.1) | 9.2  (0.0) | 9.2  (0.0) | 9.4  (0.2) | 8.9  (-0.3) | 9.1  (-0.1) |
|  |  | **Max** | 9.6 | 10.8  (1.2) | 9.3  (-0.3) | 9.6  (0.0) | 9.6  (0.0) | 9.9  (-0.3) | 9.2  (-0.4) | 9.2  (-0.4) |
|  | **3.50-3.99** | **Min** | 5.8 | 6.3  (0.5) | 6.1  (0.3) | 5.8  (0.0) | 5.8  (0.0) | 5.8  (0.0) | 5.6  (-0.2) | 6.3  (0.5) |
|  |  | **Average** | 15.3 | 16.0  (0.7) | 15.3  (0.0) | 15.3  (0.0) | 15.3  (0.0) | 15.4  (0.1) | 14.8  (-0.5) | 15.7  (0.4) |
|  |  | **Max** | 27.2 | 26.9  (-0.3) | 26.4  (-0.8) | 27.2  (0.0) | 27.2  (0.0) | 26.9  (-0.3) | 26.9  (-0.3) | 28.5  (1.3) |
|  | **4.00-4.49** | **Min** | 7.2 | 7.9  (0.7) | 6.8  (-0.4) | 7.4  (0.2) | 7.4  (0.2) | 7.2  (0.0) | 7.4  (0.2) | 7.4  (0.2) |
|  |  | **Average** | 7.2 | 7.9  (0.7) | 6.8  (-0.4) | 7.4  (0.2) | 7.4  (0.2) | 7.2  (0.0) | 7.4  (0.2) | 7.4  (0.2) |
|  |  | **Max** | 7.2 | 7.9  (0.7) | 6.8  (-0.4) | 7.4  (0.2) | 7.4  (0.2) | 7.2  (0.0) | 7.4  (0.2) | 7.4  (0.2) |
| **At the NOE limit** | **4.50-4.99** | **Min** | n.a. | n.a. | n.a. | n.a. | n.a. | n.a. | n.a. | n.a. |
|  |  | **Average** | n.a. | n.a. | n.a. | n.a. | n.a. | n.a. | n.a. | n.a. |
|  |  | **Max** | n.a. | n.a. | n.a. | n.a. | n.a. | n.a. | n.a. | n.a. |
|  | **0.00-4.99** | **Min** | 0.0 | 0.8  (0.8) | 0.6  (0.6) | 0.0  (0.0) | 0.0  (0.0) | 0.0  (0.0) | 0.0  (0.0) | 0.4  (0.4) |
|  |  | **Average** | 7.5 | 7.8  (0.3) | 7.5  (0.0) | 7.5  (0.0) | 7.5  (0.0) | 7.5  (0.0) | 7.4  (-0.1) | 7.6  (0.1) |
|  |  | **Max** | 27.2 | 26.9  (-0.3) | 26.4  (-0.8) | 27.2  (0.0) | 27.2  (0.0) | 26.9  (-0.3) | 26.9  (-0.3) | 28.5  (1.3) |

**Table S34**. Average absolute error of the quantified distances based on all 7 spectra recorded at different mixing times (100-700 ms) for the NOESY W5 (noesydbphw5). Same distances quantified from the same spectra excluding one of the mixing times. The difference between the original average absolute error and average absolute errors obtained when including only 6 different mixing times are given in-between brackets.

|  | **Distance range (Å)** |  | **Error** | **Removed mixing time** | | | | | | |
| --- | --- | --- | --- | --- | --- | --- | --- | --- | --- | --- |
|  |  |  | **All** | **100** | **200** | **300** | **400** | **500** | **600** | **700** |
| **Short reliable** | **0.00-1.99** | **Min** | 0.0 | 0.6  (0.6) | 0.0  (0.0) | 0.0  (0.0) | 0.0  (0.0) | 0.0  (0.0) | 0.0  (0.0) | 0.0  (0.0) |
|  |  | **Average** | 3.0 | 2.3  (-0.7) | 3.4  (0.4) | 3.0  (0.0) | 3.0  (0.0) | 3.0  (0.0) | 3.0  (0.0) | 3.0  (0.0) |
|  |  | **Max** | 8.5 | 5.7  (-2.8) | 9.7  (1.2) | 8.5  (0.0) | 8.5  (0.0) | 8.5  (0.0) | 9.1  (0.6) | 8.5  (0.0) |
|  | **2.00-2.49** | **Min** | 0.4 | 0.0  (-0.4) | 0.0  (-0.4) | 0.4  (0.0) | 0.4  (0.0) | 0.4  (0.0) | 0.4  (0.0) | 0.0  (-0.4) |
|  |  | **Average** | 2.3 | 2.3  (0.0) | 2.3  (0.0) | 2.4  (0.1) | 2.3  (0.0) | 2.3  (0.0) | 2.3  (0.0) | 2.6  (0.3) |
|  |  | **Max** | 7.9 | 8.4  (0.5) | 7.9  (0.0) | 7.9  (0.0) | 7.9  (0.0) | 7.5  (-0.4) | 7.1  (-0.8) | 9.2  (1.3) |
|  | **2.50-2.99** | **Min** | 0.7 | 0.4  (-0.3) | 0.7  (0.0) | 0.7  (0.0) | 0.7  (0.0) | 0.7  (0.0) | 0.7  (0.0) | 0.7  (0.0) |
|  |  | **Average** | 1.8 | 1.4  (-0.4) | 1.8  (0.0) | 1.8  (0.0) | 1.8  (0.0) | 1.8  (0.0) | 1.7  (-0.1) | 1.6  (-0.2) |
|  |  | **Max** | 2.7 | 2.4  (-0.3) | 3.1  (0.4) | 2.7  (0.0) | 2.7  (0.0) | 2.7  (0.0) | 2.6  (-0.1) | 2.7  (0.0) |
| **Long reliable** | **3.00-3.49** | **Min** | 3.3 | 3.6  (0.3) | 3.6  (0.3) | 3.3  (0.0) | 3.3  (0.0) | 3.6  (0.3) | 3.9  (0.6) | 2.1  (-1.2) |
|  |  | **Average** | 7.1 | 7.4  (0.3) | 7.4  (0.3) | 7.0  (-0.1) | 7.1  (0.0) | 7.4  (0.3) | 7.6  (0.5) | 5.8  (-1.3) |
|  |  | **Max** | 12.7 | 13.6  (0.9) | 13.3  (0.6) | 12.3  (-0.4) | 12.7  (0.0) | 13.3  (0.6) | 13.6  (0.9) | 9.9  (-2.8) |
|  | **3.50-3.99** | **Min** | 1.0 | 0.5  (-0.5) | 0.8  (-0.2) | 1.0  (0.0) | 1.0  (0.0) | 1.0  (0.0) | 1.0  (0.0) | 1.0  (0.0) |
|  |  | **Average** | 4.4 | 4.9  (0.5) | 4.4  (0.0) | 4.3  (-0.1) | 4.4  (0.0) | 4.5  (0.1) | 4.4  (0.0) | 3.8  (-0.6) |
|  |  | **Max** | 12.5 | 14.1  (1.6) | 12.7  (0.2) | 12.5  (0.0) | 12.5  (0.0) | 12.7  (0.2) | 12.7  (0.2) | 10.5  (-2.0) |
|  | **4.00-4.49** | **Min** | 2.5 | 2.5  (0.0) | 2.7  (0.2) | 2.5  (0.0) | 2.5  (0.0) | 2.5  (0.0) | 2.7  (0.2) | 2.5  (0.0) |
|  |  | **Average** | 2.5 | 2.5  (0.0) | 2.7  (0.2) | 2.5  (0.0) | 2.5  (0.0) | 2.5  (0.0) | 2.7  (0.2) | 2.5  (0.0) |
|  |  | **Max** | 2.5 | 2.5  (0.0) | 2.7  (0.2) | 2.5  (0.0) | 2.5  (0.0) | 2.5  (0.0) | 2.7  (0.2) | 2.5  (0.0) |
| **At the NOE limit** | **4.50-4.99** | **Min** | 1.2 | 1.0  (-0.2) | 1.0  (-0.2) | 0.8  (-0.4) | 1.2  (0.0) | 1.0  (-0.2) | 1.4  (0.2) | 2.2  (1.0) |
|  |  | **Average** | 1.7 | 1.8  (0.1) | 1.5  (-0.2) | 1.2  (-0.5) | 1.7  (0.0) | 1.5  (-0.2) | 1.5  (-0.2) | 2.9  (1.2) |
|  |  | **Max** | 2.2 | 2.7  (0.5) | 2.0  (-0.2) | 1.6  (-0.6) | 2.2  (0.0) | 2.0  (-0.2) | 1.6  (-0.6) | 3.7  (1.5) |
|  | **0.00-4.99** | **Min** | 0.0 | 0.0  (0.0) | 0.0  (0.0) | 0.0  (0.0) | 0.0  (0.0) | 0.0  (0.0) | 0.0  (0.0) | 0.0  (0.0) |
|  |  | **Average** | 3.0 | 2.9  (-0.1) | 3.0  (0.0) | 2.9  (-0.1) | 3.0  (0.0) | 3.0  (0.0) | 3.0  (0.0) | 2.9  (-0.1) |
|  |  | **Max** | 12.7 | 14.1  (1.4) | 13.3  (0.6) | 12.5  (-0.2) | 12.7  (0.0) | 13.3  (0.6) | 13.6  (0.9) | 10.5  (-2.2) |

**Table S35**. Average absolute error of the quantified distances based on all 7 spectra recorded at different mixing times (100-700 ms) for the ROESY (roesyph). Same distances quantified from the same spectra excluding one of the mixing times. The difference between the original average absolute error and average absolute errors obtained when including only 6 different mixing times are given in-between brackets.

|  | **Distance range (Å)** |  | **Error** | **Removed mixing time** | | | | | | |
| --- | --- | --- | --- | --- | --- | --- | --- | --- | --- | --- |
|  |  |  | **All** | **50** | **100** | **150** | **200** | **250** | **300** | **350** |
| **Short reliable** | **0.00-1.99** | **Min** | 0.0 | 0.6  (0.6) | 1.1  (1.1) | 0.0  (0.0) | 0.0  (0.0) | 0.0  (0.0) | 0.0  (0.0) | 0.6  (0.6) |
|  |  | **Average** | 2.6 | 2.7  (0.1) | 3.0  (0.4) | 2.7  (0.1) | 2.6  (0.0) | 2.6  (0.0) | 2.6  (0.0) | 3.0  (0.4) |
|  |  | **Max** | 8.0 | 7.4  (-0.6) | 8.0  (0.0) | 8.0  (0.0) | 8.0  (0.0) | 8.0  (0.0) | 8.5  (0.5) | 8.0  (0.0) |
|  | **2.00-2.49** | **Min** | 0.4 | 0.4  (0.0) | 0.4  (0.0) | 0.4  (0.0) | 0.4  (0.0) | 0.4  (0.0) | 0.4  (0.0) | 0.4  (0.0) |
|  |  | **Average** | 3.5 | 3.6  (0.1) | 3.6  (0.1) | 3.5  (0.0) | 3.5  (0.0) | 3.5  (0.0) | 3.5  (0.0) | 3.6  (0.1) |
|  |  | **Max** | 11.3 | 11.3  (0.0) | 11.3  (0.0) | 11.3  (0.0) | 11.3  (0.0) | 11.3  (0.0) | 11.3  (0.0) | 11.7  (0.4) |
|  | **2.50-2.99** | **Min** | 1.5 | 0.8  (-0.7) | 1.1  (-0.4) | 1.5  (0.0) | 1.5  (0.0) | 1.5  (0.0) | 1.5  (0.0) | 0.8  (-0.7) |
|  |  | **Average** | 3.7 | 3.5  (-0.2) | 3.9  (0.2) | 3.7  (0.0) | 3.7  (0.0) | 3.8  (0.1) | 3.6  (-0.1) | 3.3  (-0.4) |
|  |  | **Max** | 7.0 | 8.4  (1.4) | 7.3  (0.3) | 6.6  (-0.4) | 7.0  (0.0) | 7.3  (0.3) | 7.0  (0.0) | 5.1  (-1.9) |
| **Long reliable** | **3.00-3.49** | **Min** | 1.6 | 1.3  (-0.3) | 1.6  (0.0) | 1.6  (0.0) | 1.6  (0.0) | 1.3  (-0.3) | 1.3  (-0.3) | 2.2  (0.6) |
|  |  | **Average** | 3.8 | 4.0  (0.2) | 3.9  (0.1) | 3.7  (-0.1) | 3.8  (0.0) | 3.8  (0.0) | 4.0  (0.2) | 3.5  (-0.3) |
|  |  | **Max** | 7.4 | 8.0  (0.6) | 8.0  (0.6) | 7.1  (-0.3) | 7.4  (0.0) | 7.7  (0.3) | 8.3  (0.9) | 5.9  (-1.5) |
|  | **3.50-3.99** | **Min** | 0.6 | 1.1  (0.5) | 0.6  (0.0) | 0.6  (0.0) | 0.6  (0.0) | 0.8  (0.2) | 1.1  (0.5) | 0.6  (0.0) |
|  |  | **Average** | 3.3 | 3.4  (0.1) | 2.8  (-0.5) | 3.3  (0.0) | 3.3  (0.0) | 3.4  (0.1) | 3.8  (0.5) | 3.3  (0.0) |
|  |  | **Max** | 5.2 | 4.8  (-0.4) | 4.9  (-0.3) | 5.2  (0.0) | 5.2  (0.0) | 5.2  (0.0) | 5.8  (0.6) | 5.5  (0.3) |
|  | **4.00-4.49** | **Min** | 37.9 | 38.4  (0.5) | 37.4  (-0.5) | 37.9  (0.0) | 37.9  (0.0) | 37.7  (-0.2) | 37.4  (-0.5) | 38.2  (0.3) |
|  |  | **Average** | 37.9 | 38.4  (0.5) | 37.4  (-0.5) | 37.9  (0.0) | 37.9  (0.0) | 37.7  (-0.2) | 37.4  (-0.5) | 38.2  (0.3) |
|  |  | **Max** | 37.9 | 38.4  (0.5) | 37.4  (-0.5) | 37.9  (0.0) | 37.9  (0.0) | 37.7  (-0.2) | 37.4  (-0.5) | 38.2  (0.3) |
| **At the NOE limit** | **4.50-4.99** | **Min** | n.a. | n.a. | n.a. | n.a. | n.a. | n.a. | n.a. | n.a. |
|  |  | **Average** | n.a. | n.a. | n.a. | n.a. | n.a. | n.a. | n.a. | n.a. |
|  |  | **Max** | n.a. | n.a. | n.a. | n.a. | n.a. | n.a. | n.a. | n.a. |
|  | **0.00-4.99** | **Min** | 0.0 | 0.4  (0.4) | 0.4  (0.4) | 0.0  (0.0) | 0.0  (0.0) | 0.0  (0.0) | 0.0  (0.0) | 0.4  (0.4) |
|  |  | **Average** | 4.4 | 4.5  (0.1) | 4.5  (0.1) | 4.4  (0.0) | 4.4  (0.0) | 4.4  (0.0) | 4.4  (0.0) | 4.4  (0.0) |
|  |  | **Max** | 37.9 | 38.4  (0.5) | 37.4  (-0.5) | 37.9  (0.0) | 37.9  (0.0) | 37.7  (-0.2) | 37.4  (-0.5) | 38.2  (0.3) |

**Table S36**. Average absolute error of the quantified distances based on all 7 spectra recorded at different mixing times (100-700 ms) for the EASY ROESY (roesyadesjsph). Same distances quantified from the same spectra excluding one of the mixing times. The difference between the original average absolute error and average absolute errors obtained when including only 6 different mixing times are given in-between brackets.

|  | **Distance range (Å)** |  | **Error** | **Removed mixing time** | | | | | | |
| --- | --- | --- | --- | --- | --- | --- | --- | --- | --- | --- |
|  |  |  | **All** | **50** | **100** | **150** | **200** | **250** | **300** | **350** |
| **Short reliable** | **0.00-1.99** | **Min** | 0.0 | 2.8  (2.8) | 0.6  (0.6) | 0.6  (0.6) | 0.0  (0.0) | 0.0  (0.0) | 0.6  (0.6) | 1.1  (1.1) |
|  |  | **Average** | 3.7 | 4.2  (0.5) | 4.5  (0.8) | 4.0  (0.3) | 3.7  (0.0) | 3.7  (0.0) | 4.0  (0.3) | 4.0  (0.3) |
|  |  | **Max** | 7.3 | 5.6  (-1.7) | 8.5  (1.2) | 7.3  (0.0) | 7.3  (0.0) | 7.3  (0.0) | 7.3  (0.0) | 6.8  (-0.5) |
|  | **2.00-2.49** | **Min** | 0.9 | 0.4  (-0.5) | 1.3  (0.4) | 0.9  (0.0) | 0.9  (0.0) | 0.9  (0.0) | 0.9  (0.0) | 0.8  (-0.1) |
|  |  | **Average** | 4.0 | 3.8  (-0.2) | 4.1  (0.1) | 4.1  (0.1) | 4.0  (0.0) | 4.0  (0.0) | 4.1  (0.1) | 4.1  (0.1) |
|  |  | **Max** | 11.7 | 11.3  (-0.4) | 12.2  (0.5) | 12.2  (0.5) | 11.7  (0.0) | 11.7  (0.0) | 12.2  (0.5) | 12.2  (0.5) |
|  | **2.50-2.99** | **Min** | 0.4 | 0.4  (0.0) | 0.4  (0.0) | 0.4  (0.0) | 0.4  (0.0) | 0.4  (0.0) | 0.4  (0.0) | 0.7  (0.3) |
|  |  | **Average** | 3.1 | 3.0  (-0.1) | 3.1  (0.0) | 3.1  (0.0) | 3.1  (0.0) | 3.1  (0.0) | 3.1  (0.0) | 3.2  (0.1) |
|  |  | **Max** | 5.1 | 4.7  (-0.4) | 5.1  (0.0) | 5.1  (0.0) | 5.1  (0.0) | 5.1  (0.0) | 5.1  (0.0) | 5.1  (0.0) |
| **Long reliable** | **3.00-3.49** | **Min** | 0.0 | 0.3  (0.3) | 0.0  (0.0) | 0.0  (0.0) | 0.0  (0.0) | 0.0  (0.0) | 0.0  (0.0) | 0.0  (0.0) |
|  |  | **Average** | 2.9 | 3.5  (0.6) | 2.9  (0.0) | 3.1  (0.2) | 2.9  (0.0) | 2.9  (0.0) | 3.2  (0.3) | 2.4  (-0.5) |
|  |  | **Max** | 5.9 | 6.8  (0.9) | 5.9  (0.0) | 6.2  (0.3) | 5.9  (0.0) | 5.9  (0.0) | 6.5  (0.6) | 4.7  (-1.2) |
|  | **3.50-3.99** | **Min** | 0.5 | 0.5  (0.0) | 0.3  (-0.2) | 0.8  (0.3) | 0.5  (0.0) | 0.3  (-0.2) | 0.5  (0.0) | 1.0  (0.5) |
|  |  | **Average** | 0.5 | 0.5  (0.0) | 0.3  (-0.2) | 0.8  (0.3) | 0.5  (0.0) | 0.3  (-0.2) | 0.5  (0.0) | 1.0  (0.5) |
|  |  | **Max** | 0.5 | 0.5  (0.0) | 0.3  (-0.2) | 0.8  (0.3) | 0.5  (0.0) | 0.3  (-0.2) | 0.5  (0.0) | 1.0  (0.5) |
|  | **4.00-4.49** | **Min** | n.a. | n.a. | n.a. | n.a. | n.a. | n.a. | n.a. | n.a. |
|  |  | **Average** | n.a. | n.a. | n.a. | n.a. | n.a. | n.a. | n.a. | n.a. |
|  |  | **Max** | n.a. | n.a. | n.a. | n.a. | n.a. | n.a. | n.a. | n.a. |
| **At the NOE limit** | **4.50-4.99** | **Min** | n.a. | n.a. | n.a. | n.a. | n.a. | n.a. | n.a. | n.a. |
|  |  | **Average** | n.a. | n.a. | n.a. | n.a. | n.a. | n.a. | n.a. | n.a. |
|  |  | **Max** | n.a. | n.a. | n.a. | n.a. | n.a. | n.a. | n.a. | n.a. |
|  | **0.00-4.99** | **Min** | 0.0 | 0.3  (0.3) | 0.0  (0.0) | 0.0  (0.0) | 0.0  (0.0) | 0.0  (0.0) | 0.0  (0.0) | 0.0  (0.0) |
|  |  | **Average** | 3.5 | 3.5  (0.0) | 3.7  (0.2) | 3.7  (0.2) | 3.5  (0.0) | 3.5  (0.0) | 3.6  (0.1) | 3.6  (0.1) |
|  |  | **Max** | 11.7 | 11.3  (-0.4) | 12.2  (0.5) | 12.2  (0.5) | 11.7  (0.0) | 11.7  (0.0) | 12.2  (0.5) | 12.2  (0.5) |

**Table S37**. Average absolute error of the quantified distances based on all 7 spectra recorded at different mixing times (100-700 ms) for the EASY ROESY SS (roesyadesjsph). Same distances quantified from the same spectra excluding one of the mixing times. The difference between the original average absolute error and average absolute errors obtained when including only 6 different mixing times are given in-between brackets.

|  | **Distance range (Å)** |  | **Error** | **Removed mixing time** | | | | | | |
| --- | --- | --- | --- | --- | --- | --- | --- | --- | --- | --- |
|  |  |  | **All** | **50** | **100** | **150** | **200** | **250** | **300** | **350** |
| **Short reliable** | **0.00-1.99** | **Min** | 1.1 | 1.1  (0.0) | 1.7  (0.6) | 1.1  (0.0) | 1.1  (0.0) | 1.1  (0.0) | 1.1  (0.0) | 1.7  (0.6) |
|  |  | **Average** | 2.8 | 2.6  (-0.2) | 3.1  (0.3) | 2.8  (0.0) | 2.8  (0.0) | 2.8  (0.0) | 2.8  (0.0) | 3.4  (0.6) |
|  |  | **Max** | 4.5 | 4.0  (-0.5) | 4.5  (0.0) | 4.5  (0.0) | 4.5  (0.0) | 4.5  (0.0) | 4.5  (0.0) | 5.1  (0.6) |
|  | **2.00-2.49** | **Min** | 2.4 | 0.8  (-1.6) | 2.4  (0.0) | 2.4  (0.0) | 2.4  (0.0) | 2.4  (0.0) | 2.4  (0.0) | 2.8  (0.4) |
|  |  | **Average** | 5.3 | 4.6  (-0.7) | 5.4  (0.1) | 5.3  (0.0) | 5.3  (0.0) | 5.3  (0.0) | 5.3  (0.0) | 5.3  (0.0) |
|  |  | **Max** | 14.9 | 12.3  (-2.6) | 15.3  (0.4) | 14.9  (0.0) | 14.9  (0.0) | 14.5  (-0.4) | 14.9  (0.0) | 15.7  (0.8) |
|  | **2.50-2.99** | **Min** | 0.0 | 0.0  (0.0) | 0.0  (0.0) | 0.0  (0.0) | 0.0  (0.0) | 0.0  (0.0) | 0.0  (0.0) | 0.4  (0.4) |
|  |  | **Average** | 2.0 | 2.2  (0.2) | 2.0  (0.0) | 2.0  (0.0) | 2.0  (0.0) | 2.1  (0.1) | 2.0  (0.0) | 2.0  (0.0) |
|  |  | **Max** | 4.4 | 4.1  (-0.3) | 4.4  (0.0) | 4.4  (0.0) | 4.4  (0.0) | 4.4  (0.0) | 4.4  (0.0) | 4.4  (0.0) |
| **Long reliable** | **3.00-3.49** | **Min** | 1.2 | 3.2  (2.0) | 0.9  (-0.3) | 1.2  (0.0) | 1.2  (0.0) | 1.5  (0.3) | 1.2  (0.0) | 0.0  (-1.2) |
|  |  | **Average** | 5.2 | 6.5  (1.3) | 5.2  (0.0) | 5.3  (0.1) | 5.2  (0.0) | 5.4  (0.2) | 5.2  (0.0) | 4.6  (-0.6) |
|  |  | **Max** | 10.8 | 10.8  (0.0) | 11.1  (0.3) | 11.1  (0.3) | 10.8  (0.0) | 10.8  (0.0) | 10.8  (0.0) | 11.1  (0.3) |
|  | **3.50-3.99** | **Min** | 0.0 | 1.5  (1.5) | 0.3  (0.3) | 0.0  (0.0) | 0.0  (0.0) | 0.3  (0.3) | 0.3  (0.3) | 1.3  (1.3) |
|  |  | **Average** | 3.2 | 3.8  (0.6) | 3.3  (0.1) | 3.2  (0.0) | 3.2  (0.0) | 3.4  (0.2) | 3.3  (0.1) | 3.3  (0.1) |
|  |  | **Max** | 6.5 | 8.8  (2.3) | 6.2  (-0.3) | 6.2  (-0.3) | 6.5  (0.0) | 7.0  (0.5) | 7.0  (0.5) | 5.5  (-1.0) |
|  | **4.00-4.49** | **Min** | 2.3 | 3.4  (1.1) | 2.3  (0.0) | 2.0  (-0.3) | 2.3  (0.0) | 2.7  (0.5) | 1.8  (-0.5) | 0.9  (-1.4) |
|  |  | **Average** | 3.3 | 4.5  (1.2) | 3.2  (-0.1) | 3.0  (-0.3) | 3.3  (0.0) | 3.4  (0.1) | 3.2  (-0.1) | 2.3  (-1.0) |
|  |  | **Max** | 4.4 | 5.7  (1.3) | 4.2  (-0.2) | 3.9  (-0.5) | 4.4  (0.0) | 4.2  (-0.2) | 4.7  (-0.3) | 3.7  (-0.7) |
| **At the NOE limit** | **4.50-4.99** | **Min** | n.a. | n.a. | n.a. | n.a. | n.a. | n.a. | n.a. | n.a. |
|  |  | **Average** | n.a. | n.a. | n.a. | n.a. | n.a. | n.a. | n.a. | n.a. |
|  |  | **Max** | n.a. | n.a. | n.a. | n.a. | n.a. | n.a. | n.a. | n.a. |
|  | **0.00-4.99** | **Min** | 0.0 | 0.0  (0.0) | 0.0  (0.0) | 0.0  (0.0) | 0.0  (0.0) | 0.0  (0.0) | 0.0  (0.0) | 0.0  (0.0) |
|  |  | **Average** | 3.8 | 3.9  (0.1) | 3.9  (0.1) | 3.8  (0.0) | 3.8  (0.0) | 3.9  (0.1) | 3.9  (0.1) | 3.8  (0.0) |
|  |  | **Max** | 14.9 | 12.3  (-2.6) | 15.3  (0.4) | 14.9  (0.0) | 14.9  (0.0) | 14.5  (-0.4) | 14.9  (0.0) | 15.7  (0.8) |

**5. References**

[1]	J. C. Carter, G. W. Luther, T. C. Long, *J. Magn. Reson.* **1974**, *15*, 122-131.

[2]	C. P. Butts, C. R. Jones, E. C. Towers, J. L. Flynn, L. Appleby, N. J. Barron, *Org. Biomol. Chem.* **2011**, *9*, 177-184.

[3]	C. P. Butts, C. R. Jones, J. N. Harvey, *Chem. Commun.* **2011**, *47*, 1193-1195.

1. Instead of roesyadesjsph, the use of roesyadjsph is recommended when no solvent suppression is necessary. The roesyadesjsph includes two additional 180 degree pulses, adding 8 ms when the magnetization is transverse, possibly allowing for T2 relaxation and the development of coupling artefacts.
